# Targeted school‐based interventions for improving reading and mathematics for students with or at risk of academic difficulties in Grades K‐6: A systematic review

**DOI:** 10.1002/cl2.1152

**Published:** 2021-04-06

**Authors:** Jens Dietrichson, Trine Filges, Julie K. Seerup, Rasmus H. Klokker, Bjørn C. A. Viinholt, Martin Bøg, Misja Eiberg

**Affiliations:** ^1^ VIVE—The Danish Center for Social Science Research Copenhagen Denmark; ^2^ Lundbeck A/S, Copenhagen Copenhagen Denmark

## Abstract

**Background:**

Low levels of numeracy and literacy skills are associated with a range of negative outcomes later in life, such as reduced earnings and health. Obtaining information about effective interventions for children with or at risk of academic difficulties is therefore important.

**Objectives:**

The main objective was to assess the effectiveness of interventions targeting students with or at risk of academic difficulties in kindergarten to Grade 6.

**Search Methods:**

We searched electronic databases from 1980 to July 2018. We searched multiple international electronic databases (in total 15), seven national repositories, and performed a search of the grey literature using governmental sites, academic clearinghouses and repositories for reports and working papers, and trial registries (10 sources). We hand searched recent volumes of six journals and contacted international experts. Lastly, we used included studies and 23 previously published reviews for citation tracking.

**Selection Criteria:**

Studies had to meet the following criteria to be included:

- Population: The population eligible for the review included students attending regular schools in kindergarten to Grade 6, who were having academic difficulties, or were at risk of such difficulties.

- Intervention: We included interventions that sought to improve academic skills, were conducted in schools during the regular school year, and were targeted (selected or indicated).

- Comparison: Included studies used an intervention‐control group design or a comparison group design. We included randomised controlled trials (RCT); quasi‐randomised controlled trials (QRCT); and quasi‐experimental studies (QES).

- Outcomes: Included studies used standardised tests in reading or mathematics.

- Setting: Studies carried out in regular schools in an OECD country were included.

**Data Collection and Analysis:**

Descriptive and numerical characteristics of included studies were coded by members of the review team. A review author independently checked coding. We used an extended version of the Cochrane Risk of Bias tool to assess risk of bias. We used random‐effects meta‐analysis and robust‐variance estimation procedures to synthesise effect sizes. We conducted separate meta‐analyses for tests performed within three months of the end of interventions (short‐term effects) and longer follow‐up periods. For short‐term effects, we performed subgroup and moderator analyses focused on instructional methods and content domains. We assessed sensitivity of the results to effect size measurement, outliers, clustered assignment of treatment, risk of bias, missing moderator information, control group progression, and publication bias.

**Results:**

We found in total 24,414 potentially relevant records, screened 4247 of them in full text, and included 607 studies that met the inclusion criteria. We included 205 studies of a wide range of intervention types in at least one meta‐analysis (202 intervention‐control studies and 3 comparison designs). The reasons for excluding studies from the analysis were that they had too high risk of bias (257), compared two alternative interventions (104 studies), lacked necessary information (24 studies), or used overlapping samples (17 studies). The total number of student observations in the analysed studies was 226,745. There were 93% RCTs among the 327 interventions we included in the meta‐analysis of intervention‐control contrasts and 86% were from the United States. The target group consisted of, on average, 45% girls, 65% minority students, and 69% low‐income students. The mean Grade was 2.4. Most studies included in the meta‐analysis had a moderate to high risk of bias.

The overall average effect sizes (ES) for short‐term and follow‐up outcomes were positive and statistically significant (ES = 0.30, 95% confidence interval [CI] = [0.25, 0.34] and ES = 0.27, 95% CI = [0.17, 0.36]), respectively). The effect sizes correspond to around one third to one half of the achievement gap between fourth Grade students with high and low socioeconomic status in the United States and to a 58% chance that a randomly selected score of an intervention group student is greater than the score of a randomly selected control group student.

All measures indicated substantial heterogeneity across short‐term effect sizes. Follow‐up outcomes pertain almost exclusively to studies examining small‐group instruction by adults and effects on reading measures. The follow‐up effect sizes were considerably less heterogeneous than the short‐term effect sizes, although there was still statistically significant heterogeneity.

Two instructional methods, peer‐assisted instruction and small‐group instruction by adults, had large and statistically significant average effect sizes that were robust across specifications in the subgroup analysis of short‐term effects (ES around 0.35–0.45). In meta‐regressions that adjusted for methods, content domains, and other study characteristics, they had significantly larger effect sizes than computer‐assisted instruction, coaching of personnel, incentives, and progress monitoring. Peer‐assisted instruction also had significantly larger effect sizes than medium‐group instruction. Besides peer‐assisted instruction and small‐group instruction, no other methods were consistently significant across the analyses that tried to isolate the association between a specific method and effect sizes. However, most analyses showed statistically significant heterogeneity also within categories of instructional methods.

We found little evidence that effect sizes were larger in some content domains than others. Fractions had significantly higher associations with effect sizes than all other math domains, but there were only six studies of interventions targeting fractions. We found no evidence of adverse effects in the sense that no method or domain had robustly negative associations with effect sizes.

The meta‐regressions revealed few other significant moderators. Interventions in higher Grades tend to have somewhat lower effect sizes, whereas there were no significant differences between QES and RCTs, general tests and tests of subdomains, and math tests and reading tests.

**Authors’ Conclusions:**

Our results indicate that interventions targeting students with or at risk of academic difficulties from kindergarten to Grade 6 have on average positive and statistically significant short‐term and follow‐up effects on standardised tests in reading and mathematics. Peer‐assisted instruction and small‐group instruction are likely to be effective components of such interventions.

We believe the relatively large effect sizes together with the substantial unexplained heterogeneity imply that schools can reduce the achievement gap between students with or at risk of academic difficulties and not‐at‐risk students by implementing targeted interventions, and that more research into the design of effective interventions is needed.

## 1 PLAIN LANGUAGE SUMMARY

### 1.1 Targeted school‐based interventions improve achievement in reading and maths for at‐risk students in Grades K‐6

School‐based interventions that target students with, or at risk of, academic difficulties in kindergarten to Grade 6 have positive effects on reading and mathematics. The most effective interventions include peer‐assisted instruction and small‐group instruction by adults. These have substantial potential to decrease the achievement gap.

**What is the aim of this review?**

This Campbell systematic review examines the effects of targeted school‐based interventions on standardised tests in reading and mathematics. The review analyses evidence from 205 studies, 186 of which are randomised controlled trials.

### 1.2 What is this review about?

Low levels of mathematics and reading skills are associated with a range of negative outcomes in life, including reduced employment and earnings, and poor health. This review examines the impact of a broad range of school‐based interventions that specifically target students with or at risk of academic difficulties in Grades K‐6. The students in this review either have academic difficulties or are at risk of such difficulties because of their background.

Examples of interventions that are included in this review are peer‐assisted instruction, using financial and non‐financial incentives, instruction by adults to small or medium‐sized groups of students, monitoring progress, using computer‐assisted instruction, and providing coaching to teachers.

Some interventions target specific domains in reading and mathematics such as reading comprehension, fluency, number sense, and operations, while others also focus on building different skills, for example, meta‐cognition and social‐emotional learning.

The review looks at whether these interventions are effective in improving students’ performance on standardised tests of reading and/or mathematics.

### 1.3 What studies are included?

In total, 607 studies are included in this review. However, only 205 of these were of sufficiently high methodological quality to be included in the analysis. Of these, 175 are from the United States, 10 from Sweden, 7 from the United Kingdom, 3 from the Netherlands, 2 from Australia, 2 from Germany, 2 from New Zealand, and 1 each from Canada, Denmark, Ireland, and Israel.

### 1.4 Do targeted school‐based interventions improve reading and mathematics outcomes?

Yes. High‐quality evidence shows that, on average, school‐based interventions aimed at students who are experiencing, or at risk of, academic difficulties, do improve reading and mathematics outcomes in the short term.

### 1.5 What type of intervention is the most effective?

Two instructional methods stand out as being particularly and consistently effective. Both peer‐assisted instruction and small‐group instruction by adults showed the largest (short‐term) improvements in reading and mathematics. Other instructional methods showed smaller improvements however, there is substantial variation in the magnitude of these effects.

### 1.6 Are positive effects sustained in the longer term?

Follow‐up outcomes measured more than three months after the end of the intervention pertain almost exclusively to studies examining small‐group instruction and reading. There is evidence of fadeout but positive effects are still reported up to 2 years after the end of intervention. Only five studies measured intervention effects after more than 2 years.

### 1.7 What do the findings of the review mean?

School‐based interventions in Grades K‐6 can improve reading and mathematics outcomes for students with or at risk of academic difficulties. In particular, the evidence shows that using peer‐assisted instruction and small‐group instruction are two of the most effective approaches that schools can implement. These interventions make a real difference in the achievement gap for at risk students.

At the same time, we need more research to better understand why interventions work better in some contexts compared with others. We also need to know more about the long‐term effects of interventions, and of interventions implemented in other countries than the United States. Furthermore, there are fewer studies of mathematics interventions than reading interventions.

### 1.8 How up‐to‐date is this review?

The review authors searched for studies up to July 2018.

## 2 BACKGROUND

### 2.1 Description of the condition

International research has consistently shown that low academic achievement during primary school increases the risk of school dropout, and additionally decreases prospects of secondary or higher education (Berktold et al., 1998; Ensminger & Slausarcick, 1992; Finn et al., 2005; Gardnier et al., 1997; Goldschmidt & Wang, 1999; Randolph et al., 2004; Winding et al., 2013). Entering adulthood with a low level of education is associated with reduced employment prospects as well as limited possibilities for financial progression in adult life (De Ridder et al., 2012; Johnson et al., 2010; OECD, 2012; Scott & Bernhardt, 2000). Furthermore, adults with higher levels of educational attainment are more likely to live longer, show higher levels of civic engagement, and exhibit greater satisfaction with life (OECD, 2010a, 2012). Conversely, low levels of education are negatively correlated with numerous health‐related issues and risk behaviours such as drug use and crime, which have serious implications for the individual as well as for society (Berridge et al., 2001; Brook et al., 2008; Feinstein et al., 2006; Horwood et al., 2010; Sabates et al., 2013).

Overall, in the member countries of the Organisation for Economic Co‐operation and Development (OECD), almost one in five of all youth between 25‐34 years of age have not earned the equivalent of a high‐school degree/upper secondary education (OECD, 2013). Moreover, on average across the OECD countries, around 15% of 18‐24 year‐olds are neither employed, nor in education or training (OECD, 2018). The Programme for International Student Achievement (PISA) tests show that on average about 20%–25% of 15‐year‐olds in the OECD countries are not proficient readers (OECD, 2010b, 2016, 2019).^1^ Likewise, in mathematics, around 20%–25% of students could only manage the lowest level in the PISA test (OECD, 2010b, 2016, 2019).^2^ These results indicate that a large proportion of students do not obtain sufficient academic skills in school and stands outside the labour market, once they have left school.

Skill differences between groups of students with low and high risk of ending up with academic difficulties appear early and are often present already before primary school. For example, struggling readers tend to be persistently behind their peers from the early Grades (e.g., Elbro & Petersen, 2004; Francis et al., 1996) and early math and language abilities strongly predict later academic achievement (e.g., Duncan et al., 2007; Golinkoff et al., 2019). Low‐income preschool children have more behaviour problems (e.g., Huaqing & Kaiser, 2003) and there is a strong continuity between emotional and behavioural problems in preschool and psychopathology in later childhood (Link Egger & Angold, 2006). Emotional and behavioural problems are in turn linked to lower academic achievement in school (e.g., Durlak et al., 2011; Taylor et al., 2017). Lastly, the gap between majority and minority children on cognitive skills tests is large already when children are 3–4 years old (e.g., Burchinal et al., 2011; Fryer & Levitt, 2013).

The prenatal and early childhood environment appears to be an important factor that keeps students from realising their academic potential (e.g., Almond et al., 2018). Currie (2009) furthermore documented that children from families with low socioeconomic status (SES) have worse health, including measures of foetal conditions, physical health at birth, incidence of chronic conditions, and mental health problems. Immigrant and minority children are often overrepresented among low SES families and face similar risks (e.g., Bradley & Corwyn, 2002; Deater‐Deckard et al., 1998; Morgan et al., 2012).

Family environments also differ in aspects thought to affect educational achievement: low SES families are less likely to provide a rich language and literacy environment (Bus et al., 1995; Golinkoff et al., 2019; Hart & Risley, 2003). The parenting practices and access to resources such as early childhood education and intervention, health care, nutrition, and enriching spare‐time activities also differ between high‐ and low‐risk groups (Esping‐Andersson et al., 2012; Morgan et al., 2012). Low SES parents also seem to have lower academic expectations for their children (Bradley & Corwyn, 2002; Slates et al., 2012), and teachers often have lower expectations for low SES and minority students (e.g., Good et al., 2003; Timperley & Phillips, 2003). Low SES children are also more likely to experience a decline in motivation during the course of primary, secondary, and upper secondary school (Archambault et al., 2010).

The neighbourhoods that students grow up in is another potential determinant of achievement (e.g., Björklund & Salvanes, 2011; Campbell et al., 2000; Chetty et al., 2018). It seems likely that many students in high‐risk groups live in neighbourhoods that are less supportive of academic achievement in terms of, for example, peer support and role models. To get by in a disadvantaged neighbourhood may also require a very different set of skills compared with what is needed to thrive in school, something that may increase the risk that pupils have trouble decoding the “correct” behaviour in educational environments (e.g., Heller et al., 2017).

As indicated by the previous discussion, the group of students experiencing academic difficulties is diverse. It includes for instance students with learning disabilities, students who are struggling because they lack family support, because they have emotional or behavioural problems, or because they are learning the first language of the country they are living in. Some groups of students may not currently have academic difficulties but are “at risk” in the sense that they are more in danger of ending up with difficulties in the future, at least in the absence of intervention (McWhirter et al., 2004). Although being at risk points to a future negative situation, it is sometimes used to designate a current situation (McWhirter et al., 2004; Tidwell & Corona Garret, 1994), as current academic difficulties are a risk factor for future difficulties and having difficulties in one area may be a risk factor in other areas (McWhirter et al., 1994).

After this review of risk factors for academic difficulties, it is worth noting that the life circumstances placing children and youth at risk are only partially predictive. That is, risk factors increase the probability of having academic difficulties, but are not deterministic. As academic difficulties therefore cannot be perfectly predicted and may show up relatively late in a child's life, interventions in early childhood may not be enough and effective interventions during school may be needed to reduce the achievement gaps substantially.

As the test score gaps between high‐ and low‐risk groups remain relatively stable from the early grades, schools do not seem to be a major reason for the inequality in academic achievement (e.g., Heckman, 2006; Lipsey et al., 2012; von Hippel et al., 2018). Further evidence is provided by the seasonality in achievement gaps. In the United States, the gap between high and low SES students tends to widen during summer breaks when schools are out of session (e.g., Alexander et al., 2001; Gershenson, 2013; Kim & Quinn, 2013; although von Hippel et al., 2018, show that this pattern is not universal across risk groups, grades and cohorts). However, the stability of the test score gaps over time also implies that current school practice is not, in general, enough to decrease the achievement gaps. As schools are perhaps the societal arena where most children can be affected by attempts to reduce the gaps, finding effective school‐based interventions for students with or at risk of academic difficulties is a question of major importance.

Information about effective interventions for students with or at risk of academic difficulties is also of significant interest in most countries. This interest has been reflected in increased political initiatives such as the European Union (EU) Strategic Framework for Education and Training (The Council of the European Union, 2009), or comprehensive legislation such as the No Child Left Behind Act from 2001 in the United States (U.S. Congress, 2002; U.S. Department of Education, 2004).

The research on interventions aimed at academic achievement is rapidly growing, and the interventions described in the literature are numerous and very diverse in terms of for example intervention focus, target group, and delivery mode. The current review focused on targeted, school‐based interventions provided to students in kindergarten (K) to Grade 6 (ages range from 5–7 to 11–13, depending on country/state), where academic learning and skill building were the intervention aims. The outcome variables were standardised tests of achievement in reading and mathematics.

In line with the diversity of reasons for ending up with a low level of skills and educational attainment, we included interventions targeting students who for a broad range of reasons were having academic difficulties, or were at risk of such difficulties. We prioritised already having difficulties over belonging to an at‐risk group in the sense that if there was information about for example test scores and grade point averages, we did not require information about at‐risk status. Furthermore, we did not include interventions targeting high‐performing students in groups that may otherwise be at risk.

This review shares the aims, most inclusion criteria, and the search and screening process with another review about interventions for students in Grades 7–12 (Dietrichson et al., 2020). Consequently, some of the sections below are very similar and a reader that has already read that review may want to skip some parts of the background and method sections.

### 2.2 Description of the intervention

We included interventions that were targeted to students with or at risk of academic difficulties (i.e., interventions that were selected or indicated) and aimed to improve the students’ academic achievement. Targeted interventions can be delivered in various settings, including in class (e.g., peer‐assisted instruction interventions), in group sessions (e.g., the READ180 programme), or one‐to‐one. We restricted the settings to school‐based interventions, by which we mean interventions implemented in school, during the regular school year, and in which schools were one of the stakeholders. This restriction excluded for example after‐school programmes, summer camps and summer reading programmes, and interventions involving only parents and families (see e.g., Zief et al., 2006 for a review of after‐school programmes; Kim & Quinn, 2013, for a review of summer reading programmes; and Jeynes, 2012, for a review of programmes that involve families or parents).

We included a wide range of interventions that aimed to improve the academic achievement of students by changing the method of instruction—such as tutoring, peer‐assisted instruction, and computer‐assisted instruction interventions—or by changing the content of the instruction—for instance, interventions emphasising mathematical problem‐solving skills, reading comprehension, and meta‐cognitive and social‐emotional skills. Many interventions involved changes to both method and content, and included several major components. That is, we included interventions based on their aim to improve academic achievement and based on interventions targeting students with or at risk of academic achievement, and not based on the type of components used in the intervention.

Therefore, we excluded interventions that may improve academic achievement as a side effect, but did not have academic achievement as an aim. Examples are interventions where behavioural or social‐emotional problems were the primary intervention aim. However, interventions with behavioural and social‐emotional components may very well have academic achievement as one of their primary aims, and use standardised tests of reading and mathematics as one of their primary outcomes. Such interventions were included.

Universal interventions applied to improve the quality of the common learning environment at school in order to raise academic performance of all students (including average and above average students) were excluded. We also excluded whole‐school reform strategy concepts such as Success for All, as well as reduced class size interventions and general professional development interventions for principals and teachers that did not target at‐risk students. However, we included some interventions with a professional development component, for example, in the form of coaching of teachers during the implementation of the intervention, as long as the intervention specifically targeted students with or at risk of academic difficulties.

### 2.3 How the intervention might work

All the included interventions strove to improve academic achievement for students with or at risk of academic difficulties. However, they did so with different approaches and with diverse strategies of how to create that improvement. This diversity reflects the varying reasons for why students are struggling or are at risk. In turn, the theoretical background for the interventions varied accordingly. It is therefore not possible to specify one particular theory of change or one theoretical framework for this review. Instead, we briefly review three theoretical perspectives that we believe are characteristic for the majority of the included interventions. We then discuss and exemplify how existing targeted interventions may address some of the reasons for academic difficulties mentioned in Section 2.1 in the light of the theoretical perspectives.

#### 2.3.1 Theoretical perspectives

The reasons why students may be struggling laid out in the previous section are multifaceted, and the theoretical perspectives underlying the included interventions are broad. Nevertheless, three superordinate components are characteristic for the majority of the included programmes:

- Adaptation of behaviour (social learning theory).
- Individual cognitive learning (cognitive developmental theory).
- Alteration of the social learning environment (pedagogical theory).

We emphasise that the following presentation of these three theoretical perspectives is not exhaustive, and, although components are presented as demarcated, they contain some conceptual overlap.

*Social learning theory* has its origins in social and personality psychology, and was initially developed by psychologist Julian Rotter and further developed especially by Albert Bandura, (1977, 1986). From the perspective of social learning theory, behaviour and skills are primarily learned by observing and imitating the actions of others, and behaviour is in turn regulated by the recognition of those actions by others (reinforcement), or discouraged by lack of recognition or sanctions (punishment). According to social learning theory, creating the right social context for the student can therefore stimulate more productive behaviour through social modelling and reinforcement of certain behaviours that can lead to higher academic achievement.

*Cognitive developmental theory* is not one particular theory, but rather a myriad of theories about human development that focus on how cognitive functions such as language skills, comprehension, memory and problem‐solving skills enable students to think, act and learn in their social environment. Some theories emphasise a concept of intelligence where children gradually come to acquire, construct, and use cognitive functions as the child naturally matures with age (e.g., Piaget, 2001; Perry, 1999). Other theories hold a more socio‐cultural view of cognitive development and use a more culturally distinct and individualised concept of intelligence that to a greater extent includes social interaction and individual experience as the basis for cognitive development. Examples include the theories of Robert Sternberg (2009) and Howard Gardner (1999).

*Pedagogical theory* draws on the different disciplines in psychology and social theory such as cognitivism, social‐interactional theory and socio‐cultural theory of learning and development. There is not one uniform pedagogical model, but examples of contemporary models in mainstream pedagogy are concepts such as Scaffolding (Bruner, 2006) and the Zone of Proximal Development (Vygotsky, 1978), which originated in developmental and educational psychology. These notions hold that learning and development emerge through practical activity and interaction. Acquisition of new knowledge is therefore considered to be dependent on social experience and previous learning, as well as the availability and type of instruction. Accordingly, school interventions require educators to interact and organise the learning environment for the student in certain ways to fit the individual student's needs and potentials for development.

#### 2.3.2 Interventions in practice

School interventions affect academic achievement by changing the methods by which instruction is given (instructional methods) and by targeting certain content (the content domain), and many combine several intervention components as well as theoretical perspectives. Examples of instructional methods covered in earlier reviews are tutoring, coaching of personnel, cooperative learning/peer‐assisted instruction, computer‐assisted instruction, feedback and progress monitoring, and incentive programmes (e.g., Dietrichson et al., 2017). Reading interventions directed to younger students often target content domains as phonemic awareness, phonics, fluency, vocabulary, and comprehension (e.g., Slavin et al., 2009). Slavin and Lake (2008) describe differences in elementary school math curricula in terms of how they emphasise domains such as problem solving, manipulatives, concept development, algorithms, computation and word problems. Gersten et al. (2009) used the following domains to divide mathematics interventions into categories: operations (e.g., addition, subtraction, and multiplication), word problems, fractions, algebra, and general math proficiency (or multiple components). Many school interventions have additional goals concerning other aspects of the student's life, such as reducing problematic behaviour of the students (Cheung & Slavin, 2012; Slavin & Lake, 2008; Wasik, 1997; Wasik & Slavin, 1993).

As indicated, many interventions combine theoretical perspectives. For example, interventions such as tutoring and peer‐assisted instruction interventions often have in common that they comprise an eclectic theoretical model that combines components from all three perspectives on learning presented in the previous section. They are comprehensive interventions that rely on mechanisms such as increased feedback and tailor‐made instruction (pedagogical theory), regulation of behaviour by for example rewards or interaction with role models (social learning theory), and development of cognitive functions such as learning how to learn (cognitive developmental theory).

Another way of viewing these and other types of interventions is that they address the differential family and neighbourhood resources of students with high and low risk of academic difficulties. Low‐risk students are more likely to have access to “tutors” all year round, as parents, siblings, and other family members help out with homework and schoolwork. Interventions to change mindsets, increase expectations, and mitigate stereotype threat may also substitute for low‐risk families and teachers already having such expectations or teaching low‐risk students such a mindset. Different types of extrinsic rewards may be a way to bolster motivation, which may be especially important for students whose families place less weight on educational achievement.

Furthermore, if the differences between students with high and low risk of academic difficulties can be understood as a consequence of differential access to a combination of resources, then remedial efforts may need to address several problems at once to be effective. Programmes that combine certain components may therefore be more effective than others. Another reason why it is interesting to examine combinations of components relates to an often suggested explanation for missing impacts: lack of motivation among participants (e.g., Edmonds et al., 2009; Fuchs et al., 1999). It is therefore possible that programmes will be more effective if they, for example, include some form of rewards for participating students, along with other components providing for instance specific pedagogical support.

### 2.4 Why it is important to do the review

In this section, we first discuss earlier related reviews, and then the contributions of our review in relation to the earlier literature. We focus on reviews that, like our review, compared types of interventions in terms of either instructional methods or content domains.

#### 2.4.1 Prior reviews

Prior reviews have in particular covered reading interventions. Slavin et al. (2009) reviewed reading programmes for elementary Grades. They focused on all kinds of programmes and not only programmes for at‐risk or low‐performing students specifically. Wanzek et al. (2006) reviewed reading programmes directed to students in Grades K‐12 with learning disabilities, and Flynn et al. (2012), Inns et al. (2019), Scammaca et al. (2015), Slavin et al. (2011), and Wanzek et al. (2018) reviewed programmes for struggling readers in Grades 5‐9, K‐5, 4‐12, K‐5, and K‐3, respectively.^3^ These reviews thus covered low‐achieving students, but neither at‐risk students nor areas other than reading. Suggate (2016) reviewed the long‐run effects of phonemic awareness, phonics, fluency, and reading comprehension interventions from preschool up to Grade 7, but did not discern between interventions targeting students with/at risk of and without/not at risk of academic difficulties.

Mathematics interventions were reviewed in Slavin and Lake (2008) and Pellegrini et al. (2018) for general student populations in elementary school. Gersten et al. (2009) examined four types of components of mathematics instruction for students with learning disabilities, but did not include studies for students at risk of math difficulties (or other reasons for difficulties than learning disabilities). Dietrichson et al. (2017) included interventions targeting both reading and mathematics and based inclusion on the share of students with low SES, but did not consider whether students had academic difficulties or not. Fryer (2017) included both math and reading interventions for all types of student groups.^4^

All reviews that reported an overall effect size found that it was positive. Most also found substantial variation between interventions. Regarding intervention types, we provide a more detailed comparison to our results in Section 6.5 (including reviews focused on a specific intervention type), but to preview that discussion we describe some overarching results here. Among instructional methods, many reviews indicated that one‐to‐one or small‐group tutoring have relatively large effect sizes across both mathematics and reading interventions compared with other intervention types (Dietrichson et al., 2017; Fryer, 2017; Inns et al., 2019; Pellegrini et al., 2018; Slavin & Lake, 2008; Slavin et al., 2009, 2011). Peer‐assisted instruction or cooperative learning interventions also showed relatively large effect sizes in some reviews (Dietrichson et al., 2017; Inns et al., 2019; Pellegrini et al., 2018; Slavin & Lake, 2008; Slavin et al., 2009, 2011), but not in all (Gersten et al., 2009). Computer‐assisted or technology‐supported instruction have typically positive but smaller effect sizes than small‐group and peer‐assisted instruction (Dietrichson et al., 2017; Inns et al., 2019; Pellegrini et al., 2018; Slavin & Lake, 2008; Slavin et al., 2009, 2011).

Gersten et al. (2009) examined some components of mathematics instruction that do not map neatly into the categories used in the current review and some of the others. They found for example most support for explicit instruction, use of heuristics, and curriculum design. Regarding specific math domains, interventions targeting word problems had higher effect sizes than other math domains but not significantly so.

Reviews focusing on short‐term effects across reading domains reported positive effects in general but few reliable differences over reading domains (Flynn et al., 2012; Scammaca et al., 2015; Wanzek et al., 2006). An exception is that reading comprehension interventions were associated with significantly higher effect sizes than fluency interventions in Scammaca et al. (2015), but this difference disappeared when they only considered standardised tests. Suggate (2016) found that comprehension and phonemic awareness interventions showed relatively lasting effects that transferred to non‐targeted skills, whereas phonics and fluency interventions did not (mean follow‐up was around 11 months).

#### 2.4.2 The contribution of this review

Academic difficulties and lack of educational attainment are significant societal problems. Moreover, as shown by the Salamanca declaration from 1994 (UNESCO, 1994), there has for decades been a great interest among policy makers to improve the inclusion of students with academic difficulties in mainstream schooling, and a desire to increase the number of empirically supported interventions for these student groups.

The main objective of this review is to provide policy makers and educational decision‐makers at all levels—from governments to teachers—with evidence of the effectiveness of interventions aimed to improve the academic achievement of students with or at risk of academic difficulties. To this end, we chose a broad scope in terms of the target group and the types of interventions we included. We included studies that measured the effects of interventions by standardised tests in reading and mathematics. The reason is that many interventions are not directed specifically to either subject and outcomes are therefore measured in both (Dietrichson et al., 2017). Including both students with and at risk of academic difficulties in the target group should also decrease the risk of biasing the results due to omission of studies where information about either academic difficulties or at‐risk status is available, but not both. Furthermore, making comparisons over intervention components within one review, rather than across reviews, should increase the possibilities of a fair comparison. For instance, controlling that effect sizes are calculated in the same way, that the definitions of intervention components are consistent, and that moderators are coded in the same way, is easier within the scope of one review than across reviews.

Earlier reviews with a comparable focus on students with or at risk of academic difficulties have included a more narrowly defined target group. Furthermore, their analyses either did not include intervention components together with other moderators in a meta‐regression, or only included very broad categories of instructional methods and content domains. Such analyses risk confounding the effects of intervention components with for example participant characteristics, and precludes testing whether components have significantly different effect sizes. Furthermore, some reviews have coded interventions regarding the instructional methods used, or regarding the type of content taught, and used such indicators in meta‐regressions (e.g., Dietrichson et al., 2017; Gersten et al., 2009; Scammaca et al., 2015). With the exception of Gersten et al. (2009), who included an indicator for word problems alongside instructional methods‐indicators, the analyses did not include both methods, and content domain indicators. They therefore risk confounding instructional methods with content domains.

Lastly, we are not aware of another review that have provided meta‐analytic estimates of medium‐ and long‐term effects specifically for students with or at risk of academic difficulties.

## 3 OBJECTIVES

The primary objective of this review was to assess the effectiveness of targeted interventions aimed at improving the academic achievement for students with or at risk of academic difficulties in Grades K to 6.

The secondary objective was to examine the comparative effectiveness of different types of interventions, focusing on instructional methods and content domains. We conducted subgroup and moderator analyses in which we attempted to identify those methods and domains that have the strongest and most reliable associations with academic outcomes, as measured by standardised test scores in reading and mathematics.

The tertiary objective was to explore the evidence for differential effects across participant and study characteristics. We prioritised characteristics that were relevant for all types of interventions.

## 4 METHODS

### 4.1 Criteria for considering studies for this review

#### 4.1.1 Types of studies

According to our protocol, included studies should use an intervention‐control group design or a comparison group design (Dietrichson et al., 2016). Included study designs were randomised controlled trials (RCT), including cluster‐RCTs; quasi‐randomised controlled trials (QRCTs), that is, where participants are allocated by means such as alternate allocation, person's birth date, the date of the week or month, case number, or alphabetical order; and quasi‐experimental studies (QES). To be included, QES had to credibly demonstrate that outcome differences between intervention and control groups is the effect of the intervention and not the result of systematic baseline differences between groups. That is, selection bias should not be driving the results. This assessment is included as a part of the risk of bias tool, which we elaborate on in the “Risk of bias” section, and no QES was excluded on this criterion in the screening process. A fair amount of studies within educational research use single group pre–post comparisons (e.g., Edmonds et al., 2009; Wanzek et al., 2006); such studies were however excluded in the screening process due to the higher risk of bias.

Control groups received treatment‐as‐usual (TAU) or a placebo treatment. We found no studies in which the control group explicitly received nothing (i.e., a no‐treatment control), as all students experienced regular schooling. That is, control groups got whatever instruction the intervention group would have gotten, had there not been an intervention. The TAU condition can for this reason differ substantially between studies (although many studies did not describe the control condition in much detail). Eligible types of control groups included also waiting list control groups, which only differed in the time frame in which researchers estimate the effects. That is, students in both waiting list and regular control groups were offered regular schooling but after the students in the waiting list control group had received the intervention, they could no longer be used as controls.

Comparison designs compared alternative interventions against each other. That is, they made it clear that all students get something other than TAU *because* of the intervention. Effect sizes from such studies are not fully comparable to effect sizes from intervention‐control designs. We therefore planned to analyse comparison designs separately from intervention‐control designs, and use them where they may shed light on an issue, which could not be fully analysed using the sample of intervention‐control studies. However, the number of studies that were, in this sense, relevant was small and we used them only in one analysis of the effects of group sizes in small‐group instruction interventions.

Due to language restrictions in the review team, we included studies written in English, German, Danish, Norwegian, and Swedish. To ensure a certain degree of comparability between school settings and to align TAU conditions in included studies, we only included studies published in or after 1980.

#### 4.1.2 Types of participants

The population samples eligible for the review included students attending regular schools in Grades K‐6, who were having academic difficulties, or were at risk of such difficulties. Students attending regular private, public, and boarding schools were included, and students receiving special education services within these school settings were also included.

We included only studies carried out in OECD countries. This selection made it more likely that school settings and TAU conditions were comparable across included studies. Grades K‐6 corresponds roughly to primary school, defined as the first step in a three‐tier educational system consisting of primary education, secondary education and tertiary or higher education. We included studies with a student population in higher Grades than K‐6 as long as the majority of the students were in Grades K‐6. The age range included differed between countries, and sometimes between states within countries (ages range from 4–7 to 11–13, depending on country/state). Much fewer studies reported the participants’ ages than Grades, which was also our main reason to formulate the inclusion criteria in terms of Grade rather than age.

The eligible student population included both students identified in the studies by their observed academic achievement (e.g., low academic test results, low grade point average or students with specific academic difficulties such as learning disabilities), and students that were identified primarily on the basis of their educational, psychological, or social background (e.g., students from families with low socioeconomic status, students placed in care, students from minority ethnic/cultural backgrounds, and second language learners). We excluded interventions that only targeted students with physical learning disabilities (e.g., blind students), students with dyslexia/dyscalculia, and interventions that were specifically directed towards students with a certain neuropsychiatric disorder (e.g., autism, ADHD), as some interventions targeting such students are different from interventions targeting the general struggling or at‐risk student population (e.g., they include medical treatments like in Ackerman et al., 1991).

Because there was substantial overlap between students that were already struggling and groups considered at‐risk of difficulties in studies found in a previous review (Dietrichson et al., 2017), we chose to include both students with difficulties and students that were deemed at‐risk, or were considered educationally disadvantaged. If the two criteria were inconsistent, we gave priority to students having academic difficulties. For example, we excluded interventions that targeted high‐achieving students from low‐income backgrounds.

Some interventions included other students, who neither had academic difficulties nor were at risk of such difficulties. For example, in some peer‐assisted learning interventions high‐performing students were paired with struggling students. Studies of such interventions were included if the total sample (intervention and control group) included at least 50% students that were either having academic difficulties or were at risk of developing such difficulties, or if there were separate effect sizes reported for these groups.

#### 4.1.3 Types of interventions

We included interventions that sought to improve academic achievement or specific academic skills. This does not mean that the intervention had to consist of academic activities, but there had to be an expectation in the study that the intervention, regardless of the nature of the intervention content, would result in improved academic achievement or a higher skill level in a specific academic task. We however choose to exclude interventions that only sought to improve performance on a single test instead of improving a skill that would improve test scores. For similar reasons, we excluded studies of interventions where students are provided with accommodations when taking tests; for instance, when some students are allowed to use calculators and others not.

An explicit academic aim of the intervention did not per se exclude interventions that also included non‐academic objectives and outcomes. However, we excluded interventions having academic learning as a possible secondary objective. If the objectives were not explicitly stated, we used the presence of a standardised test in mathematics or reading as an indication that the authors expected the intervention to improve academic achievement. We excluded cases where such tests were included but the authors explicitly stated that they did not expect the intervention to improve reading or math skills.

Furthermore, we only included school‐based interventions. That is, interventions conducted in schools during the regular school year with schools as one of the stakeholders. This latter restriction excluded summer reading programmes, after‐school programmes, parent tutoring programmes, and other programmes delivered in the home of students.

Universal interventions that aimed to improve the quality of the common learning environment at the school level in order to raise academic achievement of all students (including average and above average students), were excluded. Interventions such as the one described in Fryer (2014) where a bundle of best practices were implemented at the school level in low‐achieving schools, where most students are struggling or at risk, was also excluded. This criterion also excluded whole‐school reform strategy concepts such as Success for All, curriculum‐based programmes like Elements of Mathematics (EMP), as well as reduced class size interventions.

This criterion also meant that we excluded interventions where teachers or principals receive professional development training in order to improve general teaching or management skills. Interventions targeting students with or at risk of academic difficulties may on the other hand include a professional development component, for example, when a reading programme includes providing teachers with reading coaches. Such interventions were therefore included.

Our protocol contained no criterion for the duration of interventions and we included interventions of all durations. We coded the duration of the interventions and this variable was included as a moderator in some of the analyses.

#### 4.1.4 Types of outcome measures

We included outcomes that cover two areas of fundamental academic skills:

- Standardised tests in reading
- Standardised tests in mathematics

Studies were only included if they considered one or more of the primary outcomes. Standardised tests included norm‐referenced tests (e.g., Gates‐MacGinitie Reading Tests and Star Math), state‐wide tests (e.g., Iowa Test of Basic Skills), and national tests (e.g., National Assessment of Educational Progress, NAEP). If it was not clear from the description of the outcome measures in the studies, we used online sources to determine whether a test was standardised or not. For example, if a commercial test has been normed, this was typically mentioned on the publisher's homepage. However, for older tests it was not always possible to find information about the test from electronic sources. In these cases, we included the test if there was a reference to a publication describing the test, which made it clear that the test had not been developed for the intervention or the study.

We restricted our attention to standardised tests in part to increase the comparability between effect sizes. Earlier related reviews of academic interventions have pointed out that effect sizes tend to be significantly lower for standardised tests compared with researcher‐developed tests (e.g., Flynn et al., 2012; Gersten et al., 2009; Scammaca et al., 2015). Scammaca et al. (2015) furthermore reported that whereas mean effect sizes differed significantly between the periods 1980–2004 and 2005–2011 for other types of tests, mean effect sizes were not significantly different for standardised tests. As researcher‐developed tests are usually less comprehensive and more likely to measure aspects of content inherent to intervention but not control group instruction (Slavin & Madden, 2011), standardised tests should provide a more reliable measure of lasting differences between intervention and control groups.

We excluded tests that provided composite results for several academic subjects other than mathematics and reading, but included tests of specific domains (e.g., vocabulary, fractions) as well as more general tests, which tested several domains of reading or mathematics. Tests of subdomains had significantly larger effect sizes compared with more general tests in Dietrichson et al. (2017). This result may indicate that it may be easier to improve scores on tests of subdomains than on tests of more general skills, or that tests of subdomains may be more likely to be inherent to intervention group instruction. At the same time, it seems reasonable that interventions that target subdomains of reading and mathematics are tested on whether they affect these subdomains. Therefore, we did not want to exclude either type of test, but coded the type of test and used it as a moderator in the analysis. However, to mitigate problems with test content being inherent to intervention and not control group instruction, we did not consider tests where researchers themselves picked a subset of questions from a norm‐referenced test as being standardised. The subset should either have been predefined (as in e.g., the passage comprehension subset of Woodcock‐Johnson Tests of Achievement) or the picked by someone other than the researchers (e.g., released items from the NAEP).

We included all postintervention tests and coded the timing of each test (see “Multiple time points” section).

### 4.2 Search methods for identification of studies

This section describes the search strategy for identifying potentially relevant studies. We used the EPPI reviewer software to track the search and screening processes. A flowchart describing the search process and specific numbers of references screened on different levels can be found in Section 5.1.2. The search documentation, reporting and details relating to the search can be found in the Supporting Information Appendix A.

#### 4.2.1 Limitations and restrictions of the search strategy

All searches were restricted to publications after 1980. This year was chosen to balance the competing demands of comparability between intervention settings and comprehensiveness of the review. We used no further limiters in the searches.

#### 4.2.2 Electronic database searches

Relevant published studies were identified through electronic searches of bibliographic databases, government and policy databanks. We searched the following electronic resources/databases:

- *Academic Search Premier* (EBSCO)
- *ERIC* (EBSCO)
- *PsycINFO* (EBSCO)
- *SocIndex* (EBSCO)
- *British Education Index* (EBSCO)
- *Teacher Reference Center* (EBSCO)
- *ECONLIT* (EBSCO)
- *FRANCIS* (EBSCO)
- *CBCA Education* (ProQuest)
- *Australian Education Index* (ProQuest)
- *Social Science Citation Index* (ISI Web of Science)
- *Science Citation Index* (ISI Web of Science)
- *Medline* (OVID)
- *Embase* (OVID)

All databases were originally searched from 1st of January 1980 to March 2016. As mentioned, we only included studies published in or after 1980 to ensure a certain degree of comparability between school settings and to align TAU conditions in included studies. We updated the searches in June/July 2018 using identical search strings. Some database searches were not updated in 2018 due to access limitations.

In Supporting Information Appendix A, we report the search strings as well as details for each electronic database and resource searched.

Note that the searches contained terms relating to secondary school, since the search contributed to a review about this older age group (Grades 7–12, see Dietrichson et al., 2020). There is overlap in the literature among the age groups, and in order to rationalise and accelerate the screening process, we decided upon performing one extensive search.

#### 4.2.3 Searching other web‐based resources

We also searched the following national/international repositories and review/trial archives/registries:

- DIVA—Swedish repository for research publications and theses (http://www.diva-portal.org/smash/search.jsf?dswid=9447)
- CRISTIN—Current Research Information Systems In Norway (https://www.cristin.no/)
- Danish National Research Database (Forskningsdatabasen.dk)
- Cochrane Library (http://www.cochranelibrary.com/)
- Social Care Online (http://www.scie-socialcareonline.org.uk/)
- Centre for Reviews and Dissemination Databases (https://www.crd.york.ac.uk/CRDWeb/)
- What Works Clearinghouse—U.S. Department of Education (https://ies.ed.gov/ncee/wwc/)
- Danish Clearinghouse for Education Research (edu.au.dk/clearinghouse)

Our protocol stated that we should search two trial registries: The Institute for Education Sciences’ (IES) Registry of Randomized Controlled Trials (http://ies.ed.gov/ncee/wwc/references/registries/index.aspx), and American Economic Association's RCT Registry (https://www.socialscienceregistry.org). We were however unable to search the IES registry as it was not available (last tried 23 July 2018). We have asked IES about availability, but have to date not received a reply. We updated the search of American Economic Association's RCT Registry on 23 July 2018.

#### 4.2.4 Hand search

The following selected journals had the highest frequency of potentially relevant studies based on the initial pilot‐searches during the development of the search string and the protocol:

- *American Educational Research Journal*
- *Journal of Educational Research*
- *Journal of Educational Psychology*
- *Journal of Learning Disabilities*
- *Journal of Research on Educational Effectiveness*
- *Journal of Education for Students Placed at Risk*

The search was performed on editions from 2015 to July 2018 (i.e., including an updated search) of the journals mentioned, in order to capture relevant studies recently published and therefore not found in the systematic search.

#### 4.2.5 Grey literature searches

We performed a wide range of searches on the below institutional and governmental resources, academic clearinghouses and repositories for relevant academic theses, reports and conference/working papers. Most of the resources searched for grey literature include multiple types of references. The resources are listed under the category of literature most prevalent in the resource, even though multiple types of unpublished/published literature might be identified in the resource.

##### Search for Dissertations

- *ProQuest dissertation & theses A&I* (ProQuest)
- Theses Canada (https://www.bac-lac.gc.ca/eng/services/theses/Pages/search.aspx)

##### Search for Working Papers/Conference Proceedings

- European Educational Research Association (http://www.eera-ecer.de/).
- American Educational Research Association (http://www.aera.net/).
- German Educational Research Association (http://www.dgfe.de/en/aktuelles.html).
- NBER working paper series (http://nber.org/).

##### Search for Reports

- OpenGrey (http://www.opengrey.eu/).
- Best Evidence Encyclopedia (http://www.bestevidence.org/).
- Google Scholar (https://scholar.google.dk/).
- Google (https://www.google.dk/).

#### 4.2.6 Contacts to international experts

We contacted international experts to identify unpublished and ongoing studies. We primarily contacted corresponding authors of the related reviews mentioned in Section 2.4.1,^5^ and authors with many and/or recent included studies. The following authors replied: Douglas Fuchs, Lynn Fuchs, Russell Gersten, Nancy Scammaca, Robert Slavin, and Sharon Vaughn. Furthermore, during work with another review about the use of randomised controlled trials in Scandinavian compulsory school, authors were contacted about studies with sometimes overlapping inclusion criteria with the current review (see Pontoppidan et al., 2018).

#### 4.2.7 Citation‐tracking/snowballing strategies

In order to identify both published studies and grey literature we used citation‐tracking/snowballing strategies. Our primary strategy was to citation‐track related systematic reviews and meta‐analyses. 1446 references from 23 existing reviews were screened in order to find further relevant grey and published studies (see Section 2.4.1 and the list in Supporting Information Appendix A, subsection *Grey Literature Searches*). The review team also checked reference lists of included primary studies for new leads.

### 4.3 Data collection and analysis

#### 4.3.1 Selection of studies

Under the supervision of the review authors, at least two review team assistants independently screened titles and abstracts to exclude studies that were clearly irrelevant. Any disagreement of eligibility was resolved by the review authors. We retrieved studies considered eligible in full text. Two review team assistants then independently screened the full texts under the supervision of the review authors. Any disagreement of eligibility was resolved by the review authors. The review authors piloted the study inclusion criteria with all review team assistants.

#### 4.3.2 Data extraction and management

Two members of the review team independently coded and extracted data from included studies. A coding sheet was piloted on several studies and revised. Any disagreements were resolved by discussion, and it was possible to reach consensus in all cases. We extracted data on the characteristics of participants, characteristics of the intervention and control/comparison conditions, research design, sample size, outcomes, and results. We contacted study authors if a study did not include sufficient information to calculate an effect size. Extracted data was stored electronically, and we used EPPI Reviewer 4, Microsoft Excel, and R as the primary software tools.

#### 4.3.3 Assessment of risk of bias in included studies

We assessed the risk of bias of effect estimates using a model developed by Prof. Barnaby Reeves in association with the Cochrane Non‐Randomised Studies Methods Group. This model is an extension of the Cochrane Collaboration's risk of bias tool and covers risk of bias in non‐randomised studies that have a well‐defined control group. The extended model is organised and follows the same steps as the risk of bias model according to the 2008‐version of the Cochrane Handbook, chapter 8 (Higgins & Green, 2008). The extension to the model is explained in the three following points:

1. The extended model specifically incorporates a formalised and structured approach for the assessment of selection bias in non‐randomised studies by adding an explicit item about confounding. This is based on a list of confounders considered to be important and defined in the protocol for the review. The assessment of confounding is made using a worksheet where, for each confounder, it is marked whether the confounder was considered by the researchers, the precision with which it was measured, the imbalance between groups, and the care with which adjustment was carried out. This assessment informed the final risk of bias score for confounding.
2. Another feature of effect estimates in non‐randomised studies that make them at high risk of bias is that they need not have a protocol in advance of starting the recruitment process (this is however also true for a very large majority of RCTs in education). The item concerning selective reporting therefore also requires assessment of the extent to which analyses (and potentially, other choices) could have been manipulated to bias the findings reported, for example, choice of method of model fitting, potential confounders considered/included. In addition, the model includes two separate yes/no items asking reviewers whether they think the researchers had a prespecified protocol and analysis plan.
3. Finally, the risk of bias assessment is refined, making it possible to discriminate between effect estimates with varying degrees of risk. This refinement is achieved with the addition of a 5‐point scale for certain items (see the next section for details).

The refined assessment is pertinent when thinking of data synthesis as it operationalises the identification of studies (especially in relation to non‐randomised studies) with a very high risk of bias. The refinement increases transparency in assessment judgements and provides justification for not including a study with a very high risk of bias in the meta‐analysis.

##### Risk of bias judgement items

The risk of bias model used in this review is based on nine items (see Supporting Information Appendix B: *Risk of bias tool* for a fuller description). The nine items refer to: sequence generation, allocation concealment, blinding, incomplete outcome data, selective outcome reporting, other potential threats to validity, a priori protocol, a priori analysis plan, and confounders (for non‐randomised studies). As all but the latter follow standard procedures described in the Cochrane Handbook (Higgins & Green, 2011), we focus on the confounding item below.

##### Confounding

An important part of the risk of bias assessment of effect estimates in non‐randomised studies is how studies deal with confounding factors. Selection bias is understood as systematic baseline differences between groups and can therefore compromise comparability between groups. Baseline differences can be observable (e.g., age and gender) and unobservable to the researcher (e.g., motivation). Included studies use for example matching and statistical controls to mitigate selection bias, or demonstrate evidence of preintervention equivalence on key risk variables and participant characteristics. In each study, we assessed whether the observable confounding factors of age and Grade level, performance at baseline, gender, and socioeconomic background had been considered, and how each study dealt with unobservables.

There is no single non‐randomised study design that always deals adequately with the selection problem. Different designs represent different approaches to dealing with selection problems under different assumptions and require different types of data. For example, differences in preintervention test score levels do not have to be a major problem in a difference‐in‐differences design, where the main identifying assumption is that the trends of the outcome variable in the intervention and control group would not have differed, had the intervention not occurred (e.g., Abadie, 2005). Similar differences in levels would, in general, be more problematic in a matching design as they indicate that the matching technique has not been able to balance the sample even on observable variables. For this reason, we did not specify thresholds in terms of preintervention differences (in say, effect sizes) for when a study has too high risk of bias on confounding.

##### Importance of prespecified confounding factors

We describe the motivation for focusing on age and Grade level, performance at baseline, gender, and socioeconomic background below.

Development of cognitive functions relating to school performance and learning are age dependent. Furthermore, systematic differences in performance level often refer to systematic differences in preconditions for further development and learning of both cognitive and social character (Piaget, 2001; Vygotsky, 1978). Therefore, to be sure that an effect estimate was a result from a comparison of groups with no systematic baseline differences it was important to control for the students' Grade level (or age).

Performance at baseline is generally a very strong predictor of posttest scores (e.g., Hedges & Hedberg, 2007), and controlling for this confounder was therefore highly important.

With respect to gender it is well‐known that there exist gender differences in school performance (e.g., Holmlund & Sund, 2005). In terms of our primary outcome measures, girls tend to outperform boys with respect to reading and boys tend outperform girls with respect to mathematics (Stoet & Geary, 2013), although parts of the literature finds that these gender differences vanish over time (Hyde et al., 1990; Hyde & Linn, 1988). As there is no consensus around the disappearance of gender differences, we found it important to include this potential confounder.

Students from more advantaged socioeconomic backgrounds on average begin school better prepared to learn (e.g., Fryer & Levitt, 2013). As outlined in Section 2, students with socio‐economically disadvantaged backgrounds have lower test scores on international tests (OECD, 2010c, 2013). Therefore, the accuracy of the estimated effects of an intervention may depend on how well socioeconomic background is controlled for. Socioeconomic background factors were for example parents’ educational level, family income, and parental occupation.

##### Bias assessment in practice

At least two review authors independently assessed the risk of bias for each included study. Disagreements were resolved by discussion, and it was possible to reach a consensus in all cases. We reported the risk of bias assessment in risk of bias tables for each included study (see Supporting Information Appendices F and G).

In accordance with Cochrane and Campbell methods we did not aggregate the 5‐point scale across items. Effect sizes given a rating of 5 on any item should be interpreted as being more likely to mislead than inform and were not be included in the meta‐analysis (the items with a 3‐point scale did not warrant exclusion). If an effect size received a rating of 5 on any item (from both reviewers), we did not continue the assessment because, as per our protocol, these effect sizes would not be included in any analysis. We discuss the risk of bias assessment, including the most common reasons for excluding an effect size, in Section 5.2. For studies with a lower than 5‐point rating, we used the ratings of the major items in sensitivity analyses.

A note is warranted for how we assessed some items in practice. Allocation concealment was assessed as a type of second‐order bias in RCTs. If there was doubt or uncertainty about how the random sequence was generated, this automatically carried over to the allocation concealment rating, which was also rated “Unclear”. Similarly, if the sequence generation rating was “High”, as for example in a QES, then the allocation concealment rating was also “High”. RCTs rated “Low” on sequence generation could get a “High” rating on allocation concealment if the sequence was not concealed from those involved in the enrolment and assignment of participants. However, if the randomisation was not done sequentially, this should not present a problem, and allocation concealment in non‐sequentially randomised RCTs were rated “Low”, given that the rating on sequence generation was also “Low”.

Blinding is in practice always a problem in the interventions we included. No included study was double‐blind for example, a standard that is very difficult to attain in an educational field trial. Furthermore, blinding was not extensively discussed in many studies, likely because it is difficult to attain in education interventions (Sullivan, 2011). For these reasons, we did not exclude any effect size due to insufficient blinding and rather than rating all studies that did not explicitly discuss blinding as “Unclear”, we sought to assess how likely it was that a particular group of participants was blind to treatment status. We used the following categories of participants: students in intervention and control groups, teachers, parents, and testers. We assessed the blinding item by the following standard: if all participant groups were likely to be aware of treatment status, we gave the study a rating of 4. If at least one group was likely blind to treatment status, it got a 3, and then we lowered the rating when more groups were blinded.

There were moreover very few studies that reported having an a priori protocol or analysis plan. We did not count hypotheses stated in the study as an a priori analysis plan. The plan should have been published before the analysis took place and we had to be able to find the plan.

This lack of prespecified outcome measures made it difficult to assess selective outcome reporting bias. However, a few studies lacked information regarding all outcomes described in, for example, the methods section of the study. To separate these effect sizes from the ones that did not contain information about a protocol or an analysis plan, we rated the latter ones with 1 (i.e., there was no evidence of selective outcome reporting). This rating should therefore not necessarily be considered as representing a low risk of bias.

#### 4.3.4 Measures of treatment effect

The analysis of effect sizes involved comparing an intervention to control or comparison conditions. We conducted separate analyses for short‐ and follow‐up outcomes. The below sections apply to both types of outcomes, unless otherwise mentioned.

##### Effect sizes using continuous data

For continuous data, we calculated standardised mean differences (SMDs) whenever sufficient information was available in the included studies. We used Hedges’ *g* to estimate SMDs, calculated as (Lipsey & Wilson, 2001, pp. 47–49):

(1) $$g=\left(1-\frac{3}{4N-9}\right)\times \left(\frac{{\bar{X}}_{1}-{\bar{X}}_{2}}{s_{p}}\right),$$

(2) $$SE_{g}=\sqrt{\frac{N}{n_{1}n_{2}}+\frac{g^{2}}{2N}},$$

where $N=n_{1}+n_{2}$ is the total sample size, $\overset{®}{X}$ the postintervention mean in each group, and $s_{p}$ the pooled standard deviation defined as

(3) $$s_{p}=\sqrt{\frac{\left(n_{1}-1\right)s_{1}^{2}+\left(n_{2}-1\right)s_{2}^{2}}{\left(n_{1}-1\right)+\left(n_{2}-1\right)}.}$$

Here, $s_{1}$ and $s_{2}$ denotes the raw standard deviation of the intervention and control group. We used covariate‐adjusted means, and the unadjusted posttest standard deviation whenever available. However, most studies did not report covariate‐adjusted means in a way that we could use. We then used the raw means instead (we test whether the studies reporting only raw means have different effect sizes in the sensitivity analysis). We decided to use the postintervention standard deviation, as more studies included this information than the preintervention standard deviation. In the few cases where the postintervention standard deviation was missing, we used the preintervention standard deviation.

All studies included in the data synthesis, except one, provided information so that we could calculate student‐level effect sizes. For the exception, we used information about intra‐cluster correlations (ICC) from Hedges and Hedberg (2007, table 6, p. 72, pre‐test covariate model for math in Grade 6, which is 0.098) to transform the teacher/class‐level effect size to a student‐level effect size.

Some studies reported an effect size where the mean difference was standardised using the control group's standard deviation (i.e., a Glass's *δ*) or reported effect sizes calculated with unclear methods (and no other information that we could use). Furthermore, a few studies used the school‐, district‐, or nation‐wide standard deviation to calculate a standardised mean effect size, but did not include information about the respective standard deviation for intervention and control group. We included these effect sizes, and tested the sensitivity to their inclusion in Section 5.4.

Our protocol stated that we would use intention‐to‐treat (ITT) estimates of the mean difference whenever possible. However, very few studies reported explicit ITTs, and the overwhelming majority only reported results for the students that actually received the intervention, rather than all for which the intervention was intended (often because they lacked outcome data for students that left the study). We therefore believe that the estimates are closer to treatment‐on‐the‐treated (TOT) effects and used TOT estimates when both ITTs and TOTs were available.

A few effect sizes are based on tests were low scores denote beneficial effects. We reverse coded these so that positive effect sizes imply beneficial effects of the intervention.

##### Effect sizes using discrete data

Only two studies exclusively reported discrete outcome measures. We transformed the outcomes into SMDs using the methods described in Sánchez‐Meca et al. (2003) and included them in the analyses together with studies reporting continuous outcomes.

#### 4.3.5 Unit of analysis issues

Errors in statistical analysis can occur when the unit of allocation differs from the unit of analysis. In cluster‐randomised trials, participants are randomised to intervention and control groups in clusters, as when participants are randomised by school. QES may also include clustered assignment of treatment. Effect sizes and standard errors from such studies may be biased if the unit‐of‐analysis is the individual and an appropriate cluster adjustment is not used (Higgins & Green, 2011).

Our protocol stated that we should adjust studies individually using the methods suggested by Hedges (2007). However, of the 61 studies with clustered assignment of treatment, less than a third contained any information about realised cluster sizes, and estimates of the ICC or the within‐cluster and between‐cluster variances (the ICC is the ratio between the between‐cluster and the total variance). Only a handful contained all the necessary information (both realised cluster sizes, and/or the ICC and the variances). We therefore adjusted all studies in a similar way and used an ICC of 0.09, which is very close to the mean of both reading and mathematics taken over Grades K‐6 in the pretest covariate models of tables 6 and 7 in Hedges and Hedberg (2007, pp. 72–73). In the sensitivity analysis, we report both results using unadjusted effect sizes and using a substantially higher ICC (0.3) than in the primary analysis.

##### Multiple intervention groups and multiple interventions per individual

Studies with multiple intervention groups with different individuals and studies using multiple tests for the same intervention groups were included in the review. To avoid problems with dependence between effect sizes, we used the robust‐variance estimation (RVE) methods developed by Hedges et al. (2010). We used the results in Tanner‐Smith and Tipton (2014) and Tipton (2015) to evaluate if there were enough studies for this method to consistently estimate the standard errors. That is, we report when the adjusted degrees of freedom are below (or close to) 4 in an analysis or for a moderator. See Section 4.3.9 for more details about the data synthesis. We treated multiple interventions over time as one combined intervention and coded the components.

##### Multiple studies using the same sample of data

We reviewed studies of interventions given to (partly) the same groups of students, but included one estimate of the effect from each sample of data in the meta‐analysis to avoid overlapping samples. We chose the estimate from the intervention that had lowest risk of bias, or contained the most information. See Supporting Information Appendix C: *Studies with overlapping samples or lacking information* for a summary description of included studies that we did not include in the meta‐analyses for this reason.

##### Multiple time points

As per our protocol we divided the analysis into:

- Short‐term effects (up to 3 months after the end of intervention).
- Medium‐ to long‐term effects (more than 3 months after the end of intervention).

In addition to the prespecified analyses, we also conducted analyses of effects measured 3.5 months to 1 year, 1–2 years, and more than 2 years after the end of intervention.

Some studies did not contain exact information about measurement timing. We interpreted these effect sizes as short‐term effects unless there was information in the study that indicated that the measurement was conducted more than 3 months after the end of an intervention. Similarly, for follow‐up measures, we coded the measurement as being within 1 year after the end of intervention when it was clear that the measurement was conducted more than three months after end of intervention but the specific timing was not reported.

If studies tested the same students two or more times within a period, then we used only the measurement closest in time to the end of intervention. That is, if a study tested students at 12 and 18 months after the end of the intervention, we only used the 12 months‐test in the analysis of effect sizes measured between 1 and 2 years after end of intervention.

As we found relatively few long‐term effects and the study variation was limited in ways we describe further below, the examination of heterogeneity and moderator analysis focused on the short‐term effects.

#### 4.3.6 Dealing with missing data

We assessed missing data and attrition rates in the individual studies using the risk of bias tool. Studies had to permit a calculation of a numeric effect size for the outcomes to be eligible for inclusion in the meta‐analysis. Where studies had missing summary data, such as missing standard deviations, we derived effect sizes where possible from, for example, *F* ratios, *t* values, *χ* ^2^ values and correlation coefficients using the methods suggested by Lipsey and Wilson (2001). If these statistics were also missing, we asked the study investigators if they could provide us with the information. We were unable to retrieve information from 24 studies. These studies were included in the review but excluded from the meta‐analysis (see Supporting Information Appendix C: *Studies with overlapping samples or lacking information* for a summary description).

Many studies did not provide data about all moderators. See Section 4.3.10 and Table 1 for information about missing moderator data. As the number of included studies limited the number of moderators we could include in the analysis, we focused on moderators that had no missing data, were relevant for all types of studies, and were not highly correlated with other moderators.

**Table 1:** Descriptive statistics: Study context, design, outcome assessment, participants, and intervention characteristics for intervention‐control studies included in the meta‐analysis

Study characteristics	*k*	*I*	*n*	*Mean*_*i*_	*SD*_*i*_	*Range*_*i*_
*Study context*						
% performed in the United States	195	327	1334	0.86	0.35	0–1
Participants	195	327	1334	280.34	1276.94	15–21,317
Districts	147	240	959	2.91	5.03	1–27
Schools	177	295	1213	12.18	13.92	1–147
*Study design and implementation*						
% QES	195	327	1334	0.07	0.26	0–1
% Implementation problems	177	285	1172	0.11	0.32	0–1
*Outcome assessment*						
% General test*	195	327	1334	0.09	0.28	0–1
% Follow‐up test*	195	327	1334	0.22	0.41	0–1
*Participant characteristics*						
% Girls	167	290	1254	44.58	10.03	0–65
Grade	195	327	1334	2.36	1.83	0–7
% Minority	154	271	1139	65.49	27.28	0–100
% Low income	120	192	802	69.48	21.11	12.1–100
*General intervention characteristics*						
% Mathematics tests*	195	327	1334	0.18	0.38	0–1
Duration in weeks	194	326	1329	23.11	18.01	1–160
Number of sessions	177	298	1280	69.08	62.14	3–400
Hours per week	178	299	1286	1.95	1.49	0.22–10
Implemented by school staff	192	320	1325	0.51	0.50	0–1
*Instructional methods*						
Coaching personnel	195	327	1334	0.11	0.31	0–1
Computer‐assisted instruction	195	327	1334	0.15	0.36	0–1
Incentives	195	327	1334	0.09	0.29	0–1
Medium‐group instruction	195	327	1334	0.06	0.25	0–1
Other method	195	327	1334	0.02	0.15	0–1
Peer‐assisted instruction	195	327	1334	0.15	0.36	0–1
Progress monitoring	195	327	1334	0.13	0.33	0–1
Small‐group instruction	195	327	1334	0.65	0.48	0–1
*Content domain*						
Comprehension	195	327	1334	0.41	0.49	0–1
Decoding	195	327	1334	0.51	0.50	0–1
Fluency	195	327	1334	0.30	0.46	0–1
Multiple reading	195	327	1334	0.55	0.50	0–1
Spelling and writing	195	327	1334	0.23	0.42	0–1
Vocabulary	195	327	1334	0.26	0.44	0–1
Algebra and pre‐algebra	195	327	1334	0.08	0.27	0‐1
Fractions	195	327	1334	0.03	0.17	0–1
Geometry	195	327	1334	0.07	0.25	0–1
Multiple math	195	327	1334	0.24	0.43	0–1
Number sense	195	327	1334	0.15	0.35	0–1
Operations	195	327	1334	0.15	0.36	0–1
Problem solving	195	327	1334	0.10	0.30	0–1
General academic skills	195	327	1334	0.04	0.19	0–1
Meta‐cognitive strategies	195	327	1334	0.13	0.33	0–1
Social‐emotional skills	195	327	1334	0.05	0.22	0–1
*Single component interventions*						
Coaching personnel	195	327	1334	0.01	0.10	0–1
Computer‐assisted instruction	195	327	1334	0.06	0.23	0–1
Incentives	195	327	1334	0.01	0.11	0–1
Medium‐group instruction	195	327	1334	0.04	0.19	0‐1
Peer‐assisted instruction	195	327	1334	0.07	0.26	0–1
Progress monitoring	195	327	1334	0.01	0.10	0–1
Small‐group instruction	195	327	1334	0.47	0.50	0–1
Comprehension	195	327	1334	0.02	0.13	0–1
Decoding	195	327	1334	0.10	0.31	0–1
Fluency	195	327	1334	0	0	0
Spelling and writing	195	327	1334	0	0	0
Vocabulary	195	327	1334	0.00	0.06	0–1
Algebra/pre‐algebra	195	327	1334	0	0	0‐1
Fractions	195	327	1334	0.00	0.06	0–1
Geometry	195	327	1334	0	0	0
Operations	195	327	1334	0.01	0.11	0–1
Number sense	195	327	1334	0.03	0.18	0–1
Problem solving	195	327	1334	0.00	0.06	0–1
General academic skills	195	327	1334	0.00	0.06	0–1
Meta‐cognitive strategies	195	327	1334	0	0	0
Social‐emotional skills	195	327	1334	0.01	0.08	0–1

*Note*: The number of studies that provided information about a variable is denoted *k*, the number of interventions *i*, and the number of effect sizes *n*. The mean, standard deviation of the mean, and the range is taken over interventions, except for variables marked with *. As these variables differ within interventions, we averaged on the effect size level.

Abbreviation: QES, quasi‐experimental studies.

In a sensitivity analysis, we used multiple imputation to assign values to moderators with relatively low levels of missing information. We defined “relatively low” as missing in less than 20% of interventions. We confined the sensitivity analyses further to moderators that were relevant to all intervention types (e.g., the number of sessions and hours per week is not relevant for incentive and progress monitoring interventions). We constructed imputed data sets using the 3.6.0 version of the *mice* package in R (first developed by Van Buuren & Groothuis‐Oudshoorn, 2011), which uses chained equations to predict missing values. We averaged our results over five imputed data sets using the methods described in Rubin (1996) and used the predictive mean matching method to assign missing values.

#### 4.3.7 Assessment of heterogeneity

Heterogeneity was assessed with *χ* ^2^ (*Q*) test, and the *I* ^2^, and *τ* ^2^ statistics (Higgins et al., 2003). In Supporting Information Appendix L, we also provide the prediction intervals for the main effects and subgroup analyses (this analysis was not included in our protocol).

#### 4.3.8 Assessment of reporting biases

Reporting bias refers to both publication bias and selective reporting of outcome data and results. Bias from selective reporting of outcome data and results is one of the main items in the risk of bias tool.

To examine possible publication bias, we used funnel plots, Egger's test (Egger et al., 1997), and tested whether studies published in scientific journals had different effect sizes compared with other studies. We used the R package *metafor* to conduct these tests, and the restricted maximum likelihood (REML) estimation procedure with the Knapp and Hartung adjustment of standard errors (Viechtbauer, 2010; this procedure was recommended by e.g., Langan et al., 2019).

The simulation results in Pustejovsky and Rodgers (2019), published after our protocol, indicated that the original Egger's test often reject the null hypothesis of no asymmetry at higher rates than the chosen level of statistical significance (i.e., the Type I errors are inflated). We therefore also conducted a version of the “Egger sandwich”‐test suggested by Rodgers and Pustejovsky (2020), which had good Type I properties in their analyses. For the Egger sandwich‐test, we used the same RVE procedure as in the primary analysis (see next section for more details) and simply added a measure of the precision of each effect size, equal to (*N*/*n*_1_ *n*_2_)^0.5^, to the respective estimating equation.

#### 4.3.9 Data synthesis

We conducted the overall data synthesis in this review when effect sizes were available. Effect sizes coded with a very high risk of bias (score of 5 on any item in our 5‐point scale) were not included in the data synthesis. The primary analysis had the following steps. We described summary and descriptive statistics of the intervention‐level characteristics, and the risk of bias assessment, and then performed analyses divided by measurement timing (end‐of‐intervention or follow‐up), which corresponded to our first objective for the review. We then explored heterogeneity across instructional methods and content domains (corresponding to the second objective), and other study characteristics (corresponding to the third objective). We describe these subgroup and moderator analyses in the next section.

We used the RVE procedure in the R command *robumeta* (Fisher et al., 2017) in all our analyses. The RVE procedure allowed us to simultaneously include all effect sizes from each study and avoid problems with dependence between effect sizes for estimation and to calculate robust standard errors (Hedges et al., 2010). We used the random‐effects model weighting scheme option, as it seemed most likely that the effects of the included interventions were not the same across studies, but follow a distribution. A fixed effects model would therefore be less appropriate in our case (e.g., Borenstein et al., 2009). As there were many more effect sizes from studies conducting several tests on the same samples than effect sizes from studies reporting results from independent samples, we chose the correlated effects model instead of the hierarchical effects model in *robumeta*.

The RVE procedure requires an initial estimate, $\hat{\rho }$, of the correlation between tests within the same study. We used $\hat{\rho }$ = 0.8 (as e.g., Dietrichson et al., 2017; Hedges et al., 2010; Wilson et al., 2011). We report 95% CIs throughout the analysis (i.e., “statistically significant” denote *p* < .05) and used the small sample adjusted standard errors and degrees of freedom suggested by Tipton (2015) to calculate CIs. We reported when the adjusted degrees of freedom were close to or below 4, as the results in Tanner‐Smith and Tipton (2014) and Tipton (2015) indicate that the standard errors are not reliable below this level.

Despite that the RVE procedure may have some disadvantages in terms of estimating heterogeneity parameters (see Tanner‐Smith et al., 2016), we chose to use the same framework for all analyses in order to make sure that disparate results were not caused by using different statistical models. We provide a sensitivity analysis for our main results in the “Publication bias” section and in Supporting Information Appendix J: *Forest plots by intervention component*, where we used study‐level effect sizes (a simple average of the effect sizes in each study) and the REML procedure in the R package *metafor* in the analysis (Viechtbauer, 2010).

#### 4.3.10 Subgroup analysis and investigation of heterogeneity

One of the main objectives of the review was to assess the comparative effectiveness of intervention components. We therefore performed subgroup and moderator analysis to attempt to identify the characteristics of interventions and study methods that were associated with effect sizes. We again used the RVE procedure in *robumeta* and reported 95% CIs for regression coefficients. We performed two types of analyses: *single‐factor subgroup analysis*, in which we estimated RVE models including only an intercept on samples of effect sizes defined by the subgroup of interest, and *multiple meta‐regression analyses*, in which we estimated RVE models including additional moderators besides the intercept. Although both types of analyses are regression‐based, we sometimes refer to the latter as just “meta‐regressions” to ease the reading. Below we describe the variables we used to define subgroups and as moderators, and thereafter a roadmap for the analysis, which includes a discussion of the advantages and disadvantages with the different types of analyses.

Most included moderators were coded as indicator variables (most variables are natural indicators, e.g., whether the study design was an RCT or not). Continuous variables were mean‐centred to facilitate interpretation. Our protocol specified the following types of moderators:

- Subject
- Study design
- Effect size measurement
- Participant characteristics
- Treatment modality
- Dosage
- Implementation quality

It is important to note that the number of included studies and the number of effect sizes were not large enough to include all coded moderators in one meta‐regression (see Supporting Information Appendix D: *Coding scheme* for a description of all coded variables). In line with the objectives of the review and our protocol, we therefore focused the analysis of subgroups and heterogeneity on instructional methods and content domains. These components are substantive features of interventions that for example teachers and principals can affect, in contrast to other moderators (e.g., participant characteristics may be more difficult to affect for a school). They were also more often (quasi‐)experimentally manipulated in studies than other moderators in our sample. They may therefore be less likely to be confounded with other, omitted moderators. However, we want to emphasise that the moderator and subgroup analysis estimate the associations between moderators and effect sizes, and may not capture the causal effects (Thompson & Higgins, 2002).

To further reduce the number of moderators, we first excluded moderators with very low variation (i.e., for which nearly all observations have the same value) or where information was missing from studies. We also excluded moderators that were not relevant for all intervention types (e.g., there is no number of sessions in an intervention that provide students with incentives to read a certain number of books).

We characterised the included interventions using two general categories of treatment modalities or intervention components: *instructional method* and *content domain*. As described in our protocol, the components were not fully prespecified, but developed and adapted during the coding process. We used previous reviews and author‐reported classifications in included studies as a starting point, and an iterative process to construct component categories. Below, we describe the coded components by treatment modality, and how we used these components to develop the moderators we included in the analyses. Note that interventions often contained more than one component and they were coded in all component categories they contained. The categories below are therefore not mutually exclusive.

We only coded that an intervention included a component when it was clear from the study that the component was used. For example, Torgesen et al. (2007) write that their intervention contained extensive “professional development and support” but do not mention whether teachers were coached or not. Therefore, we did not code the interventions in this study in the coaching of personnel‐category.

Some studies examined the effects of the same programme (e.g., READ180, PALS). In some cases, the same programme was described differently across the studies. As implementations may differ, we used the descriptions provided in the studies as much as possible and the same programme may thus be coded in different categories. In other cases, information about for example group sizes was lacking from a study and we then inferred a likely group size from information about the programme in other studies of the same programme.

##### Instructional method

The instructional method‐categories describe the method of delivering the intervention; that is, the contrast between how the intervention group and the control group were instructed. Many interventions contained more than one instructional method. In these cases, we have coded the intervention in all categories.

###### Coaching of personnel

Interventions in this category included programmes that provided teachers or other school personnel with coaches. Coaching of personnel hired by the research team, for example, ongoing training of college students acting as tutors, was not coded in this category. Furthermore, note that this component did not include professional development interventions that seek to develop more general teaching or management skills, as such interventions were never targeted to at‐risk students in our sample. The coaching in this category was mainly connected to the implementation of a specific reading or mathematics programme.

###### Computer‐assisted instruction (CAI)

This category indicated whether the intervention, or parts of the intervention, involved computers, tablets, or similar devices in the instruction of students. Computer assistance to teachers was not coded in this category. For example, in Fuchs et al. (1997) teachers get feedback from curriculum‐based measurement implemented on a computer. However, it is only the test that is taken on the computer, which is not used in the instruction of students. Consequently, we did not code this intervention in the CAI‐category (but in the progress monitoring‐category).

###### Incentives

Incentive programmes intended to increase the academic performance of students were included in this category. The incentives were not always monetary, non‐financial incentives were also included. Examples included interventions where the incentive component was the only component, for instance, students were paid to perform on interim assessments or to improve general achievement. Most interventions combined incentives with other components.

###### Peer‐assisted instruction

We separated between adult‐led instruction and peer‐assisted instruction. We defined peers as students in Grades K‐12. Interventions such as cross‐age tutoring where fourth graders tutored second graders were thus coded as peer‐assisted instruction (for both tutors and tutees if results were reported for both groups). If on the other hand college students acted as tutors to primary school students, the intervention was coded as adult‐led small‐group instruction (see below for description). We coded the exact group size, if available in the studies, but to keep the number of moderators down, we used a single moderator for the peer‐assisted instruction category in the main analysis. Most studies used small‐groups like pairs.

###### Progress monitoring

This category included interventions that added a specific progress monitoring component, where teachers received detailed information about the students’ development. Note that for example small‐group interventions of all kinds are also likely to contain increased feedback and in a sense increased (informal) progress monitoring. These interventions were not automatically coded in this category. Interventions had to add an extra component of progress monitoring, such as using curriculum‐based measurements (CBM) during the intervention, to be coded here. Few studies used progress monitoring as the only instructional method, it was almost always combined with other methods.

###### Medium‐ and small‐group instruction

As mentioned, we separated between adult‐led instruction and peer‐assisted instruction, and these two categories included adult‐led instruction. In some interventions, instruction was given in class, and not divided into smaller groups (this was, or was very likely to be, the same type of instruction given to the control group, so we did not create a moderator for this group size). We coded the exact group size whenever available but quite a few studies did not provide exact information about group size (but reported for example a range). We coded interventions without specific information about group size in the most likely category, given other information in the study or based the coding on information from other studies of the same intervention (e.g., there are several studies of READ180).

Because of the missing data, and to keep the number of moderators down, we created two moderators of adult‐led instruction in groups of at most five students (small‐group instruction) and groups of 6–20 students (medium‐group instruction). A smaller group usually meant that the information was included, and more exact, and we therefore coded interventions where it was clear that the instructional group were smaller than a whole class, but not clear whether it was small‐group or medium‐group instruction in the latter category. Some interventions vary the group size during the intervention. That is, they used both small‐group and medium‐group instruction. We then coded them in both categories. We used these two moderators in most of our analyses, but we also examined finer categories and used comparison designs that assigned group size (quasi)‐experimentally in some analyses.

Lastly, we created a category called “other method” that included interventions that were not coded in any of the above categories. There were two types of interventions making up this category: in a few cases, there was no difference in how the intervention and control group was instructed. Either only the content differed, or the intervention group was just provided extra instruction time. As many studies did not provide information about how much instruction time the intervention and the control group got in a certain area, the latter case was difficult to assess systematically for all interventions. Therefore, we did not create a separate category for extra instruction time.

##### Content domain

The content domain describes the area targeted by the intervention and the material taught. Interventions often follow the curriculum but put more focus on certain domains. The difference between the treatment and control group was therefore less sharp for content domains compared with instructional methods. We divided these components into reading, mathematics, and other areas. For reading, we used the following categories:

###### Comprehension

Reading comprehension interventions focused on the understanding and learning from text. Reading comprehension is described by the National Reading Panel (2000) as an active process where interaction between the text, the author, and the reader results in the understanding or meaning making of the text. The RAND Reading Group defines comprehension as “the process of simultaneously extracting and constructing meaning through interaction and involvement with written language” (RAND Reading Study Group, 2002, p. 720).

###### Decoding

Decoding interventions focused on the translation of print to speech. This category included for example word identification, word study, and word reading interventions. We also included interventions in this category that taught phonological awareness, phonemic awareness, and phonics. Such skills are often thought to be precursors to efficient decoding.

###### Fluency

We defined fluency as the ability to read orally with speed, accuracy, and proper expression (The National Reading Panel, 2000). Interventions in this category aimed for example to improve the ability to read in a “smooth and effortless” manner (Allinder et al., 2001).

###### Spelling & Writing

Some interventions included spelling and writing training, which, while not strictly a reading skill, we thought were related enough (and was also tested with standardised reading tests).

###### Vocabulary

This category included interventions focused on increasing the number of words a student knows. We also included the teaching of “sight words”—that is, frequently occurring words with spelling‐to‐sound irregularities (Castles et al., 2018)—in this category.

In addition to these single domains, we also coded a multiple reading domain category. Besides interventions focused on more than two of the above subdomains, this category included interventions that focused on reading in general but did not explicitly mention any subdomains. We interpreted these interventions as implicitly targeting more than one reading domain.

For math interventions, we found interventions targeting the following categories^6^:

###### Algebra/pre‐algebra

Algebra and pre‐algebra interventions focused on, for example, the basics of equations, and graphs.

###### Fractions

Fraction interventions taught the concept of fractions and how to manipulate them.

###### Geometry

Geometry refers to the study of, for example, shapes, sizes, and positions.

###### Number sense

Number sense, or number knowledge, interventions targeted basic skills such as counting, number recognition, number relations (bigger and smaller, before and after), and number set operations (e.g., Dyson et al., 2015).

###### Operations

This category included for example training in addition, subtraction, multiplication, and more generally, computational skills.

###### Problem solving

Interventions coded in this category trained students in solving word problems. For example by teaching students the structural features underlying different problem types (e.g., Fien et al., 2016).

As for reading, we coded a multiple mathematics domains category, which included interventions explicitly covering more than one domain as well as more general math interventions.

Finally, we coded three categories to characterise interventions targeting other areas instead of, or together with (subdomains of) reading and mathematics.

###### Meta‐cognitive strategies

Meta‐cognitive strategies and self‐regulation interventions aimed to help students think about their own learning more explicitly, and develop strategies for such learning, including managing one's own motivation towards and engagement with learning. The intention was often to give pupils a repertoire of strategies to choose from during learning activities, including study skills and learning how to learn. In comparison to the next domain, social‐emotional skills, the skills trained in this category were more focused on the individual student, and less on the relations to other students or school staff.

###### Social‐emotional skills

Interventions in this category focused on improving academic achievement through e.g., improving social skills, and mitigating problematic behaviour. They thus had a more relational focus compared with meta‐cognitive interventions.

###### General academic skills

This category included studies without a particular content domain or a more general academic focus than just reading and math. As the authors studying such interventions still included a standardised test in reading or math, we interpreted the authors as expecting the intervention to improve achievement in these subjects.

##### Other intervention characteristics

When coding other intervention characteristics, we used information about the intervention group, if available. If information was not available on the intervention group level, we used joint intervention and control group information, and then higher levels, such as Grades and schools. We treated information on levels higher than schools, such as school districts, as missing. Some studies included only information about intervention characteristics given in a range. In these cases, we used the midpoint of that range. Below, we first describe the moderators used in the analysis in more detail and then describe moderators that we coded, but for different reasons did not include in the analysis.

###### Study context

We coded the country where the information was performed. When information was missing, we made an assessment based on e.g., where the authors were based at the time of the study, and on the mentioning of country‐specific reforms like No Child Left Behind in the United States. We reported the number of participants, schools and districts involved in the study.

###### Study design characteristics

We coded a moderator indicating whether the study was a QES. We found only one QRCT and coded this study in the QES category. That is, the reference category is RCTs. We coded whether implementation was monitored by the researchers in some way, whether problems were mentioned, and if so, what type of problems that was mentioned. Some problems mentioned by more than one study were low attendance, that implementers had low quality of implementation or low motivation, and that some in the control group might have received (some of) the intervention. In a sensitivity analysis, we used an indicator equal to one if implementation problems were explicitly mentioned.

###### Effect size measurement

We calculated effect sizes on the basis of different types of tests, which may cause heterogeneity. We therefore coded the content domains of the tests. In the meta‐regressions, we included one moderator indicating whether a test was general, in the sense that it covered two or more subdomains. We furthermore coded four moderators that relate to the calculation of effect sizes, which we used for sensitivity analyses. The first indicated whether we had to use the raw means to calculate the effect size. Glass's *δ* indicated whether the SMD was standardised with the control group's standard deviation. This was the case in some studies that did not include information about the pooled standard deviation, and rather than excluding them, we tested whether our results are sensitive to their inclusion. Another moderator indicated effect sizes where it was unclear exactly how the effect size was calculated (we believed that it was either Hedges’ *g*, Cohen's *d*, or Glass's *δ*). We also used an indicator equal to one if the SMD had been standardised with a standard deviation from a super‐population (e.g., Grade, district, or state) instead of the intervention and control group, or if the number of included schools, districts or regions was larger than the intervention median.

Both standardisation with a super‐population and including more schools, districts, and regions may imply that the variance in the sample could be larger and effect sizes mechanically smaller, as the study included a possibly more varied group than other studies (see e.g., Lipsey et al., 2012). As there were few studies that standardised with a super‐population, we chose to make one variable for these two related problems.

###### Participant and sample characteristics

We measured the gender distribution by the share of girls. We coded both age and Grade (minimum, maximum, and mean for both) but the information about age, as well as minimum and maximum for both variables, were missing for far more interventions. We therefore focused on the mean Grade, and used the information about the mean age and the school system in the few studies missing Grade information to estimate a mean Grade. If we only had information about a range, we used the midpoint of that range. That is, if a study reported students in kindergarten to Grade 4, we used Grade 2 as the mean. Outcomes were normally measured in the same Grade that the intervention was performed, but in some cases interventions spanned one or more Grades. The Grade variable we used refers to the Grade in which (most of) the intervention was implemented. To standardise the start of primary school across countries, we used the United States as a starting point and coded kindergarten as Grade 0. That is, if the average grade in a study is 0.5, then half the treatment group were kindergartners and half first‐graders. We recoded Year or Grade 1 to Grade 0 for studies conducted in countries like Australia, New Zealand, and the United Kingdom, where Year/Grade 1 denotes the first year of primary school.

In addition to the mean Grade, we coded the share of minority students (defined as not being part of the largest population group in a country) and the share of students from low‐income families, which was almost always measured as the share of students with free‐ or reduced price meals. As the criteria for getting free‐ or reduced price meals differ between countries, the latter variable was difficult to define consistently across countries.

###### Dosage

We coded three variables related to the dosage of an intervention. We measured duration in weeks. We used 40 weeks for a school year, and consequently 20 weeks for a semester. We measured the frequency of an intervention by the total number of sessions, and the intensity by the total number of intervention hours per week. For these dosage‐variables we coded both intended and received dosage. However, many studies either lacked information about the intended or received dosage. We used received dosage as a starting point, and added intended in the cases were the received number was missing.

###### Implementers

We used information about who implemented the intervention to develop an indicator for interventions implemented by school staff (e.g., teachers, special education teachers, coaches, teaching assistants, and teacher aides) with other types of implementers. Peer‐assisted instruction interventions, where peers deliver the instruction, where indicated in this category if school staff facilitated the instruction. The reference category included other type of instructors and implementers such as researchers, college students, or adult volunteers.

Our protocol mentioned and we coded information from several moderators, which we in the end could not use. As we believed they were unlikely to be representative of the literature, we did not report descriptive statistics about these moderators. The main reason for not using them was lack of information: For example, only 3 studies included in the meta‐analysis provided information about parental occupation and 14 about parental education. The share of students speaking the majority language as a second language (e.g., English language learners) was only included in 59 studies.

Two cases where we lack information are, we believe, especially important to highlight: First, the instruction given to the control group differed between interventions. Control group instruction was nearly always some form of TAU, but it was difficult to separate different TAUs from each other as the information was not detailed enough. We were therefore unable to create moderators measuring the quality of the control group instruction. We describe a way to test the sensitivity to differences in control group instruction further below. Second, we coded information about the target group of the intervention, but it was difficult to use this information to develop a moderator measuring the severity of academic difficulties. One reason was that many studies did not provide sufficient detail about how they assessed difficulties or at‐risk status. A few studies specifically targeted students diagnosed with learning disabilities, which is an option for a moderator measuring academic difficulties. However, other studies also included some learning disabled students and many more did not include information about the share of learning disabled students. Due to the small number of studies and the unclear contrast, we refrained from using learning disability as a moderator.

For other moderators, there was very little variation: almost all studies were conducted only in schools, and few had target groups that were not defined in terms of having academic difficulties. That is, few studies defined the target group purely in terms of for instance the students’ SES. We coded whether implementers received training before the intervention, but if this was not the case, it almost always meant that it was a researcher or someone affiliated with the research team who performed the intervention. The information is therefore overlapping with the variables measuring who implemented the intervention. We coded whether the control group was a waitlist design, but it was often not explicitly mentioned whether the control group got the intervention after the intervention group.

##### Roadmap for the subgroup and moderator analysis

Our investigation of the heterogeneity of the short‐term effects have five main parts:

1. We first tested whether effect sizes based on math tests were larger than effect sizes based on reading tests in single‐factor subgroup analyses defined by the type of test. We then categorised interventions by subgroups defined by the instructional methods and the content domains defined above, and again conducted single‐factor subgroup analyses on the sample of effect sizes containing the method or domain in question. As many interventions have more than one component (e.g., include more than one instructional method, or target more than one content domain), the effect sizes in this analysis should be interpreted as the weighted average effect size for interventions that *included* a certain component, not the effect size of that component in isolation. If an intervention included instructional methods or content domains, it consequently contributed effect sizes to more than one method/domain category. The advantages of this analysis are that more studies can be included in each subgroup and that the effect sizes are comparable with those estimated in almost all earlier reviews. There are also a few important drawbacks. As interventions often combined two or more methods and domains, we cannot separately identify the association of any method and domain. We may also confound methods with domains as well as both methods and domains with other study characteristics.
2. In a second analysis, we examined interventions that included only one instructional method or one content domain (called single method and single domain interventions henceforth). We again used single‐factor subgroup analysis (i.e., intercept‐only RVE models) on the sample of effect sizes from these interventions. This estimation strategy thus isolates the association between methods/domains and effect sizes better than the previous. The main drawback is that few components were examined in enough single method/single domain studies for this strategy to give reliable results. Furthermore, we may again confound methods and domains with each other, and with other study characteristics.
3. In a third set of analyses, we ran multiple meta‐regressions on the full sample of short‐term effect sizes, in which we included indicators for each instructional method and content domain, as well as all study characteristics without missing observations (indicators for QES, math tests, general tests, and the mean Grade). These meta‐regressions provide an estimate of the isolated association between each component and effect sizes, conditional on other components and study characteristics. However, it is difficult to rule out that the inclusion of moderators do not introduce bias instead of reducing it.^7^ Furthermore, we were unable to include interactions between components in these regressions. The reason is that there were few recurring combinations in our sample. Lastly, this specification relies on the linear model being a reasonable approximation to the true relation between effect sizes and moderators.
4. In the fourth subgroup analysis, we examined the effect sizes of the few recurring combinations in single‐factor subgroup analyses. This analysis suffers from similar limitations as subgroup analysis 1 and 2.
5. Lastly, we examined some of the intervention components with stable associations with effect sizes further using both single‐factor subgroup analyses and multiple meta‐regressions. In one such analysis, we included comparison designs that contrasted adult‐led one‐to‐one instruction with adult‐led instruction in slightly larger groups. As these comparison designs contained experimental manipulation of the group size, and no other variables, they get closer to capturing the causal effect of group size than moderator analyses of intervention‐control designs with different group sizes. Comparison designs were not included in any other analysis.

#### 4.3.11 Sensitivity analysis

We performed the following sensitivity analyses.

##### Effect size measurement

We tested sensitivity to measurement of effect sizes in the following ways: We included four moderators that indicated whether we used the raw means to calculate the effect size, standardised the SMDs with the control group standard deviation (i.e., Glass's *δ*), the standardisation was unclear, or the effect size was standardised with a standard deviation from a super‐population.

##### Outliers

We examined the distributions of effect sizes for the presence of outliers and the sensitivity of our main results by methods suggested by Lipsey and Wilson (2001): trimming the distribution by dropping the outliers and by winsorizing the outliers to the nearest non‐outlier value.

##### Clustered assignment of treatment

We tested sensitivity to clustered assignment of treatment by the methods described in Section 4.3.5.

##### Moderators with missing values

We used multiple imputation to account for missing values in some moderators with relatively low rate of missing values, as described in Section 4.3.6.

##### Control group progression

We coded a description about the control group condition, but it proved difficult to develop an informative moderator based on this coding (due to missing or insufficient information in a large share of studies). To gauge whether differences in the quality of instruction given to the control group may explain some of the effect size heterogeneity, we calculated the control group progression from pre‐ to posttest and divided by the control group posttest standard deviation in studies that included this information. We then (a) tested whether this control group “effect size” was heterogeneous across studies, and (b) used a meta‐regression to examine whether intervention components explained some of the heterogeneity. If there is heterogeneity and some of this heterogeneity is systematic across components, then we risk confounding the intervention components with the quality of control group instruction. However, not all studies included the necessary information. More progression may also mean that it is easier to improve the achievement of the students in that sample (intervention and control group) than in others, which would not necessarily bias our estimates. This test should therefore be interpreted with caution.

##### Risk of bias

We used the items with numerical ratings from the risk‐of‐bias assessment to examine if methodological quality was associated with effect sizes. The items with non‐numerical ratings are not relevant for all types of studies, and there was also low variation in their ratings. Because the items are categorical variables, we recoded them to indicator variables. For blinding, incomplete outcome reporting, and other bias, we contrasted effect sizes given a rating of 4 or unclear to those given lower ratings than 4. For the selective outcome reporting item, we contrasted those rated 1 (a large majority) with those given higher ratings or an unclear rating. The confounding item is only relevant for QES and captures features that are included in the other bias item for RCTs. It was therefore difficult to compare QES and RCTs in the same analysis. As we included few QES in the meta‐analysis (17) and our ratings of the confounding item exhibited relatively low variation (about 70% of the effect sizes received a rating of 4), we omitted the QES from this sensitivity analysis.

##### Publication bias

Lastly, we examined publication bias by the methods described in Section 4.3.8.

### 4.4 Deviations from protocol

The search strategy in our protocol listed “Education Research Complete” among the databases. However, at the time of the search, we no longer had institutional access to this database and did not include it either in our original search or in the updated search. Furthermore, when we wrote protocol, we did not have access to the Teacher Reference Center database. During the search process, we did have access, so we chose to search and include the database in the review.

Due to lack of institutional access, we did not search British Education Index, FRANCIS, Dissertation & theses A&I, CBCA Education, and Australian Education Index in the updated search.

According to our protocol, we were supposed to contact international experts to identify studies and give them the list of included studies. We thought it would be advantageous to involve experts earlier in the process and asked them about relevant studies before our screening process was completed. Therefore, they did not receive a list of included studies. As mentioned earlier, we could not search the Institute for Education Sciences’ Registry of Randomized Controlled Trials, as the webpage was shut down at the time of both the original and updated search.

Our protocol stipulated that we would use ITT estimates whenever available. Very few studies reported ITT estimates, however. The estimates reported were in our view closer to TOT estimates and we therefore decided to use the TOT estimates also in studies that reported both ITT and TOT estimates.

Our protocol did not mention the calculation of prediction intervals. As prediction intervals provide a measure of the dispersion of effect sizes (i.e., how effect sizes vary across populations; Borenstein et al., 2017), we reported the prediction intervals for the main effects and subgroup analysis as a complement to the other heterogeneity statistics (see Supporting Information Appendix L).

After the publication of our protocol, Pustejovsky and Rodgers (2019) showed that that the original Egger's test often reject the null hypothesis of no asymmetry in funnel plots at higher rates than the chosen level of statistical significance. To check weather our results were sensitive to this problem, we also conducted a version of the “Egger sandwich”‐test suggested by Rodgers and Pustejovsky (2020).

Lastly, we conducted the analyses in R instead of Stata due to better availability of packages for meta‐analysis.

## 5 RESULTS

### 5.1 Description of studies

#### 5.1.1 Results of the search

**Figure 1:**
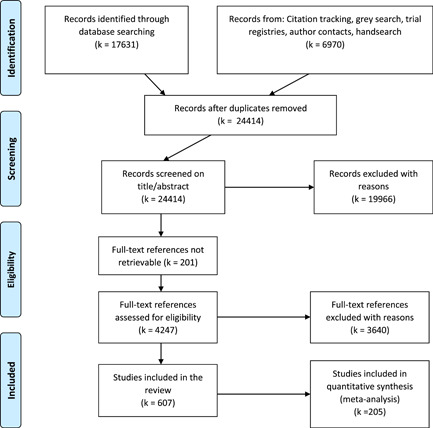
Flowchart of the search and screening process

Figure 1 displays the results of the search process. The total number of potentially relevant records was 24,414 after excluding 187 duplicates (database search: 17,444; grey literature: 3014; citation tracking: 1509; author contacts: 576; hand search: 1024; trial registries and others: 847).

We screened all records based on title and abstract. Of the ones that we did not exclude, 201 records were not retrievable in full text. Older reports and dissertations were overrepresented among the records we could not retrieve. We screened the remaining 4247 retrievable records in full text. A large number of studies were not relevant for this review due to the Grade of the participating students. Studies that were relevant except for the grade of participating students were included in a review covering Grades 7–12 (Dietrichson et al., 2020). In total, 607 studies met the inclusion criteria for this review and we extracted data from these studies.

#### 5.1.2 Included studies

We did not include all 607 studies in the meta‐analyses. We were unable to retrieve sufficient information from 24 studies/study authors to calculate an effect size. We excluded 17 studies, which used samples that overlapped with other included studies and had either higher risk of bias or contained less information. 104 studies were not included in the meta‐analyses due to their study design. These studies used comparison designs that contrasted two alternative interventions and the contrast was not recurring in enough studies for meta‐analysis to be meaningful (one study included both an intervention‐control contrast and a comparison design and we counted it among the studies included in the meta‐analysis). We did not include 257 studies in the meta‐analysis due to the risk of bias assessment. All eligible outcomes in these studies had, in our view, too high risk of bias (for more details, see further below).

Furthermore, some of the remaining 205 (202 intervention‐control and 3 comparison designs) studies contained overlapping samples, but included for example information about short‐term and follow‐up outcomes. These were all included in some meta‐analyses, but never in the same. We treated studies that reported short‐ and follow‐up outcomes from the same intervention in separate papers as one study in the analysis (i.e., when clustering the standard errors). This definition left us with 195 included clusters of studies in the analysis of intervention‐control studies (none of the comparison design studies had this problem).

We discuss the results of the risk of bias assessment further in Section 5.2. See also the Risk of bias tables in Supporting Information Appendix F for details of the assessment for effect sizes that we included in the meta‐analysis, as well as those that we deemed had too high risk of bias (Supporting Information Appendix G). We describe the comparison designs in the Supporting Information Appendix E: *Description of comparison designs*. For more information about studies with overlapping samples or that lacked sufficient information for the calculation of an effect size, see the Supporting Information Appendix C: *Studies with overlapping samples or lacking information*.

The data we coded for the 195 (clusters of) intervention‐control studies and the comparison design studies included in the meta‐analyses are included in Supporting Information Appendices H and I. Below we describe the characteristics of the 195 intervention‐control studies, which we based most of the analyses on.

**Figure 2:**
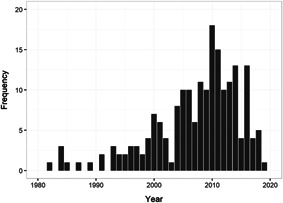
Number of intervention‐control studies included in the meta‐analysis by publication year

**Figure 3:**
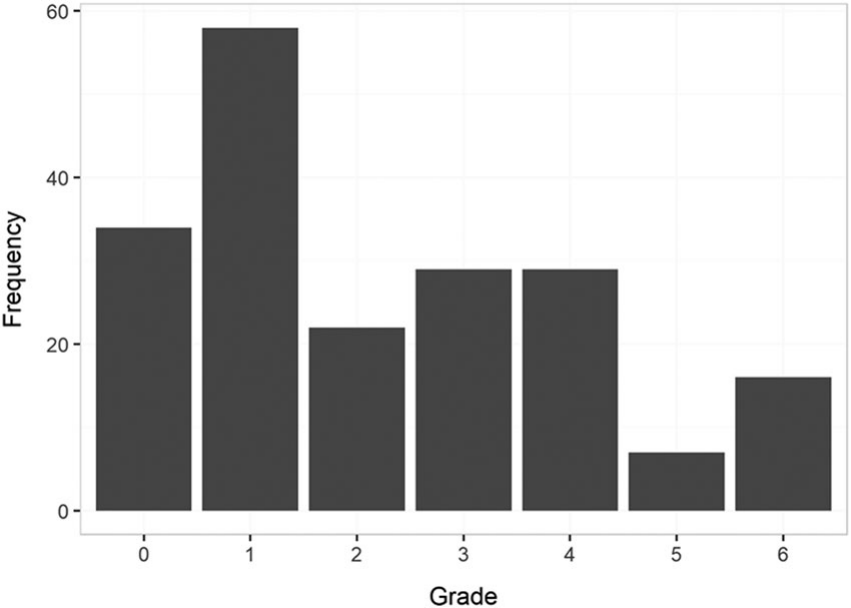
Number of intervention‐control studies included in the meta‐analysis by mean grade

Figure 2 displays the included studies by publication year. Most studies were published in the last 10–15 years, 28 included studies were published before the year 2000 and only 7 before 1990. It is therefore unlikely that we missed many relevant studies by restricting our searches to studies published after 1980. Figure 3 shows the 195 studies by the mean Grade of participating students during the implementation of the intervention. There were more studies with participants in kindergarten and, in particular, first Grade than in the higher Grades. The mean Grade was 2.4.

Table 1 contains descriptive statistics of the intervention‐control studies included in the meta‐analysis. Many studies contained more than one intervention, the effects of which may have been tested with more than one standardised test from which we calculated the effect sizes. We denoted the number of studies that provided information about a certain characteristic with *k*, the number of interventions with *i*, and the number of effect sizes with *n* (note that these are not necessarily unique study populations, as some studies with more than one intervention group used only one control group). As most characteristics vary on the intervention level, we averaged – with three exceptions marked with *—over interventions to calculate the mean, standard deviation, and range. For example, in a study with two interventions where each intervention and control group take two tests, we averaged by intervention over the two tests. These averages are the basis for means, standard deviations, and ranges in Table 1 (which is why there is an *i* subscript in the table). There were in total 195 studies, 327 interventions, and 1334 effect sizes.

Included interventions were to a large extent conducted in the United States (86%). The remaining interventions were from Australia (0.6%), Canada (0.9%), Denmark (0.3%), Germany (1.2%), Ireland (0.3%), Israel (0.3%), Netherlands (2.1%), New Zealand (2.1%), Sweden (3.7%) and the United Kingdom (2.1%). The mean number of participants, schools, and districts were 280, 12, and 3, respectively, but sample sizes varied quite widely and many studies were small. Most included study designs were RCTs, only 7% of interventions were QES. A small share, 11%, reported having some form of implementation problems, and a large majority of the tests (91%) tested a single reading or mathematics domain, that is, they were not general in our terminology. In 22% of the interventions, follow‐up tests were conducted (i.e., students were tested more than 3 months after the end of intervention).

Participants were more likely to be boys (45% were girls), and a majority were minority (65%) and low‐income students (70%). Information about minority and, in particular, low‐income students was relatively often missing. Note that we often based the share of low‐income students on the share receiving free‐ and reduced price lunches in studies from the United States, which is not necessarily directly comparable to low‐income variables used in other countries (e.g., free school meals in the United Kingdom).

The effects of interventions were more often examined using reading tests, 18% used a mathematics test. Note that the separation of effect sizes was made based on the test, not the subject targeted, as there were several interventions that used tests in both reading and mathematics (*i* = 19), or used a composite reading and mathematics tests (*i* = 2). This is also a main reason why we do not separate results into reading and mathematics interventions to start with (we will return to this issue in the analysis of heterogeneity).

The mean duration was about 23 weeks, and the mean frequency and intensity equalled 69 sessions and 2 h per week. The range was wide for all three variables measuring intervention dosage and note that we lack information for quite a few interventions regarding frequency and intensity (for 28 and 27 interventions, respectively). Among half of the interventions were implemented by school staff.

Among the instructional methods, small‐group instruction is clearly the most studied method. Around 65% of all interventions included small‐group instruction. The shares otherwise ranged from 2% in the other method‐category to 15% in the CAI and peer‐assisted instruction category.

The proportions of interventions targeting reading domains were more similar, from fluency being targeted in 30% of all interventions to decoding that was targeted by 51% of all interventions. Most interventions (55%) targeted multiple reading areas. Fewer interventions targeted mathematics and the shares ranged from 3% for fractions to 15% for number sense and operations. Targeting more than one domain was common also for math interventions, 24% of interventions targeted more than one math domain. Regarding the non‐math and non‐reading domains, meta‐cognitive strategies was taught by 13%, social‐emotional skills by 5%, and general academic skills by 4%.

The fact that combinations of both instructional methods and content domains was relatively common imply that there are few single component interventions. As shown in the lower part of the table, it is only small‐group instruction that have been studied alone in a large number of studies. Small‐group instruction was the only instructional method in 50% of all interventions. Among the content domains, single domain interventions were rare.

#### 5.1.3 Excluded studies

Due to the large number of studies screened in full text, we were unable to describe all excluded studies. To exemplify how we applied the inclusion criteria, this section describes studies that met all but one of our criteria.

The included study designs contrasted intervention and control groups, or alternative interventions, to estimate effects. Compton et al. (2012) examined 129 first Grade children who were unresponsive to classroom reading instruction and were randomly assigned to 14 weeks of small‐group intervention. They defined nonresponders and responders using a standardised test of word identification, and then used logistic regression models to examine which information predicted the group students ended up in. As the study did not compare an intervention group to a control group, we excluded it.

We included interventions that sought to improve academic achievement or specific academic skills. We however excluded interventions that used accommodations to improve results on tests or only sought to improve test‐taking skills, like Scruggs and Mastropieri (1986). We also excluded interventions that primarily aimed to improve student attendance, like the intervention examined by Guryan et al. (2017). Although improving attendance may also improve student achievement, it need not do so on standardised tests. Furthermore, in groups of low attenders, improving attendance in the intervention group compared with the control group changes the composition of the students who take standardised tests, making it difficult to evaluate whether academic skills have improved (e.g., because the programme may affect student achievement through other channels than just attendance, an instrumental variables analysis need not work, even if the programme is randomly assigned).

Interventions had to be targeting students with or at risk of academic difficulties to be included. Al Otaiba et al. (2011) examined an intervention where teachers received help to differentiate reading instruction in kindergarten based upon students’ ongoing assessments of language and literacy skills. Although the intervention included students’ from at‐risk groups (e.g., students who qualified for special education) and some schools were Title I and Reading First schools (indicating that a relatively large share of the students were from disadvantaged groups), we excluded the study. The programme did not specifically target students with or at risk of academic difficulties, but all students in the participating schools, including high‐performing students.

Interventions should be school‐based to be included, meaning that they were conducted in school during regular school‐hours and semesters. Kim (2006) studied a voluntary summer reading intervention. Although some reading lessons took place in during the spring semester, most of the intervention period was outside the regular school year. We therefore excluded the study.

Studies had to test the effects of interventions using standardised tests in reading and mathematics. Fortner (1986) tested intervention effects with the Test of Written Language (TOWL). TOWL is standardised but primarily a test of writing. However, it includes a subtest of vocabulary but the students’ vocabularies are assessed based on their writing products. Therefore, we thought this subtest was more of a writing than a reading assessment, and excluded the study.

### 5.2 Risk of bias in included studies

We excluded 257 studies that met our inclusion criteria from the meta‐analyses because we rated all relevant effect sizes as having too high risk of bias. That is, our assessment was that all effect sizes in the 257 studies were more likely to mislead than inform the analysis. Studies in which some effect sizes but not all were rated too high risk of bias are counted among the 205 studies included in the meta‐analysis.

It is important to note that we assessed the risk of bias of the effect sizes that met our inclusion criteria and, in turn, the effect estimates we could use to calculate effect sizes, not all effect estimates in a study. That is, there may well be estimates with low risk of bias in the studies that we excluded from the meta‐analysis because they did not fit our inclusion criteria, or because they were reported in a way that we could not use them. For example, to calculate effect sizes from certain model‐based estimates require information about correlations between the variables in the model (Lipsey & Wilson, 2001). Such correlations were rarely included in the studies. Then we could only use the raw means to calculate effect sizes, which, at least in QES, often have too high risk of bias. Furthermore, contrasts between, for example, at‐risk and not‐at‐risk students or at‐risk students and national norms, are often informative but not comparable to effects estimated with a control group of at‐risk students. Thus, a too high risk of bias rating is not a synonym for a low‐quality study.

Included studies for which we rated all effect sizes as having too high risk of bias were disproportionally dissertations (33% compared with 7% among the intervention‐control studies included in the meta‐analysis), older studies (mean publication year is 2004 compared with 2007), and QES (84% of the excluded studies were QES). The most common reasons for giving a too high risk of bias rating were:

1. Inadequate adjustment for confounding factors (114 studies). Examples include QES that did not report whether intervention and control groups were balanced on relevant confounders, that did not adjust for pretests, or that had very large preintervention imbalances on important confounders. Large preintervention imbalances on important confounders was rarely the only reason for a too high risk of bias rating (and was never the only reason for RCTs). Instead, we gave too high risk of bias ratings when there were large imbalances and the assignment procedure seemed unlikely to produce comparable treatment and control groups.
2. Confounding of intervention effects with for example school, teacher, class, or cohort effects (70 studies). For example, if studies assign the intervention on the school level and there is only one intervention and one control school, then the risk of confounding the intervention effects with school effects is very high. Our lower limit was two assigned units each in the intervention and the control group, which may still imply a relatively high risk of bias.
3. Non‐comparable intervention and control groups (38 studies). Examples include studies that compared at‐risk students with not‐at‐risk students or voluntary participants with students who declined participation.

We excluded 40 RCTs. RCTs were for example excluded because randomisation was compromised and there was inadequate control for confounding (17 studies), because only one unit was assigned to the intervention or control group (10 studies), because the studies reported results for a subset of included tests or students (6 studies), or because of large‐scale attrition (4 studies). We excluded the 3 remaining studies for more idiosyncratic reasons. We listed the reasons for giving a rating of too high risk of bias by study (for both RCTs and QES) in Supporting Information Appendix G: *Studies with a too high risk of bias rating*. We reported the main reason per study, but note that there were cases with more than one reason for too high risk of bias ratings.

#### 5.2.1 Risk of bias of effect sizes included in the meta‐analysis

**Figure 4:**
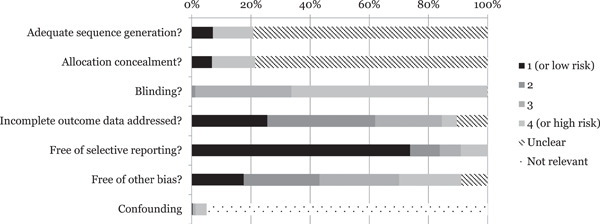
Summary of risk of bias items for effect sizes included in the meta‐analysis

Figure 4 shows the distribution of the assessments for intervention‐control effect sizes included in the meta‐analysis by the items in the risk of bias tool. See Supporting Information Appendix F: Risk of bias in studies included in the meta‐analysis for a description of the ratings by study and item.

Few RCTs reported how they generated the random sequence used to assign students to intervention and control groups, only 7% of effect sizes were given a low‐risk assessment (QES have high risk by default on this item and therefore also on allocation concealment). More generally, the procedure of randomisation was often not described in detail. In almost all cases where the random sequence generation was described and was adequate, the allocation was likely concealed, as the randomisation was not done sequentially.

All studies had problems with the blinding of treatment status. No effect size received a rating of 1 on this item and very few studies provided an explicit discussion about blinding. There was some variation between effect sizes though: For around 66% of effect sizes, there was no indication that any participant group was blind to treatment status. About 32% of effect sizes had one group that was blind to treatment status (usually the persons performing the tests), and in 2%, several groups were likely blinded. The ratings for this item vary to some extent also within studies, as some studies, for example, used both tests performed by persons outside the study (e.g., state‐wide tests) and by involved, non‐blinded, study personnel.

The distribution of assessments for incomplete outcome reporting was more mixed. Only 5% had a high risk of bias rating on this item and almost all studies provided information. We rated a large majority (74%) of effect sizes to be free of selective reporting, but this does not mean that the studies followed a prespecified protocol and analysis plan. The figure omits the items examining if the study followed an a priori protocol and analysis plan, as just two studies mention a protocol and three an analysis plan explicitly written before conducting the analysis. Torgerson et al. (2011) was the only study for which we could retrieve both a protocol and an analysis plan. Lastly, about 17% of effect sizes were rated as having a high risk of bias for the other bias item.

The confounding item was only assessed for the 19 QES; 75% of effect sizes from these studies received a rating of 4. That is, a high risk of bias. Only 7% of effect sizes from QES received a rating of 1 or 2.

In sum, the included effect sizes and studies have relatively often a high risk of bias, but there is also variation. We return to the sensitivity of our results to different part of the risk of bias assessment in Section 5.4.

### 5.3 Effects of interventions

#### 5.3.1 Overall short‐term and medium‐ to long‐term effects

This section presents the results from the robust‐variance estimation of the overall short‐term effects—that is, from the end of intervention to 3 months after—and effects with a longer follow‐up period.

We included 1030 effect sizes, 189 clusters, and 206,186 student observations^8^ in the analysis of short‐term effects. Eleven individual studies did not provide results from a short‐term test. The weighted average short‐term effect size was positive and statistically significant (ES = 0.30, CI = [0.25, 0.34]). This effect size corresponds to a 58.4% chance that a randomly selected score of a student who received the intervention is greater than the score of a randomly selected student who did not (a null effect would imply a 50% chance, see e.g., Ruscio, 2008, for a conversion formula). The *Q*‐statistic was 797.3 (*p* < .01), the *τ* ^2^ 0.067 and the *I* ^2^ was 76.4. All three heterogeneity measures therefore indicated substantial heterogeneity.^9^

**Figure 5:**
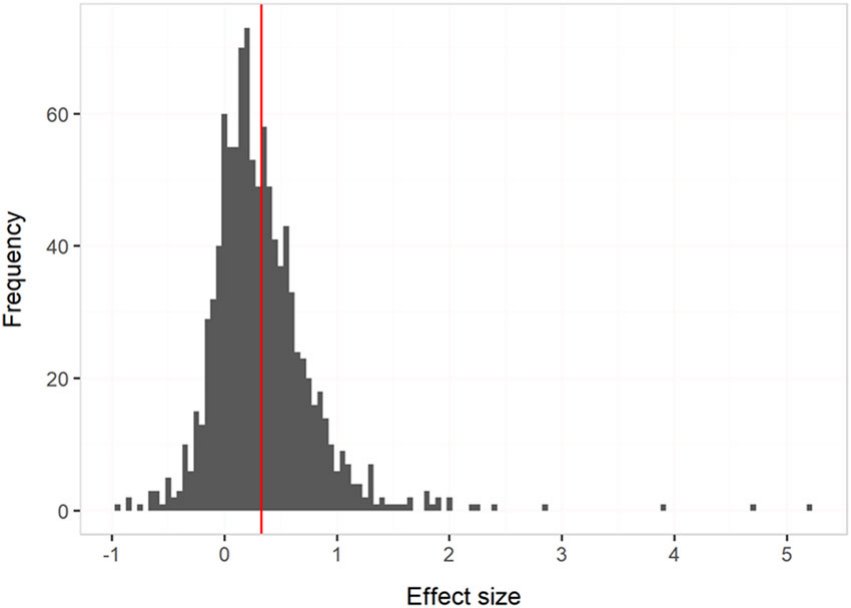
Distribution of short‐term effect sizes

Figure 5 displays the distribution of short‐term effect sizes. The figure underscores that the effect sizes are heterogeneous. Although most effect sizes are centred around the mean (the red line), there are examples of very large positive effect sizes as well as large negative effect sizes, indicating substantial heterogeneity.

Most studies did not report follow‐up effect sizes: there were 195 effect sizes from 27 studies measured more than 3 months after the end of intervention, which included 19,902 student observations. The weighted average follow‐up effect size was positive and significant (ES = 0.27, CI = [0.17, 0.36]). This effect size corresponds to a 57.5% chance that a randomly selected score of a student who received the intervention is greater than the score of a randomly selected student who did not. The *Q*‐statistic was 47.8 *(p *< .01), the *τ* ^2^ 0.03, and the *I* ^2^ was 45.6, which, although lower than for the short‐term effects, indicated that there was some systematic variation in the effect sizes and significant heterogeneity.^10^

The average follow‐up effect size was almost as large as the average short‐term effect size. The reason is not only that studies measured outcomes very close to 3 months after the end of intervention. Exploratory analyses revealed that the average effect size measured between 4 and 12 months after the end of intervention was ES = 0.26 (CI = [0.15, 0.37]) and between 12 and 24 months was ES = 0.17 (CI = [−0.03, 0.37]). These analyses included 22 and 9 studies, respectively. That is, the effects were still substantial also for the longer follow‐up periods. There were only 5 studies measuring effects after more than 24 months (ES = 0.11, CI = [−0.14, 0.36]) and the adjusted degrees of freedom fell below 4. This result was therefore unreliable.

It is possible that the follow‐up measurements are mainly confined to interventions that were successful in the short‐term (we found no example of a study with a protocol that detailed a follow‐up measurement in advance). We found an average short‐term effect size of ES = 0.40 (CI = [0.24, 0.55]) among the 22 studies that provided both a short‐term measure and at least one follow‐up measure. That this effect size was larger than the effect size for all studies is an indication that successful interventions are more likely to be examined with follow‐up tests. In turn, this may explain part of the similarity between the short‐term and follow‐up average effect sizes in our sample.

Studies with follow‐up measurements were also different in two other ways. All but five studies used small‐group instruction (i.e., student groups of five or below) and all but eight studies tested effects only on reading measures (two of the eight studies tested both math and reading). Among the exceptions, two studies used peer‐assisted instruction, one study used groups of max eight students, one study used CAI and incentives, and one study changed only the content domain. Thus, evidence of medium‐ to long‐term effects pertains almost exclusively to small‐group instruction interventions that examined the effects on reading measures. Confining the analysis of follow‐up effects (>3 months after end‐of‐intervention) to studies using small‐group instruction yields an ES = 0.28 (CI = [0.16, 0.39]) and confining the analysis further to include only interventions using small‐group instruction and testing effects on reading measures yields an ES = 0.27 (CI = [0.12, 0.42]).

Summarising the analysis thus far, we found evidence of reasonably large and statistically significant short‐term and follow‐up effects. All measures indicated substantial heterogeneity of the short‐term effects, which included a mix of intervention types. The evidence for the follow‐up effects pertains almost exclusively to studies examining small‐group instruction and to effects on reading measures, and we found few studies that have examined effects more than two years after the end of intervention. Because of the small number of studies, and the few instructional methods and content domains used in the follow‐up studies, we focused the subgroup analysis and investigation of heterogeneity in the following section on the short‐term effects.

#### 5.3.2 Results of the subgroup analysis and investigation of heterogeneity

The previous analyses indicated substantial heterogeneity of the short‐term effects. This section examines if we can explain some of this heterogeneity using subgroup and moderator analysis. The analysis follows the five‐step roadmap laid out in Section 4.3.10:

1. We first tested whether the effect sizes differ between math and reading tests, and then divided interventions into subgroups defined by instructional methods and content domains, and calculated the weighted average effect size for interventions that *included* a certain component (i.e., we used single‐factor subgroup analysis).
2. We examined interventions that included only one instructional method or one content domain and ran separate regressions using only effect sizes from these interventions (i.e., a single‐factor subgroup analysis using only single method and single domain interventions).
3. We ran multiple meta‐regressions on the full sample of short‐term effect sizes where we included indicators for each instructional method and content domain, as well as additional study characteristics. To retain enough (adjusted) degrees of freedom, we included only study characteristics without missing values. Table 2 contains the correlation coefficients between all the moderators.
4. We examined the effect sizes of the few recurring combinations of instructional methods.
5. The first four types of analyses indicated that there was strong evidence for substantial, stable, and statistically significant effect sizes of two instructional methods: peer‐assisted instruction and small‐group instruction. As our definitions of these instructional methods were relatively broad, we examined them further.

**Table 2:** Correlation matrix for moderators

	(1)	(2)	(3)	(4)	(5)	(6)	(7)	(8)	(9)	(10)	(11)	(12)	(13)	(14)	(15)	(16)	(17)	(18)	(19)	(20)	(21)	(22)	(23)	(24)	(25)	(26)	(27)
1	**1**																										
2	–0.07	**1**																									
3	0.06	–0.06	**1**																								
4	–0.09	–0.06	–0.04	**1**																							
5	–0.05	–0.03	–0.03	–0.03	**1**																						
6	–0.13	0.03	0.13	0	–0.04	**1**																					
7	0.11	0.07	0.03	–0.06	–0.04	0.05	**1**																				
8	–0.35	–0.01	–0.17	–0.31	–0.19	**–0.51**	–0.13	**1**																			
9	0.06	0.17	0.02	–0.03	–0.09	–0.07	–0.04	0.11	**1**																		
10	–0.05	0	–0.16	0.05	–0.02	–0.30	–0.01	0.23	0.32	**1**																	
11	–0.17	0.13	0.08	0.02	–0.10	−0.08	–0.07	0.20	**0.53**	0.29	**1**																
12	–0.14	0.07	–0.03	0	–0.09	–0.13	–0.08	0.24	**0.67**	0.47	**0.60**	**1**															
13	0.02	–0.03	–0.11	0.01	–0.03	–0.17	–0.02	0.12	0.16	0.37	0.09	0.43	**1**														
14	−0.03	0.14	–0.04	0.12	–0.04	–0.17	–0.11	0.07	**0.52**	0.29	0.42	0.45	0.09	**1**													
15	0	–0.03	–0.01	–0.05	−0.03	–0.01	0.02	0	–0.23	–0.30	–0.18	–0.31	–0.13	–0.15	**1**												
16	–0.05	0.01	0.01	–0.04	–0.02	–0.05	0.06	0.01	–0.15	–0.20	–0.13	–0.21	–0.09	–0.10	0	**1**											
17	0.09	–0.07	–0.07	–0.06	0.01	–0.01	0.01	–0.07	–0.24	–0.32	–0.20	–0.33	–0.14	–0.16	0.50	0.36	**1**										
18	0.06	−0.03	0.21	−0.05	0.05	0.04	0.10	−0.14	−0.43	**−0.57**	−0.35	**−0.55**	−0.25	−0.27	**0.53**	0.34	**0.57**	**1**									
19	0.10	−0.06	0.19	−0.08	−0.01	0.01	0.07	−0.08	−0.34	−0.44	−0.27	−0.45	−0.19	−0.21	0.12	0.30	0.42	**0.66**	**1**								
20	0.11	−0.05	0.16	−0.09	−0.01	0	0.11	−0.10	−0.37	−0.48	−0.30	−0.49	−0.21	−0.23	0.33	0.34	0.47	**0.76**	**0.72**	**1**							
21	0.01	−0.07	0.16	−0.06	−0.03	0	−0.02	0.01	−0.25	−0.33	−0.20	−0.34	−0.15	−0.15	**0.72**	−0.01	0.41	**0.57**	0.29	0.43	**1**						
22	−0.09	0.06	0.02	−0.05	0.07	−0.03	−0.06	0.02	−0.20	−0.25	−0.16	−0.18	−0.12	−0.13	−0.04	−0.03	−0.04	0.04	−0.03	−0.03	−0.05	**1**					
23	−0.08	0.1	0.32	−0.03	0.05	0.14	−0.03	−0.08	−0.07	−0.18	−0.10	−0.04	−0.14	0.02	−0.03	0	−0.04	0.03	0.07	0.03	0.03	0.20	**1**				
24	−0.08	0.04	−0.05	0.02	0.12	0.14	−0.04	−0.08	−0.18	−0.17	−0.07	−0.14	−0.11	−0.08	−0.04	−0.03	−0.04	0.01	−0.06	−0.07	−0.04	0.19	0.17	**1**			
25	−0.05	−0.07	0.06	0.17	0.05	0.04	−0.04	−0.04	−0.05	−0.20	−0.07	−0.05	0.05	0.01	0.06	−0.03	−0.05	0.05	−0.04	0	0.05	−0.04	−0.07	−0.04	**1**		
26	0.01	0.16	0.01	−0.05	0.15	0.16	0.13	−0.21	−0.16	−0.32	−0.20	−0.22	−0.11	−0.12	0.10	−0.03	0.02	0.19	0.22	0.09	0.03	0.31	0.15	0.18	0.13	**1**	
27	0.12	−0.01	0.04	0.14	0.02	0.19	0.02	−0.31	0.19	−0.21	0.08	0.15	0.03	0.08	0.05	0.06	−0.04	0.01	‐0.19	−0.12	0.04	0.04	0.13	0.14	0.05	0.11	**1**

*Note*: Correlation coefficients >0.5 are marked in bold. The rows and columns correspond to the following moderators: 1. CAI. 2. Coaching of personnel. 3. Incentives. 4. Medium‐group instruction. 5. Other method. 6. Peer‐assisted instruction. 7. Progress monitoring. 8. Small‐group instruction. 9. Comprehension. 10. Decoding. 11. Fluency. 12. Multiple reading. 13. Spelling and Writing. 14. Vocabulary. 15. Algebra/Pre‐algebra. 16. Fractions. 17. Geometry. 18. Multiple math. 19. Number sense. 20. Operations. 21. Problem solving. 22. General academic. 23. Meta‐cognitive. 24. Social‐emotional. 25. QES. 26. General test. 27. Mean Grade.

##### Math and reading tests and combined intervention components

We first estimated a model including all posttest effect sizes, an intercept representing effect sizes based on reading tests, and an indicator for math tests. The overall effect size for reading tests was 0.28 and on math tests 0.33, and the difference was not significant (math indicator CI = [−0.04, 0.15]). In the rest of this section, we pooled math and reading tests in the analysis.

**Figure 6:**
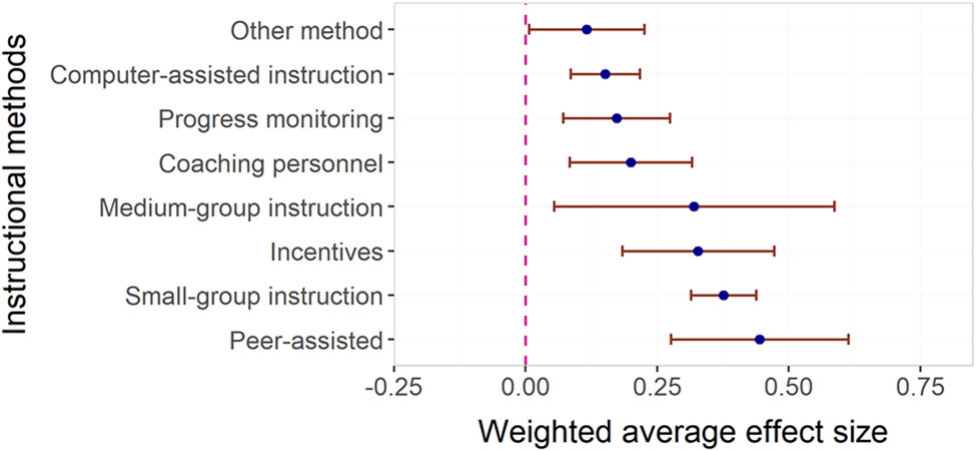
Subgroup analyses: Weighted average effect sizes and 95% confidence intervals by instructional method

**Figure 7:**
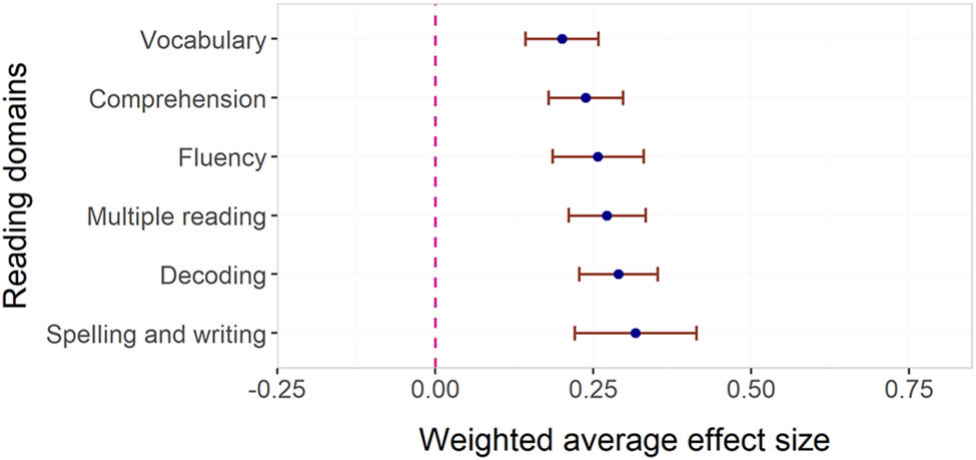
Subgroup analyses: Weighted average effect sizes and 95% confidence intervals by reading domain

**Figure 8:**
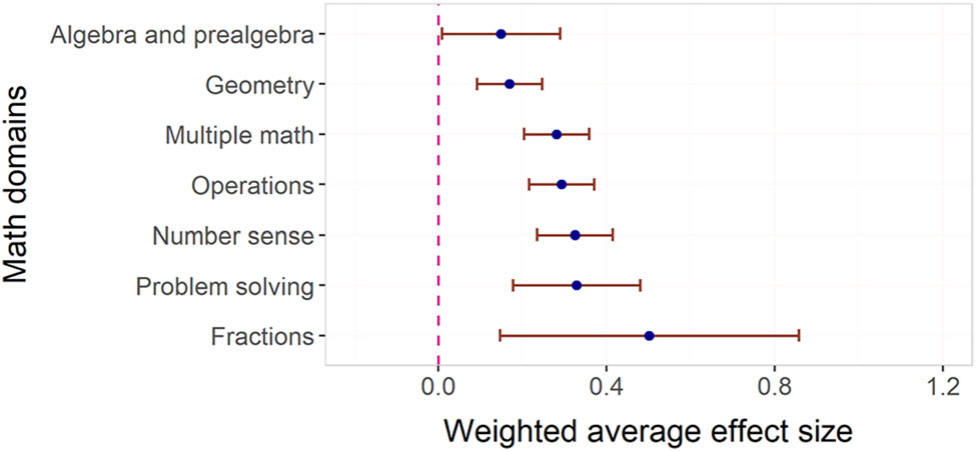
Subgroup analyses: Weighted average effect sizes and 95% confidence intervals by math domain

**Figure 9:**
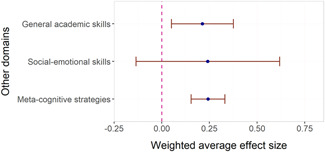
Subgroup analyses: Weighted average effect sizes and 95% confidence intervals other content domains

Figures 6, 7, 8, 9 show weighted average effect sizes and 95% CIs from RVE estimations by instructional method (Figure 6) and by content domain (Figure 7, 8, 9). We derived each effect size from a meta‐regression including just a constant with the outcome variable being the effect sizes from interventions including the component in question. Note that the effect sizes should be interpreted as the weighted average effect size for interventions that included a certain component, not the effect size of that component in isolation (see below for such estimates). Figure 6 indicates that all instructional methods were associated with positive and statistically significant average effect sizes. Peer‐assisted instruction is associated with the largest effect sizes (ES = 0.44, CI = [0.28, 0.61]) and other methods with the smallest effect sizes (ES = 0.12, CI = [0.01, 0.23]).

The average effect sizes of the reading domains are all reasonably close to each other in Figure 7. The effect sizes range from about 0.20 to 0.30, all of which are statistically significant. Figure 8 shows that the average effect sizes of the math domains varied more than the reading domains: from 0.14 for algebra/pre‐algebra domain to 0.47 for fractions (all effect sizes are statistically significant). The effect sizes for interventions targeting meta‐cognitive, social‐emotional, and general academic skills, shown in Figure 9, were also positive. However, the effect size for the social‐emotional domain was not significant.

Table 3 summarises the average effect sizes per component, the CIs, number of studies, effect sizes, student observations, the adjusted degrees freedom, and provides heterogeneity statistics. The adjusted degrees of freedom are above 4 for all components, but are relatively close for components examined in few studies, such as other methods, algebra/pre‐algebra, fractions, geometry, and general academic skills. Although the heterogeneity was reduced compared with the analysis of overall short‐term effect sizes, the average effect sizes for most components were still substantially heterogeneous. Partial exceptions were CAI (*τ* ^2^ **= 0.014, *I* ^2^ = 31.5, *Q* = 39.4), progress monitoring (*τ* ^2^ = 0.021, *I* ^2^ = 44.6, *Q* = 32.5), algebra/pre‐algebra (*τ* ^2^ = 0.024, *I* ^2^ = 47.1, *Q* = 22.7), and geometry (*τ* ^2^ = 0.028, *I* ^2^ **= 52.6, *Q* = 19.0), but the *Q*‐test indicated that heterogeneity was still statistically significant also for these components.

**Table 3:** Subgroup analyses: Effect sizes, confidence intervals, number of studies, effect sizes, and heterogeneity measures by intervention component

Component	Avg. ES	95% CI lower bound	95% CI upper bound	*K*	*n*	*N*	Adj. *df*s	*τ* ^2^	*I* ^2^	*Q*
CAI	0.151	0.086	0.217	28	171	29,910	19.6	0.014	31.5	39.4
Coaching	0.200	0.084	0.316	21	82	24,945	16.6	0.045	75.3	81.1
Incentives	0.328	0.184	0.472	19	78	30,812	16.5	0.068	67.2	54.9
Medium‐group	0.320	0.054	0.587	18	62	13,684	16.2	0.169	86.8	129.2
Other method	0.116	0.006	0.225	8	14	47,511	6.0	0.064	85.7	48.9
Peer‐assisted	0.444	0.276	0.613	32	97	8725	29.6	0.168	74.9	123.4
Progress mon.	0.173	0.071	0.274	19	102	23,164	11.7	0.021	44.6	32.5
Small‐group	0.376	0.314	0.438	118	756	89,295	107.4	0.088	75.3	472.9
Comprehension	0.238	0.179	0.297	73	538	69,469	58.8	0.041	62.3	191.2
Decoding	0.290	0.228	0.352	92	673	74,627	79.8	0.061	66.5	271.6
Fluency	0.258	0.186	0.329	56	432	56,055	47.3	0.053	65.9	161.3
Multiple reading	0.272	0.211	0.333	103	685	138,453	87.5	0.057	77.1	444.9
Spelling/writing	0.317	0.220	0.413	42	276	29,953	35.5	0.062	70.1	137.2
Vocabulary	0.200	0.143	0.258	55	325	52,181	42.5	0.029	58.2	129.3
Algebra	0.148	0.007	0.289	13	46	12,495	8.8	0.024	47.1	22.7
Fractions	0.501	0.146	0.857	6	22	4260	4.8	0.181	86.6	37.2
Geometry	0.169	0.092	0.246	10	53	11,112	6.6	0.028	52.6	19.0
Multiple math	0.281	0.203	0.358	48	149	87,328	41.6	0.061	79.4	227.9
Number sense	0.324	0.234	0.414	30	96	22,335	27.0	0.058	72.1	103.8
Operations	0.292	0.215	0.370	31	112	23,740	26.4	0.041	61.4	77.7
Problem solving	0.328	0.176	0.480	17	56	7399	14.3	0.057	58.5	38.5
Gen. academic	0.213	0.050	0.375	11	37	23,713	8.7	0.053	77.3	44.1
Meta‐cognitive	0.242	0.153	0.331	31	87	24,746	25.0	0.044	73.9	114.7
Social‐emotional	0.241	‐0.135	0.618	13	35	9186	11.6	0.216	87.3	94.3

*Note*: The number of studies contributing to the average effect size is denoted *k*, the number of effect sizes is denoted *n*, and the number of student observations is denoted *N. N* is calculated by taking the sample size for each effect size included in an estimation, which is equal to the number of students in the intervention and control group for which the effect size is calculated, and summing the sample sizes over effect sizes.

Abbreviations: CAI, computer‐assisted instruction; CI, confidence interval; ES, effect sizes.

In Supporting Information Appendix J: *Forest plots by intervention component*, we show forest plots corresponding to the analyses in Table 3. We estimated the forest plots using study‐level average effect sizes and the REML option in the R package *metafor* (Viechtbauer, 2010). This study‐level analysis provided a robustness check of the RVE procedure and made the forest plots more legible. The results corroborate the results reported here: the effect sizes are in all instances close to those in reported in Table 3. All estimates that are statistically significant in Table 3 are also significant in Supporting Information Appendix J, except for the other methods‐category. Averaging effect sizes by study reduces the heterogeneity. For example, the *Q*‐test is not significant for CAI, other method, progress monitoring, algebra/pre‐algebra, geometry, and operations in Supporting Information Appendix J. However, most components display a broad range of minimum and maximum values and the prediction intervals include or are at zero for all except CAI, and geometry.

The advantages of this analysis are that more studies can be included in each subgroup and that the effect sizes are more comparable with those estimated in some earlier reviews. As mentioned, there are also a few important drawbacks. If two or more components are included in an intervention, we cannot separately identify the association of any component. As shown in Table 1, several components were always used in combination with at least one other component. Table 2 contains the correlation coefficients between our moderators and indicates that some components are highly correlated, and that few correlations are zero. This risk was therefore pertinent. In the next section, we show estimates from interventions that contained a single instructional method or a single content domain to mitigate this risk.

##### Single instructional method and content domain interventions

Table 4 shows the results for the single instructional method and single domain estimations. Single method interventions examined interventions using only one of our categories of instructional methods. Single domain interventions targeted only one content domain. As some content domains were not studied in isolation in more than one study in our sample, we omitted them from Table 4. By definition, this analysis does not include the multiple domain‐categories. Furthermore, we omitted the other method category from the table, which is a single method (or no method) category already in Table 3.

**Table 4:** Subgroup analyses: Effect sizes, confidence intervals, number of studies, effect sizes, and heterogeneity measures by intervention component in single component studies

Component (single)	Avg. ES	95% CI lower bound	95% CI upper bound	*k*	*n*	*N*	Adj. *df*s	*τ* ^2^	*I* ^2^	*Q*
CAI	0.128	0.018	0.239	12	67	10,995	7.9	0.015	37.0	17.5
Coaching	−0.047	−0.878	0.783	3	4	2957	1.7	0.050	68.9	6.4
Incentives	0.046	−0.086	0.178	3	7	22,154	1.3	0.000	0.0	2.0
Medium‐group	0.091	−0.092	0.274	10	45	9219	7.4	0.044	61.0	23.1
Peer‐assisted	0.387	0.257	0.518	16	54	4208	12.6	0.023	19.8	18.7
Progress mon.	0.277	−3.085	3.638	2	4	90	1.0	0.000	0.0	1.0
Small‐group	0.375	0.304	0.446	85	538	51,445	76.7	0.084	70.6	285.4
Comprehension	0.205	−0.409	0.819	3	13	1504	1.7	0.021	23.6	2.6
Decoding	0.305	0.145	0.465	25	111	6653	22.9	0.148	66.5	71.6
Number sense	0.510	0.143	0.877	6	13	2296	4.5	0.110	82.1	27.9
Operations	0.165	−0.149	0.479	3	10	3653	1.7	0.049	54.5	4.4

*Note*: The number of studies contributing to the average effect size is denoted *k*, the number of effect sizes is denoted *n*, and the number of student observations is denoted *N. N* is calculated by taking the sample size for each effect size included in an estimation, which is equal to the number of students in the intervention and control group for which the effect size is calculated, and summing the sample sizes over effect sizes.

Abbreviations: CAI, computer‐assisted instruction; CI, confidence interval; ES, effect sizes.

Few single components were examined in enough studies for this strategy to give reliable results. Coaching of personnel, incentives, and progress monitoring as well as all content domains except decoding and number sense have adjusted degrees of freedom below 4. For the components that the analysis resulted in adjusted degrees of freedom above 4, the average effect sizes are mostly similar to the ones in Table 3. The exception is number sense, where the effect size in single domain interventions is quite a lot larger (0.51 instead of 0.32). Among the content domains, decoding was the only other statistically significant domain besides number sense. Both peer‐assisted instruction and small‐group instruction continued to be associated with large and statistically significant effect sizes (0.38 and 0.37, respectively). CAI had smaller (0.13) but statistically significant positive effects.

For most components, the number of studies also precluded conclusions about heterogeneity. Confining the comments to components examined in more than 2 studies, CAI and peer‐assisted instruction was examined in reasonably many studies (12 and 16), had relatively low *τ* ^2^ (0.015 and 0.023, respectively) and *I* ^2^ (37.0 and 19.8, respectively), and had a *Q*‐statistic that imply that we cannot reject the null hypothesis of no heterogeneity (*p* = .10 and *p* = .23, respectively). Medium‐ and small‐group instruction, decoding, and number sense had significant levels of heterogeneity.

This estimation strategy isolates the association between methods/domains and effect sizes better than the previous, but few components were examined in enough single method/single domain studies for this strategy to give reliable results. So far, neither analysis have adjusted for other study characteristics, and not for instructional methods and content domains at the same time, which is what we do in the next section.

##### Multiple meta‐regressions on the full sample of short‐term effect sizes

Table 5 shows results from four multiple meta‐regressions in which we included indicators for each instructional method and content domain, and, in some regressions, additional study characteristics. The table displays the coefficient estimates and 95% CIs (directly beside the coefficient estimates). To retain enough (adjusted) degrees of freedom, we included only study characteristics without missing information: the mean Grade (mean centred) and indicators for QES, general tests, and mathematics test.

**Table 5:** Results from multiple meta‐regressions

	(1)		(2)		(3)		(4)	
Moderator	Coef.	95% CI	Coef.	95% CI	Coef.	95% CI	Coef.	95% CI
*CAI*	0.10	[−0.02,0.21]	0.08	[−0.07, 0.23]	0.17	[−0.02, 0.36]	−0.14	[−0.28, 0.01]
*Coaching*	0.01	[−0.11,0.12]	−0.03	[−0.15, 0.09]	0.03	[−0.12, 0.17]	−0.23	[−0.40, −0.06]
*Incentives*	0.08	[−0.04,0.21]	0.09	[−0.06, 0.24]	0.06	[−0.08, 0.20]	0.07	[−0.08, 0.23]
*Medium‐group*	0.14	[−0.06,0.34]	0.18	[−0.10, 0.47]	0.28	[−0.07, 0.63]	−0.34	[−0.78, 0.11]
*Other method*	0.10	[−0.07,0.27]						
*Peer‐assisted*	0.41	[0.24,0.59]	0.40	[0.13, 0.67]	0.42	[0.07, 0.76]	0.38	[0.12, 0.65]
*Progress mon*.	−0.12	[−0.22,‐0.01]	−0.09	[−0.20, 0.02]	−0.09	[−0.21, 0.03]	−0.07	[−0.27, 0.13]
*Small‐group*	0.36	[0.26,0.47]	0.34	[0.12, 0.56]	0.32	[0.07, 0.58]	0.24	[0.11, 0.37]
*Comprehension*	0.00	[−0.15,0.14]	0.04	[−0.10, 0.18]				
*Decoding*	0.04	[−0.07,0.15]	0.01	[−0.11, 0.13]				
*Fluency*	−0.06	[−0.20,0.08]	−0.07	[−0.20, 0.06]				
*Multiple reading*	0.00	[−0.15,0.14]						
*Spelling/writing*	0.11	[−0.02,0.23]	0.11	[0.00, 0.23]				
*Vocabulary*	−0.08	[−0.19,0.03]	−0.07	[−0.19, 0.05]				
*Algebra*	−0.13	[−0.27,0.02]	−0.16	[−0.31, −0.01]				
*Fractions*	0.33	[0.01,0.65]	0.37	[0.06, 0.67]				
*Geometry*	0.03	[−0.10,0.15]	0.04	[−0.15, 0.22]				
*Multiple math*	−0.01	[−0.23,0.21]						
*Number sense*	0.09	[−0.09,0.27]	−0.02	[−0.20, 0.16]				
*Operations*	−0.08	[−0.26,0.10]	−0.10	[−0.27, 0.07]				
*Problem solving*	0.05	[−0.07,0.17]	0.08	[−0.04, 0.19]				
*Gen. academic*	−0.05	[−0.24,0.15]	−0.09	[−0.33, 0.15]				
*Meta‐cognitive*	−0.05	[−0.20,0.10]	0.00	[−0.14, 0.13]				
*Social‐emotional*	−0.07	[−0.38,0.25]	−0.07	[−0.39, 0.24]				
*QES*			−0.04	[−0.22, 0.15]	0.04	[−0.14, 0.21]	0.03	[−0.40, 0.46]
*General test*			0.07	[−0.10, 0.25]	0.07	[−0.13, 0.26]	−0.03	[−0.19, 0.12]
*Mean Grade*			−0.03	[−0.06, −0.00]	−0.04	[−0.07, *−*0.02]	−0.01	[−0.05, 0.02]
*Math*			0.07	[−0.12, 0.26]				
*Constant*			0.00	[−0.21, 0.20]	−0.03	[−0.31, 0.24]	0.19	[0.01, 0.37]
Effect sizes	1030		1030		829		199	
Study clusters	189		189		138		64	
*N*	206,186		206,186		132,046		74,608	
*Q*	481.0		430.8		343.3		142.6	
*I* ^2^	65.7		62.2		63.0		62.8	
τ ^2^	0.063		0.053		0.051		0.047	

*Note*: Coefficients from multiple meta‐regressions and 95% CIs (in brackets) are presented. Column 1 includes indicators for all components but no constant and no additional study characteristics. Column 2 adds a constant and the study characteristics without missing values, and excluded the indicators for other method, multiple reading, and multiple math to get a reference category. Columns 3 and 4 separate the analysis into effect sizes based on reading tests (column 3) and based on mathematics tests (column 4) and leave out all content domains. The number of studies contributing to the average effect size is denoted *k*, the number of effect sizes is denoted *n*, and the number of student observations is denoted *N*.

Abbreviations: CAI, computer‐assisted instruction; CI, confidence interval; ES, effect sizes.

Column 1 presents results from a specification including indicators for all components but no constant and no additional study characteristics. This specification reports the total marginal association between each component and effect sizes, conditional on all other components. Among the instructional methods, there are two statistically significant associations with effect sizes: peer‐assisted instruction (*β* = .41) and small‐group instruction (*β* = .36). Medium‐group instruction, CAI, and other method have insignificant associations slightly above 0.1, while all other methods have smaller positive or negative associations (all insignificant). Fractions is the only content domain with a significant positive association with effect sizes (*β* = .33). The coefficients for most other domains are close to zero, only spelling and writing is above 0.1. All component indicators have adjusted degrees of freedom above 4 and the only component close to 4 is fractions (adjusted degrees of freedom = 4.3), which, as seen in Table 3, has been examined in only 6 studies.

In column 2, we added a constant and the study characteristics without missing values, and excluded the indicators for other method, multiple reading, and multiple math to get a reference category and avoid multicollinearity. The total marginal association for a specific component is therefore the sum of the constant and the coefficient on the component. However, as the constant is virtually 0, the total marginal association can be read off the individual coefficients.

Adding study characteristics did not change the results much. Spelling and writing has a positive and statistically significant association and algebra/pre‐algebra a negative and statistically significant association, but the size of the total marginal associations are close to those in column 1. Both the size and significance of the other instructional methods and content domains are close to the results in column 1 with the partial exception of number sense, for which the association decreases by around 0.1 (it is still insignificant).

Mean Grade is the only statistically significant study characteristic. The coefficient indicates that interventions in higher Grades have smaller effect sizes (about −0.03 per Grade). The results for the other study characteristics indicate that effect sizes from QES were not significantly different from RCTs, general tests did not have significantly different effect sizes from tests of subdomains, and effect sizes based on math tests were not significantly different from those based on reading tests. All included variables have adjusted degrees of freedom above 4 (with fractions again being closest to 4, with a value of 5.3).

In columns 3 and 4, we report results where we separated the analysis into effect sizes based on reading tests (column 3) and based on mathematics tests (column 4) and leave out all content domains. Although we found no strong indication that effect sizes were different between the two subjects in column 2, associations for specific instructional methods may still differ across subjects. Indeed, there are some differences: both CAI and coaching of personnel have positive associations with effect sizes based on reading tests, and negative associations with effect sizes based mathematics tests. Only the coefficient for coaching on math tests is significant. Note however that the constant is much larger in column 4 compared with column 3, meaning that the differences across the specifications between the total marginal associations for CAI and coaching of personnel are smaller. The association between medium‐group instruction and effect sizes also shifts from positive and relatively large for reading tests to negative and relatively large for mathematics tests. However, neither of the two coefficients is statistically significant and the adjusted degrees of freedom falls below 4 in column 4. Otherwise, all included variables in both columns 3 and 4 have adjusted degrees of freedom above 4. Lastly, the coefficients on peer‐assisted instruction and small‐group instruction are reasonably similar across the specifications, large, and statistically significant.

As there were few content domains with large, stable, and significant associations with effect sizes and few instructional methods with enough studies/effect sizes, we refrained from using a similar specification for content domains. The systematic variation in effect sizes, as measured by the *I* ^2^, was between 62 and 66 throughout the specifications. Furthermore, both the *Q* and τ ^2^ statistics indicated substantial heterogeneity.

Table 5 reports whether the coefficients (marginal associations) are significantly different from zero, but not whether they are significantly different from each other. In Table 6, we report results where we used the most comprehensive specification (column 2) of Table 5 to examine if coefficients are significantly different from each other (see the note below the table for details on how we implemented the test). To keep the table at a manageable length, we focused on the three components with statistically significant coefficients that were stable across specifications: peer‐assisted instruction, small‐group instruction, and fractions. We compared peer‐assisted instruction and small‐group instruction to the other instructional methods and fractions with the other math domains.

**Table 6:** Tests of differences between intervention components

	Coefficient difference	*F*‐statistic	*df*	*p* Value
Hypothesis	(1)	(2)	(3)	(4)
Peer‐assisted = CAI	0.31	10.65	51.76	0.002
Peer‐assisted = Coaching personnel	0.42	8.84	43.26	0.005
Peer‐assisted = Incentives	0.31	8.49	33.26	0.006
Peer‐assisted = Medium‐group	0.21	4.40	34.30	0.043
Peer‐assisted = Progress monitoring	0.49	9.28	43.48	0.004
Peer‐assisted = Small group	0.06	0.91	45.58	0.345
Small‐group = CAI	0.26	13.51	35.75	0.001
Small‐group = Coaching personnel	0.37	9.08	48.32	0.004
Small‐group = Incentives	0.25	8.46	27.60	0.007
Small‐group = Medium‐group	0.15	3.58	25.21	0.070
Small‐group = Progress monitoring	0.43	10.45	38.39	0.003
Fractions = Algebra/Pre‐algebra	0.53	14.15	10.18	0.004
Fractions = Geometry	0.33	6.12	10.21	0.032
Fractions = Number sense	0.39	8.12	7.16	0.024
Fractions = Operations	0.47	10.09	6.29	0.018
Fractions = Problem solving	0.29	6.21	8.95	0.035

Column 1 reports the difference between the coefficients of the two components mentioned in the Hypothesis‐column. To calculate the *F*‐statistic, the degrees of freedom, and the (two‐sided) *p* value in columns 2–4, we used the test described in Tipton and Pustejovsky (2015), and implemented it using our own extension to the Wald_test function in R package clubSandwich (Pustejovsky, 2020) and the “HTZ” small‐sample correction procedure. The coefficients and variance estimates are from the model reported in Table 5, column 2.

Abbreviation: CAI, computer‐assisted instruction.

Peer‐assisted instruction and small‐group instruction were not significantly different from one another. Peer‐assisted instruction was associated with significantly larger effect sizes than all other instructional methods. Small‐group instruction was associated with significantly larger effect sizes than all but one instructional method, medium‐group instruction where *p* = .07. Fractions were associated with significantly larger effect sizes than all other math domains. Note that we are testing multiple hypotheses here and that the reported *p* values are not adjusted for the number of tests. As we did not know the type of tests and number of hypotheses to be tested beforehand, our protocol did not specify an adjustment procedure.

The multiple meta‐regressions provide an estimate of the isolated association between each component and effect sizes, conditional on other components and study characteristics. As discussed in Section 4.3.10, it is difficult to rule out that we did not introduce bias by including moderators in the regressions. Furthermore, we were unable to include interactions between components in these regressions. The reason is that there were few recurring combinations in our sample. In the next section, we therefore used subgroup analysis to examine the effect sizes of these recurring combinations.

##### Specific combinations of instructional methods

We found few recurring combinations of instructional methods in our data. Only two analyses of pairs of instructional methods produced adjusted degrees of freedom above 4: coaching of personnel and small‐group instruction, and incentives and small‐group instruction. The first combination, examined in nine studies, had a lower average effect size (ES = 0.31, CI = [0.10, 0.52]) than small‐group instruction alone (compare Table 4). The incentives and small‐group instruction combination, examined in seven studies, had a larger effect size than small‐group instruction alone (ES = 0.47, CI = [0.36, 0.58]). We found no combination of three or more instructional methods examined in more than two studies.

##### Peer‐assisted instruction and small‐group instruction

The only two instructional methods with stable, large, and statistically significant associations with effect sizes in the previous analyses were peer‐assisted instruction and small‐group instruction. Our definitions of the peer‐assisted and small‐group instruction categories are relatively broad and they may contain diverse interventions. We therefore examined them further in this section. We focused on single method interventions and short‐term effects to better isolate the contribution of the instructional method and reduce heterogeneity due to measurement timing.

Most other instructional methods have been studied in few single method interventions and were part of few recurring combinations. Partial exceptions were CAI (examined in 12 studies of single method interventions) and medium‐group instruction (examined in 10 studies of single method interventions). The multiple meta‐regressions indicated that both these methods had different associations with effect sizes based on math and reading tests. However, only 4 out of 12 CAI studies and only 1 out of 10 medium‐group studies tested effects using math tests.^11^ Examining this issue further was therefore difficult. All content domains that the previous meta‐regressions indicated were associated with larger effect sizes have been examined in few single domain interventions.

The peer‐assisted instruction category is less diverse than it may seem from our definition. Only 3 out of 16 single method studies used cross‐age peer‐tutoring, the rest was interventions where same‐age peers worked together (often called cooperative learning). The average effect size for the 13 same‐age peers studies was slightly smaller than the one reported in Table 4 for peer‐assisted instruction, but still large and statistically significant (ES = 0.32, CI = [0.25, 0.42]). The average effect size in the three cross‐age peer‐tutoring studies was large (ES = 0.85, CI = [−0.36, 2.1]) but the adjusted degrees of freedom was below 4 and the result was unreliable. Four studies used larger peer‐groups than pairs. The effect size was larger when pairs were used (ES = 0.42, CI = [0.27, 0.58]) than when larger groups were used (ES = 0.28, CI = [−0.22, 0.78]). The effect size in the 6 peer‐assisted instruction studies using math tests was larger (ES = 0.54, CI = [0.06, 1.0]) than the effect size in the 13 studies testing reading (ES = 0.33, CI = [0.22, 0.44]), but the degrees of freedom were only 4.5 in the analysis of math tests so the result should be viewed with caution. The uniformity of peer‐assisted interventions may be one explanation of the relatively low level of heterogeneity reported in Table 4. Furthermore, the prediction interval based on the analysis Table 4 does not include zero, but ranges from 0.06 to 0.71.

Adult‐led small‐group instruction is a more diverse and larger category of interventions than peer‐assisted instruction (there were 85 studies of single method small‐group instruction interventions). The heterogeneity reported in Table 4 is also substantial, and a prediction interval based on this analysis ranges from −0.20 to 0.95. Interventions in this group of studies targeted either subjects like reading and mathematics or non‐subject‐specific areas like social‐emotional skills. Although it is difficult to draw a sharp line between the two, the former are usually some form of tutoring while the latter are more often called mentoring. However, if we define mentoring as interventions that do not target any subject‐specific domain, our sample only includes three studies of such interventions. Thus, with these definitions of tutoring and mentoring, our results for small‐group instruction mainly pertains to tutoring interventions. The effect sizes in single method small‐group interventions were reasonably similar in math (37 studies, ES = 0.39, CI  = [0.30, 0.48]) and reading (65 studies, ES = 0.34, CI = [0.26, 0.42]).

We therefore combined the subjects and focused the further examination on group sizes and additional study characteristics. Medium‐group instruction is only different from small‐group instruction by our definition of small and medium, and we therefore included single medium‐group instruction interventions in this examination as well to increase statistical power (10 studies). In total, this left us with 95 studies, 157 interventions, and 583 effect sizes.

In column 1 of Table 7, we split up the small‐group instruction group into three: instruction one‐to‐one, one‐to‐two or three, and one‐to‐four or five. The reference category in this specification, and in column 2 of this table, is the medium‐group instruction category, where groups range from 6 to 20 (or are of unclear size). There were 67 interventions of one‐to‐one instruction, 48 of one‐to‐two or three, 35 of one‐to‐four or five, and 11 in the medium‐group category. Group sizes sometimes vary between interventions within studies and some interventions used more than one group size (e.g., both one‐to‐one and one‐to‐two), so the sum count to more than 157, although no intervention use both small‐group and medium‐group instruction. The small‐group instruction categories are all associated with larger effect sizes than the medium‐group category, although the differences are not significant. The coefficients on the one‐to‐one, one‐to‐two or three, and one‐to‐four or five are close to one another.

**Table 7:** Group sizes in small‐ and medium‐group instruction interventions

	Excl study characteristics	Incl study characteristics
Moderator	(1)	(2)
One‐to‐one	0.171	0.077
	[−0.035, 0.377]	[−0.124, 0.278]
One‐to‐two or three	0.199	0.081
	[−0.015, 0.414]	[−0.150, 0.311]
One‐to‐four or five	0.169	0.015
	[−0.066, 0.404]	[−0.238, 0.268]
QES		−0.059
		[−0.327, 0.208]
Math		0.078
		[−0.095, 0.252]
General		0.030
		[−0.155, 0.217]
Mean Grade		−0.048
		[−0.084, −0.011]
Duration		−0.003
		[−0.008, 0.003]
Constant	0.180	0.260
	[−0.010, 0.369]	[0.06, 0.456]
Effect sizes	583	583
Clusters	95	95
*N*	60,664	60,664
*Q*	304.0	272.1
*I* ^2^	70.1	68.4
τ ^2^	0.08	0.08

*Note*: Coefficients and 95% confidence intervals (in brackets) from multiple meta‐regressions using *robumeta* with all short‐term effect sizes from single method medium‐group and small‐group instruction interventions are reported. The specification in column 1 includes a constant, which is mean effect size in the reference category (medium‐group intervention effect sizes), and indicators for one‐to‐one, one‐to‐two or three, and one‐to‐four or five small‐group instruction. The specification in column 2 adds study characteristics without missing values.

Abbreviation: QES, quasi‐experimental studies.

Column 2 adds the study characteristics without missing values, which for these interventions include duration. Adding study characteristics make the differences between the small‐group categories and the medium‐group category even smaller. Only the mean Grade is statistically significant among the study characteristics. As in the full sample, the coefficient is negative and indicates that interventions in higher Grades are associated with smaller effects. Note further that the *I* ^2^, *τ* ^2^, and *Q*‐statistics indicate that there was still systematic variation and substantial heterogeneity in this restricted sample.

Although this analysis tried to isolate associations between group size and effect sizes, the regressions are unlikely to uncover the causal effect of group size reductions. For example, one‐to‐one tutoring may be used for students with the greatest academic difficulties whereas larger groups are used for students with less grave difficulties. As mentioned, we were unable to control directly for the students’ level of difficulties. Therefore, if it is harder to improve the achievement of the group with the greatest difficulties or if group sizes work differently depending on the level of difficulties, then the group size associations are confounded by the students’ level of difficulties.

A better way to examine the effects of group size is to use comparison designs and meta‐analyse interventions that changed the group size, while keeping everything else constant. That is, studies that assign the group size randomly or quasi‐experimentally. However, we found only four studies that contrasted a one‐to‐one tutoring programme with the same, or a highly similar, programme using groups of two to five students (three RCTs and one QES). Running a meta‐analysis on these four studies yielded a negative (indicating an advantage of one‐to‐one tutoring) but far from significant effect size (ES = −0.14, CI = [−0.70, 0.42], 4 studies, 24 effect sizes, 658 student observations). Moreover, the adjusted degrees of freedom was below 4. While the heterogeneity statistics did not indicate significant heterogeneity, the number of studies likely imply that the estimations and the statistics are unreliable and the test of heterogeneity underpowered.

As mentioned, RVE may have trouble estimating the heterogeneity and standard errors when the number of studies is very small. However, the conclusion about group sizes was not sensitive to changes of the specification. We used study‐level averages to estimate the between‐study variance (using the REML option in the R package *metafor*), adjusted for pretest differences in the one study for which we based the effect sizes on raw means, and excluded the one intervention that used groups of five students (all others contrasted one‐to‐one with groups of two or three). The results were very similar (see Supporting Information Appendix K: *Extra sensitivity analyses*).

### 5.4 Results of sensitivity analyses

The sections below report results from our sensitivity analyses. We focused the sensitivity analyses on our main results: we found positive, substantial, and statistically significant overall average short‐term and follow‐up effect sizes, and that peer‐assisted instruction and small‐group instruction had large, stable, and statistically significant average effect sizes across specifications. For all other instructional methods and all content domains, our results were either based on few studies, particularly of single method or single domain interventions, or were not stable across specifications. We are therefore hesitant to make conclusions about their effectiveness and, as finding that the results are sensitive would not change any conclusions, we did not run the sensitivity tests for them.

We tested whether effect sizes were associated with effect size measurement, whether they were sensitive to adjusting for outliers and different adjustment for clustered assignment of treatment, to multiple imputation of moderators with missing values, and to adjusting for the risk of bias ratings. We also examined whether there were signs of heterogeneity across control group conditions, and finally, if there were indications of publication bias.

**Figure 10:**
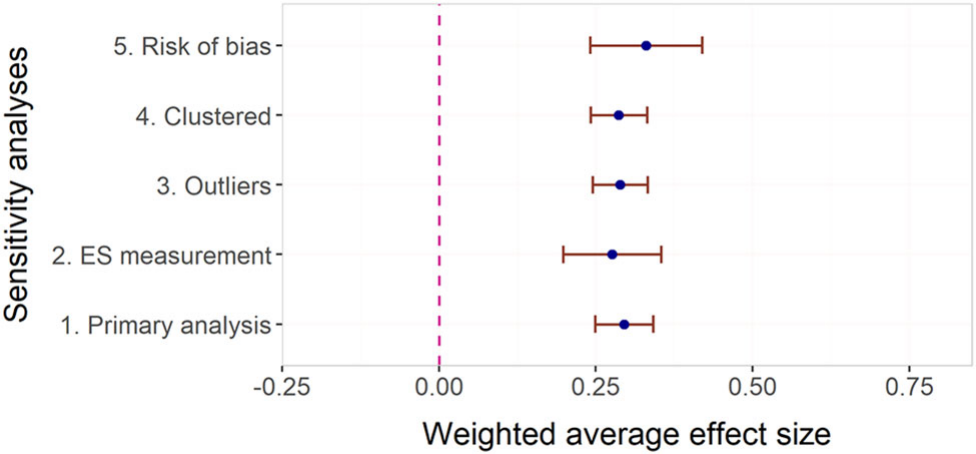
Sensitivity analyses: Overall short‐term average effect size

**Figure 11:**
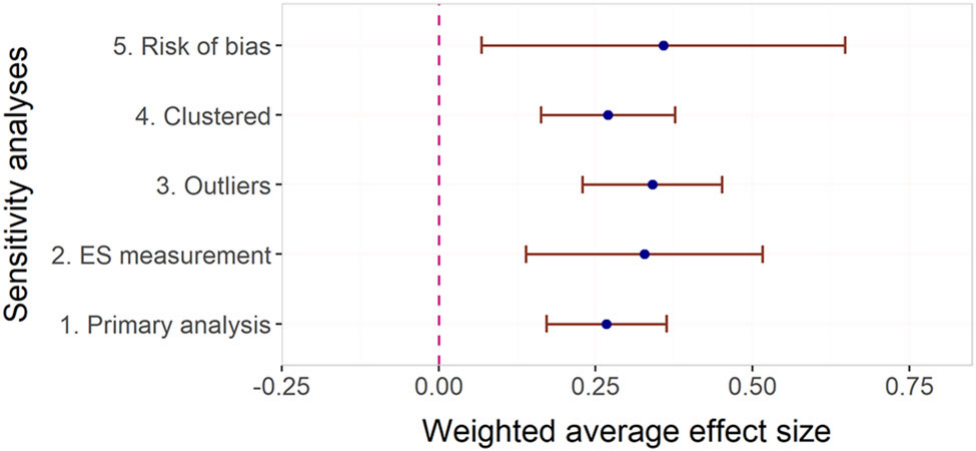
Sensitivity analyses: Overall follow‐up average effect size

**Figure 12:**
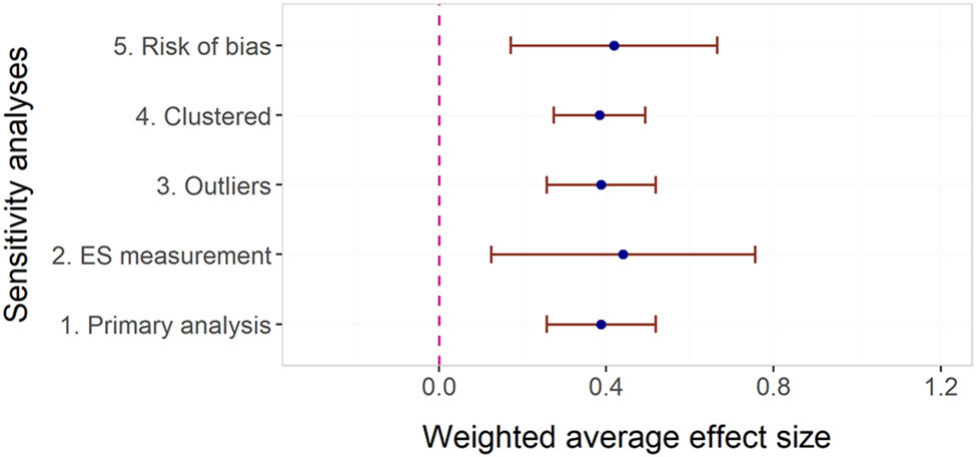
Sensitivity analyses: Peer‐assisted instruction

**Figure 13:**
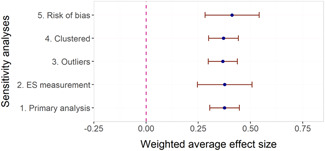
Sensitivity analyses: Small‐group instruction

We present some of the results of these sensitivity analyses in four figures (Figures 10, 11, 12, 13), corresponding to overall short‐term effects, overall follow‐up effects, peer‐assisted instruction, and small‐group instruction. Each figure has the effect size and its CI from the primary analysis at the bottom of the figure for easy reference. As we believe the single method interventions have the best chances of isolating the effects of instructional methods, we used them in the analyses of peer‐assisted instruction and small‐group instruction. We confined the sensitivity analysis to short‐term effects for peer‐assisted and small‐group instruction, but recall that the follow‐up effects were largely from small‐group instruction interventions.

Table 8 reports the results from the sensitivity analyses of effect size measurement, outliers, clustered assignment of treatment, and risk of bias using the most comprehensive specification among our meta‐regressions (reported in column 2 of Table 5). As these meta‐regressions are the only specifications including moderators in the primary analysis, we conducted the multiple imputation of moderators using this specification. Similarly, we examined the heterogeneity of control group progression using this specification. We report multiple meta‐regressions from the latter two types of analyses in Table 9. We comment on the figures and table by type of sensitivity analysis below.

**Table 8:** Sensitivity analysis of effect size measurement, outliers, clustered assignment of treatment and risk of bias items using multiple meta‐regressions

	ES measurement		Outliers		Clustered		Risk of bias	
Moderator	Coef.	95% CI	Coef.	95% CI	Coef.	95% CI	Coef.	95% CI
*CAI*	0.09	[−0.07, 0.25]	0.07	[−0.06, 0.20]	0.07	[−0.08, 0.22]	0.07	[−0.11, 0.25]
*Coaching*	−0.02	[−0.14, 0.10]	−0.02	[−0.14, 0.09]	−0.03	[−0.15, 0.08]	−0.02	[−0.15, 0.10]
*Incentives*	0.09	[−0.06, 0.25]	0.08	[−0.06, 0.22]	0.09	[−0.06, 0.23]	0.08	[−0.09, 0.26]
*Medium‐group*	0.18	[−0.11, 0.47]	0.15	[−0.07, 0.38]	0.17	[−0.09, 0.43]	0.21	[−0.20, 0.62]
*Peer‐assisted*	0.40	[0.12, 0.69]	0.36	[0.14, 0.59]	0.39	[0.13, 0.64]	0.39	[0.04, 0.73]
*Progress mon*.	−0.09	[−0.20, 0.03]	−0.08	[−0.19, 0.02]	−0.08	[−0.19, 0.02]	−0.09	−[0.22, 0.03]
*Small‐group*	0.34	[0.11, 0.57]	0.31	[0.14, 0.48]	0.32	[0.11, 0.53]	0.33	[0.06, 0.59]
*Comprehension*	0.03	[−0.12, 0.17]	0.04	[−0.09, 0.17]	0.04	[−0.09, 0.18]	0.01	[−0.15, 0.16]
*Decoding*	0.00	[−0.12, 0.13]	0.01	[−0.10, 0.13]	0.00	[−0.12, 0.12]	0.05	[−0.09, 0.20]
*Fluency*	−0.05	[−0.19, 0.08]	−0.06	[−0.19, 0.06]	−0.06	[−0.19, 0.06]	−0.06	[−0.21, 0.10]
*Spelling/writing*	0.11	[−0.01, 0.22]	0.11	[0.00, 0.22]	0.12	[0.01, 0.24]	0.09	[−0.03, 0.22]
*Vocabulary*	−0.07	[−0.19, 0.05]	−0.07	[−0.18, 0.04]	−0.06	[−0.17, 0.05]	−0.08	[−0.20, 0.05]
*Algebra*	−0.16	[−0.31, −0.01]	−0.16	[−0.31, −0.01]	−0.15	[−0.30, −0.01]	−0.17	[−0.35, 0.02]
*Fractions*	0.37	[0.06, 0.67]	0.36	[0.05, 0.67]	0.37	[0.09, 0.65]	0.33	[0.05, 0.62]
*Geometry*	0.03	[−0.17, 0.22]	0.02	[−0.15, 0.18]	0.03	[−0.15, 0.22]	0.04	[−0.20, 0.28]
*Number sense*	−0.01	[−0.19, 0.18]	−0.02	[−0.19, 0.16]	−0.01	[−0.18, 0.16]	−0.09	[−0.27, 0.10]
*Operations*	−0.11	[−0.28, 0.06]	−0.10	[−0.26, 0.06]	−0.10	[−0.26, 0.06]	−0.09	[−0.27, 0.09]
*Prob. solving*	0.07	[−0.04, 0.19]	0.08	[−0.04, 0.19]	0.07	[−0.04, 0.19]	0.10	[−0.04, 0.24]
*Gen. academic*	−0.08	[−0.32, 0.16]	−0.09	[−0.28, 0.11]	−0.06	[−0.28, 0.16]	−0.12	[−0.36, 0.12]
*Meta‐cognitive*	−0.02	[−0.17, 0.13]	0.01	[−0.11, 0.13]	0.00	[−0.14, 0.14]	0.01	[−0.13, 0.16]
*Social‐emotional*	−0.08	[−0.41, 0.25]	−0.10	[−0.36, 0.16]	−0.09	[−0.40, 0.23]	−0.09	[−0.43, 0.24]
*QES*	−0.04	[−0.22, 0.15]	−0.02	[−0.19, 0.15]	−0.05	[−0.24, 0.15]		
*General test*	0.06	[−0.11, 0.24]	0.06	[−0.09, 0.20]	0.07	[−0.10, 0.24]	0.08	[−0.12, 0.27]
*Mean Grade*	−0.03	[−0.06, ‐0.00]	−0.03	[−0.06, −0.01]	−0.03	[−0.06, −0.01]	−0.03	[−0.05, 0.00]
*Math*	0.07	[−0.12, 0.26]	0.08	[−0.08, 0.24]	0.07	[−0.12, 0.26]	0.11	[−0.10, 0.33]
*Raw mean*	0.03	[−0.06, 0.13]						
*Glass's δ*	0.24	[−0.01, 0.48]						
*Unclear ES type*	−0.16	[−1.11, 0.80]						
*Super‐pop*.	0.01	[−0.20, 0.22]						
*Blinding*							0.01	[−0.08, 0.10]
*Incomplete out*.							−0.04	[−0.17, 0.09]
*Reporting*							−0.05	[−0.16, 0.07]
*Other bias*							0.02	[−0.09, 0.13]
*Constant*	−0.02	[−0.24, 0.21]	0.01	[−0.16, 0.18]	0.01	[−0.19, 0.20]	0.00	[−0.26, 0.26]
Effect sizes	1030		1030		1030		981	
Study clusters	189		189		189		172	
*N*	206,186		206,186		206,186		152,581	
*Q*	412.1		382.3		488.0		385.3	
*I* ^2^	61.4		57.4		66.6		62.9	
τ ^2^	0.056		0.043		0.048		0.058	

Abbreviations: CAI, computer‐assisted instruction; CI, confidence interval; ES, effect sizes; QES, quasi‐experimental studies.

**Table 9:** Multiple imputation, heterogeneity of control group progression, publishing status, and funnel plot asymmetry

	Multiple imputation		Control progression		Publishing status		Asymmetry	
Moderator	Coef.	95% CI	Coef.	95% CI	Coef.	95% CI	Coef.	95% CI
*CAI*	0.08	[−0.07, 0.21]	−0.08	[−0.39, 0.23]	0.07	[−0.07, 0.21]	0.05	[−0.09, 0.19]
*Coaching*	−0.03	[−0.15, 0.10]	0.62	[−0.22, 1.46]	−0.02	[−0.15, 0.10]	0.04	[−0.09, 0.16]
*Incentives*	0.09	[−0.06, 0.24]	−0.23	[−0.62, 0.16]	0.09	[−0.06, 0.24]	0.10	[−0.05, 0.24]
*Medium‐gr*.	0.18	[−0.10, 0.43]	0.07	[−0.27, 0.40]	0.19	[−0.10, 0.48]	0.17	[−0.10, 0.44]
*Peer‐assist*.	0.38	[0.13, 0.61]	−0.02	[−0.25, 0.28]	0.40	[0.13, 0.66]	0.40	[0.13, 0.68]
*Progress*	−0.08	[−0.19, 0.04]	0.20	[−0.15, 0.55]	−0.10	[−0.21, 0.02]	−0.08	[−0.20, 0.04]
*Small‐group*	0.34	[0.12, 0.54]	0.17	[−0.12, 0.45]	0.34	[−0.12, 0.55]	0.30	[0.10, 0.50]
*Comprehen*.	0.04	[−0.10, 0.18]	−0.30	[−0.62, 0.02]	0.04	[−0.10, 0.18]	0.04	[−0.10, 0.17]
*Decoding*	0.01	[−0.13, 0.11]	−0.09	[−0.52, 0.33]	0.02	[−0.10, 0.14]	−0.01	[−0.13, 0.11]
*Fluency*	−0.07	[−0.18, 0.07]	0.12	[−0.14, 0.38]	−0.07	[−0.20, 0.06]	−0.07	[−0.20, 0.05]
*Spell./writing*	0.11	[0.00, 0.24]	0.05	[−0.22, 0.32]	−0.12	[0.00, 0.23]	0.10	[−0.01, 0.22]
*Vocabulary*	−0.06	[−0.16, 0.07]	0.09	[−0.24, 0.42]	−0.08	[−0.19, 0.04]	−0.06	[−0.18, 0.05]
*Algebra*	−0.16	[−0.33, −0.01]	−0.66	[−1.48, 0.16]	−0.16	[−0.31, −0.01]	−0.17	[−0.33, −0.02]
*Fractions*	0.36	[0.08, 0.62]	0.07	[−0.75, 0.90]	0.37	[0.07, 0.68]	0.38	[−0.08, 0.67]
*Geometry*	0.06	[−0.11, 0.25]	−0.11	[−0.82, 0.59]	0.04	[−0.15, 0.22]	0.04	[−0.16, 0.24]
*Number sen*.	−0.02	[−0.19, 0.15]	0.57	[0.10, 1.03]	−0.02	[−0.21, 0,17]	−0.03	[−0.20, 0.15]
*Operations*	−0.10	[−0.29, 0.05]	−0.05	[−0.65, 0.55]	−0.09	[−0.27, 0,09]	−0.09	[−0.26, 0.07]
*Prob. solving*	0.07	[−0.04, 0.19]	0.18	[−0.50, 0.87]	0.08	[−0.04, 0.21]	0.08	[−0.03, 0.19]
*Gen. acad*.	−0.09	[−0.30, 0.17]	0.13	[−0.60, 0.86]	−0.08	[−0.31, 0.16]	−0.09	[−0.34, 0.15]
*Meta‐cog*.	−0.01	[−0.15, 0.12]	0.02	[−0.27, 0.31]	0.00	[−0.14, 0.13]	−0.02	[−0.16, 0.12]
*Social‐emot*.	−0.07	[−0.42, 0.23]	−0.29	[−0.61, 0.03]	−0.07	[−0.39, 0.24]	−0.08	[−0.39, 0.23]
*QES*	−0.04	[−0.25, 0.15]	0.13	[−0.29, 0.55]	−0.05	[−0.25, 0.15]	−0.02	[−0.20, 0.15]
*General test*	0.08	[−0.09, 0.25]	0.03	[−0.43, 0.49]	0.06	[−0.11, 0.23]	0.08	[−0.10, 0.25]
*Mean Grade*	−0.03	[−0.06, 0.00]	−0.10	[−0.17, −0.03]	−0.03	[−0.06, −0.01]	−0.03	[−0.06, −0.01]
*Math*	0.06	[−0.12, 0.27]	−0.05	[−0.41, 0.30]	0.07	[−0.12, 0.26]	0.06	[−0.13, 0.25]
*Imp. prob*.	−0.04	[−0.14, 0.08]						
*Share girls*	−0.00	[−0.01, 0.00]						
*Minority*	0.00	[−0.00, 0.00]						
*Duration*	0.00	[−0.01, 0.00]						
*School staff*	0.03	[−0.10, 0.11]						
*Journal*					−0.06	[−0.21, 0.08]		
*Large pop*.							−0.05	[−0.14, 0.04]
*Clustered*							−0.11	[−0.22, −0.01]
*Constant*	0.09	[−0.27, 0.45]	0.51	[−0.04, 1.07]	0.05	[−0.15, 0.25]	0.10	[−0.10, 0.29]
Effect sizes	1030		637		1030		1030	
Clusters	189		142		189		189	
*N*	201,734		120,925		206,186		206,186	
*Q*	412.2		2393.2		430.3		425.2	
*I* ^2^	61.7		95.2		62.4		62.1	
τ ^2^	0.055		0.714		0.054		0.054	

Abbreviations: CAI, computer‐assisted instruction; CI, confidence interval; ES, effect sizes; QES, quasi‐experimental studies.

#### 5.4.1 Effect size measurement

To test sensitivity to how effect sizes were measured and calculated, we included four moderators indicating whether we used the raw means to calculate the effect size, standardised the SMDs with the control group standard deviation (i.e., used a Glass's *δ*), if the standardisation was unclear, or the effect size was standardised with a standard deviation from a super‐population. There were no Glass's *δ*, unclear effect size types, and effect sizes standardised with a super‐population among the follow‐up outcomes and among the single method peer‐assisted instruction effect sizes. In these regressions, we just included the raw means‐indicator. There were no Glass's *δ* and no effect sizes standardised with a super‐population among the single method small‐instruction effect sizes. Thus, this regression included the raw means‐indicator and the indicator for unclear effect sizes.

Including effect size measurement‐moderators in the analyses did not change the estimated average effect sizes much (all increased somewhat). Although the average effect sizes retained their statistical significance in all four analyses, the CIs became broader in all cases. This was expected, as many effect sizes in all analyses was measured or calculated differently and the effect size shown in the figures is the average among those that were not. In particular, we used the raw means to calculate a relatively large proportion of the effect sizes.

The raw means‐indicator was not statistically significant in any analysis, and changed sign between specifications. Effect sizes of unclear type were associated with smaller effect sizes in the three specifications in which we could include it and so were those standardised with a super‐population in the analysis of overall short‐term effects. The only statistically significant moderator was the unclear effect size type in the analysis of small‐group instruction interventions (*β* = −.27, CI = [−0.40, −0.14]).

In the multiple meta‐regression in Table 8, Glass's *δ* and unclear effect size type are relatively large but not significant, whereas raw means and standardising with a super‐population are small and not significant. Peer‐assisted instruction and small‐group instruction retained both their magnitude and statistical significance in the meta‐regression compared with the primary analysis.

#### 5.4.2 Outliers

We examined the distributions of effect sizes for the presence of outliers and the sensitivity of our main results by methods suggested by Lipsey and Wilson (2001): trimming the distribution by dropping the outliers and by winsorizing the outliers to the nearest non‐outlier value. We show the latter results in the figures below. Supporting Information Appendix K: *Extra sensitivity analyses* contains figures showing the effect size distributions of the four types of effect sizes and the results of additional analyses.

Although is difficult to come up with a definition of outliers that is not in some sense arbitrary, there are quite a few effect sizes, particularly in the small‐group instruction category and among the short‐term effects (many of which are the same), which seem like clear outliers. Outliers seem rarer among the follow‐up and peer‐assisted effect sizes, although there a few potential examples. The short‐term and small‐group distributions start to thin out around 1.5 and −0.5, respectively. We used these values as cut‐offs when winsorizing.

The studies with larger and smaller effect sizes than these cut‐offs were almost all small sample studies. All except one effect size was based on a total sample size of 60 or under. Otherwise, they were not exceptional in terms of other potential explanations, for example, study design, risk of bias, or whether we used raw means to calculate effect sizes or not.

Winsorizing outliers had a small impact on our results. All average effect sizes in the subgroup analyses are reasonably close to those in the primary analysis and were still statistically significant (the follow‐up effect size increased to 0.34, which is the largest difference). Peer‐assisted instruction and small‐group instruction were still sizeable and statistically significant in the multiple meta‐regression.

In Supporting Information Appendix K, we report results from analyses where we sequentially remove outliers down to effect sizes in between −0.25 and 1. The results are also again relatively close to those in the primary analysis. Thus, outliers do not seem to be driving our results. It is also worth noting that when we removed outliers, the heterogeneity decreased by quite a lot. For example, with the harshest cut‐off, the *I* ^2^ was 34.4% and the *τ* ^2^ was 0.02 in the meta‐regression including both instructional methods, content domains, and study characteristics.

#### 5.4.3 Clustered assignment of treatment

We tested sensitivity to clustered assignment of treatment by the methods described in Section 4.3.5. In the effect size estimates shown in Figures 10, 11, 12, 13 (named “4. Clustered”), we did not adjust for clustering. We report results in text from specifications in which we instead adjusted effect sizes using a substantially higher ICC (0.3) than in the primary analysis.

As can be seen in Figures 10, 11, 12, 13 and for peer‐assisted instruction and small‐group instruction in Table 8, not adjusting for clustered assignment of treatment left our estimates virtually unchanged. Although adjusting for clustering decreases the individual effect sizes, the average effect sizes decreased slightly when we used unadjusted effect sizes. The reason is likely connected to the fact that the variances of studies with clustered assignment treatment is larger when we adjust for clustering. Therefore, adjusting for clustering gives the adjusted effect sizes smaller weights in the meta‐analyses. As effect sizes from studies using clustered assignment of treatment tend to be smaller in our sample, the average effect size decreased when these studies receive more weight.

A more surprising result was that the CI actually increased a little with unadjusted effect sizes in the analysis of follow‐up effects (the other intervals became slightly broader). The reason may be that the between‐study heterogeneity decreased as well (*τ* ^2^ increased from 0.031 to 0.042). If effect sizes using clustered assignment were further away from the average than effect sizes using individual assignment when not adjusted, they will receive less weight when we adjust. If the ensuing reduction in *τ* ^2^ is large enough, it will dominate the effect of increasing the individual variances by adjusting for clustering.

Increasing the ICC to 0.3 strengthened the above tendencies, although the differences to the primary analysis were again very small. All our main results retained their significance also with this substantially higher ICC (see Supporting Information Appendix K for these results).

#### 5.4.4 Risk of bias

We used the items with numerical ratings from the risk‐of‐bias assessment to examine if the ratings were associated with effect sizes. As described in Section 4.3.11, we coded indicator variables that contrasted effect sizes given a higher risk of bias rating to effect sizes with a lower risk. We defined higher risk as 4 or unclear for the items blinding, incomplete outcome reporting, and other bias, and as ratings higher than 1 for the selective outcome reporting item. We coded all indicators so that they equalled 1 for ratings indicating a higher risk of bias and included them in the multiple meta‐regressions.

As discussed in Section 4.3.11, we omitted the QES from this sensitivity analysis. The coefficient on the constant in the meta‐regressions for overall short‐term effects, overall follow‐up effects, peer‐assisted instruction, and small‐group instruction should therefore be interpreted as the average effect size in RCTs with a relatively low risk of bias. In the analysis of peer‐assisted instruction, we could not include the blinding indicator because there were only two studies rated low risk. Including it together with the other indicators meant that the reference category would have been empty. The constant in the meta‐regressions including both intervention components and study characteristics without missing values is the average effect size in RCTs that did not use any of the included components/characteristics and was at the mean of the mean‐centred variables.

All average effect sizes in Figures 10, 11, 12, 13 increased slightly when we adjusted for the risk of bias ratings compared with the primary analysis. That is, effect sizes with less risk of bias tended to be slightly larger, although the differences were minor. The CIs became broader in all analyses, reflecting that we rated relatively few studies as having a (relatively) low risk of bias on all included items. However, all average effect sizes in RCTs with a low risk of bias were statistically significantly different from zero. Both peer‐assisted instruction and small‐group instruction retained both the magnitude and statistical significance in the multiple meta‐regression. The risk of bias‐item indicators all had small and statistically insignificant associations with effect sizes in this regression.

#### 5.4.5 Moderator**s** with missing values

In the multiple meta‐regressions reported in Table 5, we omitted potentially important moderators because they had missing values. In this section, we report results from specifications using multiple imputation to account for missing values of moderators with relatively low rate of missing values, as described in Section 4.3.6. We imputed values for the following moderators: an indicator for implementation problems, share of girls, share of minority students, duration, and an indicator for whether school staff implemented the intervention (as opposed to e.g., researchers or volunteers). Information about these moderators were missing from <20% of interventions and they are potentially relevant for all types of interventions. Moderators such as the number of sessions and hours per week were also missing from <20% of interventions but are not relevant for all types of interventions (e.g., there are no sessions in incentive and progress monitoring interventions).

The results in column 1 of Table 9 indicate that adjusting for these moderators does not change our main results. The coefficients on peer‐assisted instruction and small‐group instruction are of similar size and both are still statistically significant. Furthermore, all other coefficients are close to the values displayed in column 2, Table 5. The coefficients on the imputed moderators are all small and not statistically significant.

#### 5.4.6 Heterogeneity of contro**l** group progression

This section examines whether there is heterogeneity across the control group conditions by examining the control group's progression from pre‐ to posttest. As in our analysis of heterogeneity of the overall effect sizes, we confined this analysis to the short‐term effects. We calculated a control group “effect size”, as the difference between pre‐ and posttest divided by the control group posttest standard deviation. We used the posttest standard deviation to be able to better compare the progress with the effect sizes found in the primary analysis.

We then (a) tested whether this control group “effect size” was heterogeneous across studies and (b) used multiple meta‐regression to examine whether study characteristics explained some of the heterogeneity. We included 142 studies that supplied the necessary information in the analysis. That is, this sample is different from our primary analysis sample and the results reported should be viewed with some caution in relation to the heterogeneity of control group progression in the primary analysis sample.

We found substantial heterogeneity in the control group progression across studies. We first estimated a specification including just a constant using the same RVE procedure as in the primary analysis (not shown in Table 9). The constant therefore shows the weighted average control group progression, which is 0.62 (CI = [0.50, 0.74]). There is substantial variation around this average: the *τ* ^2^ is 0.70, the *I* ^2^ is 96%, and the *Q‐*statistic is 3949, which is highly significant. There continues to be heterogeneity when we confine the set of interventions to those using peer‐assisted instruction (mean = 0.47, *τ* ^2^ = 0.07, *I* ^2^ = 55.4, and *Q* = 31.4) or small‐group instruction (mean = 0.61, *τ* ^2^ = 0.28, *I* ^2^ **= 88.2, and *Q* = 534.3) as their only instructional method.

The results in column 2 of Table 9, which mimics the specification shown in Table 5, column 2, shows that very few of the instructional methods and content domains were significantly associated with the control group progression. The only two significant associations are with number sense and mean Grade. Number sense is positively associated and mean Grade negatively associated with effect sizes. These results are not surprising, given that students on average make less progress, the higher the Grade (Lipsey et al., 2012), and that number sense is typically a focus in the early years of primary school.

Thus, the results indicated that control group progression was strongly heterogeneous, which may imply that the quality of control group instruction is an important explanation of the heterogeneity we see in most of our analyses. However, it was reassuring that we did not find strong evidence of an association between the control group progression and intervention components, and in particular that we did not find a significant association with peer‐assisted instruction and small‐group instruction.

#### 5.4.7 Publication bias

This section examines publication bias by testing whether unpublished studies have different effect sizes compared with published studies, and by using funnel plots and Egger's test (Egger et al., 1997). However, we want to acknowledge that these tests are for several reasons difficult to interpret as direct evidence of publication bias, or the lack thereof. The effect size estimates and corresponding standard errors we have analysed are not necessarily the ones used by authors, editors, and reviewers to decide whether a paper should be published. We used only effect sizes from standardised tests that had low enough risk of bias, which were included in studies found through our search and screening process. There may thus be publication bias in the literature that would not show up in our sample and other processes than publication bias can cause asymmetries in funnel plots. We discuss the interpretation of funnel plots further below.

We found no evidence that effect sizes from studies published in scientific journals were larger than effect sizes from studies not published in journals (e.g., in government reports, working papers, and dissertations). We added an indicator equal to 1 if the study was published in a journal to the specification in column 2 of Table 5 for the full sample of short‐term effects. The estimate, reported in column 3 of Table 9, indicated that studies published in journals, conditional on all other moderators without missing values, have slightly lower effect sizes (*β* = −.06), but not significantly so.

For the funnel plots and test of asymmetry, we averaged effect sizes and variances over studies and estimated a random‐effects model by using the REML option with the Knapp and Hartung adjustment of standard errors in the R package *metafor* (Viechtbauer, 2010). As mentioned, this procedure also provided a sensitivity test of our primary analysis.

Table 10 shows the average effect sizes, CIs, and heterogeneity statistics for the REML procedure compared with the RVE procedure used in the primary analysis. The effect sizes and CIs from the two procedures are very close in all four cases. The heterogeneity statistics indicate more heterogeneity in the RVE procedure, which seems reasonable as the RVE procedure takes into account variation in effect sizes within studies. However, our conclusions would be similar using study‐level averages. There is substantial heterogeneity across short‐term and small‐group instruction effect sizes. The heterogeneity across follow‐up effect sizes is smaller, but still statistically significant, whereas the heterogeneity among the effect sizes from peer‐assisted instruction intervention is relatively small and not significant.

**Table 10:** Comparing the primary analysis with the study‐level analysis

	Primary analysis					Study‐level effect sizes				
	(1)	(2)	(3)	(4)	(5)	(6)	(7)	(8)	(9)	(10)
Analysis	ES	95% CI	*Q*	*τ* ^2^	*I* ^2^	ES	95% CI	*Q*	*τ* ^2^	*I* ^2^
Short‐term	0.30	[0.25, 0.34]	797.3	0.067	76.4	0.29	[0.24, 0.34]	617.6	0.053	72.1
Follow‐up	0.27	[0.17, 0.36]	47.8	0.031	45.6	0.25	[0.16, 0.34]	35.8	0.004	8.6
Peer‐assisted	0.39	[0.26, 0.52]	18.7	0.023	19.8	0.38	[0.24, 0.52]	11.5	0.000	0.00
Small‐group	0.38	[0.30, 0.45]	285.4	0.084	70.6	0.37	[0.30, 0.44]	205.9	0.051	59.3

*Note*: The short‐term and follow‐up results were reported in Section 5.3.1 of the main text, and the peer‐assisted and small‐group instruction results in Table 4. Columns 6–10 report estimates using study‐level average effect sizes and the REML option with the Knapp and Hartung adjustment of standard errors in the R package *metafor* to estimate weighted average effect sizes, confidence intervals (in brackets), and heterogeneity.

Abbreviations: CI, confidence interval; ES, effect sizes.

Figure 13 displays funnel plots of the study‐level short‐term effect sizes (upper left corner), follow‐up effect sizes (upper right corner), peer‐assisted instruction effect sizes (lower left corner), and small‐group instruction effect sizes (lower right corner). The effect sizes are shown on the *x*‐axis and standard errors on the *y*‐axis. The center‐line displays the weighted average in each analysis (i.e., the effect size from column 6 in Table 10).

There are indications of asymmetries primarily for short‐term effects and small‐group instruction. There seem to be more outliers with positive effects and more large than small studies with effect sizes around null. Although they seem less asymmetric, the drastically smaller number of studies in the follow‐up and peer‐assisted categories makes these plots harder to interpret. Conducting Egger's test (Egger et al., 1997; we used the *regtest* option in *metafor*), we rejected the null hypothesis of no asymmetry for the short‐term effect sizes (*p* < .001), and small‐group instruction effect sizes (*p* = .002) but not for follow‐up effect sizes (*p* = .165) and peer‐assisted instruction (*p* = .518). We got qualitatively similar results when we used the “Egger sandwich” test suggested by Rodgers and Pustejovsky (2020): we again rejected the null hypothesis for the short‐term effect sizes (*p* < .001), and small‐group instruction effect sizes (*p* = .007) but not for follow‐up effect sizes (*p* = .107) and peer‐assisted instruction (*p* = .606).

**Figure 14:**
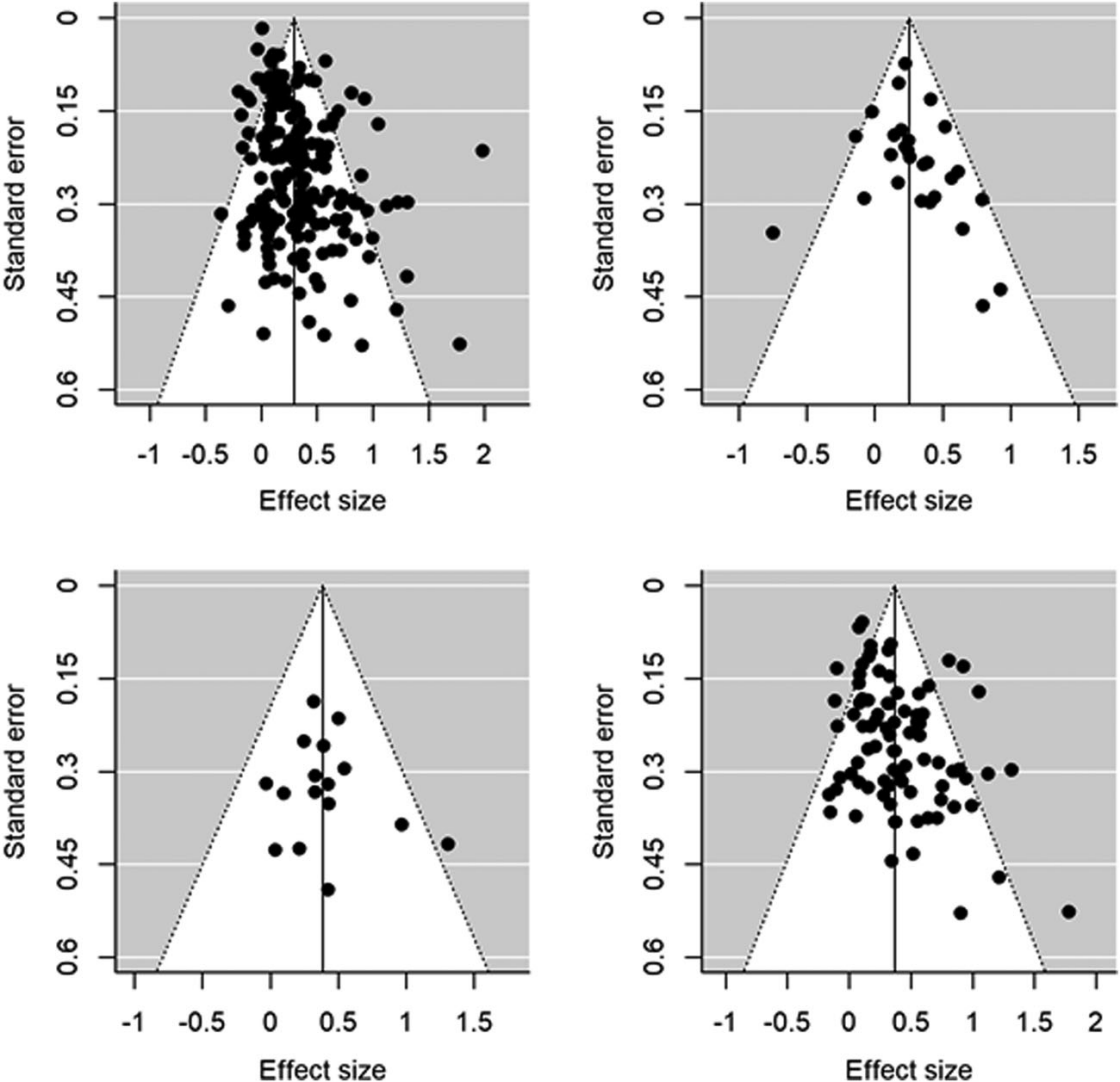
Funnel plots of study‐level short‐term effect sizes (upper left corner), follow‐up effect sizes (upper right corner), peer‐assisted instruction effect sizes (lower left corner), and small‐group instruction effect sizes (lower right corner)

Asymmetric funnel plots may have other causes than publication bias. In general, asymmetry is a sign of small‐study effects, of which there can be many causes beside publication bias (Sterne et al., 2005). Small‐study effects would show up as heterogeneity (Egger et al., 1997). In line with this idea, the analyses displaying more heterogeneity across effect sizes in Table 10 and that have more studies outside the funnel lines in Figure 14 also have lower *p* values in Egger's test. A general reason to expect small‐study effects is that researchers often perform statistical power analyses before embarking on an intervention. As interventions with large expected effects require smaller sample sizes for a given level of power, small studies will have larger effects if researchers are reasonably good at guessing the effect sizes (Hedges & Vevea, 2005).

In our context, there are several further reasons why studies with larger sample sizes and consequently smaller standard errors could have smaller effect sizes. One reason is that larger samples tend to be more heterogeneous and may therefore have larger standard deviations (e.g., because they include school district variation and not just school variation as argued by Lipsey et al., 2012). Larger standard deviations would mean smaller standardised mean differences in large studies, even if the effects were exactly the same.

A second reason is that it may be easier to get large positive effects in studies with small samples. Students and teachers can be given more attention and be better monitored by researchers in small studies and there is less risk of coordination and implementation problems. More attention and better monitoring seem likely to increase effect sizes (see Thomas et al., 2018, for an interesting discussion and results in line with this hypothesis) and coordination and implementation problems may cause both smaller effect estimates of otherwise effective programmes and decrease the chances of publication, regardless of publication bias.

A third reason has to do with the recruitment of schools and teachers to studies. In small‐scale studies, researchers typically recruit schools or teachers directly, which implies that only schools or teachers that want to participate are included in the sample. In large‐scale studies, larger administrative units like school districts or municipalities are more likely to be the unit of recruitment. In turn, these larger units may determine which schools and teachers that participate, meaning that some schools and teachers that do not want to participate are included in the sample. If being motivated to participate is important for how well schools and teachers implement the intervention, which seems reasonable (e.g., Kennedy, 2016), then, regardless of publication bias, we should expect smaller effects in large‐scale studies. More generally, it may be easier for small‐scale interventions to select sites that are particularly likely to benefit whereas large‐scale studies is more informative about the population‐wide effects. Depending on the type of policy‐maker (or researcher), both types of effects are interesting, but they are not likely to be the same.

Such connections between sample sizes and effect sizes would violate the assumptions needed to interpret asymmetric funnel plots and Egger's test as publication bias (it would also violate the assumptions underlying the selection models suggested by, e.g., Hedges, 1992 and Andrews & Kasy, 2019). Furthermore, if sample sizes are systematically different across intervention components, the analyses we presented in this review may risk confounding the associations of intervention components with sample sizes.

We examined this issue further by including two study‐level moderators related to some of the reasons stated above in our analyses. The first moderator is equal to one if the effect size/study included a larger than the median number of schools (median = 8), districts (1), or regions (1). If the study contained no information, it was coded as zero along with studies below the median. About 57% of the effect sizes and 52% of studies had a larger number of units regarding at least one of these three units. The second moderator is an indicator for clustered assignment of treatment. As mentioned, 61 studies (around 31%) used a clustered assignment of treatment, which amounted to 17% of the effect sizes (i.e., clustered studies included on average fewer standardised tests/effect sizes). Both of these indicators typically means that the sample size is larger and may imply more coordination problems, as well as a different recruitment process (unfortunately, we did not code how participants were recruited or whether a power analysis was conducted).

We first added the two moderators to the specification reported in column 2, Table 5. As can be seen in Table 9, both indicators were negatively associated with effect sizes but only the indicator for clustered assignment was statistically significant. Reassuringly, none of our other results changed much compared with our primary analysis. We then proceeded to add the same two moderators in the specification underlying Egger's test in the two analyses where this test indicated funnel plot asymmetry. Although the moderators continued to be negatively associated with effect sizes, we still found a significant asymmetry in the funnel plots of overall short‐term effect sizes (*p* < .001) and small‐group instruction effect sizes (*p* = .004). As the heterogeneity may depend on outliers, we also ran the test using winsorized effect sizes (with cut‐offs at −0.5 and 1.5). This did not change the outcome of the test, which was still significant for both short‐term effect sizes (*p* < .001) and small‐group instruction (*p* = .010).

Of course, these results do not prove that there is publication bias, the moderators are imperfect indicators of the phenomena we want to capture and there may, as discussed, be other reasons for the funnel plot asymmetry.

#### 5.4.8 Summary of sensitivity analysis

The sensitivity analysis showed that the positive and statistically significant overall average short‐term and follow‐up effect sizes, and the positive and statistically significant associations between peer‐assisted instruction and small‐group instruction were generally robust. The exceptions were that there were too few peer‐assisted instruction effect sizes with a relatively low risk of bias on the blinding item for us to include this item in the analysis, and that there were indications of asymmetric funnel plots in the analyses of short‐term and small‐group instruction effect sizes. While we therefore should be more cautious in the interpretation of our results, these results should not be interpreted as a confirmation that there is publication bias. There are other reasons for asymmetric funnel plots, which seem likely to apply in our case.

## 6 DISCUSSION

### 6.1 Summary of main results

Our main objective was to assess the effectiveness of targeted interventions for students with or at risk of academic difficulties from kindergarten to Grade 6, as measured by standardised tests in reading and mathematics. We found in total 607 studies that met our inclusion criteria and included 205 of these studies in meta‐analyses: 202 included intervention‐control contrasts and 3 that contained only comparison designs (intervention‐control and comparison design contrasts were never included in the same meta‐analysis). The reasons for not including studies in the meta‐analyses were that they had too high risk of bias (257), that they compared two alternative (and non‐recurring) interventions instead of an intervention and a control group (104 studies), that we were unable to retrieve enough information to calculate an effect size (24 studies), or that the studies used samples that overlapped with other included studies, and had either a higher risk of bias or contained less information (17 studies). Furthermore, 6 studies reported estimates from the same interventions in separate reports/articles, and we treated them as one study cluster in the analysis. Of the 195 study clusters, 327 interventions, and 1334 effect sizes that we included in a meta‐analysis of intervention‐control effect sizes, 93% of interventions were RCTs.

The weighted average short‐term effect size was positive and statistically significant (ES = 0.30, CI = [0.25, 0.34], estimated using 1030 effect sizes and 189 study clusters). The weighted average follow‐up effect size, measured more than 3 months after the end of intervention, was positive and significant (ES = 0.27, CI = [0.17, 0.36], estimated using 195 effect sizes from 27 study clusters). Interventions for students with or at risk of academic difficulties are therefore, on average, effective, and the effects do not disappear immediately after the end of intervention.

All measures of heterogeneity indicated substantial variation among short‐term effect sizes. Follow‐up effect sizes displayed some heterogeneity, but to a much smaller degree. For example, the short‐term *τ* ^2^ was 0.067 and the *I* ^2^ was 76.4, and the corresponding statistics were 0.031 and 45.6 for the follow‐up effect sizes. A very large share of follow‐up effect sizes examined small‐group instruction and tested effects on reading measures. We therefore focused the subgroup analysis and investigation of heterogeneity on the short‐term effects.

The subgroup and heterogeneity analyses focused on instructional methods and content domains. We examined average effect sizes in single‐factor subgroup analyses of interventions that included a certain method or domain, and single method and single domain interventions, and recurring combinations of methods, and we used multiple meta‐regressions to examine models that included indicators for methods and domains as well as additional study characteristics.

The main results were that peer‐assisted instruction and small‐group instruction (groups of five students or less instructed by an adult) had large, stable, and statistically significant average effect sizes across specifications (around 0.35–0.45). Both peer‐assisted instruction and small‐group instruction had large effects in studies of interventions where they were the only instructional method used. They furthermore had significantly larger effect sizes than CAI, coaching of personnel, incentives, and progress monitoring in a meta‐regression that included indicators for all coded instructional methods, content domains, and additional study characteristics. Peer‐assisted instruction also had significantly larger effect sizes than medium‐group instruction (groups of six or more students), whereas small‐group instruction did not (*p* = .07, however). We summarise the results for all components more in detail below, when we compare our results to other reviews. Although all other instructional methods had positive average effect sizes when we analysed interventions including them (and possibly other methods), none was consistently significant across the analyses that tried to isolate the association between a specific method and effect sizes.

The evidence for the average effectiveness of peer‐assisted instruction and small‐group instruction was thus strong, whereas most other methods were examined in only a few single method interventions (CAI and medium‐group instruction were the exceptions with 12 and 10 studies, respectively). However, with the exception of CAI and peer‐assisted instruction in single method interventions, there was still significant heterogeneity within the categories of instructional methods (including substantial heterogeneity in single method small‐group instruction interventions).

We defined peer‐assisted instruction and small‐group instruction broadly. We tried to examine narrower categories, but found only weak evidence of differences. The effect sizes were similar on math and reading tests. Most interventions in the peer‐assisted category examined same‐age peer‐tutoring (or cooperative learning) in pairs. Few examined cross‐age peer‐tutoring or larger groups. The heterogeneity was not significant in single method interventions.

Almost all small‐group instruction interventions targeted academic subjects rather than for example social‐emotional or general academic skills. That is, they were closer to tutoring than mentoring. We found no large or statistically significant differences between instruction one‐to‐one and in groups of two to three, or four to five students. The heterogeneity of the small‐group instruction remained substantial also in these analyses. Using studies that directly compared one‐to‐one instruction with instruction groups of two to five students, we found no significant differences but our statistical power to detect differences was low.

Effect sizes based on math tests was larger than those based on reading tests but the difference was small (around 0.05) and not significant. We found furthermore little evidence that effect sizes were larger in some content domains than others. The exception was interventions targeting fractions, which had significantly higher associations with effect sizes than all other math domains. However, we found only six studies of interventions targeting fractions, and only one of them targeted only fractions. This finding reflected a more general pattern: most interventions targeted more than one content domain, which made it more difficult to isolate the associations between effect sizes and content domains than between effect sizes and instructional methods.

The multiple meta‐regressions revealed few other significant moderators. The mean Grade of the intervention was negatively associated with effect sizes, implying that interventions in higher Grades tend to have somewhat lower effect sizes (a reduction with around 0.03 per Grade). The results for the other study characteristics indicated that effect sizes from QES were not substantially or significantly different from RCTs (we excluded a large share of QES because we assessed them to have too high risk of bias). Furthermore, general tests did not have significantly different effect sizes from tests of subdomains and effect sizes based on math tests were not significantly different from those based on reading tests. In a sensitivity analysis, we used multiple imputation to include variables measuring implementation problems, share of girls, share of minority students, duration, and whether school staff implemented the intervention. None of them was significantly associated with effect sizes.

### 6.2 Overall completeness and applicability of evidence

We conducted a comprehensive search of electronic databases and national indexes/repositories and trial/review archives, combined with grey literature searching, hand searching of key journals, and extensive citation tracking. In addition, we consulted experts in the field. We found a large number of records, which was screened and coded independently by at least two review team members. We searched for studies back to 1980, but included few studies conducted before 2000 and very few conducted before 1990. Therefore, we do not believe that the period limit made us miss a substantial number of relevant studies. We were however unable to retrieve all potentially relevant records in full text (*k* = 201). For reasons we discuss in the next section, we believe that they are unlikely to have biased our results.

In line with the comprehensive search, we included many forms of publications: journal articles, working papers, conference papers, dissertations, and government reports. However, there may still be recent unpublished studies that we did not find, as educational researchers do not have a tradition of publishing working papers. Publication bias may be another source of missing unpublished studies. We discuss this issue further in the next section. We were also unable to include 24 studies that met our inclusion criteria in the meta‐analysis because they lacked information necessary to calculate an effect size, and we were unable retrieve the missing information from the authors. These studies were more often than included studies not published in scientific journals: around half were either reports or dissertations whereas 82% of the studies in the meta‐analysis were published in a scientific journal. The studies with missing information were older than the average included study: around a third were published before 2000 compared with 14% in the meta‐analysis. The country distribution was similar (21 out of 24 were from the United States) and we have otherwise no reasons to expect them to have a different impact than our included studies.

A large share of the studies included in the meta‐analysis were of interventions conducted in the United States. Several other countries were represented among the included studies—Australia, Canada, Denmark, Germany, Ireland, Israel, Netherlands, New Zealand, Sweden, and the United Kingdom—but there were few studies from each of these countries. In a strict sense, our results therefore mainly apply to the United States school context. However, we believe our main results are transportable to other school contexts outside the United States. There are examples of successful targeted interventions in most countries. Instructional methods like small‐group and peer‐assisted instruction are not particularly dependent on the type of school system in place and would in principle be possible to implement in almost any school.

### 6.3 Quality of the evidence

We excluded many effect sizes from the meta‐analyses because our assessment resulted in a rating of too high risk of bias. That is, our assessment was that they were more likely to mislead than inform the analysis. The most common reasons for this assessment were: inadequate (often no) adjustment for confounding factors, confounding of intervention effects with for example school, teacher, class or cohort effects, and non‐comparable intervention and control groups.

A large majority (84%) of the effect sizes with too high risk of bias were from QES. Reasons for excluding effect sizes from RCTs were for example that the randomisation was compromised and there was inadequate control for confounding, because only one unit was assigned to the intervention or control group, because the studies reported results for a subset of included tests or students, or because of large‐scale attrition. QES were not associated with higher effect sizes than RCTs in the primary analysis, the QES made up a small share of the included interventions (7%), and our results were similar when we only included RCTs in the analysis. We therefore do not believe that the inclusion of QES biased our results.

The effect sizes included in the meta‐analysis had a risk of bias that ranged from low to high, with most effect sizes having a moderate to high risk on at least some items. We conducted a sensitivity analysis in which we adjusted for ratings on the risk of bias items. There were no significant associations between risk of bias ratings and effect sizes and our main results were unaffected by the inclusion of moderators based on the risk of bias items. However, the low number of effect sizes from peer‐assisted instruction interventions rated with relatively low risk on the blinding item made it impossible for us to include this item in the sensitivity analysis.

Despite the lack of associations with effect sizes, it is worth discussing in some more detail what caused the high risk of bias ratings and how the risk of bias potentially can be decreased in future studies. Information about how the random sequence was generated was lacking in most RCTs, and the description of the randomisation procedure was often sparse. As such information is easy to include, this is an area where the reporting of studies can be improved.

Blinding was a problem in all included studies. Complete blinding is difficult to achieve in educational research, but it is for example often possible to use testers that are blind to treatment status. Lack of blinding could both bias the results in favour of the intervention group and in favour of the control group (Glennerster & Takavarasha, 2013). For example, if knowledge about treatment status and that they are participating in an experiment make students try harder—that is, a Hawthorne effect—then the beneficial effects are overstated. However, control group students (or their parents or teachers) may seek out help elsewhere or try harder because they know they did not get the intervention or because they want to compete with the intervention group (i.e., a John Henry effect). In that case, the beneficial effects are understated. We were unable examine this issue with the material at hand, and we are not aware of any other study that have examined this issue in educational interventions.

Around 25% of effect sizes had a low risk of bias (rating 1) in terms of incomplete outcomes. If attrition by comparatively low‐achieving students in the intervention group is more common, then the effects in our meta‐analysis would be overestimated. However, it seems plausible that successful interventions may also make low‐achieving treated students stay in school or show up at testing occasions at a higher rate than the control group. Such a pattern would instead imply that the effects were underestimated. Incomplete outcome data is of course difficult to avoid completely. Nevertheless, some studies could do more to mitigate these problems by examining and testing whether there is differential attrition between intervention and control groups, and adjust for such attrition if present. The data needed to perform such tests and adjustments are usually available to study authors.

Skewed data increase the risk of bias when analysing continuous outcomes, particularly in small sample studies. Consequently, such data may bias our meta‐analysis. We found little evidence of problems with skewed data in our risk of bias assessment, although one reason may be that relatively few studies provided information about more moments of the distribution of outcome variables than means and variances. Another problematic feature of the included studies is the near universal lack of pre‐published protocols and analysis plans. This made it difficult for us to assess whether there was selective reporting or not, but, more importantly, pre‐publishing trial protocols and analysis plans could also mitigate researcher bias and promote transparency.

Besides including the risk of bias items as moderators, we tested the sensitivity of our results to how effect sizes were measured, to outliers, by adjusting for the clustered assignment of treatment, and by including moderators with missing observations. Our main results were robust across these sensitivity analyses.

We also tested if there was heterogeneity across the control group's progression from pre‐ to posttest. One interpretation of such heterogeneity is that the quality of control group instruction differs across interventions, which in turn would be a source of bias. As we were unable to develop moderators based on the control group instruction, such heterogeneity may also explain heterogeneity across effect sizes. While we found strong indications of heterogeneity, there were few significant associations between intervention components and the control group progression.

We found some indications of asymmetric funnel plots for the studies included in the analyses of short‐term and small‐group instruction effect sizes. We performed a thorough search for studies not published in scientific journals and we found only small and not significant differences between effect sizes from studies published in journals and from studies published elsewhere. A possible interpretation of these results is that the missing effect sizes are mainly a file‐drawer problem (Rosenthal, 1979). This interpretation is consistent with the evidence presented in Franco et al. (2014), which indicates that the file‐drawer problem is the main culprit behind publication bias in the social sciences.

There are many other possible explanations for the asymmetric funnel plots, which do not involve publication bias. We believe at least four reasons why small studies tend to show larger effects may be pertinent in our case: First, statistical power analyses may generate a connection between sample size and effect sizes. Second, larger samples tend to be more heterogeneous, and may therefore have larger standard deviations and smaller standardised mean differences. Third, small samples makes monitoring, implementation, and coordination easier. Fourth, larger studies may be more likely to recruit schools and teachers that do not volunteer to participate.

### 6.4 Potential biases in the review process

We performed a comprehensive search and all records were screened and coded by at least two independent screeners in order to minimise the risk of bias in the review process. Three features of the process are however worth discussing in some more detail as they may be a cause for concern.

First, the review team has included many people during the screening and coding phases, which increases the risk of inconsistencies. All team members were thoroughly introduced to the review methods used, and extensive pilot screening and coding were undertaken in each case. All uncertain cases during both first and second level screening were assessed by at least one review author, in addition to two review team assistants. The number of people involved in the coding and assessment of studies was smaller, which should increase the level of consistency. For example, at least one of the first two authors assessed risk of bias for all studies.

Second, we were unable to retrieve 201 records in full text, which amounts to 5% of the total number of records screened in full text, and 0.8% of the total number of records. A minority share of these records likely pertains to another review about students in Grades 7–12, which shared the search and screening process with this review but included less studies (247 compared with 607; see Dietrichson et al., 2020). Furthermore, the records were such that we could not exclude them on being obviously irrelevant in the first level screening, not records that necessarily were relevant. Around 5% of the studies screened in full text were included in any meta‐analysis in this review. Furthermore, older reports from the 1980s and dissertations were overrepresented among the potentially missing studies, which were types of studies that less often met our inclusion criteria. Due to these features, we believe that very few of the 201 not retrievable studies would have been included in our analyses, and that the risk that our results are biased because of these missing records is low.

Third, most of our included interventions were implemented in English‐speaking countries, and 86% were from the United States. Although our search was not limited to records written in English and we did find studies in other languages, we had to restrict the included studies to languages that the review team understood (Danish, English, German, Norwegian, and Swedish). As a result, we may have missed studies from countries where none of these languages are used. Another reason for the dominance of studies from English‐speaking countries could be the, at least historically, stronger focus on qualitative methods in educational research in some European countries (e.g., Pontoppidan et al., 2018).

### 6.5 Agreements and disagreements with other studies or reviews

All reviews including students with or at risk of academic difficulties in kindergarten to Grade 6 that we are aware of have found positive average effect sizes. Furthermore, most reviews that compared different intervention types found substantial heterogeneity of effect sizes. In that sense, our overall short‐term results are in agreement with other reviews.

We are not aware of another review that have provided meta‐analytic estimates of medium‐ or long‐term effects of interventions targeting students with or at risk of academic difficulties. Suggate (2016) reviewed the long‐run effects of phonemic awareness, phonics, fluency, and reading comprehension interventions from preschool up to Grade 7 and included both at‐risk and not‐at‐risk students. Suggate reported positive effect sizes in general, but they were on average reduced by 40% at follow‐up compared with posttest. The mean duration between posttest and follow‐up in Suggate's review was around 11 months. This result is relatively close to our result that effect sizes measured between 12 and 24 months after the end of intervention was 0.17, whereas the short‐term effect sizes in studies that provided a follow‐up measurement was 0.40 (i.e., the follow‐up effects were reduced by 58%).

Below, we comment by intervention component on the most closely related reviews that have used meta‐analysis to examine effect sizes based on standardised or non‐researcher‐developed tests in reading and mathematics for similar at‐risk groups and intervention types in a similar group of countries. Some of the definitions of intervention types used in these reviews were however not comparable to ours, and we only comment on those parts that we deemed were comparable. There are also differences across the reviews in how outcomes were measured and how effect sizes were calculated. We provide the most comparable average effect sizes from the reviews below, but the reader should be aware that they may still not be fully comparable with our effect sizes. Furthermore, with the exception of Gersten et al. (2009), none of the reviews mentioned below used multiple meta‐regressions to examine the association of individual intervention components with effect sizes while adjusting for instructional methods, content domains, and moderators based on study characteristics. Most reviews did not explicitly examine single instructional method or single content domains and their results are therefore closest to the results we reported in Table 3.

#### 6.5.1 CAI

We found significant average effect sizes of CAI across interventions that included this component (ES = 0.15) and in single method interventions (ES = 0.13), but smaller and not significant associations in meta‐regressions where we adjusted for other components and study characteristics. Dietrichson et al. (2017) found a combined average effect size in reading and mathematics of 0.11 for CAI interventions targeting students with low SES. Slavin et al. (2011) and Inns et al. (2019) reviewed interventions for struggling readers and similarly defined instructional technology interventions or programmes. They found average effect sizes of 0.09 and 0.05, respectively. Slavin and Lake (2008) found an overall effect size of 0.19 for CAI interventions targeting mathematics and general student populations, but stated that effects were similar for disadvantaged students and students with a non‐majority ethnic background. Our results for CAI are thus reasonably close to those from comparable analyses in earlier reviews.

#### 6.5.2 Coaching of personnel

The average effect size of coaching of personnel was around 0.20 and statistically significant in interventions that included this instructional method. We found no evidence that coaching was associated with substantial or significant effects in meta‐regressions or in single method interventions. Combining math and reading outcomes, Dietrichson et al. (2017) found an average effect size of 0.16 for coaching interventions targeting students with low SES. They did not examine single method interventions or included coaching in meta‐regressions. Our results for coaching of personnel are thus close to the comparable analysis in their review (Kraft et al., 2018, also reviewed coaching interventions but did not report results for at‐risk groups).

#### 6.5.3 Incentives

We found a relatively large average effect size in interventions including an incentive component (ES = 0.33). The average effect size was smaller and not significant in single method interventions (ES = 0.05) and in the meta‐regressions. Dietrichson et al. (2017) found an average effect size of 0.01 for incentive interventions targeting students with low SES. Although their coding and analysis methods were most comparable to the one we used to obtain the effect size of 0.33, our result seemed to be driven by the inclusion of interventions that combined incentives with other methods, in particular small‐group instruction. Most of these studies were not included in Dietrichson et al. (2017). Our single method and meta‐regression results are close to their results.

#### 6.5.4 Medium‐group instruction

Instruction in medium‐sized groups had a relatively large and significant average effect size in interventions including such a component (ES = 0.32), but smaller and not significant in single method interventions (ES = 0.10) and in the meta‐regressions. Dietrichson et al. (2017) found an average effect size of 0.24 for a similarly defined category (called small‐group instruction) in their review of interventions targeting students with low SES. As the methods used to derive their result was closest to our ES = 0.32, the results are reasonably close.

#### 6.5.5 Peer‐assisted instruction

Peer‐assisted instruction had a relatively large effect size averaged over interventions that included this component (ES = 0.44), in single method interventions (ES = 0.39), and retained significance and a similar size in the meta‐regressions. Dietrichson et al. (2017) found an average effect size of 0.22 for cooperative learning interventions targeting students with low SES. Slavin et al. (2011) found a large average effect size of cooperative learning interventions for struggling readers (ES = 0.58). Inns et al. (2019) found an average effect size of 0.29 for “classroom approaches”, where four out of five studies were of cooperative learning/same‐age peer‐tutoring programmes. Gersten et al. (2009) found very large effects for cross‐age tutoring (ES = 1.02) in their review of math interventions for school‐age learning disabled students, but they included only two such studies. The effects for same‐age (within‐class) peer‐assisted instruction was lower, 0.14, and decreased further when they adjusted for other methods and study characteristics in a meta‐regression.

The target group in Gersten et al. likely had more severe academic difficulties than the average student in our review (as well as the target group in Slavin et al., 2011, and Inns et al., 2019). One explanation of the differences across reviews is therefore that peer‐assisted instruction may work less well for students with the greatest difficulties. Another possible explanation may be that Gersten et al. also included effect sizes based on non‐standardised tests, but they adjusted for this in the meta‐regression and non‐standardised tests typically yield larger effects. Gersten et al. also included interventions targeting older students (although most of their included studies included participants in our Grade range). Note that Gersten et al. only included math interventions, but our results were similar for peer‐assisted instruction across reading and math tests.

#### 6.5.6 Progress monitoring

Interventions including progress monitoring had a significant average effect size of 0.17, whereas there were only two progress monitoring interventions where this method was the only one (ES = 0.28). The associations with effect sizes was small and insignificant in our meta‐regressions. Dietrichson et al. (2017) found an average effect size of 0.32 for progress monitoring interventions targeting students with low SES, but they included only four studies of such interventions and this method was in all cases combined with other methods. Gersten et al. (2009) examined math interventions for students with learning disabilities. Their category teacher feedback combined (ES = 0.23) was reasonably close to how we defined progress monitoring. As in our case, when Gersten et al. included other moderators in a meta‐regression the teacher feedback components (Gersten et al. split the component in two in 7their meta‐regression) lost their statistical significance.

#### 6.5.7 Small‐group instruction

Small‐group instruction showed consistently significant and relatively large effect sizes in analyses of interventions including this component (ES = 0.38), in single method interventions (ES = 0.38), and retained both size and significance in meta‐regressions. Dietrichson et al. (2017) found an average effect size of 0.36 for tutoring interventions targeting students with low SES in an analysis similar to our Table 3. Inns et al. (2019) found an average effect size for “one‐to‐small group” tutoring of 0.20 and 0.25 for one‐to‐one tutoring. Slavin et al. (2011) found an average effect size for one‐to‐one tutoring by teachers of 0.39, by paraprofessionals of 0.38, and volunteers of 0.16, and an average effect size for small‐group tutoring of 0.31 (both Inns et al., 2019, and Slavin et al., 2011, reviewed programmes/interventions for struggling readers in Grades K‐5/6). Wanzek et al. (2016) found larger average effect sizes of one‐to‐one (ES = 0.50), one‐to‐two or three (ES = 0.61), and one‐to‐four or five (ES = 0.44) student small‐group instruction, but they included only interventions in Grades K‐3 in their review of students with or at risk of reading difficulties. Wanzek et al. (2018) found average effect sizes of 0.59 for one‐to‐one tutoring and 0.33 for small‐group tutoring in the review of intensive (100 sessions or more) reading interventions for at‐risk students in Grade K‐3. The results of our review was thus in between the effect sizes reported in earlier reviews, which may be explained by our broader inclusion criteria and larger number of studies.

Other reviews with a target group consisting of more general student populations have also analysed small‐group instruction and tutoring. As tutoring nearly always is targeting students with or at risk of difficulties, we discuss them as well. Fryer (2017) reported an average effect size of 0.31 for math achievement and 0.23 for reading for “high‐dosage tutoring”, which was quite close to our definition. Pellegrini et al. (2018) found an average effect size of 0.25 for combined one‐to‐one and small‐group tutoring in math. Nickow, Oreopoulos, and Quan (2020) examined RCTs in pre‐K to Grade 12 of tutoring, defined as one‐on‐one or small‐group instructional programming by teachers, paraprofessionals, volunteers, or parents, and found an overall pooled effect size of 0.37. Ritter et al. (2006) reviewed the effectiveness of volunteer tutoring programmes for improving the academic skills of student enroled in Grades K‐8 (in the United States), and found effect sizes of 0.30 for reading outcomes and 0.27 in mathematics. As our small‐group instruction category contained almost only tutoring interventions, these results agreed reasonably well with ours.

#### 6.5.8 Content domains

We found few differences between math and reading effect sizes, and between effect sizes from interventions targeting more narrowly defined content domains. Interventions targeting the reading domains comprehension, decoding, fluency, spelling and writing, and vocabulary had average effect sizes ranging from 0.20 to 0.32. We examined the following mathematics domains: algebra/pre‐algebra, fractions, geometry, number sense, operations, and problem solving, which had average effect sizes ranging from 0.15 to 0.50. Only comprehension (ES = 0.21), decoding (ES = 0.31), number sense (ES = 0.51), and operations (ES = 0.17) had been examined in more than one single domain intervention. The meta‐regressions revealed few significant differences across content domains. The exception was fractions, which had a significantly stronger association with effect sizes compared with the other math domains. However, given the small number of interventions targeting fractions and that just one of them targeted only fractions, we caution against strong conclusions based on this result.

Few reviews have examined effect sizes by the domains targeted by interventions for our target group. Gersten et al. (2009) examined the following math domains: operations, word problems, fractions, algebra, and general math proficiency. The categories with similar names as ours also had similar definitions. Their word problems category was similar to our problem‐solving category and their general math proficiency similar to our multiple math category. They found larger effect sizes for word problem interventions in a meta‐regression, but the differences to other domains were not significantly different.

Regarding reading domains, Wanzek et al. (2016) examined effects of interventions on standardised measures of language and comprehension, in Grade K‐3. They coded studies by the outcomes, not the by the targeted domain. However, it seems likely that interventions target the areas that are tested, so their coding may be reasonably close to ours. They found an average effect size of 0.38 for language and comprehension outcomes among interventions in Grade K‐3. While this was slightly larger than our effect size for comprehension, students in their review were in lower Grades and we found larger effects for the lower Grades.

Scammaca et al. (2015) included no study using standardised tests in 4–5th Grade, which was their analytical category closest to our Grade range. The overall average effects in Scammaca et al. (2015) measured by standardised tests were 0.25 in Grade 6–8, which was close to our overall effect size on reading tests. Their effect sizes reported by reading domain were larger than ours for reading comprehension (ES =0 .47) and word study (ES = 0.68), and reasonably similar for fluency (ES = 0.17) and multiple components (ES = 0.14). These analyses were not grouped by Grade however, and thus included secondary students as well. Flynn et al. (2012) found larger effects on standardised tests for comprehension (ES = 0.73), decoding (ES = 0.43), and word identification (ES = 0.41) than we did, but found negative effects on fluency (ES = −0.29; they included students in Grade 5–6 as well). Both Scammaca et al. (2015) and, especially, Flynn et al. (2012) included substantially fewer studies in the comparable Grades and it is also unclear what instructional methods were included in their analyses.

Interventions targeting more domain‐general skills than reading and mathematics had positive effect sizes in our review. The average effect sizes for general academic skills (ES = 0.21) and meta‐cognitive skills (ES = 0.24) were statistically significant, while social‐emotional skills (ES = 0.24) was not significant. These domains were rarely the only targeted domain and we found no evidence that interventions targeting domain‐general skills had larger effect sizes than interventions that only targeted reading and mathematics domains. However, we included only standardised tests in reading and mathematics in the analysis, and targeting domain‐general skills may have important effects on other types of tests.

Few of the earlier reviews with similar target groups as ours included similar categories of domain‐general skills. Dietrichson et al. (2017) included a category called psychological/behavioural interventions (ES = 0.05), which was somewhat similar to a combination of our meta‐cognitive and social‐emotional categories. Pellegrini et al. (2018) included a social‐emotional learning category but their category included whole‐school reforms, which were not included in our review, and their review included interventions targeting general student populations. Thus, their average effect size of 0.03 is difficult to compare with ours.

## 7 AUTHORS' CONCLUSIONS

### 7.1 Implications for practice

Our results indicate that interventions targeting students with or at risk of academic difficulties from kindergarten to Grade 6 have on average positive and statistically significant short‐term and follow‐up effects on standardised tests in reading and mathematics. We believe these average effect sizes are of an educationally meaningful magnitude. Both short‐term and follow‐up effects were larger than the 0.25 standard deviations deemed “substantively important” by What Works Clearinghouse (2014). They are in between the 70th and 80th percentile of the distribution of effect sizes from RCTs of educational interventions evaluated on standardised tests presented in Kraft (2020). Both effect sizes correspond to around a 58% chance that a randomly selected score of a student who received the intervention is greater than the score of a randomly selected student who did not. The short‐term average effect size was around 50% of the estimated average progression of the control groups in the studies in our sample that provided this information. That is, compared with this estimate, the intervention groups progressed on average 50% more than the control groups during the intervention period.

The average effect sizes are around 30%–50% of the gaps in fourth grade between low and high SES students, and between majority and minority students, in the United States (the proportion depends on subject, see Hill et al., 2008, and Lipsey et al., 2012). Although this comparison should not be interpreted as targeted interventions necessarily reducing the achievement gaps by these proportions (see e.g., Kraft, 2020, for a discussion), we believe the magnitudes imply that targeted school‐based interventions of average effectiveness tend to have meaningful impacts on the gaps. At least in the short‐term and if given only to at‐risk students, the most effective interventions in our sample have the potential to eradicate the gaps.

Educational policy makers either have to choose a specific programme, or design their own (e.g., because evidence‐based programmes are not available in their country). While our review did not use programmes as the basis for the analysis (see Inns et al., 2019 and Pellegrini et al., 2018, for such reviews), our results indicate that peer‐assisted instruction and small‐group instruction were significantly associated with effect sizes in all analyses, and in both mathematics and reading. They were associated with significantly larger effect sizes than almost all other instructional methods in meta‐regressions. Peer‐assisted and small‐group instruction are thus likely to be effective components of reading and mathematics programmes targeting students with or at risk of difficulties. Moreover, small‐group instruction had relatively large effect sizes also at follow‐up measurements, meaning that the effects are unlikely to fadeout immediately after the end of intervention.

We did not find robust evidence that other intervention components (the instructional methods or the targeted content domains) were, on average, associated with positive effect sizes when used on their own. Most components were significant when we analysed interventions that included them as one part. However, as these interventions also could include other components, this analysis cannot identify the effects of the separate components.

Three things are important to note about the interpretation of these results. First, we found few single method and domain interventions. For example, among the instructional methods, only a handful of studies examined coaching of personnel, incentives, and progress monitoring in single method interventions. Fractions did have significantly stronger associations with effect sizes than all other math domains. However, as only one intervention targeted only fractions, more research is needed.

Second, with the exception of fractions, we found no evidence that any of the reading, math, or general content domains were significantly associated with effect sizes when we adjusted for other components and study characteristics. This does not mean that interventions did not improve the achievement in the targeted domain, just that content domains were not important predictors of the effect size. A possible interpretation is that the achievement of students with or at risk of academic difficulties can be improved in all domains targeted by interventions. However, our analysis included a moderator based on the targeted domain in the interventions, not the tested domain. It may be the case that interventions targeting, say, reading comprehension failed to improve comprehension but instead improved other domains that were not explicitly targeted in the intervention but were included among the outcomes.

Third, with the exception of single method CAI and peer‐assisted instruction, our analyses showed both substantial and statistically significant heterogeneity of effect sizes. Our results are (weighted) averages and there may thus be highly effective interventions using a particular component, which are “hidden” as the average also contains ineffective interventions. While the average small‐group instruction intervention was effective, some interventions in this category did not have any effects for reasons that we could not fully explain.

We found larger effect sizes in the lower Grades. Although this result is in line with calls for early interventions (e.g., Heckman, 2006), we want to stress that our results do not provide a strong basis for choosing between earlier and later interventions. As intervention types may differ across the Grades in ways we could not adjust for, the moderator analysis does not provide causal estimates of the effect of implementing interventions in different Grades.

Furthermore, the choice between earlier and later interventions should also take into account the long‐term cost‐effectiveness of interventions. Examining the costs of interventions was outside the scope of this review (but few studies included information about costs). Although we found positive and significant follow‐up effects, evidence about long‐term effects was scarce in our review. Therefore, evidence about long‐run cost‐effectiveness was also scarce. This lack of evidence should not be confused with a lack of cost‐effectiveness. We do not know whether the short‐ and medium‐term effects found in many targeted school‐based interventions are long‐lasting. We also do not know why there is fadeout of effects (Bailey et al., 2017; Kang et al., 2019), and therefore do not know the long‐term cost‐effectiveness of interventions.

A final caveat is that our review does not provide information about which interventions, and intervention components, that are suitable for particular groups of students with or at risk of academic difficulties. As our definition of the target group was broad, it contains students with different severity of difficulties. This is for example pertinent for peer‐assisted instruction. For this method, our results, and the results of other reviews, did not agree with the results in Gersten et al. (2009), who studied math interventions for learning disabled students. Learning disabled students was but one of the groups we included and this group has likely more severe difficulties than the average student in our target group. Gersten et al. found small and statistically insignificant average effect sizes of same‐age peer‐tutoring, the dominant type of peer‐assisted instruction in our review. As hypothesised by, for example, McMaster, Fuchs, Fuchs, and Compton (2005) and Slavin et al. (2011), some students may need more intensive support than peer‐assisted instruction. McMaster et al. (2005) was the only study testing this hypothesis in our sample and we could not analyse the question further.

The question of whether some interventions are more or less effective for certain target groups has a parallel to our discussion of group sizes in small‐group instruction. We found highly similar effect sizes in one‐to‐one and small‐group interventions. While we were able to meta‐analyse interventions in which only the group size differed between the intervention groups, the number of studies was so few that the analysis did not yield reliable results. We therefore do not know whether reducing group sizes increase effect sizes and whether students with more difficulties benefit more from smaller group sizes.

### 7.2 Implications for research

While the research literature on the effects of targeted school‐based interventions to students with or at risk of academic difficulties have grown a lot in the last two decades, there is still much to learn about how to design and implement effective interventions. We found substantial heterogeneity in most of our analyses. For example, effect sizes varied considerably within categories of single method/domain interventions, and there was systematic variation also after adjusting for a large set of intervention and study characteristics in meta‐regressions. Our discussion about group sizes in the previous section highlights that more research is needed about relatively basic features of otherwise well‐studied instructional methods such as small‐group instruction. More studies comparing single features of programmes, such as the group size, while keeping all other programme components constant between the treatment and control group would be important additions to the literature. Similarly, more research comparing the effects of the same programme for well‐defined target groups, for example in terms of severity of difficulties or age, would be interesting.

The design of interventions would also benefit from more knowledge of the details of the interventions than we were able to include in our analysis. The number of recurring combinations of instructional methods was for example few. Future studies could draw inspiration from our results and how they relate to pedagogical and psychological theories of learning and instruction. For example, we found a large average effect size in the few interventions that combined small‐group instruction with incentives. It is possible that interventions have larger effects, if they combine for example rewards that reinforce desirable behaviour, as emphasised by social learning theory, with a method that includes other potentially effective features, as emphasised by cognitive developmental theory and pedagogical theory. Peer‐assisted and small‐group instruction interventions have many features that seem beneficial from the perspectives of social learning theory, cognitive developmental theory, and pedagogical theory. They include rapid feedback and instruction that can be tailored to individual students, and they train regulation of behaviour by for example interaction with role models. Such interaction may also improve the development of higher‐order cognitive functions such as learning how to learn. Most other instructional methods we examined do not tick more than one or two of these boxes. In this sense, our results were well aligned with the three theories.

However, CAI can include rapid feedback and tailor‐made instruction, and can include regulation of behaviour through for example rewards. There should furthermore be no doubt that children can be highly motivated to play computer games. Despite these potential strengths, single method CAI interventions had a relatively small (but significant) average effect size and a significantly smaller association with effect sizes than both peer‐assisted and small‐group instruction in meta‐regressions. The most salient difference between CAI and both peer‐assisted and small‐group instruction is the amount of social interaction. In terms of the theoretical perspectives, being instructed by adults or peers, and giving instruction to peers (in cooperative learning interventions) may facilitate higher‐order learning better than computer‐assisted instruction. Being seen and encouraged by adults or peers may improve self‐efficacy and motivation in a way that may be difficult to achieve without social interaction. It would therefore be interesting to see studies of interventions that combine CAI with a social interaction component.

We had difficulties coding some aspects of the interventions. Most pertinent, we believe, are the severity of the difficulties facing the target group and the quality of the control group instruction. As for example, tests differ, comparing the severity of difficulties across contexts is problematic. Comparing treatment and control group test scores to norms that are representative for the student population in the country would be one way to make the severity assessment easier. Another way would be to include more information about risk group status. It was often difficult to classify the control condition, as it was described in much less detail (or not at all) than the intervention condition in many of the included studies. It may often be much more difficult to get precise information about the control group condition, but such information is essential for the interpretation of effect sizes. We believe these two aspects are important sources of the unexplained heterogeneity found throughout the analyses.

The risk of bias of effect sizes included in this review was, in general, high. We excluded a large number of effect sizes from the meta‐analyses, because of our assessment that they had too high risk of bias. Although some of this risk is difficult or costly to fully mitigate in educational research, we believe it is important to improve research designs. Many QES did not show balance tests and did not adjust for any confounders. Another common reason for too high risk of bias ratings was that studies assigned only one unit (e.g., a school, teacher, or class) to the intervention group or the control group, in which case the intervention effect is likely to be confounded with “unit”‐effects. In both these cases, research designs can be improved using relatively small means. There are also several other steps that researchers can take to decrease the risk of bias. Examples include more detailed reporting about how the randomisation, or more generally the assignment of treatment, was done (including assessments of the risk of selection into treatment); using external testers that are blind to treatment status; testing for differential attrition between intervention and control groups; and pre‐publishing protocols and analysis plans.

We believe two more aspects of study characteristics are worth mentioning. First, although the number of studies from other countries has increased during the last years, the literature is still dominated by studies from the United States. Second, as mentioned, studies following students over extended periods after the end of intervention are still rare. The growing literature on the long‐term effects of targeted preschool interventions indicates that it is possible to follow participants for much longer periods (e.g., Conti et al., 2016; Heckman et al., 2010; Reynolds & Temple, 2008; Reynolds et al., 2010; Rossin‐Slater & Wüst, 2019). Our results should be encouraging for such studies in the sense that the results indicate that there may be longer lasting effects. Long‐term effect estimates would also make it possible to examine the ratio of benefits‐to‐costs and thereby give educational policy makers a better basis for choosing interventions to improve the achievement of students with or at risk of academic difficulties.

## ROLES AND RESPONSIBILITIES

Dietrichson, Bøg, Eiberg, Filges, and Anne‐Marie Klint Jørgensen contributed to the writing and revising of the protocol. The search strategy was developed by Anne‐Marie Klint Jørgensen. All authors contributed to the writing of the review. The following review team assistants provided valuable help with screening and coding: Anja Bondebjerg, Christiane Præstgaard Christensen, Anton Dam, Ole Gregersen, Astrid Broni Heinemeier, Freja Jørgensen, Caroline Fromberg Kiehn, Ida Lykke Kristiansen, Erika Lundqvist, Julie Schou Nicolajsen, Vivian Poulsen, Ida Scheel Rasmussen, Tróndur Møller Sandoy, Ida Skytt, Mette Trane, Mai Tødsø Jensen, and Amanda Weber. Jens Dietrichson will be responsible for updating this review as additional evidence accumulates and as funding becomes available.

## CONTRIBUTIONS OF AUTHORS

- Content: Martin Bøg, Jens Dietrichson, Misja Eiberg, Trine Filges, Rasmus Henriksen Klokker, Julie Kaas Seerup.
- Systematic review methods: Martin Bøg, Jens Dietrichson, Trine Filges, Rasmus Henriksen Klokker, Julie Kaas Seerup.
- Statistical analysis: Jens Dietrichson, Julie Kaas Seerup, Rasmus Henriksen Klokker.
- Information retrieval: Bjørn Viinholt.

## SOURCES OF SUPPORT

### Internal sources

VIVE—The Danish Center for Social Science Research.

### External sources

No sources of external support.

## DECLARATIONS OF INTEREST

Three of the authors were involved in a previous review on a related topic: the effects of interventions targeting low SES students (Dietrichson et al., 2017). Dietrichson and Bøg have co‐authored two studies included in the review, both of them started after the protocol of this review was approved. Dietrichson and Bøg were not involved in the screening, coding, and risk of bias assessment of these two studies. The authors have no vested interest in the outcomes of this review, nor any incentive to represent findings in a biased manner.

## PLANS FOR UPDATING THE REVIEW

Jens Dietrichson will be responsible for updating this review, as new studies and additional funding becomes available.

## CHARACTERISTICS OF STUDIES

### Characteristics of included studies

See Supporting Information Appendices H and I.

### Characteristics of excluded studies

Due to the large number of studies screened in full text, we were unable to describe all excluded studies. For a full list of studies excluded in first and second level screening, please contact Jens Dietrichson (jsd@vive.dk).

### Characteristics of studies awaiting classification

No studies are awaiting classification.

### Characteristics of ongoing studies

We found no ongoing relevant studies.

## Supporting information

Supporting information

Supporting information

Supporting information

Supporting information

Supporting information

Supporting information

Supporting information

Supporting information

Supporting information

Supporting information

Supporting information

Supporting information

## Footnotes

1 OECD defines proficiency by reaching Level 2 in the PISA test: “Level 2 [in reading] can be considered a baseline level of proficiency, at which students begin to demonstrate the reading literacy competencies that will enable them to participate effectively and productively in life” (OECD, 2010b, p. 52).

2 “Students proficient at the lowest level can answer questions involving familiar contexts where all relevant information is present and the questions are clearly defined. They are able to identify information and to carry out routine procedures according to direct instructions in explicit situations. They can perform obvious actions that follow immediately from the given stimuli” (OECD, 2010b, p. 132).

3 Despite the choice of grades, Flynn et al. (2012) contain very few studies of interventions targeting students over Grade 6. Note also that all studies in Wanzek et al. (2013), a review of extensive intervention programmes for struggling readers covering Grades 3–12, are included in Scammaca et al. (2015).

4 The following reviews are also related, but focus on more general populations and/or have a more narrow scope (topic and target population in parentheses): McMaster & Fuchs (2002, cooperative learning for students with learning disabilities), Goodwin & Ahn (2010, morphological interventions for children with literacy difficulties), Alfieri et al. (2011, discovery‐based instruction for general student populations), Dexter & Hughes (2011, graphic organizers for students with learning disabilities), Cheung & Slavin (2012, technology applications for general student populations), Kyndt et al. (2013), cooperative learning for general student populations), de Boer et al. (2014, attributes of interventions for general student populations), and Reljić, Ferring, and Martin (2015, bilingual programs to European students). We used these reviews for citation tracking.

5 Authors of reviews, which are mentioned in that section but which were published after our search was completed were not contacted.

6 In addition, we coded a domain called probability, which included, for example, statistics, but we found only one study including this domain and have therefore left it out of the analysis.

7 To illustrate, consider an example with three explanatory variables: an instructional method, a moderator, and an omitted determinant. If the moderator is affected by the omitted determinant of effect sizes and by the instructional method while there is no association between the instructional method and the omitted determinant, then including the moderator will introduce bias in the estimate of the instructional method (so‐called “collider bias”, e.g., Pearl, 2009).

8 We calculated the number of student observations by taking the sample size for each effect size, which is equal to the number of students in the intervention and control group for which the effect size is calculated, and summing over effect sizes. This is *not* the same as the number of participants, as participants take more than one test in most studies and control groups were sometimes compared with more than one intervention group. In these cases, participants contributed to more than one effect size. Our estimations are based on effect sizes and their standard errors and therefore indirectly on the number of student observations.

9 Adding the study characteristics without missing observations (indicators for QES, math tests, and general tests, and the mean Grade) did not visibly change this result (ES = 0.30, CI = [0.24, 0.35]), and reduced heterogeneity only a little (*Q* = 605.1, *τ* ^2^ = 0.061, *I* ^2^ = 69.6).

10 Adding the study characteristics without missing observations (indicators for QES, math tests, and general tests, and the mean Grade) yielded an average ES = 0.24, CI = [0.09, 0.39]), and did not reduce heterogeneity (*Q* = 42.0, *τ* ^2^ = 0.04, *I* ^2^ = 47.6).

11 The average effect size in the 8 CAI studies testing reading is statistically significant (ES = 0.21, CI [0.03, 0.39]), but the adjusted degrees of freedom is 4.3 so the result should be viewed with some caution. The average effect size in the 9 medium‐group studies testing reading was not significant and reasonably close to the estimate in Table 4 (ES = 0.14, CI [−0.04, 0.31], adjusted degrees of freedom = 6.3).

## References

[cl21152-bib-0001] Al Otaiba, S. , Schatschneider, C. , & Silverman, E. (2005). Tutor‐assisted intensive learning strategies in kindergarten: How much is enough? Exceptionality, 13(4), 195–208.

[cl21152-bib-0002] Al‐Hazza, T. C. (2002). *An examination of the effects of the America Reads tutoring program and tutor training on the attitude and academic achievement of urban at‐risk minority students* (Doctoral dissertation). Available from ProQuest Dissertations and Theses (UMI No. 3068649).

[cl21152-bib-0003] Allor, J. , & McCathren, R. (2004). The efficacy of an early literacy tutoring program implemented by college students. Learning Disabilities Research and Practice, 19(2), 116–129.

[cl21152-bib-0004] Allor, J. H. , Fuchs, D. , & Mathes, P. G. (2001). Do students with and without lexical retrieval weaknesses respond differently to instruction? Journal of Learning Disabilities, 34(3), 264–275. 15499880 10.1177/002221940103400306

[cl21152-bib-0005] Amendum, S. J. , Vernon‐Feagans, L. , & Ginsberg, M. C. (2011). The effectiveness of a technologically facilitated classroom‐based early reading intervention: The Targeted Reading Intervention. Elementary School Journal, 112(1), 107–131.

[cl21152-bib-0006] Apel, K. , & Diehm, E. (2014). Morphological awareness intervention with kindergarteners and first and second grade students from low SES homes: A small efficacy study. Journal of Learning Disabilities, 47(1), 65–75. 24191977 10.1177/0022219413509964

[cl21152-bib-0007] Arens, S. A. , Stoker, G. , Barker, J. , Shebby, S. , Wang, X. , Cicchinelli, L. F. , & Williams, J. M. (2012). *Effects of curriculum and teacher professional development on the language proficiency of elementary English language learner students in the Central Region*. (NCEE 2012‐4013). Mid‐continent Research for Education and Learning.

[cl21152-bib-0008] Arvans, R. (2010). *Improving reading fluency and comprehension in elementary students using read naturally* (Doctoral dissertation). Available from ProQuest Dissertations and Theses (UMI No. 3392136).

[cl21152-bib-0009] Baker, D. L. , Basaraba, D. L. , Smolkowski, K. , Conry, J. , Hautala, J. , Richardson, U. , English, S. , & Cole, R. (2017). Exploring the cross‐linguistic transfer of reading skills in Spanish to English in the context of a computer adaptive reading intervention. Bilingual Research Journal, 40(2), 222–239.

[cl21152-bib-0010] Baker, D. L. , Burns, D. , Kame'enui, E. J. , Smolkowski, K. , & Baker, S. K. (2016). Does supplemental instruction support the transition from Spanish to English reading instruction for first‐grade English learners at risk of reading difficulties? Learning Disability Quarterly, 39(4), 226–239.

[cl21152-bib-0011] Baker, S. , Gersten, R. , & Keating, T. (2000). When less may be more: A 2‐Year longitudinal evaluation of a volunteer tutoring program requiring minimal training. Reading Research Quarterly, 35(4), 494–519.

[cl21152-bib-0012] Bar‐Eli, N. , & Raviv, A. (1982). Underachievers as tutors. Journal of Educational Research, 75(3), 139–143.

[cl21152-bib-0013] Barker, T. A. , & Torgesen, J. K. (1995). An evaluation of computer‐assisted instruction in phonological awareness with below average readers. Journal of Educational Computing Research, 13(1), 89–103.

[cl21152-bib-0014] Begeny, J. C. , Mitchell, R. C. , Whitehouse, M. H. , Harris Samuels, F. , & Stage, S. A. (2011). Effects of the HELPS reading fluency program when implemented by classroom teachers with low‐performing second‐grade students. Learning Disabilities Research & Practice, 26(3), 122–133.

[cl21152-bib-0015] Begeny, J. C. , Ross, S. G. , Greene, D. J. , Mitchell, R. C. , & Whitehouse, M. H. (2012). Effects of the Helping Early Literacy with Practice Strategies (HELPS) reading fluency program with Latino English language learners: A preliminary evaluation. Journal of Behavioral Education, 21(2), 134–149.

[cl21152-bib-0016] Benner, G. J. , Nelson, J. R. , Sanders, E. A. , & Ralston, N. C. (2012). Behavior intervention for students with externalizing behavior problems: Primary‐level standard protocol. Exceptional Children, 78(2), 181–198.

[cl21152-bib-0017] Bernstein, L. , Dun Rappaport, C. , Olsho, L. , Hunt, D. , & Levin, M. (2009). *Impact evaluation of the U.S. Department of Education's Student Mentoring Program* (NCEE 2009‐4047). U.S. Department of Education, National Center for Education Evaluation and Regional Assistance, Institute of Education Sciences.

[cl21152-bib-0018] Bettinger, E. P. (2012). Paying to learn: The effect of financial incentives on elementary school test scores. Review of Economics and Statistics, 94(3), 686–698.

[cl21152-bib-0019] Blachman, B. A. , Ball, E. W. , Black, R. S. , & Tangel, D. M. (1994). Kindergarten teachers develop phoneme awareness in low‐income, inner‐city classrooms. Reading and Writing, 6(1), 1–18.

[cl21152-bib-0020] Blachman, B. A. , Schatschneider, C. , Fletcher, J. M. , Francis, D. J. , Clonan, S. M. , Shaywitz, B. A. , & Shaywitz, S. E. (2004). Effects of intensive reading remediation for second and third graders and a 1‐Year follow‐up. Journal of Educational Psychology, 96(3), 444–461.

[cl21152-bib-0021] Blachman, B. A. , Schatschneider, C. , Fletcher, J. M. , Murray, M. S. , Munger, K. A. , & Vaughn, M. G. (2014). Intensive reading remediation in grade 2 or 3: Are there effects a decade later? Journal of Educational Psychology, 106(1), 46–57. 24578581 10.1037/a0033663 PMC3933175

[cl21152-bib-0022] Blanco, P. J. , & Ray, D. C. (2011). Play therapy in elementary schools: A best practice for improving academic achievement. Journal of Counseling & Development, 89(2), 235–243.

[cl21152-bib-0023] Block, C. C. , Parris, S. R. , Reed, K. L. , Whiteley, C. S. , & Cleveland, M. D. (2009). Instructional approaches that significantly increase reading comprehension. Journal of Educational Psychology, 101(2), 262–281.

[cl21152-bib-0024] Block, N. F. (2008). *A study of a response to intervention model for urban sixth‐grade: Analyzing reading, language, and learning differences in Tier 1 and Tier 2* (Doctoral dissertation). Available from ProQuest Dissertations and Theses (UMI No. 3351235).

[cl21152-bib-0025] Borman, G. D. , Benson, J. G. , & Overman, L. (2009). A randomized field trial of the Fast ForWord Language computer‐based training program. Educational Evaluation and Policy Analysis, 31(1), 82–106.

[cl21152-bib-0026] Bryant, D. P. , Bryant, B. R. , Roberts, G. , Vaughn, S. , Pfannenstiel, K. H. , Porterfield, J. , & Gersten, R. (2011). Early numeracy intervention program for first‐grade students with mathematics difficulties. Exceptional Children, 78(1), 7–23.

[cl21152-bib-0027] Burns, M. K. , Senesac, B. V. , & Symington, T. (2004). The effectiveness of the HOSTS program in improving the reading achievement of children at‐risk for reading failure. Reading Research and Instruction, 43(2), 87–103.

[cl21152-bib-0028] Bøg, M. , Dietrichson, J. , & Isaksson, A. A. (2019). *A multi‐sensory tutoring program for students at‐risk of reading difficulties* (IFAU Working Paper 2019:7). Uppsala: IFAU—Institute for Evaluation of Labour Market and Education Policy.

[cl21152-bib-0029] Caggiano, J. A. (2007). *Addressing the learning needs of struggling adolescent readers: The impact of a reading intervention program on students in a middle school setting* (Doctoral dissertation). Available from ProQuest Dissertations and Theses (UMI No. 3257319).

[cl21152-bib-0030] Calhoon, M. B. , Al Otaiba, S. , Cihak, D. , King, A. , & Avalos, A. (2007). Effects of a peer‐mediated program on reading skill acquisition for two‐way bilingual first‐grade classrooms. Learning Disability Quarterly, 30(3), 169–184.

[cl21152-bib-0031] Calhoon, M. B. , Al Otaiba, S. , Greenberg, D. , King, A. , & Avalos, A. (2006). Improving reading skills in predominantly Hispanic title 1 first‐grade classrooms: The promise of peer‐assisted learning strategies. Learning Disabilities Research & Practice, 21(4), 261–272.

[cl21152-bib-0032] Campbell, C. A. , & Brigman, G. (2005). Closing the achievement gap: A structured approach to group counseling. The Journal for Specialists in Group Work, 30(1), 67–82.

[cl21152-bib-0033] Campuzano, L. , Dynarski, M. , Agodini, R. , & Rall, K. (2009). *Effectiveness of reading and mathematics software products: Findings from two student cohorts* (NCEE 2009‐4041). U.S. Department of Education, National Center for Education Evaluation and Regional Assistance, Institute of Education Sciences.

[cl21152-bib-0034] Cantrell, S. C. , Almasi, J. F. , Carter, J. C. , Rintamaa, M. , & Madden, A. (2010). The impact of a strategy‐based intervention on the comprehension and strategy use of struggling adolescent readers. Journal of Educational Psychology, 102(2), 257–280.

[cl21152-bib-0035] Cantrell, S. C. , Almasi, J. F. , Rintamaa, M. , & Carter, J. C. (2016). Supplemental reading strategy instruction for adolescents: A randomized trial and follow‐up study. Journal of Educational Research, 109(1), 7–26.

[cl21152-bib-0036] Carlson, H. L. , Ellison, D. , & Dietrich, J. E. (1984). *Servicing low achieving pupils and pupils with learning disabilities: A comparison of two approaches* (Report). Available from ERIC database (ED283341).

[cl21152-bib-0037] Carlson, J. S. , & Das, J. P. (1997). A process approach to remediating word‐decoding deficiencies in Chapter 1 children. Learning Disability Quarterly, 20(2), 93–102.

[cl21152-bib-0038] Case, L. Pericola , Speece, D. L. , Silverman, R. , Ritchey, K. D. , Schatschneider, C. , Cooper, D. H. , Montanaro, E. , & Jacobs, D. (2010). Validation of a supplemental reading intervention for first‐grade children. Journal of Learning Disabilities, 43(5), 402–417. 20375291 10.1177/0022219409355475 PMC3070172

[cl21152-bib-0039] Christ, T. J. , & Davie, J. (2009). *Empirical evaluation of Read Naturally effects: A randomized control trial* (Report). Minneapolis, MN: University of Minnesota.

[cl21152-bib-0040] Cirino, P. T. , Vaughn, S. , Linan‐Thompson, S. , Cardenas‐Hagan, E. , Fletcher, J. M. , & Francis, D. J. (2009). One‐year follow‐up outcomes of Spanish and English interventions for English language learners at risk for reading problems. American Educational Research Journal, 46(3), 744–781.

[cl21152-bib-0041] Clarke, B. , Doabler, C. T. , Cary, M. S. , Kosty, D. , Baker, S. , Fien, H. , & Smolkowski, K. (2014). Preliminary evaluation of a tier 2 mathematics intervention for first‐grade students: Using a theory of change to guide formative evaluation activities. School Psychology Review, 43(2), 160–178.

[cl21152-bib-0042] Clarke, B. , Doabler, C. T. , Smolkowski, K. , Baker, S. K. , Fien, H. , & Cary, M. S. (2016). Examining the efficacy of a tier 2 kindergarten mathematics intervention. Journal of Learning Disabilities, 49(2), 152–165. 24944163 10.1177/0022219414538514

[cl21152-bib-0043] Clarke, B. , Doabler, C. , Smolkowski, K. , Kurtz Nelson, E. , Fien, H. , Baker, S. K. , & Kosty, D. (2016). Testing the immediate and long‐term efficacy of a Tier 2 kindergarten mathematics intervention. Journal of Research on Educational Effectiveness, 9(4), 607–634.

[cl21152-bib-0044] Clarke, B. , Smolkowski, K. , Baker, S. K. , Fien, H. , Doabler, C. T. , & Chard, D. J. (2011). The impact of a comprehensive tier 1 core kindergarten program on the achievement of students at risk in mathematics. Elementary School Journal, 111(4), 561–584.

[cl21152-bib-0045] Coats, J. H. (2013). *Evaluating the effectiveness of an intervention mathematics class for low achieving middle school students in northwest Georgia* (Doctoral dissertation, Liberty University).

[cl21152-bib-0046] Cooley‐Strickland, M. R. , Griffin, R. S. , Darney, D. , Otte, K. , & Ko, J. (2011). Urban African American youth exposed to community violence: A school‐based anxiety preventive intervention efficacy study. Journal of Prevention & Intervention in the Community, 39(2), 149–166. 21480032 10.1080/10852352.2011.556573 PMC3080109

[cl21152-bib-0047] Deke, J. , Dragoset, L. , Bogen, K. , & Gill, B. (2012). *Impacts of title I supplemental educational services on student achievement* (NCEE 2012‐4053). Washington, DC: U.S. Department of Education, National Center for Education Evaluation and Regional Assistance, Institute of Education Sciences.

[cl21152-bib-0048] Denton, C. A. , Anthony, J. L. , Parker, R. , & Hasbrouck, J. E. (2004). Effects of two tutoring programs on the English reading development of Spanish‐English bilingual students. Elementary School Journal, 104(4), 289–305.

[cl21152-bib-0049] Denton, C. A. , Kethley, C. , Nimon, K. , Kurz, T. B. , Mathes, P. G. , Minyi, S. , & Swanson, E. A. (2010). Effectiveness of a supplemental early reading intervention scaled up in multiple schools. Exceptional Children, 76(4), 394–416.

[cl21152-bib-0050] Drummond, K. , Chinen, M. , Duncan, T. G. , Miller, H. R. , Fryer, L. , Zmach, C. , & Culp, K. (2011). *Impact of the Thinking Reader® software program on grade 6 reading vocabulary, comprehension, strategies, and motivation* (NCEE 2010‐4035). Washington, DC: National Center for Education Evaluation and Regional Assistance, Institute of Education Sciences, U.S. Department of Education.

[cl21152-bib-0051] Dunsmuir, S. , Thomas, C. , May, R. , Monroe, J. , Roiter, T. , & Wellman, S. (2008). Developing an intervention for pupils with writing difficulties: Conceptualisation and analysis. Educational & Child Psychology, 25(3), 150–164.

[cl21152-bib-0052] Dyson, N. I. , Jordan, N. C. , & Glutting, J. (2013). A number sense intervention for low‐income kindergartners at risk for mathematics difficulties. Journal of Learning Disabilities, 46(2), 166–181. 21685346 10.1177/0022219411410233 PMC3566272

[cl21152-bib-0053] Dyson, N. , Jordan, N. C. , Beliakoff, A. , & Hassinger‐Das, B. (2015). A kindergarten number‐sense intervention with contrasting practice conditions for low‐achieving children. Journal for Research in Mathematics Education, 46(3), 331–370. 26388651 10.5951/jresematheduc.46.3.0331 PMC4572740

[cl21152-bib-0054] Elbro, C. , & Petersen, D. K. (2004). Long‐term effects of phoneme awareness and letter sound training: An intervention study with children at risk for dyslexia. Journal of Educational Psychology, 96(4), 660–670.

[cl21152-bib-0055] Fälth, L. , Gustafson, S. , Tjus, T. , Heimann, M. , & Svensson, I. (2013). Computer‐assisted interventions targeting reading skills of children with reading disabilities—A longitudinal study. Dyslexia, 19(1), 37–53. 23338977 10.1002/dys.1450

[cl21152-bib-0056] Fälth, L. , Nilvius, C. , & Anvegård, E. (2015). Intensive reading with reading lists: An intervention study. Creative Education, 6, 2403–2409.

[cl21152-bib-0057] Fien, H. , Doabler, C. T. , Nelson, N. J. , Kosty, D. B. , Clarke, B. , & Baker, S. K. (2016). An examination of the promise of the NumberShire Level 1 Gaming Intervention for improving student mathematics outcomes. Journal of Research on Educational Effectiveness, 9(4), 635–661.

[cl21152-bib-0058] Fives, A. , Kearns, N. , Devaney, C. , Canavan, J. , Russell, D. , Lyons, R. , Eaton, P. , & O'Brien, A. (2013). A one‐to‐one programme for at‐risk readers delivered by older adult volunteers. Review of Education, 1(3), 254–280.

[cl21152-bib-0059] Foster, M. E. , Anthony, J. L. , Clements, D. H. , Sarama, J. , & Williams, J. M. (2016). Improving mathematics learning of kindergarten students through computer‐assisted instruction. Journal for Research in Mathematics Education, 47(3), 206–232.

[cl21152-bib-0060] Foster, M. E. , Anthony, J. L. , Clements, D. H. , Sarama, J. , & Williams, J. J. (2018). Hispanic dual language learning kindergarten students' response to a numeracy intervention: A randomized control trial. Early Childhood Research Quarterly, 43, 83–95.

[cl21152-bib-0061] Fryer, R. G., Jr. (2011). Financial incentives and student achievement: Evidence from randomized trials. Quarterly Journal of Economics, 126(4), 1755–1798.

[cl21152-bib-0062] Fuchs, D. , Elleman, A. M. , Fuchs, L. S. , Peng, P. , Kearns, D. M. , Compton, D. L. , & Miller, A. C. (2016). A randomized control trial of explicit instruction with and without cognitive training to strengthen the reading comprehension of poor readers in first grade. Nashville, TN: Peabody College of Vanderbilt University.

[cl21152-bib-0063] Fuchs, D. , Fuchs, L. S. , Mathes, P. G. , & Simmons, D. C. (1997). Peer‐assisted learning strategies: Making classrooms more responsive to diversity. American Educational Research Journal, 34(1), 174–206.

[cl21152-bib-0064] Fuchs, D. , Fuchs, L. S. , Thompson, A. , Otaiba, S. A. , Yen, L. , Yang, N. J. , Braun, M. , & O'Connor, R. E. (2001). Is reading important in reading‐readiness programs? A randomized field trial with teachers as program implementers. Journal of Educational Psychology, 93(2), 251–267.

[cl21152-bib-0065] Fuchs, D. , Roberts, P. H. , Fuchs, L. S. , & Bowers, J. (1996). Reintegrating students with learning disabilities into the mainstream: A two‐year study. Learning Disabilities Research & Practice, 11(4), 214–229.

[cl21152-bib-0066] Fuchs, L. S. , Compton, D. L. , Fuchs, D. , Paulsen, K. , Bryant, J. D. , & Hamlett, C. L. (2005). The prevention, identification, and cognitive determinants of math difficulty. Journal of Educational Psychology, 97(3), 493–513.

[cl21152-bib-0067] Fuchs, L. S. , Fuchs, D. , Craddock, C. , Hollenbeck, K. N. , Hamlett, C. L. , & Schatschneider, C. (2008). Effects of small‐group tutoring with and without validated classroom instruction on at‐risk students' math problem solving: Are two tiers of prevention better than one? Journal of Educational Psychology, 100(3), 491–509. 19122881 10.1037/0022-0663.100.3.491 PMC2536765

[cl21152-bib-0068] Fuchs, L. S. , Fuchs, D. , Hamlett, C. L. , Phillips, N. B. , & Bentz, J. (1994). Classwide curriculum‐based measurement: Helping general educators meet the challenge of student diversity. Exceptional Children, 60(6), 518–537.

[cl21152-bib-0069] Fuchs, L. S. , Fuchs, D. , Hamlett, C. L. , & Stecker, P. M. (1991). Effects of curriculum‐based measurement and consultation on teacher planning and student achievement in mathematics operations. American Educational Research Journal, 28(3), 617–641.

[cl21152-bib-0070] Fuchs, L. S. , Fuchs, D. , & Karns, K. (2001). Enhancing kindergartners' mathematical development: Effects of peer‐assisted learning strategies. Elementary School Journal, 101(5), 495–510.

[cl21152-bib-0071] Fuchs, L. S. , Fuchs, D. , Karns, K. , Hamlett, C. L. , & Dutka, S. (1997). Effects of task‐focused goals on low‐ achieving students with and without learning disabilities. American Educational Research Journal, 34(3), 513–543.

[cl21152-bib-0072] Fuchs, L. S. , Fuchs, D. , Kazdan, S. , & Allen, S. (1999). Effects of peer‐assisted learning strategies in reading with and without training in elaborated help giving. Elementary School Journal, 99(3), 201–219.

[cl21152-bib-0073] Fuchs, L. S. , Fuchs, D. , Phillips, N. B. , & Hamlett, C. L. (1995). Acquisition and transfer effects of classwide peer‐assisted learning strategies in mathematics for students with varying learning histories. School Psychology Review, 24, 604–620.

[cl21152-bib-0074] Fuchs, L. S. , Fuchs, D. , Yazdian, L. , & Powell, S. R. (2002). Enhancing first‐grade children's mathematical development with peer‐assisted learning strategies. School Psychology Review, 31(4), 569–583.

[cl21152-bib-0075] Fuchs, L. S. , Geary, D. C. , Compton, D. L. , Fuchs, D. , Schatschneider, C. , Hamlett, C. L. , DeSelms, J. , Seethaler, P. M. , Wilson, J. , Craddock, C. F. , Bryant, J. D. , Luther, K. , & Changas, P. (2013). Effects of first‐grade number knowledge tutoring with contrasting forms of practice. Journal of Educational Psychology, 105(1), 58–77. 24065865 10.1037/a0030127 PMC3779611

[cl21152-bib-0076] Fuchs, L. S. , Malone, A. S. , Schumacher, R. F. , Namkung, J. , Hamlett, C. L. , Jordan, N. C. , Siegler, R. S. , Gersten, R. , & Changas, P. (2016). Supported self‐explaining during fraction intervention. Journal of Educational Psychology, 108(4), 493–508.

[cl21152-bib-0077] Fuchs, L. S. , & Mathes, P. G. (1991). *Peer‐mediated reading instruction in special education resource room settings. Final report*. Available from ERIC database (ED408804).

[cl21152-bib-0078] Fuchs, L. S. , Powell, S. R. , Cirino, P. T. , Schumacher, R. F. , Marrin, S. , Hamlett, C. L. , Fuchs, D. , Compton, D. L. , & Changas, P. C. (2014). Does calculation or word‐problem instruction provide a stronger route to prealgebraic knowledge? Journal of Educational Psychology, 106(4), 990–1006. 25541565 10.1037/a0036793 PMC4274629

[cl21152-bib-0079] Fuchs, L. S. , Powell, S. R. , Seethaler, P. M. , Cirino, P. T. , Fletcher, J. M. , Fuchs, D. , & Hamlett, C. L. (2010). The effects of strategic counting instruction, with and without deliberate practice, on number combination skill among students with mathematics difficulties. Learning and Individual Differences, 20, 89–100. 20383313 10.1016/j.lindif.2009.09.003 PMC2850218

[cl21152-bib-0080] Fuchs, L. S. , Powell, S. R. , Seethaler, P. M. , Cirino, P. T. , Fletcher, J. M. , Fuchs, D. , Hamlett, C. L. , & Zumeta, R. O. (2009). Remediating number combination and word problem deficits among students with mathematics difficulties: A randomized control trial. Journal of Educational Psychology, 101(3), 561–576. 19865600 10.1037/a0014701 PMC2768320

[cl21152-bib-0081] Fuchs, L. S. , Schumacher, R. F. , Long, J. , Namkung, J. , Hamlett, C. L. , Cirino, P. T. , Jordan, N. C. , Siegler, R. , Gersten, R. , & Changas, P. (2013). Improving at‐risk learners’ understanding of fractions. Journal of Educational Psychology, 105(3), 683–700.

[cl21152-bib-0082] Fuchs, L. S. , Schumacher, R. F. , Long, J. , Namkung, J. , Malone, A. S. , Wang, A. , Hamlett, C. L. , Jordan, N. C. , Siegler, R. S. , & Changas, P. (2016). Effects of intervention to improve at‐risk fourth graders' understanding, calculations, and word problems with fractions. The Elementary School Journal, 116(4), 625–651.

[cl21152-bib-0083] Fuchs, L. S. , Schumacher, R. F. , Sterba, S. K. , Long, J. , Namkung, J. , Malone, A. , Hamlett, C. L. , Jordan, N. C. , Gersten, R. , Siegler, R. S. , & Changas, P. (2014). Does working memory moderate the effects of fraction intervention? An aptitude‐treatment interaction. Journal of Educational Psychology, 106(2), 499–514.

[cl21152-bib-0084] Fuchs, L. S. , Seethaler, P. M. , Powell, S. R. , Hamlett, C. L. , & Fletcher, J. M. (2008). Effects of preventative tutoring on the mathematical problem solving of third‐grade students with math and reading difficulties. Exceptional Children, 74(2), 155–173. 20209074 10.1177/001440290807400202 PMC2832201

[cl21152-bib-0085] Gattis, M. N. , Morrow‐Howell, N. , McCrary, S. , Lee, M. , Jonson‐Reid, M. , McCoy, H. , Tamar, K. , Molina, A. , & Invernizzi, M. (2010). Examining the effects of New York Experience Corps® program on young readers. Literacy Research and Instruction, 49(4), 299–314.

[cl21152-bib-0086] Gersten, R. , Rolfhaus, E. , Clarke, B. , Decker, L. E. , Wilkins, C. , & Dimino, J. (2015). Intervention for first graders with limited number knowledge: Large‐scale replication of a randomized controlled trial. American Educational Research Journal, 52(3), 516–546.

[cl21152-bib-0087] Gilbert, J. K. , Compton, D. L. , Fuchs, D. , Fuchs, L. S. , Bouton, B. , Barquero, L. A. , & Cho, E. (2013). Efficacy of a first‐grade responsiveness‐to‐intervention prevention model for struggling readers. Reading Research Quarterly, 48(2), 135–154.

[cl21152-bib-0088] Gillies, R. M. , & Ashman, A. F. (2000). The effects of cooperative learning on students with learning difficulties in the lower elementary school. Journal of Special Education, 34(1), 19–27.

[cl21152-bib-0089] Gillum, F. (2013). *The impact of a counseling/tutorial program on at‐risk students* (Doctoral dissertation). Available from ProQuest Dissertations and Theses (UMI No. 3542691).

[cl21152-bib-0090] Given, B. K. , Wasserman, J. D. , Chari, S. A. , Beattie, K. , & Eden, G. F. (2008). A randomized, controlled study of computer‐based intervention in middle school struggling readers. Brain and Language, 106(2), 83–97. 18657684 10.1016/j.bandl.2007.12.001

[cl21152-bib-0091] Greenwood, C. R. , Terry, B. , Utley, C. A. , & Montagna, D. (1993). Achievement, placement, and services: Middle school benefits of Classwide Peer Tutoring used at the elementary school. School Psychology Review, 22(3), 497–516.

[cl21152-bib-0092] Gunn, B. , Smolkowski, K. , Biglan, A. , Black, C. , & Blair, J. (2005). Fostering the development of reading skill through supplemental instruction: Results for Hispanic and non‐Hispanic students. Journal of Special Education, 39(2), 66–85. 17364009 10.1177/00224669050390020301 PMC1828030

[cl21152-bib-0093] Gustafson, S. , Falth, L. , Svensson, I. , Tjus, T. , & Heimann, M. (2011). Effects of three interventions on the reading skills of children with reading disabilities in grade 2. Journal of Learning Disabilities, 44(2), 123–135. 21383105 10.1177/0022219410391187

[cl21152-bib-0094] Gustafson, S. , Ferreira, J. , & Rönnberg, J. (2007). Phonological or orthographic training for children with phonological or orthographic decoding deficits. Dyslexia, 13(3), 211–229. 17624906 10.1002/dys.339

[cl21152-bib-0095] Gustafson, S. , Samuelsson, S. , & Rönnberg, J. (2000). Why do some resist phonological intervention? A Swedish longitudinal study of poor readers in grade 4. Scandinavian Journal of Educational Research, 44(2), 145–162.

[cl21152-bib-0096] Hansson, Å. (2014). Effekter av intensivundervisning i matematik. Utvärdering av ett pilotprojekt med personlig tränare i matematik för elever i behov av särskilt stöd. Gothenburg: University of Gothenburg.

[cl21152-bib-0097] Hassler Hallstedt, M. , Klingberg, T. , & Ghaderi, A. (2018). Short and long‐term effects of a mathematics tablet intervention for low performing second graders. Journal of Educational Psychology, 110(8), 1127–1148.

[cl21152-bib-0098] Hatcher, P. J. , Hulme, C. , Miles, J. N. V. , Carroll, J. M. , Hatcher, J. , Gibbs, S. , Smith, G. , Bowyer‐Crane, C. , & Snowling, M. J. (2006). Efficacy of small group reading intervention for beginning readers with reading‐delay: A randomised controlled trial. Journal of Child Psychology and Psychiatry, 47(8), 820–827. 16898996 10.1111/j.1469-7610.2005.01559.x

[cl21152-bib-0099] Hatz, H. , & Sachse, S. (2010). Prävention von Lese‐Rechtschreibstörungen. Zeitschrift für Entwicklungspsychologie und Pädagogische Psychologie, 42, 226–240.

[cl21152-bib-0100] Heistad, D. (2005). The effects of Read Naturally on fluency and reading comprehension: A supplemental service intervention (four‐school study). Saint Paul, MN: Read Naturally, Inc.

[cl21152-bib-0101] Helf, S. , Cooke, N. L. , & Flowers, C. P. (2009). Effects of two grouping conditions on students who are at risk for reading failure. Preventing School Failure: Alternative Education for Children and Youth, 53(2), 113–128.

[cl21152-bib-0102] Heller, L. R. , & Fantuzzo, J. W. (1993). Reciprocal peer tutoring and parent partnership: Does parent involvement make a difference? School Psychology Review, 22(3), 517–534.

[cl21152-bib-0103] Hempenstall, K. (2008). Corrective Reading: An evidence‐based remedial reading intervention. Australasian Journal of Special Education, 32(1), 23–54.

[cl21152-bib-0104] Hitchcock, J. , Dimino, J. , Kurki, A. , Wilkins, C. , & Gersten, R. (2011). *The impact of Collaborative Strategic Reading on the reading comprehension of grade 5 students in linguistically diverse schools* (NCEE 2011‐4001). U.S. Department of Education, National Center for Education Evaluation and Regional Assistance, Institute of Education Sciences.

[cl21152-bib-0105] Hooper, S. , Costa, L.‐J. , McBee, M. , Anderson, K. , Yerby, D. , Childress, A. , & Knuth, S. (2013). A written language intervention for at‐risk second grade students: A randomized controlled trial of the process assessment of the learner lesson plans in a tier 2 response‐to‐intervention (RtI) model. Annals of Dyslexia, 63(1), 44–64. 21837551 10.1007/s11881-011-0056-y

[cl21152-bib-0106] Hurry, J. , & Sylva, K. (2007). Long‐term outcomes of early reading intervention. Journal of Research in Reading, 30(3), 227–248.

[cl21152-bib-0107] Jacob, R. , Armstrong, C. , Bowden, A. B. , & Pan, Y. (2016). Leveraging volunteers: An experimental evaluation of a tutoring program for struggling readers. Journal of Research on Educational Effectiveness, 9(Suppl. 1), 67–92.

[cl21152-bib-0108] Jenkins, J. R. , Peyton, J. A. , Sanders, E. A. , & Vadasy, P. F. (2004). Effects of reading decodable texts in supplemental first‐grade tutoring. Scientific Studies of Reading, 8(1), 53–85.

[cl21152-bib-0109] Jones, S. M. , Brown, J. L. , Hoglund, W. L. , & Aber, J. L. (2010). A school‐randomized clinical trial of an integrated social–emotional learning and literacy intervention: Impacts after 1 school year. Journal of Consulting and Clinical Psychology, 78(6), 829–842. 21114343 10.1037/a0021383

[cl21152-bib-0110] Jordan, N. C. , Glutting, J. , Dyson, N. , Hassinger‐Das, B. , & Irwin, C. (2012). Building kindergartners' number sense: A randomized controlled study. Journal of Educational Psychology, 104(3), 647–660. 25866417 10.1037/a0029018 PMC4389641

[cl21152-bib-0111] Kerins, M. R. , Trotter, D. , & Schoenbrodt, L. (2010). Effects of a Tier 2 intervention on literacy measures: Lessons learned. Child Language Teaching and Therapy, 26(3), 287–302.

[cl21152-bib-0112] Kidd, J. K. , Pasnak, R. , Gadzichowski, K. M. , Gallington, D. A. , McKnight, P. , Boyer, C. E. , & Carlson, A. (2014). Instructing first‐grade children on patterning improves reading and mathematics. Early Education and Development, 25(1), 134–151.

[cl21152-bib-0113] Klingner, J. K. , Vaughn, S. , & Schumm, J. S. (1998). Collaborative strategic reading during social studies in heterogeneous fourth‐grade classrooms. Elementary School Journal, 99(1), 3–22.

[cl21152-bib-0114] Lee, Y. S. , Morrow‐Howell, N. , Jonson‐Reid, M. , & McCrary, S. (2012). The effect of the Experience Corps**®** program on student reading outcomes. Education and Urban Society, 44(1), 97–118.

[cl21152-bib-0115] León, A. , Villares, E. , Brigman, G. , Webb, L. , & Peluso, P. (2011). Closing the achievement gap of Latina/Latino students: A school counseling response. Counseling Outcome Research and Evaluation, 2(1), 73–86.

[cl21152-bib-0116] Lesaux, N. K. , Kieffer, M. J. , Faller, S. E. , & Kelley, J. G. (2010). The effectiveness and ease of implementation of an academic vocabulary intervention for linguistically diverse students in urban middle schools. Reading Research Quarterly, 45(2), 196–228.

[cl21152-bib-0117] Lesaux, N. K. , Kieffer, M. J. , Kelley, J. G. , & Harris, J. R. (2014). Effects of academic vocabulary instruction for linguistically diverse adolescents: Evidence from a randomized field trial. American Educational Research Journal, 51(6), 1159–1194.

[cl21152-bib-0118] Little, C. A. , McCoach, D. B. , & Reis, S. M. (2014). Effects of differentiated reading instruction on student achievement in middle school. Journal of Advanced Academics, 25(4), 384–402.

[cl21152-bib-0119] Macaruso, P. , Hook, P. E. , & McCabe, R. (2006). The efficacy of computer‐based supplementary phonics programs for advancing reading skills in at‐risk elementary students. Journal of Research in Reading, 29(2), 162–172.

[cl21152-bib-0120] Mackiewicz, S. M. (2010). *Effects of task training on kindergarten students' performance on early literacy measures* (Doctoral dissertation). Available from ProQuest Dissertations and Theses (UMI No. 3404852).

[cl21152-bib-0121] Marx, E. , & Keller, K. (2010). Effekte eines induktiven Denktrainings auf die Denk‐und Sprachentwicklung bei Vorschulkindern und Erstklässlern in benachteiligten Stadtteilen. Zeitschrift für Pädagogische Psychologie, 24, 139–146.

[cl21152-bib-0122] Mathes, P. G. , & Fuchs, L. S. (1993). Peer‐mediated reading instruction in special education resource rooms. Learning Disabilities Research & Practice, 8(4), 233–243.

[cl21152-bib-0123] Mathes, P. G. , Torgesen, J. K. , Clancy‐Menchetti, J. , Santi, K. , Nicholas, K. , Robinson, C. , & Grek, M. (2003). A comparison of teacher‐directed versus peer‐assisted instruction to struggling first‐grade readers. The Elementary School Journal, 103(5), 459–479.

[cl21152-bib-0124] May, H. , Gray, A. , Sirinides, P. , Goldsworthy, H. , Armijo, M. , Sam, C. , Gillespie, J. N. , & Tognatta, N. (2015). Year one results from the multisite randomized evaluation of the i3 scale‐up of Reading Recovery. American Educational Research Journal, 52(3), 547–581.

[cl21152-bib-0125] Mayfield, L. G. (2000). *The effects of structured one‐on‐one tutoring in sight word recognition of first grade students at‐risk for reading failure*. Paper Presented at the Annual Meeting of the Mid‐South Educational Research Association, in Bowling Green, KY, November 15–17, 2000. Available from ERIC database (ED449630).

[cl21152-bib-0126] Meier, J. D. , & Invernizzi, M. (2001). Book buddies in the Bronx: Testing a model for America Reads. Journal of Education for Students Placed at Risk, 6(4), 319–333.

[cl21152-bib-0127] Messer, D. , & Nash, G. (2018). An evaluation of the effectiveness of a computer‐assisted reading intervention. Journal of research in reading, 41(1), 140–158.

[cl21152-bib-0128] Ming, K. (2007). *The effects of a fluency intervention on the oral reading fluency of first grade students at risk for reading failure* (Doctoral dissertation). Available from ProQuest Dissertations and Theses (UMI No. 3268792).

[cl21152-bib-0129] Mitchell, M. J. , & Fox, B. J. (2001). The effects of computer software for developing phonological awareness in low‐progress readers. Reading Research and Instruction, 40(4), 315–332.

[cl21152-bib-0130] Nelson, J. R. , Benner, G. J. , & Gonzales, J. (2005). An investigation of the effects of a prereading intervention on the early literacy skills of children at risk of emotional disturbance and reading problems. Journal of Emotional and Behavioral Disorders, 13(1), 3–12.

[cl21152-bib-0131] Nelson, J. R. , Stage, S. A. , Epstein, M. H. , & Pierce, C. D. (2005). Effects of a prereading intervention on the literacy and social skills of children. Exceptional Children, 72(1), 29–45.

[cl21152-bib-0132] Nielsen, D. C. , & Friesen, L. D. (2012). A study of the effectiveness of a small‐group intervention on the vocabulary and narrative development of at‐risk kindergarten children. Reading Psychology, 33(3), 269–299.

[cl21152-bib-0133] Nunnery, J. A. , Ross, S. M. , & McDonald, A. (2006). A randomized experimental evaluation of the impact of Accelerated Reader/Reading Renaissance implementation on reading achievement in grades 3 to 6. Journal of Education for Students Placed at Risk, 11(1), 1–18.

[cl21152-bib-0134] Nussbaum, S. S. (2010). *The effects of ‘Brain Gym' as a general education intervention: Improving academic performance and behaviors* (Doctoral dissertation). Available from ERIC database (ED517276).

[cl21152-bib-0135] O'Connor, R. E. , Bell, K. M. , Harty, K. R. , Larkin, L. K. , Sackor, S. M. , & Zigmond, N. (2002). Teaching reading to poor readers in the intermediate grades: A comparison of text difficulty. Journal of Educational Psychology, 94(3), 474–485.

[cl21152-bib-0136] O'Connor, R. E. , Bocian, K. , Beebe‐Frankenberger, M. , & Linklater, D. L. (2010). Responsiveness of students with language difficulties to early intervention in reading. Journal of Special Education, 43(4), 220–235.

[cl21152-bib-0137] O'Shaughnessy, T. E. , & Swanson, H. L. (2000). A comparison of two reading interventions for children with reading disabilities. Journal of Learning Disabilities, 33(3), 257–277. 15505964 10.1177/002221940003300304

[cl21152-bib-0138] Pasnak, R. , Kidd, J. K. , Gadzichowski, M. K. , Gallington, D. A. , Saracina, R. P. , & Addison, K. T. (2009). Promoting early abstraction to promote early literacy and numeracy. Journal of Applied Developmental Psychology, 30(3), 239–249.

[cl21152-bib-0139] Pasnak, R. , Madden, S. E. , Malabonga, V. A. , Holt, R. , & Martin, J. W. (1996). Persistence of gains from instruction in classification, seriation, and conservation. The Journal of Educational Research, 90(2), 87–92.

[cl21152-bib-0140] Portes, P. R. , González Canché, M. , Boada, D. , & Whatley, M. E. (2018). Early evaluation findings from the Instructional Conversation study: Culturally responsive teaching outcomes for diverse learners in elementary school. American Educational Research Journal, 55(3), 488–531.

[cl21152-bib-0141] Ransford‐Kaldon, C. , Flynt, E. S. , & Ross, C. (2011). *A randomized controlled trial of a response‐to‐intervention (RTI) Tier 2 literacy program: Leveled Literacy Intervention (LLI)*. Available from ERIC database (ED518772).

[cl21152-bib-0142] Rashotte, C. A. , MacPhee, K. , & Torgesen, J. K. (2001). The effectiveness of a group reading instruction program with poor readers in multiple grades. Learning Disability Quarterly, 24(2), 119–134.

[cl21152-bib-0143] Rehmann, R. (2005). *The effect of Earobics Step 1, software on student acquisition of phonological awareness skills* (Doctoral dissertation, University of Oregon).

[cl21152-bib-0144] Ritchey, K. D. , Palombo, K. , Silverman, R. D. , & Speece, D. L. (2017). Effects of an informational text reading comprehension intervention for fifth‐grade students. Learning Disability Quarterly, 40(2), 68–80.

[cl21152-bib-0145] Rouse, C. E. , & Krueger, A. B. (2004). Putting computerized instruction to the test: A randomized evaluation of a “scientifically based” reading program. Economics of Education Review, 23(4), 323–338.

[cl21152-bib-0146] Rutt, S. , Easton, C. , & Stacey, O. (2014). Catch up numeracy: Evaluation report and executive summary. Education Endowment Foundation.

[cl21152-bib-0147] Ryder, J. F. , Tunmer, W. E. , & Greaney, K. T. (2008). Explicit instruction in phonemic awareness and phonemically based decoding skills as an intervention strategy for struggling readers in whole language classrooms. Reading and Writing, 21(4), 349–369.

[cl21152-bib-0148] Sáenz, L. M. (2002). *Peer‐assisted Learning Strategies for Limited English Proficient Students with Learning Disabilities* (Doctoral dissertation). Available from ProQuest Dissertations and Theses (UMI no. 3058720).

[cl21152-bib-0149] Santa, C. M. , & Høien, T. (1999). An assessment of Early Steps: A program for early intervention of reading problems. Reading Research Quarterly, 34(1), 54–79.

[cl21152-bib-0150] Schwartz, R. M. (2005). Literacy learning of at‐risk first‐grade students in the reading recovery early intervention. Journal of Educational Psychology, 97(2), 257–267.

[cl21152-bib-0151] Schwartz, R. M. , Schmitt, M. C. , & Lose, M. K. (2012). Effects of teacher‐student ratio in response to intervention approaches. The Elementary School Journal, 112(4), 547–567.

[cl21152-bib-0152] Scientific Learning Corporation . (2005). Improved early reading skills by students in three districts who used Fast ForWord® to Reading 1. MAPS for Learning: Product Reports, 9(1), 1–5.

[cl21152-bib-0153] Simmons, D. , Hairrell, A. , Edmonds, M. , Vaughn, S. , Larsen, R. , Willson, V. , Rupley, W. , & Byrns, G. (2010). A comparison of multiple‐strategy methods: Effects on fourth‐grade students' general and content‐specific reading comprehension and vocabulary development. Journal of Research on Educational Effectiveness, 3(2), 121–156.

[cl21152-bib-0154] Slavin, R. E. , Madden, N. A. , & Leavey, M. (1984a). Effects of cooperative learning and individualized instruction on mainstreamed students. Exceptional Children, 50(5), 434–448. 6698102 10.1177/001440298405000506

[cl21152-bib-0155] Slavin, R. E. , Madden, N. A. , & Leavey, M. (1984b). Effects of team assisted individualization on the mathematics achievement of academically handicapped and nonhandicapped students. Journal of Educational Psychology, 76(5), 813–819.

[cl21152-bib-0156] Slavin, R. , Chamberlain, A. , Daniels, C. , & Madden, N. A. (2009). The Reading Edge: A randomized evaluation of a middle school cooperative reading program. Effective Education, 1(1), 13–26.

[cl21152-bib-0157] Smith, J. L. M. , Nelson, N. J. , Fien, H. , Smolkowski, K. , Kosty, D. , & Baker, S. K. (2016). Examining the efficacy of a multitiered intervention for at‐risk readers in grade 1. Elementary School Journal, 116(4), 549–573.

[cl21152-bib-0158] Smith, T. M. , Cobb, P. , Farran, D. C. , Cordray, D. S. , & Munter, C. (2013). Evaluating math recovery: Assessing the causal impact of a diagnostic tutoring program on student achievement. American Educational Research Journal, 50(2), 397–428.

[cl21152-bib-0159] Solari, E. J. , Denton, C. A. , Petscher, Y. , & Haring, C. (2018). Examining the effects and feasibility of a teacher‐implemented tier 1 and Tier 2 intervention in word reading, fluency, and comprehension. Journal of Research on Educational Effectiveness, 11(2), 163–191.

[cl21152-bib-0160] Sood, K. A. (1999). *The effect of attribution and persistence retraining on reading achievement in third, fourth and fifth grade learning‐disabled students* (Doctoral dissertation). Available from ProQuest Dissertations and Theses (UMI no. 9939559).

[cl21152-bib-0161] Swanson, H. L. , Moran, A. S. , Bocian, K. , Lussier, C. , & Zheng, X. (2012). Generative strategies, working memory, and word problem solving accuracy in children at risk for math disabilities. Learning Disability Quarterly, 36(4), 203–214.

[cl21152-bib-0162] Swanson, H. L. , Moran, A. , Lussier, C. , & Fung, W. (2014). The effect of explicit and direct generative strategy training and working memory on word problem‐solving accuracy in children at risk for math difficulties. Learning Disability Quarterly, 37(2), 111–123.

[cl21152-bib-0163] Swanson, H. L. , & O'Connor, R. (2009). The role of working memory and fluency practice on the reading comprehension of students who are dysfluent readers. Journal of Learning Disabilities, 42(6), 548–575. 19745196 10.1177/0022219409338742

[cl21152-bib-0164] Swanson, H. L. , Orosco, M. J. , & Lussier, C. M. (2014). The effects of mathematics strategy instruction for children with serious problem‐solving difficulties. Exceptional Children, 80(2), 149–168.

[cl21152-bib-0165] Therrien, W. J. , Wickstrom, K. , & Jones, K. (2006). Effect of a combined repeated reading and question generation intervention on reading achievement. Learning Disabilities Research & Practice, 21(2), 89–97.

[cl21152-bib-0166] Tolan, P. , Gorman‐Smith, D. , & Henry, D. (2004). Supporting families in a high‐risk setting: Proximal effects of the SAFE Children Preventive Intervention. Journal of Consulting and Clinical Psychology, 72(5), 855–869. 15482043 10.1037/0022-006X.72.5.855

[cl21152-bib-0167] Toll, S. , & Van Luit, J. (2012). Early numeracy intervention for low‐performing kindergartners. Journal of Early Intervention, 34(4), 243–264.

[cl21152-bib-0168] Toll, S. , & Van Luit, J. (2014). Effects of remedial numeracy instruction throughout kindergarten starting at different ages: Evidence from a large‐scale longitudinal study. Learning & Instruction, 33, 39–49.

[cl21152-bib-0169] Top, B. L. , & Osguthorpe, R. T. (1987). Reverse‐role tutoring: The effects of handicapped students tutoring regular class students. Elementary School Journal, 87(4), 413–423.

[cl21152-bib-0170] Torgerson, C. J. , Wiggins, A. , Torgerson, D. J. , Ainsworth, H. , Barmby, P. , Hewitt, C. , & Tymms, P. (2011). *Every Child Counts: The independent evaluation. Technical report* (Research Report DFE‐RR091a). Department for Education.

[cl21152-bib-0171] Torgesen, J. K. , Wagner, R. K. , Rashotte, C. A. , Herron, J. , & Lindamood, P. (2010). Computer‐assisted instruction to prevent early reading difficulties in students at risk for dyslexia: Outcomes from two instructional approaches. Annals of Dyslexia, 60(1), 40–56. 20052566 10.1007/s11881-009-0032-y PMC2888606

[cl21152-bib-0172] Torgesen, J. K. , Wagner, R. K. , Rashotte, C. A. , Rose, E. , Lindamood, P. , Conway, T. , & Garvan, C. (1999). Preventing reading failure in young children with phonological processing disabilities: Group and individual responses to instruction. Journal of Educational Psychology, 91(4), 579–593.

[cl21152-bib-0173] Torgesen, J. , Myers, D. , Schirm, A. , Stuart, E. , Vartivarian, S. , Mansfield, W. , & Haan, C. (2006). *National Assessment of Title I: Interim report. Volume II: Closing the reading gap—First year findings from a randomized trial of four reading interventions for striving readers* (NCEE 2006‐4002). U.S. Department of Education, National Center for Education Evaluation and Regional Assistance, Institute of Education Sciences.

[cl21152-bib-0174] Torgesen, J. , Schirm, A. , Castner, L. , Vartivarian, S. , Mansfield, W. , Myers, D. , & Haan, C. (2007). *National Assessment of Title I. Final report. Volume II: Closing the reading gap‐‐Findings from a randomized trial of four reading interventions for striving readers* (NCEE 2008‐4013). U.S. Department of Education, National Center for Education Evaluation and Regional Assistance, Institute of Education Sciences.

[cl21152-bib-0175] Toste, J. R. , Capin, P. , Vaughn, S. , Roberts, G. J. , & Kearns, D. M. (2017). Multisyllabic word‐reading instruction with and without motivational beliefs training for struggling readers in the upper elementary grades: A pilot investigation. Elementary School Journal, 117(4), 593–615.

[cl21152-bib-0176] Tse, L. , & Nicholson, T. (2014). The effect of phonics‐enhanced Big Book reading on the language and literacy skills of 6‐year‐old pupils of different reading ability attending lower SES schools. Frontiers in Psychology, 5, 1222. 25431560 10.3389/fpsyg.2014.01222 PMC4230049

[cl21152-bib-0177] Vadasy, P. F. , Elizabeth, A. S. , & Tudor, S. (2007). Effectiveness of paraeducator‐supplemented individual instruction beyond basic decoding skills. Journal of Learning Disabilities, 40(6), 508–525. 18064977 10.1177/00222194070400060301

[cl21152-bib-0178] Vadasy, P. F. , Jenkins, J. R. , & Pool, K. (2000). Effects of tutoring in phonological and early reading skills on students at risk for reading disabilities. Journal of Learning Disabilities, 33(6), 579–590. 15495399 10.1177/002221940003300606

[cl21152-bib-0179] Vadasy, P. F. , & Sanders, E. A. (2008a). Benefits of repeated reading intervention for low‐achieving fourth‐ and fifth‐grade students. Remedial and Special Education, 29(4), 235–249.

[cl21152-bib-0180] Vadasy, P. F. , & Sanders, E. A. (2008b). Code‐oriented instruction for kindergarten students at risk for reading difficulties: A replication and comparison of instructional groupings. Reading and Writing: An Interdisciplinary Journal, 21(9), 929–963.

[cl21152-bib-0181] Vadasy, P. F. , & Sanders, E. A. (2008c). Repeated reading intervention: Outcomes and interactions with readers' skills and classroom instruction. Journal of Educational Psychology, 100(2), 272–290.

[cl21152-bib-0182] Vadasy, P. F. , & Sanders, E. A. (2010). Efficacy of supplemental phonics‐based instruction for low‐skilled kindergarteners in the context of language minority status and classroom phonics instruction. Journal of Educational Psychology, 102(4), 786–803.

[cl21152-bib-0183] Vadasy, P. F. , & Sanders, E. A. (2011). Efficacy of supplemental phonics‐based instruction for low‐skilled first graders: How language minority status and pretest characteristics moderate treatment response. Scientific Studies of Reading, 15(6), 471–497.

[cl21152-bib-0184] Vadasy, P. F. , & Sanders, E. A. (2012). Two‐year follow‐up of a kindergarten phonics intervention for English learners and native English speakers: Contextualizing treatment impacts by classroom literacy instruction. Journal of Educational Psychology, 104(4), 987–1005.

[cl21152-bib-0185] Vadasy, P. F. , & Sanders, E. A. (2013). Two‐year follow‐up of a code‐oriented intervention for lower‐skilled first‐graders: The influence of language status and word reading skills on third‐grade literacy outcomes. Reading and Writing, 26(6), 821–843.

[cl21152-bib-0186] Vadasy, P. F. , Sanders, E. A. , & Peyton, J. A. (2006). Paraeducator‐supplemented instruction in structural analysis with text reading practice for second and third graders at risk for reading problems. Remedial and Special Education, 27(6), 365–378.

[cl21152-bib-0187] Van De Rijt, B. A. , & Van Luit, J. E. (1998). Effectiveness of the Additional Early Mathematics program for teaching children early mathematics. Instructional Science, 26(5), 337–358.

[cl21152-bib-0188] Vaughn, S. , Cirino, P. T. , Linan‐Thompson, S. , Mathes, P. G. , Carlson, C. D. , Hagan, E. C. , Pollard‐Durodola, S. D. , Fletcher, J. M. , & Francis, D. J. (2006). Effectiveness of a Spanish intervention and an English intervention for English‐language learners at risk for reading problems. American Educational Research Journal, 43(3), 449–487.

[cl21152-bib-0189] Vaughn, S. , Cirino, P. T. , Tolar, T. , Fletcher, J. M. , Cardenas‐Hagan, E. , Carlson, C. D. , & Francis, D. J. (2008). Long‐term follow‐up of Spanish and English interventions for first‐grade English language learners at risk for reading problems. Journal of Research on Educational Effectiveness, 1(3), 179–214.

[cl21152-bib-0190] Vaughn, S. , Cirino, P. T. , Wanzek, J. , Wexler, J. , Fletcher, J. M. , Denton, C. D. , Barth, A. , Romain, M. , & Francis, D. J. (2010). Response to intervention for middle school students with reading difficulties: Effects of a primary and secondary intervention. School psychology review, 39(1), 3–21. 21479079 PMC3072689

[cl21152-bib-0191] Vaughn, S. , Linan‐Thompson, S. , Kouzekanani, K. , Pedrotty Bryant, D. , Dickson, S. , & Blozis, S. A. (2003). Reading instruction grouping for students with reading difficulties. Remedial and Special Education, 24(5), 301–315.

[cl21152-bib-0192] Vaughn, S. , Linan‐Thompson, S. , Mathes, P. G. , Cirino, P. T. , Carlson, C. D. , Pollard‐Durodola, S. D. , Cardenas‐Hagan, E. , & Francis, D. J. (2006b). Effectiveness of Spanish intervention for first‐grade English language learners at risk for reading difficulties. Journal of Learning Disabilities, 39(1), 56–73. 16512083 10.1177/00222194060390010601

[cl21152-bib-0193] Vaughn, S. , Mathes, P. , Linan‐Thompson, S. , Cirino, P. , Carlson, C. , Pollard‐Durodola, S. , Cardenas‐Hagan, E. , & Francis, D. (2006c). Effectiveness of an English intervention for first‐grade English language learners at risk for reading problems. Elementary School Journal, 107(2), 153–181. 10.1177/00222194060390010601 16512083

[cl21152-bib-0194] Vaughn, S. , Solís, M. , Miciak, J. , Taylor, W. P. , & Fletcher, J. M. (2016). Effects from a randomized control trial comparing researcher and school‐implemented treatments with fourth graders with significant reading difficulties. Journal of Research on Educational Effectiveness, 9(1), 23–44. 28491206 10.1080/19345747.2015.1126386 PMC5421628

[cl21152-bib-0195] Wang, C. , & Algozzine, B. (2008). Effects of targeted intervention on early literacy skills of at‐risk students. Journal of Research in Childhood Education, 22(4), 425–439.

[cl21152-bib-0196] Wang, H. , & Woodworth, K. (2011). *A randomized controlled trial of two online mathematics curricula*. Available from ERIC database (ED528686).

[cl21152-bib-0197] Wanzek, J. , Petscher, Y. , Al Otaiba, S. , Kent, S. C. , Schatschneider, C. , Haynes, M. , Rivas, B. K. , & Jones, F. G. (2016). Examining the average and local effects of a standardized treatment for fourth graders with reading difficulties. Journal of Research on Educational Effectiveness, 9(1), 45–66.

[cl21152-bib-0198] Wanzek, J. , Petscher, Y. , Otaiba, S. A. , Rivas, B. K. , Jones, F. G. , Kent, S. C. , Schatschneider, C. , & Mehta, P. (2017). Effects of a year long supplemental reading intervention for students with reading difficulties in fourth grade. Journal of Educational Psychology, 109(8), 1103–1119.

[cl21152-bib-0199] Wanzek, J. , & Roberts, G. (2012). Reading interventions with varying instructional emphases for fourth graders with reading difficulties. Learning Disability Quarterly, 35(2), 90–101.

[cl21152-bib-0200] Weichenthal, D. M. (1985). *An experimental study of the performance and transfer effects of a metacognitive, self‐regulatory strategy on reading for learning‐disabled students* (Doctoral dissertation). San Francisco: University of San Francisco.

[cl21152-bib-0201] Weiss, J. A. , Thurlow, M. L. , Christenson, S. L. , & Ysseldyke, J. E. (1989). *Paired reading with adult volunteer tutors as a reading intervention for students with reading difficulties*. Paper presented at the Annual Meeting of the American Educational Research Association, March 27‐31, San Francisco, CA. Retrieved from ERIC database (ED305606).

[cl21152-bib-0202] White, P. M. (2000). *Promoting mathematics achievement, academic efficacy, and cognitive development of at‐risk adolescents through deliberate psychological education* (Doctoral dissertation). Available from ProQuest Dissertations and Theses (UMI No. 9965210).

[cl21152-bib-0203] Wolff, U. (2011). Effects of a randomised reading intervention study: An application of structural equation modelling. Dyslexia, 17, 295–311. 21953739 10.1002/dys.438

[cl21152-bib-0204] Wolff, U. (2016). Effects of a randomized reading intervention study aimed at 9‐year‐olds: A 5‐year follow‐up. Dyslexia, 22, 85–100. 27146373 10.1002/dys.1529

[cl21152-bib-0205] Xin, F. (1996). *The effects of computer‐assisted cooperative learning in mathematics in integrated classrooms for students with and without disabilities. Final report*. Retrieved from ERIC database (ED412696).

[cl21152-bib-0206] Abbott, S. P. , & Berninger, V. W. (1999). It's never too late to remediate: Teaching word recognition to students with reading disabilities in grades 4–7. Annals of dyslexia, 49(1), 221–250.

[cl21152-bib-0207] Anthony, J. L. (2016). For which children of economic disadvantage and in which instructional contexts does Earobics Step 1 improve kindergarteners’ literacy? Journal of Research on Educational Effectiveness, 9(1), 54–76.

[cl21152-bib-0208] Baker, D. L. , Park, Y. , Baker, S. K. , Basaraba, D. L. , Kame'enui, E. J. , & Beck, C. T. (2012). Effects of a paired bilingual reading program and an English‐only program on the reading performance of English learners in Grades 1–3. Journal of School Psychology, 50(6), 737–758. 23245498 10.1016/j.jsp.2012.09.002

[cl21152-bib-0209] Baroody, A. J. , Eiland, M. D. , Purpura, D. J. , & Reid, E. E. (2013). Can computer‐assisted discovery learning foster first graders’ fluency with the most basic addition combinations? American Educational Research Journal, 50(3), 533–573.

[cl21152-bib-0210] Berninger, V. W. , Abbott, R. D. , Brooksher, R. , Lemos, Z. , Ogier, S. , Zook, D. , & Mostafapour, E. (2000). A connectionist approach to making the predictability of English orthography explicit to at‐risk beginning readers: Evidence for alternative, effective strategies. Developmental Neuropsychology, 17(2), 241–271. 10955205 10.1207/S15326942DN1702_06

[cl21152-bib-0211] Brailsford, A. , Snart, F. , & Das, J. P. (1984). Strategy training and reading comprehension. Journal of Learning Disabilities, 17(5), 287–290. 6726071 10.1177/002221948401700508

[cl21152-bib-0212] Brown, K. J. , Morris, D. , & Fields, M. (2005). Intervention after grade 1: Serving increased numbers of struggling readers effectively. Journal of Literacy Research, 37(1), 61–94.

[cl21152-bib-0213] Brush, T. A. (1997). The effects of group composition on achievement and time on task for students completing ILS activities in cooperative pairs. Journal of Research on Computing in Education, 30(1), 2–17.

[cl21152-bib-0214] Burns, D. A. (2011). *Examining the effect of an overt transition intervention on the reading development of at‐risk English‐language learners in first grade* (Doctoral dissertation). Available from ProQuest Dissertations and Theses (UMI No. 3466320).

[cl21152-bib-0215] Bussjaeger, J. J. (1993). *The effectiveness of Project Read on the reading achievement of students with learning disabilities* (Master's thesis). Available from ProQuest Dissertations and Theses (UMI No. 1352850).

[cl21152-bib-0216] Cantrell, S. C. , Almasi, J. F. , Rintamaa, M. , Carter, J. C. , Pennington, J. , & Buckman, D. M. (2014). The impact of supplemental instruction on low‐achieving adolescents’ reading engagement. The Journal of Educational Research, 107(1), 36–58.

[cl21152-bib-0217] Caputo, M. T. (2007). *A comparison of the effects of the accelerated math program and the Delaware procedural fluency workbook program on academic growth in grade six at x middle school* (Doctoral dissertation). Available from ProQuest Dissertations and Theses (UMI No. 3283302).

[cl21152-bib-0218] Cook, J. M. , & Welch, M. W. (1980). Reading as a function of visual and auditory process training. Learning Disability Quarterly, 3(3), 76–87.

[cl21152-bib-0219] Cox, D. J. (1997). *The effectiveness of Project Read and visualization and verbalization reading comprehension strategies to improve reading comprehension in at‐risk and learning disabled students* (Doctoral dissertation). Available from ProQuest Dissertations and Theses (UMI No. 1386273).

[cl21152-bib-0220] Coyne, M. D. , McCoach, D. B. , & Kapp, S. (2007). Vocabulary intervention for kindergarten students: Comparing extended instruction to embedded instruction and incidental exposure. Learning Disability Quarterly, 30(2), 74–88.

[cl21152-bib-0221] Coyne, M. D. , Little, M. , Rawlinson, D. , Simmons, D. , Kwok, O. , Kim, M. , Simmons, L. , Hagan‐Burke, S. , & Civetelli, C. (2013). Replicating the impact of a supplemental beginning reading intervention: The role of instructional context. Journal of Research on Educational Effectiveness, 6(1), 1–23.

[cl21152-bib-0222] Coyne, M. D. , Simmons, D. C. , Hagan‐Burke, S. , Simmons, L. E. , Kwok, O. M. , Kim, M. , Fogarty, M. , Oslund, E. L. , Taylor, A. B. , Capozzoli‐Oldham, A. , Ware, S. , Little, M. E. , & Rawlinson, D. M. (2013). Adjusting beginning reading intervention based on student performance: An experimental evaluation. Exceptional Children, 80(1), 25–44.

[cl21152-bib-0223] Doss, D. L. (2015). *The impact of reading interventions on the academic achievement of middle school students* (Doctoral dissertation). Available from ProQuest Dissertations and Theses (UMI No. 3730047).

[cl21152-bib-0224] Duerr, S. A. (2007). *Communication, efficacy, and student reading achievement: An unbroken circle* (Doctoral dissertation). Available from ProQuest Dissertations and Theses (UMI No. 3277505).

[cl21152-bib-0225] Faggella‐Luby, M. , & Wardwell, M. (2011). RTI in a middle school: Findings and practical implications of a tier 2 reading comprehension study. Learning Disability Quarterly, 34(1), 35–49.

[cl21152-bib-0226] Fantuzzo, J. W. , Davis, G. Y. , & Ginsburg, M. D. (1995). Effects of parent involvement in isolation or in combination with peer tutoring on student self‐concept and mathematics achievement. Journal of Educational Psychology, 87(2), 272–281.

[cl21152-bib-0227] Fenty, N. , Mulcahy, C. , & Washburn, E. (2015). Effects of computer‐assisted and teacher‐led fluency instruction on students at risk for reading failure. Learning Disabilities—A Contemporary Journal, 13(2), 141–156.

[cl21152-bib-0228] Fisher, B. , Cozens, M. E. , & Greive, C. (2007). Look‐Say‐Cover‐Write‐Say‐Check and Old Way/New Way‐Mediational Learning: A comparison of the effectiveness of two tutoring programs for children with persistent spelling difficulties. Education papers and Journal Articles. Paper 31.

[cl21152-bib-0229] Foorman, B. R. , Francis, D. J. , Winikates, D. , Mehta, P. , Schatschneider, C. , & Fletcher, J. M. (1997). Early interventions for children with reading disabilities. Scientific Studies of Reading, 1(3), 255–276.

[cl21152-bib-0230] Fryer, R. G., Jr. , Devi, T. , & Holden, R. T. (2016). *Vertical versus horizontal incentives in education: Evidence from randomized trials*. Unpublished manuscript, Harvard University, Cambridge, MA.

[cl21152-bib-0231] Fuchs, L. S. , Fuchs, D. , & Deno, S. L. (1985). Importance of goal ambitiousness and goal mastery to student achievement. Exceptional Children, 52(1), 63–71. 4043188 10.1177/001440298505200108

[cl21152-bib-0232] Fuchs, L. S. , Fuchs, D. , Hamlet, C. L. , Powell, S. R. , Capizzi, A. M. , & Seethaler, P. M. (2006). The effects of computer‐assisted instruction on number combination skill in at‐risk first graders. Journal of Learning Disabilities, 39(5), 467–475. 17004677 10.1177/00222194060390050701

[cl21152-bib-0233] Fuchs, L. S. , Powell, S. R. , Hamlett, C. L. , Fuchs, D. , Cirino, P. T. , & Fletcher, J. M. (2008). Remediating computational deficits at third grade: A randomized field trial. Journal of Research on Educational Effectiveness, 1(1), 2–32. 21709759 10.1080/19345740701692449 PMC3121170

[cl21152-bib-0234] Hendricks, C. , Trueblood, L. , & Pasnak, R. (2006). Effects of teaching patterning to 1st‐graders. Journal of Research in Childhood Education, 21(1), 79–89.

[cl21152-bib-0235] Hill, S. (2009). *An investigation of the impact of asynchronous online learning on student achievement* (Doctoral dissertation). Available from ProQuest Dissertations and Theses (UMI No. 3387212).

[cl21152-bib-0236] Hogan‐Gancarz, C. R. (1999). *Working memory and mathematics: Cognitive learning strategies use with students with learning disabilities* (Doctoral dissertation). Available from ProQuest Dissertations and Theses (UMI No. 9901748).

[cl21152-bib-0237] Holmes, B. C. (1985). The effects of a strategy and sequenced materials on the inferential‐comprehension of disabled readers. Journal of Learning Disabilities, 18(9), 542–546. 4067413 10.1177/002221948501800909

[cl21152-bib-0238] Hudson, R. F. , Isakson, C. , Richman, T. , Lane, H. B. , & Arriaza‐Allen, S. (2011). An examination of a small‐group decoding intervention for struggling readers: Comparing accuracy and automaticity criteria. Learning Disabilities Research & Practice, 26(1), 15–27.

[cl21152-bib-0239] Hunt, J. H. (2014). Effects of a supplemental intervention focused in equivalency concepts for students with varying abilities. Remedial and Special Education, 35(3), 135–144.

[cl21152-bib-0240] Jessup, B. (2017). *A Study of Instructional Methods on Fourth Grade Reading Achievement* (Doctoral dissertation). Available from ProQuest Dissertations and Theses (UMI No. 10638121).

[cl21152-bib-0241] Jitendra, A. K. , Griffin, C. C. , Haria, P. , Leh, J. , Adams, A. , & Kaduvettoor, A. (2007). A comparison of single and multiple strategy instruction on third‐grade students' mathematical problem solving. Journal of Educational Psychology, 99(1), 115–127.

[cl21152-bib-0242] Jitendra, A. K. , Dupuis, D. N. , Rodriguez, M. C. , Zaslofsky, A. F. , Slater, S. , Cozine‐Corroy, K. , & Church, C. (2013). A randomized controlled trial of the impact of schema‐based instruction on mathematical outcomes for third‐grade students with mathematics difficulties. The Elementary School Journal, 114(2), 252–276.

[cl21152-bib-0243] Jitendra, A. K. , Rodriguez, M. , Kanive, R. , Huang, J. P. , Church, C. , Corroy, K. A. , & Zaslofsky, A. (2013). Impact of small‐group tutoring interventions on the mathematical problem solving and achievement of third‐grade students with mathematics difficulties. Learning Disability Quarterly, 36(1), 21–35.

[cl21152-bib-0244] Joiner, S. M. (2012). *Reading Recovery and student achievement* (Doctoral dissertation). Available from ProQuest Dissertations and Theses (UMI No. 3549472).

[cl21152-bib-0245] Kamps, D. , Abbott, M. , Greenwood, C. , Arreaga‐Mayer, C. , Wills, H. , Longstaff, J. , Culpepper, M. , & Walton, C. (2007). Use of evidence‐based, small‐group reading instruction for English language learners in elementary grades: Secondary‐tier intervention. Learning Disability Quarterly, 30(3), 153–168.

[cl21152-bib-0246] Kamps, D. , Abbott, M. , Greenwood, C. , Wills, H. , Veerkamp, M. , & Kaufman, J. (2008). Effects of small‐group reading instruction and curriculum differences for students most at risk in kindergarten: Two‐year results for secondary‐and tertiary‐level interventions. Journal of Learning Disabilities, 41(2), 101–114. 18354931 10.1177/0022219407313412

[cl21152-bib-0247] Kestel, E. , & Forgasz, H. (2018). Targeted tuition delivered by personal videoconferencing for students with Mathematical Learning Difficulties. Learning Disabilities Research & Practice, 33(2), 112–122.

[cl21152-bib-0248] Kim, J. S. , Samson, J. F. , Fitzgerald, R. , & Hartry, A. (2009). A randomized experiment of a mixed‐methods literacy intervention for struggling readers in grades 4–6: Effects on word reading efficiency, reading comprehension and vocabulary, and oral reading fluency. Reading and Writing, 23(9), 1109–1129.

[cl21152-bib-0249] Lalley, J. P. , & Miller, R. H. (2006). Effects of pre‐teaching and re‐teaching on math achievement and academic self‐concept of students with low achievement in math. Education, 126(4), 747–755.

[cl21152-bib-0250] Lamminmäki, T. , Ahonen, T. , de Barra, H. T. , Tolvanen, A. , Michelsson, K. , & Lyytinen, H. (1997). Comparing efficacies of neurocognitive treatment and homework assistance programs for children with learning difficulties. Journal of Learning Disabilities, 30(3), 333–345. 9146099 10.1177/002221949703000308

[cl21152-bib-0251] Leh, J. M. , & Jitendra, A. K. (2012). Effects of computer‐mediated versus teacher‐mediated instruction on the mathematical word problem‐solving performance of third‐grade students with mathematical difficulties. Learning Disability Quarterly, 36(2), 68–79.

[cl21152-bib-0252] Little, M. E. , Rawlinson, D. , Simmons, D. C. , Kim, M. , Kwok, O. , Hagan‐Burke, S. , Simmons, L. E. , Fogarty, M. , Oslund, E. , & Coyne, M. D. (2012). A comparison of responsive interventions on kindergarteners’ early reading achievement. Learning Disabilities Research & Practice, 27(4), 189–202.

[cl21152-bib-0253] Lovett, M. W. , Borden, S. L. , DeLuca, T. , Lacerenza, L. , Benson, N. J. , & Brackstone, D. (1994). Treating the core deficits of developmental dyslexia: Evidence of transfer of learning after phonologically‐ and strategy‐based reading training programs. Developmental Psychology, 30(6), 805–822.

[cl21152-bib-0254] Lovett, M. W. , De Palma, M. , Frijters, J. , Steinbach, K. , Temple, M. , Benson, N. , & Lacerenza, L. (2008). Interventions for reading difficulties: A comparison of response to intervention by ELL and EFL struggling readers. Journal of Learning Disabilities, 41(4), 333–352. 18560021 10.1177/0022219408317859

[cl21152-bib-0255] Lovett, M. W. , Lacerenza, L. , Borden, S. L. , Frijters, J. C. , Steinbach, K. A. , & De Palma, M. (2000). Components of effective remediation for developmental reading disabilities: Combining phonological and strategy‐based instruction to improve outcomes. Journal of Educational Psychology, 92(2), 263–283.

[cl21152-bib-0256] Lovett, M. W. , & Steinbach, K. A. (1997). The effectiveness of remedial programs for reading disabled children of different ages: Does the benefit decrease for older children? Learning Disability Quarterly, 20(3), 189–210.

[cl21152-bib-0257] Lovett, M. W. , Warren‐Chaplin, P. M. , Ransby, M. J. , & Borden, S. L. (1990). Training the word recognition skills of reading disabled children: Treatment and transfer effects. Journal of Educational Psychology, 82(4), 769–780.

[cl21152-bib-0258] Lysynchuk, L. M. , Pressley, M. , & Vye, N. J. (1990). Reciprocal teaching improves standardized reading‐comprehension performance in poor comprehenders. Elementary School Journal, 90(5), 469–484.

[cl21152-bib-0259] Mathes, P. G. , Denton, C. A. , Fletcher, J. M. , Anthony, J. L. , Francis, D. J. , & Schatschneider, C. (2005). The effects of theoretically different instruction and student characteristics on the skills of struggling readers. Reading Research Quarterly, 40(2), 148–182.

[cl21152-bib-0260] McArthur, G. , Castles, A. , Kohnen, S. , Larsen, L. , Jones, K. , Anandakumar, T. , & Banales, E. (2015). Sight word and phonics training in children with dyslexia. Journal of Learning Disabilities, 48(4), 391–407. 24085229 10.1177/0022219413504996

[cl21152-bib-0261] McDermott, P. A. , & Stegemann, J. H. (1987). *The comparative effects of computer‐assisted instruction of motivation and achievement of learning disabled and nonlearning disabled students* (Report). Retrieved from ERIC database (ED309611).

[cl21152-bib-0262] McMaster, K. L. , Fuchs, D. , Fuchs, L. S. , & Compton, D. L. (2005). Responding to nonresponders: An experimental field trial of identification and intervention methods. Exceptional Children, 71(4), 445–463.

[cl21152-bib-0263] Miller, C. (2009). *Main idea identification with students with mild intellectual disabilities/specific learning disabilities: A comparison between an explicit and a basal instructional approach* (Doctoral dissertation). Available from ProQuest Dissertations and Theses (UMI No. 3386215).

[cl21152-bib-0264] Morris, R. D. , Lovett, M. W. , Wolf, M. , Sevcik, R. A. , Steinbach, K. A. , Frijters, J. C. , & Shapiro, M. B. (2012). Multiple‐component remediation for developmental reading disabilities: IQ, socioeconomic status, and race as factors in remedial outcome. Journal of Learning Disabilities, 45(2), 99–127. 20445204 10.1177/0022219409355472 PMC9872281

[cl21152-bib-0265] Morse‐Taylor, B. C. (2010). *The relationship between ability grouping and the reading scores of low achieving 3rd grade students in high poverty urban schools in Georgia* (Doctoral dissertation). Available from ProQuest Dissertations and Theses (UMI No. 3454826).

[cl21152-bib-0266] Nash, H. , & Snowling, M. (2006). Teaching new words to children with poor existing vocabulary knowledge: A controlled evaluation of the definition and context methods. International Journal of Language & Communication Disorders, 41(3), 335–354. 16702097 10.1080/13682820600602295

[cl21152-bib-0267] O'Connor, E. E. , Cappella, E. , McCormick, M. P. , & McClowry, S. G. (2014). An examination of the efficacy of INSIGHTS in enhancing the academic and behavioral development of children in early grades. Journal of Educational Psychology, 106(4), 1156–1169.

[cl21152-bib-0268] O'Connor, E. E. , Cappella, E. , McCormick, M. P. , & McClowry, S. G. (2014). Enhancing the academic development of shy children: A test of the efficacy of INSIGHTS. School Psychology Review, 43(3), 239–259.

[cl21152-bib-0269] Olympia, D. E. , Sheridan, S. M. , Jenson, W. R. , & Andrews, D. (1994). Using student‐managed interventions to increase homework completion and accuracy. Journal of Applied Behavior Analysis, 27(1), 85–99. 16795827 10.1901/jaba.1994.27-85 PMC1297779

[cl21152-bib-0270] O'Connor, R. E. , Fulmer, D. , Harty, K. R. , & Bell, K. M. (2005). Layers of reading intervention in kindergarten through third grade: Changes in teaching and student outcomes. Journal of Learning Disabilities, 38(5), 440–455. 16329445 10.1177/00222194050380050701

[cl21152-bib-0271] Oudeans, M. K. (2003). Integration of letter‐sound correspondences and phonological awareness skills of blending and segmenting: A pilot study examining the effects of instructional sequence on word reading for kindergarten children with low phonological awareness. Learning Disability Quarterly, 26(4), 258–280.

[cl21152-bib-0272] Parrila, R. K. , Das, J. P. , Kendrick, M. E. , Papadopoulos, T. C. , & Kirby, J. R. (1999). Efficacy of a cognitive reading remediation program for at‐risk children in grade 1. Developmental Disabilities Bulletin, 27(2), 1–31.

[cl21152-bib-0273] Pascarella, E. T. , Pflaum, S. W. , Bryan, T. H. , & Pearl, R. A. (1983). Interaction of internal attribution for effort and teacher response mode in reading instruction: A replication note. American Educational Research Journal, 20(2), 269–276.

[cl21152-bib-0274] Pasnak, R. , Kidd, J. K. , Gadzichowski, K. M. , Gallington, D. A. , Schmerold, K. L. , & West, H. (2015). Abstracting sequences: Reasoning that is a key to academic achievement. Journal of Genetic Psychology, 176(3), 171–193. 26135563 10.1080/00221325.2015.1024198

[cl21152-bib-0275] Pavchinski, P. (1988). *The effects of operant procedures and cognitive behavior modification on learning disabled students’ math skills* (Doctoral dissertation). Gainesville, FL: University of Florida.

[cl21152-bib-0276] Pflaum, S. W. , Pascarella, E. T. , Auer, C. , Augustyn, L. , & Boswick, M. (1982). Differential effects of four comprehension‐facilitating conditions on LD and normal elementary‐school readers. Learning Disability Quarterly, 5(2), 106–116.

[cl21152-bib-0277] Pinnell, G. S. (1988). *Success of children at risk in a program that combines writing and reading* (Technical Report no. 417). Center for the Study of Reading, University of Illinois, Urbana‐Champaign.

[cl21152-bib-0278] Pinnell, G. S. , Deford, D. E. , & Lyons, C. A. (1988). *Reading recovery: Early intervention for at‐risk first graders* (ERS Monograph). Arlington, VA: Educational Research Services. Retrieved from ERIC database (ED303790).

[cl21152-bib-0279] Reis, S. M. , McCoach, D. B. , Little, C. A. , Muller, L. M. , & Kaniskan, R. B. (2011). The effects of differentiated instruction and enrichment pedagogy on reading achievement in five elementary schools. American Educational Research Journal, 48(2), 462–501.

[cl21152-bib-0280] Reutzel, D. R. , Petscher, Y. , & Spichtig, A. N. (2012). Exploring the value added of a guided, silent reading intervention: Effects on struggling third‐grade readers’ achievement. Journal of Educational Research, 105(6), 404–415. 26346539 10.1080/00220671.2011.629693 PMC4557881

[cl21152-bib-0281] Ritchey, K. D. , Silverman, R. D. , Montanaro, E. A. , Speece, D. L. , & Schatschneider, C. (2012). Effects of a tier 2 supplemental reading intervention for at‐risk fourth‐grade students. Exceptional Children, 78(3), 318–334. 22685347 10.1177/001440291207800304 PMC3370413

[cl21152-bib-0282] Ruggiero Palombo, P. (2004). *All learners in one classroom: The impact of partial inclusion on elementary students' academic achievement, attitudes, and perceptions* (Doctoral dissertation). Available from ProQuest Dissertations and Theses (UMI No. 3134447).

[cl21152-bib-0283] Russell, T. , & Ford, D. F. (1983). Effectiveness of peer tutors vs. resource teachers. Psychology in the Schools, 20(4), 436–441.

[cl21152-bib-0284] Rzoska, K. M. , & Ward, C. (1991). The effects of cooperative and competitive learning methods on the mathematics achievement, attitudes toward school, self‐concepts and friendship choices of Maori, Pakeha and Samoan children. New Zealand Journal of Psychology, 20(1), 17–24.

[cl21152-bib-0285] Saine, N. L. , Lerkkanen, M. K. , Ahonen, T. , Tolvanen, A. , & Lyytinen, H. (2011). Computer‐assisted remedial reading intervention for school beginners at risk for reading disability. Child Development, 82(3), 1013–1028. 21418055 10.1111/j.1467-8624.2011.01580.x

[cl21152-bib-0286] Saine, N. L. , Lerkkanen, M. K. , Ahonen, T. , Tolvanen, A. , & Lyytinen, H. (2013). Long‐term intervention effects of spelling development for children with compromised preliteracy skills. Reading & Writing Quarterly, 29(4), 333–357.

[cl21152-bib-0287] Santoro, L. E. , Coyne, M. D. , & Simmons, D. C. (2006). The reading–spelling connection: Developing and evaluating a beginning spelling intervention for children at risk of reading disability. Learning Disabilities Research & Practice, 21(2), 122–133.

[cl21152-bib-0288] Simmons, D. C. , Coyne, M. D. , Hagan‐Burke, S. , Kwok, O. M. , Simmons, L. , Johnson, C. , Zou, Y. , Taylor, A. B. , Mcalenney, A. L. , Ruby, M. , & Crevecoeur, Y. C. (2011). Effects of supplemental reading interventions in authentic contexts: A comparison of kindergarteners’ response. Exceptional Children, 77(2), 207–228.

[cl21152-bib-0289] Simmons, D. C. , Kame'enui, E. J. , Harn, B. , Coyne, M. D. , Stoolmiller, M. , Edwards Santoro, L. , Smith, S. B. , Beck, C. T. , & Kaufman, N. K. (2007). Attributes of effective and efficient kindergarten reading intervention: An examination of instructional time and design specificity. Journal of Learning Disabilities, 40(4), 331–347. 17713132 10.1177/00222194070400040401

[cl21152-bib-0290] Simmons, D. C. , Kim, M. , Kwok, O. , Coyne, M. D. , Simmons, L. E. , Oslund, E. , Fogarty, M. , Hagan‐Burke, S. , Little, M. E. , & Rawlinson, D. (2015). Examining the effects of linking student performance and progression in a Tier 2 kindergarten reading intervention. Journal of Learning Disabilities, 48(3), 255–270. 23907163 10.1177/0022219413497097

[cl21152-bib-0291] Slavin, R. E. , Madden, N. , Calderón, M. , Chamberlain, A. , & Hennessy, M. (2011). Reading and language outcomes of a multiyear randomized evaluation of transitional bilingual education. Educational Evaluation and Policy Analysis, 33(1), 47–58.

[cl21152-bib-0292] Smith‐Davis, S. (2007). *Does success for all impact reading achievement of students with learning disabilities?* (Doctoral dissertation). Available from ProQuest Dissertations and Theses (UMI No. 3276390).

[cl21152-bib-0293] Soltero‐González, L. , Sparrow, W. , Butvilofsky, S. , Escamilla, K. , & Hopewell, S. (2016). Effects of a paired literacy program on emerging bilingual children's biliteracy outcomes in third grade. Journal of Literacy Research, 48(1), 80–104.

[cl21152-bib-0294] Spies, T. G. , Lara‐Alecio, R. , Tong, F. , Irby, B. J. , Garza, T. , & Huerta, M. (2018). The effects of developing English language and literacy on Spanish reading comprehension. The Journal of Educational Research, 111(5), 517–529.

[cl21152-bib-0295] Tong, F. , Irby, B. J. , Lara‐Alecio, R. , & Mathes, P. G. (2008). English and Spanish acquisition by Hispanic second graders in developmental bilingual programs: A 3‐year longitudinal randomized study. Hispanic Journal of Behavioral Sciences, 30(4), 500–529.

[cl21152-bib-0296] Tong, F. , Lara‐Alecio, R. , Irby, B. , Mathes, P. , & Kwok, O. M. (2008). Accelerating early academic oral English development in transitional bilingual and structured English immersion programs. American Educational Research Journal, 45(4), 1011–1044.

[cl21152-bib-0297] Torgesen, J. K. , Alexander, A. W. , Wagner, R. K. , Rashotte, C. A. , Voeller, K. K. , & Conway, T. (2001). Intensive remedial instruction for children with severe reading disabilities: Immediate and long‐term outcomes from two instructional approaches. Journal of Learning Disabilities, 34(1), 33–58. 15497271 10.1177/002221940103400104

[cl21152-bib-0298] Torgesen, J. K. , Wagner, R. K. , Rashotte, C. A. , & Herron, J. (2003). Summary of outcomes from first grade study with “Read, Write, and Type” and “Auditory Discrimination in Depth” instruction and software with at‐risk children (Technical Report; no. 2, Florida Center for Reading Research). Tallahassee, FL: Department of Psychology, Florida State University.

[cl21152-bib-0299] Tremblay, P. (2013). Comparative outcomes of two instructional models for students with learning disabilities: inclusion with co‐teaching and solo‐taught special education. Journal of Research in Special Educational Needs, 13(4), 251–258.

[cl21152-bib-0300] Wade, J. , & Kass, C. E. (1986). Component deficit and academic remediation of learning disabilities. Journal of Learning Disabilities, 19(1), 23–25. 10.1177/002221948702000714 3655555

[cl21152-bib-0301] Wages, M. M. (2013). *A comparison of two bilingual programs on student reading achievement in a public elementary school in Texas* (Doctoral dissertation). Available from ProQuest Dissertations and Theses (UMI No. 3557443).

[cl21152-bib-0302] Wang, M. H. , Grandieri, C. , Adley, A. R. , & Pence, K. (2016). *Evaluation of middle school reading interventions programs using student growth on Gates‐MacGinitie Reading Test*. Paper presented at the 2016 annual meeting of the American Educational Research Association. Retrieved from the AERA Online Paper Repository.

[cl21152-bib-0303] Wanzek, J. , & Vaughn, S. (2008). Response to varying amounts of time in reading intervention for students with low response to intervention. Journal of Learning Disabilities, 41(2), 126–142. 18354933 10.1177/0022219407313426 PMC3322477

[cl21152-bib-0304] Wesson, C. (1983). *Two student self‐management techniques applied to data‐based program modification* (Technical Report; no. 143). Minneapolis, MN: Minneapolis Institute for Research on Learning Disabilities, Minnesota University.

[cl21152-bib-0305] Wilson, B. N. , & Kaplan, B. J. (1994). Follow‐up assessment of children receiving sensory integration treatment. Occupational Therapy Journal of Research, 14(4), 244–266.

[cl21152-bib-0306] Wise, B. W. , Ring, J. , Sessions, L. , & Olson, R. K. (1997). Phonological awareness with and without articulation: A preliminary study. Learning Disability Quarterly, 20(3), 211–225.

[cl21152-bib-0307] Wise, J. C. (2005). *The growth of phonological awareness: Response to reading intervention by children with reading disabilities who exhibit typical or below‐average language skills* (Doctoral dissertation). Available from ProQuest Dissertations and Theses (UMI No. 3180049).

[cl21152-bib-0308] Woodward, J. (2006). Developing automaticity in multiplication facts: Integrating strategy instruction with timed practice drills. Learning Disability Quarterly, 29(4), 269–289.

[cl21152-bib-0309] Xin, J. F. (1999). Computer‐assisted cooperative learning in integrated classrooms for students with and without disabilities. Information Technology in Childhood Education Annual, 1999(10), 61–78.

[cl21152-bib-0310] Xin, Y. P. , Zhang, D. , Park, J. Y. , Tom, K. , Whipple, A. , & Si, L. (2011). A comparison of two mathematics problem‐solving strategies: Facilitate algebra‐readiness. Journal of Educational Research, 104(6), 381–395.

[cl21152-bib-0311] Baker, S. K. , Smolkowski, K. , Chaparro, E. A. , Smith, J. L. , & Fien, H. (2015). Using regression discontinuity to test the impact of a tier 2 reading intervention in first grade. Journal of Research on Educational Effectiveness, 8(2), 218–244.

[cl21152-bib-0312] Chaparro, E. A. , Smolkowski, K. , Baker, S. K. , Fien, H. , & Smith, J. L. M. (2012). *An examination of treatment effects of a first grade literacy intervention using a regression discontinuity design* (Evaluative Report). Society for Research on Educational Effectiveness. Retrieved from ERIC database (ED530445).

[cl21152-bib-0313] Fien, H. , Smith, J. L. M. , Smolkowski, K. , Baker, S. K. , Nelson, N. J. , & Chaparro, E. (2015). An examination of the efficacy of a multitiered intervention on early reading outcomes for first grade students at risk for reading difficulties. Journal of Learning Disabilities, 48(6), 602–621. 24532827 10.1177/0022219414521664

[cl21152-bib-0314] Fives, A. (2016). Modeling the interaction of academic self‐beliefs, frequency of reading at home, emotional support, and reading achievement: An RCT study of at‐risk early readers in first grade and second grade. Reading Psychology, 37(3), 339–370.

[cl21152-bib-0315] Foster, M. (2014). *Structure of mathematics achievement and response to intervention in children with mild disabilities* (Doctoral dissertation). Available from ProQuest Dissertations and Theses (UMI No. 3637676).

[cl21152-bib-0316] Fuchs, D. , Fuchs, L. S. , Thompson, A. , Otaiba, S. A. , Yen, L. , Yang, N. J. , Braun, M. , & O'Connor, R. E. (2002). Exploring the importance of reading programs for kindergartners with disabilities in mainstream classrooms. Exceptional Children, 68(3), 295–311.

[cl21152-bib-0317] Fuchs, L. S. , Fuchs, D. , Compton, D. L. , Wehby, J. , Schumacher, R. F. , Gersten, R. , & Jordan, N. C. (2015). Inclusion versus specialized intervention for very‐low‐performing students: What does access mean in an era of academic challenge? Exceptional Children, 81(2), 134–157.

[cl21152-bib-0318] Gunn, B. , Biglan, A. , Smolkowski, K. , & Ary, D. (2000). The efficacy of supplemental instruction in decoding skills for Hispanic and non‐Hispanic students in early elementary school. Journal of Special Education, 34(2), 90–103.

[cl21152-bib-0319] Gunn, B. , Smolkowski, K. , Biglan, A. , & Black, C. (2002). Supplemental instruction in decoding skills for Hispanic and non‐Hispanic students in early elementary school: A follow‐up. Journal of Special Education, 36(2), 69–79. 10.1177/00224669050390020301 PMC1828030 17364009

[cl21152-bib-0320] Hagan‐Burke, S. , Coyne, M. D. , Kwok, O. , Simmons, D. C. , Kim, M. , Simmons, L. E. , Skidmore, S. T. , Hernandez, C. L. , & McSparran Ruby, M. (2013). The effects and interactions of student, teacher, and setting variables on reading outcomes for kindergarteners receiving supplemental reading intervention. Journal of Learning Disabilities, 46(3), 260–277. 21940462 10.1177/0022219411420571

[cl21152-bib-0321] Kamps, D. M. , & Greenwood, C. R. (2005). Formulating secondary‐level reading interventions. Journal of Learning Disabilities, 38(6), 500–509. 16392691 10.1177/00222194050380060501

[cl21152-bib-0322] Linan‐Thompson, S. , Cirino, P. T. , & Vaughn, S. (2007). Determining English language learners' response to intervention: Questions and some answers. Learning Disability Quarterly, 30(3), 185–195.

[cl21152-bib-0323] Linan‐Thompson, S. , Vaughn, S. , Prater, K. , & Cirino, P. T. (2006). The response to intervention of English language learners at risk for reading problems. Journal of Learning Disabilities, 39(5), 390–398. 17004672 10.1177/00222194060390050201

[cl21152-bib-0324] Powell, S. R. , Fuchs, L. S. , Cirino, P. T. , Fuchs, D. , Compton, D. L. , & Changas, P. C. (2015). Effects of a multitier support system on calculation, word problem, and prealgebraic performance among at‐risk learners. Exceptional Children, 81(4), 443–470. 26097244 10.1177/0014402914563702 PMC4470425

[cl21152-bib-0325] Sáenz, L. M. , Fuchs, L. S. , & Fuchs, D. (2005). Peer‐assisted learning strategies for English language learners with learning disabilities. Exceptional Children, 71(3), 231–247.

[cl21152-bib-0326] Tong, F. , Lara‐Alecio, R. , Irby, B. J. , & Mathes, P. G. (2011). The effects of an instructional intervention on dual language development among first‐grade Hispanic English‐learning boys and girls: A two‐year longitudinal study. Journal of Educational Research, 104(2), 87–99.

[cl21152-bib-0327] Wanzek, J. , Vaughn, S. , Roberts, G. , & Fletcher, J. M. (2011). Efficacy of a reading intervention for middle school students with learning disabilities. Exceptional Children, 78(1), 73–87. 22485052 10.1177/001440291107800105 PMC3324302

[cl21152-bib-0328] Babinski, L. M. , Amendum, S. J. , Knotek, S. E. , Sánchez, M. , & Malone, P. (2018). Improving young English learners’ language and literacy skills through teacher professional development: A randomized controlled trial. American Educational Research Journal, 55(1), 117–143.

[cl21152-bib-0329] Baenen, N. , Bernholc, A. , Dulaney, C. , & Banks, K. (1997). Reading Recovery: Long‐term progress after three cohorts. Journal of Education for Students Placed at Risk, 2(2), 161–181.

[cl21152-bib-0330] Boardman, A. G. , Vaughn, S. , Buckley, P. , Reutebuch, C. , Roberts, G. , & Klingner, J. (2016). Collaborative strategic reading for students with learning disabilities in upper elementary classrooms. Exceptional Children, 82(4), 409–427.

[cl21152-bib-0331] Bramlett, R. K. (1992). *Cooperative learning: A field study with implications for school psychologists*. Paper presented at the Annual Convention of the American Psychological Association, Washington DC. Retrieved from ERIC database (ED351654).

[cl21152-bib-0332] Carlo, M. S. , August, D. , McLaughlin, B. , Snow, C. E. , Dressler, C. , Lippman, D. N. , Lively, T. J. , & White, C. E. (2004). Closing the gap: Addressing the vocabulary needs of English‐language learners in bilingual and mainstream classrooms. Reading Research Quarterly, 39(2), 188–215.

[cl21152-bib-0333] Carter, J. L. , & Russell, H. L. (1985). Use of EMG biofeedback procedures with learning disabled children in a clinical and an educational setting. Journal of Learning Disabilities, 18(4), 213–216. 3886819 10.1177/002221948501800406

[cl21152-bib-0334] Chiang, H. Y. (2007). *Effects and users’ perceptions of computer‐based instruction* (Doctoral dissertation). Available from ProQuest Dissertations and Theses (UMI No. 3259811).

[cl21152-bib-0335] Chin, A. , Daysal, N. M. , & Imberman, S. A. (2012). Impact of bilingual education programs on limited English proficient students and their peers: Regression discontinuity evidence from Texas (Working Paper; no. 18197). National Bureau of Economic Research, Massachusetts.

[cl21152-bib-0336] Chodkiewicz, A. R. , & Boyle, C. (2016). Promoting positive learning in Australian students aged 10‐to 12‐years‐old using attribution retraining and cognitive behavioral therapy: A pilot study. School Psychology International, 37(5), 519–535.

[cl21152-bib-0337] Cobb, J. B. (2001). The effects of an early intervention program with preservice teachers as tutors on the reading achievement of primary grade at risk children. Reading Horizons, 41(3), 3.

[cl21152-bib-0338] Glassman, P. (1988). *A study of cooperative learning in mathematics, writing and reading as implemented in third, fourth and fifth grade classes: A focus upon achievement, attitudes and self‐esteem for males, females, blacks, Hispanics and Anglos*. Paper presented at the Annual Meeting of the American Educational Research Association, New Orleans, LA. Retrieved from ERIC database (ED294926).

[cl21152-bib-0339] Gottshall, D. L. (2007). Gottshall Early Reading Intervention: A phonics based approach to enhance the achievement of low performing, rural, first grade boys. University of North Texas.

[cl21152-bib-0340] Greenwood, C. R. (1991). Longitudinal analysis of time, engagement, and achievement in at‐risk versus non‐risk students. Exceptional Children, 57(6), 521–535. 2070811 10.1177/001440299105700606

[cl21152-bib-0341] Hummel, W. , & Hahn, R. (1982). *A model program in microcomputer utilization with handicapped students. Final performance report* (Technical Report; no. 143). Retrieved from ERIC database (ED244493).

[cl21152-bib-0342] Mackey, A. P. , Park, A. T. , Robinson, S. T. , & Gabrieli, J. D. (2017). A pilot study of classroom‐based cognitive skill instruction: Effects on cognition and academic performance. Mind, Brain, and Education, 11(2), 85–95.

[cl21152-bib-0343] Miller, S. , & Connolly, P. (2013). A randomized controlled trial evaluation of time to read, a volunteer tutoring program for 8‐to 9‐year‐olds. Educational Evaluation and Policy Analysis, 35(1), 23–37.

[cl21152-bib-0344] Peña, N. A. P. (2008). *The power of reading: A multilevel study of the longitudinal effect of a paired intergenerational reading aloud program on academically at‐risk elementary students’ reading attitudes, reading motivation and academic achievement* (Doctoral dissertation). Available from ProQuest Dissertations and Theses (UMI No. 3340176).

[cl21152-bib-0345] Scientific Learning Corporation . (2006). Improved reading skills by students who used Fast ForWord® to Reading Prep. MAPS for learning: Product Reports, 10(1), 1–6.

[cl21152-bib-0346] Stavros, D. (1989). *Evaluation of the instrumental enrichment project. 1988‐89* (Evaluative Report; no. 142). Retrieved from ERIC database (ED331936).

[cl21152-bib-0347] Taub, G. E. , McGrew, K. S. , & Keith, T. Z. (2007). Improvements in interval time tracking and effects on reading achievement. Psychology in the Schools, 44(8), 849–863.

[cl21152-bib-0348] Taub, G. E. , McGrew, K. S. , & Keith, T. Z. (2015). Effects of improvements in interval timing on the mathematics achievement of elementary school students. Journal of Research in Childhood Education, 29(3), 352–366.

[cl21152-bib-0349] Woods, S. J. (1986). Hypnosis as a means of achieving cognitive modification in the treatment of academic anxiety: III. Australian Journal of Clinical Hypnotherapy and Hypnosis, 7(2, Eratum), 106–121.

[cl21152-bib-0350] Ysseldyke, J. , Spicuzza, R. , Kosciolek, S. , Teelucksingh, E. , Boys, C. , & Lemkuil, A. (2003). Using a curriculum‐based instructional management system to enhance math achievement in urban schools. Journal of Education for Students Placed at Risk, 8(2), 247–265.

[cl21152-bib-0351] Zunker, L. J. (2008). *Computer‐based instruction and mathematics skills of elementary students with learning disabilities* (Doctoral dissertation). Available from ProQuest Dissertations and Theses (UMI No. 3332683).

[cl21152-bib-0352] Aagaard, L. J. , Roach, R. G. , Wall, T. , & Stamm, V. (2016). An evaluation of a math response to intervention training program in the primary grades. Paper presented at the 2016 annual meeting of the American Educational Research Association, Washington DC. Retrieved from the AERA Online Paper Repository.

[cl21152-bib-0353] Adams, W. (2011). *Comparative study of reading first schools reading achievement to non‐reading first schools* (Doctoral dissertation). Available from ProQuest Dissertations and Theses (UMI No. 3462040).

[cl21152-bib-0354] Algozzine, B. , Marr, M. B. , Kavel, R. L. , & Dugan, K. K. (2009). Using peer coaches to build oral reading fluency. Journal of Education for Students Placed at Risk, 14(3), 256–270.

[cl21152-bib-0355] Allen, K. (2017). An Evaluation Study of Fifth‐Grade Independent Reading and Reading Achievement (Doctoral dissertation). *Dissertation Abstracts International Section A: Humanities and Social Sciences, 4*‐A(E).

[cl21152-bib-0356] Allinder, R. M. , Bolling, R. M. , Oats, R. G. , & Gagnon, W. A. (2000). Effects of teacher self‐monitoring on implementation of curriculum‐based measurement and mathematics computation achievement of students with disabilities. Remedial and Special Education, 21(4), 219–226.

[cl21152-bib-0357] Anderson, V. , Chan, C. K. , & Henne, R. (1995). The effects of strategy instruction on the literacy models of reading and writing delayed middle school students. In *Perspectives on literacy research and practice: Forty‐forth yearbook of the National Reading Conference*. National Reading Conference.

[cl21152-bib-0358] Arnbak, E. , & Elbro, C. (2000). The effects of morphological awareness training on the reading and spelling skills of young dyslexics. Scandinavian Journal of Educational Research, 44(3), 229–251.

[cl21152-bib-0359] Arnold, K. (2013). *A comparison of Georgia criterion referenced competency test math scores between at‐risk fifth grade students receiving computer based math instruction and at‐risk students not receiving computer based math instruction* (Doctoral dissertation). Liberty University.

[cl21152-bib-0360] Arnold, T. F. (2009). *The effects of early reading interventions on student reading levels and achievement* (Doctoral dissertation). Lindenwood University.

[cl21152-bib-0361] Banerji, M. (1988). Longitudinal effects of retention and promotion in kindergarten on academic achievement. Florida Journal of Educational Research, 30(1), 59–72.

[cl21152-bib-0362] Barrett, D. C. (2010). *Using chess to improve math achievement for students who receive special education services* (Doctoral dissertation). Available from ProQuest LLC (UMI No. 3445877).

[cl21152-bib-0363] Bartik, T. , & Lachowska, M. (2014 *). The effects of doubling instruction efforts on middle school students' achievement: Evidence from a multiyear regression‐discontinuity design* (Upjohn Institute Working Paper No. 14‐205). Retrieved from ERIC database (ED559204).

[cl21152-bib-0364] Bass, G. , Ries, R. , & Sharpe, W. (1986). Teaching basic skills through microcomputer assisted instruction. Journal of Educational Computing Research, 2(2), 207–219.

[cl21152-bib-0365] Bates, C. C. , D'Agostino, J. V. , Gambrell, L. , & Xu, M. (2016). Reading Recovery: Exploring the effects on first‐graders' reading motivation and achievement. Journal of Education for Students Placed at Risk, 21(1), 47–59.

[cl21152-bib-0366] Bauer, C. (2013). *Impact of an extended‐day kindergarten intervention on school‐related variables: A longitudinal study* (Doctoral dissertation). State University of New York at Albany. Available from ProQuest LLC (UMI No. 3588505).

[cl21152-bib-0367] Becker, H. J. (1990). *Effects of computer use on mathematics achievement. Findings from a nationwide field experiment in grades five to eight. Classes: Rationale, study design, and aggregate effect sizes* (Report No. 51). Washington DC: Office of Educational Research and Improvement.

[cl21152-bib-0368] Becker, W. C. , & Gersten, R. (1982). *A follow‐up of Follow Through: The later effects of the Direct Instruction Model on children in fifth and sixth grades*. Paper presented at the Biennial Meeting of the Society for Research in Child Development, Boston. Retrieved from ERIC database (ED202601).

[cl21152-bib-0369] Bell, J. N. (2008). *The effects of three reading intervention programs on third grade students' reading achievement* (Doctoral dissertation). Available from ProQuest LLC (UMI No. 3313256).

[cl21152-bib-0370] Bellert, A. (2009). Narrowing the gap: A report on the QuickSmart mathematics intervention. Australian Journal of Learning Difficulties, 14(2), 171–183.

[cl21152-bib-0371] Biemiller, A. , & Siegel, L. S. (1997). A longitudinal study of the effects of the BRIDGE reading program for children at risk for reading failure. Learning Disability Quarterly, 20(2), 83–92.

[cl21152-bib-0372] Black, K. (2010). *Closing the achievement gap: Impact of inclusion upon achievement rates of students with special needs* (Doctoral dissertation). Available from ProQuest Dissertations and Theses (UMI No. 3426841).

[cl21152-bib-0373] Bøg, M. , Dietrichson, J. , & Isaksson, A. A. (2018). *A multi‐sensory literacy program for at‐risk students in kindergarten ‐ Promising results from a small‐scale Swedish intervention*. Unpublished Manuscript.

[cl21152-bib-0374] Boges, C. E. (2015). *The effects of differentiated instruction on the achievement scores of struggling fourth grade readers* (Doctoral dissertation). Available from ProQuest Information and Learning (UMI No. 3671229).

[cl21152-bib-0375] Bonnville, J. K. (2013). *The impact of two response‐to‐intervention tier 2 literacy programs on middle level achievement* (Doctoral dissertation). Retrieved from ERIC database (ED555386).

[cl21152-bib-0376] Bowey, J. A. , & Hansen, J. (1994). The development of orthographic rimes as units of word recognition. Journal of Experimental Child Psychology, 58(3), 465–488.

[cl21152-bib-0377] Brady, S. , Fowler, A. , Stone, B. , & Winbury, N. (1994). Training phonological awareness: A study with inner‐city kindergarten children. Annals of Dyslexia, 44(1), 26–59. 24234045 10.1007/BF02648154

[cl21152-bib-0378] Brand‐Gruwel, S. , Aarnoutse, C. A. J. , & Van Den Bos, K. P. (1998). Improving text comprehension strategies in reading and listening settings. Learning and Instruction, 8(1), 63–81.

[cl21152-bib-0379] Bribiescas, S. M. (2011). *Arizona instrument to measure standards: A comparison of ELL students of teaming and non‐teaming schools* (Doctoral dissertation). Northern Arizona University.

[cl21152-bib-0380] Brigman, G. , & Campbell, C. (2003). Helping students improve academic achievement and school success behavior. Professional School Counseling, 7(2), 91–98.

[cl21152-bib-0381] Brigman, G. , Webb, L. , & Campbell, C. (2007). Building skills for school success: Improving the academic and social competence of students. Professional School Counseling, 10(3), 279–288.

[cl21152-bib-0382] Brown, I. S. , & Felton, R. H. (1990). Effects of instruction on beginning reading skills in children at risk for reading disability. Reading and Writing, 2(3), 223–241.

[cl21152-bib-0383] Brunson, K. M. (2016). *An investigation of the impact of Georgia's early intervention program on at‐risk student reading achievement* (Doctoral dissertation). Available from ProQuest LLC. (UMI No. 10106251).

[cl21152-bib-0384] Bryant, D. P. , Bryant, B. R. , Gersten, R. M. , Scammacca, N. N. , & Chavez, M. M. (2008). Mathematics intervention for first‐and second‐grade students with mathematics difficulties: The effects of tier 2 intervention delivered as booster lessons. Remedial and Special Education, 29(1), 20–32.

[cl21152-bib-0385] Bryant, D. P. , Bryant, B. R. , Gersten, R. M. , Scammacca, N. N. , Funk, C. , Winter, A. , Shih, M. , & Pool, C. (2008). The effects of tier 2 intervention on the mathematics performance of first‐grade students who are at risk for mathematics difficulties. Learning Disability Quarterly, 31(2), 47–63.

[cl21152-bib-0386] Bunn, T. K. (2006). *The effectiveness of additional interventions for children with literacy difficulties in Years 3 & 4* (Doctoral dissertation). University of Warwick.

[cl21152-bib-0387] Burns, M. K. , Kanive, R. , & DeGrande, M. (2012). Effect of a computer‐delivered math fact intervention as a supplemental intervention for math in third and fourth grades. Remedial and Special Education, 33(3), 184–191.

[cl21152-bib-0388] Burton, S. (2005). *The effects of ability grouping on academic gains of rural elementary school students* (Doctoral dissertation). Available from ProQuest Dissertations and Theses (UMI No. 3170198).

[cl21152-bib-0389] Calhoon, M. B. (2005). Effects of a peer‐mediated phonological skill and reading comprehension program on reading skill acquisition for middle school students with reading disabilities. Journal of Learning Disabilities, 38(5), 424–433. 16329443 10.1177/00222194050380050501

[cl21152-bib-0390] Calhoun, V. L. (2007). *The effects of a supplemental program on the reading achievement of learning‐disabled students* (Doctoral dissertation). Available from ProQuest Dissertations and Theses (UMI No. 3262897).

[cl21152-bib-0391] Campbell, D. T. (2000). *The current structure of intellect remediation lab as an intervention for deficient readers in grades 3, 4, and 5* (Doctoral dissertation). Available from ProQuest Dissertations and Theses (UMI No. 9979880).

[cl21152-bib-0392] Cardelle‐Elawar, M. (1992). Effects of teaching metacognitive skills to students with low mathematics ability. Teaching and Teacher Education, 8(2), 109–121.

[cl21152-bib-0393] Cartelli, L. M. (1980). Reading comprehension: A matter of referents. Academic Therapy, 15(4), 421–430.

[cl21152-bib-0394] Cazabon, M. (1993). *Two‐way bilingual education: A progress report on the Amigos program* (Research Report: 7). Retrieved from ERIC database (ED359787).

[cl21152-bib-0395] Center, Y. , Wheldall, K. , Freeman, L. , Outhred, L. , & McNaught, M. (1995). An evaluation of reading recovery. Reading Research Quarterly, 30(2), 240–263.

[cl21152-bib-0396] Cesa, C. A. (2012). *Fifth‐grade readers’ use of metacognitive strategies to comprehend social studies nonnarrative texts* (Doctoral dissertation). Available from ProQuest Dissertations and Theses (UMI No. 3495553).

[cl21152-bib-0397] Chappell, S. , Arnold, P. , Nunnery, J. , & Grant, M. (2015). An examination of an online tutoring program's impact on low‐achieving middle school students’ mathematics achievement. Online Learning, 19(5), 37–53.

[cl21152-bib-0398] Cho, E. , Roberts, G. J. , Capin, P. , Roberts, G. , Miciak, J. , & Vaughn, S. (2015). Cognitive Attributes, Attention, and Self‐Efficacy of Adequate and Inadequate Responders in a Fourth Grade Reading Intervention. Learning Disabilities Research & Practice, 30(4), 159–170. 26997755 10.1111/ldrp.12088 PMC4793275

[cl21152-bib-0399] Choi, S. , & Lemberger, M. E. (2010). Influence of a supervised mentoring program on the achievement of low‐income South Korean students. Mentoring & Tutoring: Partnership in Learning, 18(3), 233–248.

[cl21152-bib-0400] Clark, T. (2017). *The K‐12 service‐learning standards and fourth grade students' math achievement: A quasi‐experimental study in Georgia* (Doctoral dissertation). Available from ProQuest LLC. (UMI No. 10743197).

[cl21152-bib-0401] Claus, R. N. , & Quimper, B. E. (1987). *Long‐term continuous and sustained effects of chapter 1 participation 1983‐1985. Evaluation report*. Retrieved from ERIC database (ED291855).

[cl21152-bib-0402] Clipson‐Boyles, S. (2000). The Catch Up Project: A reading intervention in Year 3 for Level 1 readers. Journal of Research in Reading, 23(1), 78–84.

[cl21152-bib-0403] Colamarino, G. (2008). *The impact of ability grouping on the academic growth of at‐risk students* (Doctoral dissertation). Available from ProQuest Dissertations and Theses (UMI No. 3336656).

[cl21152-bib-0404] Commeyras, M. (1992). *Dialogical‐thinking reading lessons: promoting critical thinking among learning‐disabled students* (Reading Technical Report; no. 553). Retrieved from ERIC database (ED343115).

[cl21152-bib-0405] Conring, J. M. (2009). *The effects of cooperative learning on mathematic achievement in second graders* (Doctoral dissertation). Retrieved from ProQuest Dissertations and Theses (UMI No. 3379802).

[cl21152-bib-0406] Coratti, N. E. (2009). *The effects of increased school time on the literacy achievement of at‐risk kindergarten students* (Doctoral dissertation). Retrieved from ERIC database (ED535379).

[cl21152-bib-0407] Daunic, A. , Corbett, N. , Smith, S. , Barnes, T. , Santiago‐Poventud, L. , Chalfant, P. , Pitts, D. , & Gleaton, J. (2013). Brief report: Integrating social‐emotional learning with literacy instruction: An intervention for children at risk for emotional and behavioral disorders. Behavioral Disorders, 39(1), 43–51.

[cl21152-bib-0408] Davis, C. E. (1995). *Comparing Direct Instruction reading with a basal reading program in relation to achievement, attitude toward school, and self‐concept* (Doctoral dissertation). Available from ProQuest Information and Learning (UMI No. 9615257).

[cl21152-bib-0409] Davis, D. A. (2012). *Examining the effects of READ 180 with sixth grade students in a Southwest United States school district based on a formative assessment—Measures of Academic Progress—and its impact on leadership decisions* (Doctoral dissertation). New Mexico State University.

[cl21152-bib-0410] Davis, J. M. (2008). *The effectiveness of a late‐exit/transitional bilingual program related to the reading achievement of Hispanic limited English proficient elementary school students*. (Doctoral dissertation). Available from ProQuest Dissertations and Theses (UMI No. 3363981).

[cl21152-bib-0411] Dawes, S. (2011). *The effects of school‐wide behavior support on special education students’ achievement and office discipline referrals* (Doctoral dissertation). Tennessee State University.

[cl21152-bib-0412] De La Garza, J. V. , & Medina, M., Jr. (1985). Academic achievement as influenced by bilingual instruction for Spanish‐dominant Mexican American children. Hispanic Journal of Behavioral Sciences, 7(3), 247–259.

[cl21152-bib-0413] Denton, C. A. , Fletcher, J. M. , Anthony, J. L. , & Francis, D. J. (2006). An evaluation of intensive intervention for students with persistent reading difficulties. Journal of Learning Disabilities, 39(5), 447–466. 17004676 10.1177/00222194060390050601

[cl21152-bib-0414] Donawerth, A. S. (2013). *Bridging the gap: Fourth grade before‐school computer math lab and its impact on California Standardized Test Scores* (Doctoral dissertation). Available from ProQuest Dissertation and Theses (UMI No. 1586255).

[cl21152-bib-0415] Dougherty Stahl, K. A. , Keane, A. E. , & Simic, O. (2013). Translating policy to practice: Initiating RTI in urban schools. Urban Education, 48(3), 350–379.

[cl21152-bib-0416] Ehri, L. C. , Dreyer, L. G. , Flugman, B. , & Gross, A. (2007). Reading Rescue: An effective tutoring intervention model for language‐minority students who are struggling readers in first grade. American Educational Research Journal, 44(2), 414–448.

[cl21152-bib-0417] Englert, C. S. , Garmon, A. , Mariage, T. , Rozendal, M. , Tarrant, K. , & Urba, J. (1995). The early literacy project: Connecting across the literacy curriculum. Learning Disability Quarterly, 18(4), 253–275.

[cl21152-bib-0418] Ennemoser, M. , & Krajewski, K. (2007). Effekte der Förderung des Teil‐Ganzes‐Verständnisses bei rechenschwachen Erstklässlern. Vierteljahreszeitschrift für Heilpädagogik und ihre Nachbargebiete, 76(3), 228–240.

[cl21152-bib-0419] Esteves, K. J. (2007). Audio‐assisted reading with digital audiobooks for upper elementary students with reading disabilities. Scholarship and Professional Work—Education. Paper 78.

[cl21152-bib-0420] Eversole, V. L. (2010). *Response to intervention (RTI) model and reading achievement of elementary Latino students* (Doctoral dissertation). Available from ProQuest LLC. (UMI No. 3411934).

[cl21152-bib-0421] Falke, T. R. (2012). *The effects of implementing a computer‐based reading support program on the reading achievement of sixth graders* (Doctoral dissertation). Retrieved from ERIC database (ED549189).

[cl21152-bib-0422] Felton, R. H. (1993). Effects of instruction on the decoding skills of children with phonological‐processing problems. Journal of Learning Disabilities, 26(9), 583–589. 8283124 10.1177/002221949302600904

[cl21152-bib-0423] Fielding‐Barnsley, R. , & Hay, I. (2012). Comparative effectiveness of phonological awareness and oral language intervention for children with low emergent literacy skills. Australian Journal of Language and Literacy, 35(3), 271–286.

[cl21152-bib-0424] Flores, B. (2014). *A comparison of reading comprehension performance and student success of special education fourth grade students with moderate disabilities who received direct instruction and those who did not receive direct instruction* (Doctoral dissertation). Available from ProQuest LLC. (UMI No. 3616803).

[cl21152-bib-0425] Foorman, B. R. , Francis, D. J. , Fletcher, J. M. , Schatschneider, C. , & Mehta, P. (1998). The role of instruction in learning to read: Preventing reading failure in at‐risk children. Journal of Educational Psychology, 90(1), 37–55.

[cl21152-bib-0426] Frantz, S. O. S. (2000). *Effectiveness of the infusion of Reading Component Model based remedial reading instruction on the reading achievement of students in learning disabilities and Title I remedial reading programs* (Doctoral dissertation). Available from ProQuest Information & Learning (UMI No. 9965708).

[cl21152-bib-0427] Friesen, J. D. , & Der, D. F. (1984). The outcomes of three models of counselling and consulting. International Journal for the Advancement of Counselling, 7(1), 67–75.

[cl21152-bib-0428] Galluzzo, C. A. (2010). *The long‐term effectiveness of Reading Recovery and the cost‐efficiency of Reading Recovery relative to the Learning Disabled classification rate* (Doctoral dissertation). Available from ERIC database (ED521799).

[cl21152-bib-0429] Garcia, D. A. N. (2012). *The impact of the Brazosport Model on English Language Learners' reading achievement in grades 3‐5* (Doctoral dissertation). Available from ERIC database (ED549223).

[cl21152-bib-0430] Gerber, M. , Jimenez, T. , Leafstedt, J. , Villaruz, J. , Richards, C. , & English, J. (2004). English reading effects of small‐group intensive intervention in Spanish for K–1 English learners. Learning Disabilities Research & Practice, 19(4), 239–251.

[cl21152-bib-0431] Gifford, L. J. (2004). *Effects of phonemic awareness training on first‐grade students with learning disabilities* (Doctoral dissertation). Available from ProQuest Dissertations and Theses (UMI No. 1420993).

[cl21152-bib-0432] Glaeser, B. J. C. (1998). *The effects of an instructional model for improving reading comprehension achievement of students with learning disabilities, normally‐achieving, at‐risk, and gifted students in a multi‐age, inclusive general education classroom* (Doctoral dissertation). Available from ProQuest Dissertations and Theses (UMI No. 9905450).

[cl21152-bib-0433] Gonzalez, R. M. (1996). *Bilingual/ESL Programs Evaluation, 1995‐96. Publication Number 95‐01* (Evaluative Report). Austin Independent School District, Tex. Retrieved from ERIC database (ED404877).

[cl21152-bib-0434] Gordon, E. W. , & Armour‐Thomas, E. (2006). *The effects of dynamic pedagogy on the mathematics achievement of ethnic minority students* (Final Report). National Research Center on the Gifted and Talented, University of Connecticut.

[cl21152-bib-0435] Gottesman, R. L. , Croen, L. G. , Cerullo, F. M. , & Nathan, R. G. (1983). Diagnostic intervention for inner‐city primary graders with learning difficulties. Elementary School Journal, 83(3), 239–249.

[cl21152-bib-0436] Graham, L. , & Pegg, J. (2010). *A longitudinal evaluation of “QuickSmart”: An effective Australian intervention to improve numeracy*. Paper presented at the May 2010 American Educational Research Association Conference, Denver, CO. Retrieved from ERIC database (ED520252).

[cl21152-bib-0437] Graham, L. , & Pegg, J. (2013). *Enhancing the academic achievement of indigenous students in rural Australia*. Paper presented at the 2013 Annual Meeting of the American Educational Research Association, San Francisco, CA. Retrieved from ERIC database (ED543243).

[cl21152-bib-0438] Graham, L. , Bellert, A. , Thomas, J. , & Pegg, J. (2007). QuickSmart: A basic academic skills intervention for middle school students with learning difficulties. Journal of Learning Disabilities, 40(5), 410–419. 17915495 10.1177/00222194070400050401

[cl21152-bib-0439] Grant, S. M. (1985). The kinesthetic approach to teaching: Building a foundation for learning. Journal of Learning Disabilities, 18(8), 455–462. 4056610 10.1177/002221948501800803

[cl21152-bib-0440] Graves, A. W. , Duesbery, L. , Pyle, N. B. , Brandon, R. R. , & McIntosh, A. S. (2011). Two studies of tier II literacy development: Throwing sixth graders a lifeline. Elementary School Journal, 111(4), 641–661.

[cl21152-bib-0441] Greenwood, C. R. , Delquadri, J. C. , & Hall, R. V. (1989). Longitudinal effects of classwide peer tutoring. Journal of Educational Psychology, 81(3), 371–383.

[cl21152-bib-0442] Greenwood, C. R. , Dinwiddie, G. , Terry, B. , Wade, L. , Stanley, S. O. , Thibadeau, S. , & Delquadri, J. C. (1984). Teacher‐versus peer‐mediated instruction: An ecobehavioral analysis of achievement outcomes. Journal of Applied Behavior Analysis, 17(4), 521–538. 6526770 10.1901/jaba.1984.17-521 PMC1307973

[cl21152-bib-0443] Gretzula, W. J. (2007). *An analysis of a pre‐first grade program on academic, social and emotional achievement* (Doctoral dissertation). Available from ProQuest Information & Learning (UMI No. 3255873).

[cl21152-bib-0444] Guinn, T. R. (2009). *The effectiveness of SRA Corrective Reading and SRA Connecting Math programs as reading and math intervention programs for students with disabilities* (Doctoral dissertation). Available from ProQuest LLC. (UMI No. 3390671).

[cl21152-bib-0445] Guthrie, J. T. , McRae, A. , Coddington, C. S. , Lutz Klauda, S. , Wigfield, A. , & Barbosa, P. (2009). Impacts of comprehensive reading instruction on diverse outcomes of low‐and high‐achieving readers. Journal of Learning Disabilities, 42(3), 195–214. 19264929 10.1177/0022219408331039

[cl21152-bib-0446] Gutierrez, S. (2012). *The impact of school‐based mentoring on student achievement and school engagement in elementary aged at‐risk students: Implications for leadership* (Doctoral dissertation). Available from ProQuest Information & Learning (UMI No. 3515596).

[cl21152-bib-0447] Gutman, T. E. (2011). *The effects of Read Naturally on reading fluency and comprehension for students of low socioeconomic status* (Doctoral dissertation). Available from ProQuest LLC. (UMI No. 3479155).

[cl21152-bib-0448] Halvorsen, A. L. , Duke, N. K. , Brugar, K. A. , Block, M. K. , Strachan, S. L. , Berka, M. B. , & Brown, J. M. (2012). *Narrowing the achievement gap in second‐grade social studies and content area literacy: The promise of a project‐based approach* (Working Paper: 26). The Education Policy Center, Michigan State University.

[cl21152-bib-0449] Hasselbring, T. S. , & Moore, P. R. (1996). Developing mathematical literacy through the use of contextualized learning environments. Journal of Computing in Childhood Education, 7(3‐4), 199–222.

[cl21152-bib-0450] Hayward, D. , Das, J. P. , & Janzen, T. (2007). Innovative programs for improvement in reading through cognitive enhancement: A remediation study of Canadian First Nations children. Journal of Learning Disabilities, 40(5), 443–457. 17915499 10.1177/00222194070400050801

[cl21152-bib-0451] Hernandez‐Gutierrez, J. (2008). *The effects of academic interventions on the development of reading academic competence in fourth grade students* (Doctoral dissertation). University of North Texas.

[cl21152-bib-0452] Hock, M. F. , Brasseur‐Hock, I. F. , Hock, A. J. , & Duvel, B. (2017). The effects of a comprehensive reading program on reading outcomes for middle school students with disabilities. Journal of Learning Disabilities, 50(2), 195–212. 26721889 10.1177/0022219415618495

[cl21152-bib-0453] Holmes, J. , & Gathercole, S. E. (2014). Taking working memory training from the laboratory into schools. Educational Psychology, 34(4), 440–450. 26494933 10.1080/01443410.2013.797338 PMC4579053

[cl21152-bib-0454] Hopkins, K. R. (1996). *A study of the effect of interactive language in the stimulation of cognitive functioning for students with learning disabilities* (Doctoral dissertation). Available from ProQuest Information & Learning (UMI No. 9622283).

[cl21152-bib-0455] Hotulainen, R. , Mononen, R. , & Aunio, P. (2016). Thinking skills intervention for low‐achieving first graders. European Journal of Special Needs Education, 31(3), 360–375.

[cl21152-bib-0456] Hunt, G. (1994). *The improvement of reading comprehension of the second grade at‐risk students using multisensory methods of instruction* (Master's Thesis). Retrieved from ERIC database (ED371293).

[cl21152-bib-0457] Hurford, D. P. , Johnston, M. , Nepote, P. , Hampton, S. , Moore, S. , Neal, J. , Mueller, A. , McGeorge, K. , Huff, L. , Awad, A. , Tatro, C. , Juliano, C. , & Huffman, D. (1994). Early identification and remediation of phonological‐processing deficits in first‐grade children at risk for reading disabilities. Journal of Learning Disabilities, 27(10), 647–659. 7844481 10.1177/002221949402701005

[cl21152-bib-0458] Interactive, Inc . (2002). *An efficacy study of READ 180: A print and electronic adaptive intervention program grades 4 and above* (Final Report Study of READ 180 in the Council of Great City Schools). Scholastic Inc.

[cl21152-bib-0459] Irby, B. J. , Tong, F. , Lara‐Alecio, R. , Guerero, C. L. , Lopez, T. , Cocoran, R. , & Slavin, R. (2016). *Evaluating instructional intervention in promoting students’ English development and science learning in an urban district*. Paper presented at the 2016 Annual Meeting of the American Educational Research Association. Retrieved from AERA Online Paper Repository.

[cl21152-bib-0460] Ito, H. R. (1980). Long‐term effects of resource room programs on learning disabled children's reading. Journal of Learning Disabilities, 13(6), 36–40. 7410955

[cl21152-bib-0461] Iversen, S. , & Tunmer, W. E. (1993). Phonological processing skills and the Reading Recovery Program. Journal of Educational Psychology, 85(1), 112–126.

[cl21152-bib-0462] Jack, D. L. (2011). *An examination of the achievement and disciplinary incidents of grade 4 students participating in a web‐based language arts and writing intervention program* (Doctoral dissertation). Available from ProQuest Dissertations and Theses (UMI No. 3476351).

[cl21152-bib-0463] Jesson, R. , & Limbrick, L. (2014). Can gains from early literacy interventions be sustained? The case of Reading Recovery. Journal of research in reading, 37(1), 102–117.

[cl21152-bib-0464] Jimerson, S. , Carlson, E. , Rotert, M. , Egeland, B. , & Sroufe, L. A. (1997). A prospective, longitudinal study of the correlates and consequences of early grade retention. Journal of School Psychology, 35(1), 3–25.

[cl21152-bib-0465] Jones, E. A. , Sudweeks, R. R. , Young, R. , & Larsen, R. A. (2016). *Using regression discontinuity with multiple cutoffs to estimate treatment effects in elementary school reading interventions*. Paper presented at the 2016 Annual meeting of the American Educational Research Association. Retrieved from the AERA Online Paper Repository.

[cl21152-bib-0466] Jones‐Mason, K. S. (2012). *A comparison of service delivery models for special education middle school students receiving moderate intervention services* (Doctoral dissertation). Retrieved from ERIC database (ED549027).

[cl21152-bib-0467] Jordan, N. C. , Dyson, N. , & Glutting, J. (2011 *). Developing Number Sense in Kindergartners at Risk for Learning Difficulties in Mathematics* (Conference Paper). Society for Research on Educational Effectiveness. Retrieved from ERIC database (ED528836).

[cl21152-bib-0468] Juel, C. (1996). What makes literacy tutoring effective? Reading Research Quarterly, 31(3), 268–289.

[cl21152-bib-0469] Kajamies, A. , Vauras, M. , & Kinnunen, R. (2010). Instructing low‐achievers in mathematical word problem solving. Scandinavian Journal of Educational Research, 54(4), 335–355.

[cl21152-bib-0470] Kamberg, S. (2009). *Sustained impact of the Reading Recovery early intervention as students move into more content‐laden grades*. (Doctoral dissertation). Available from ProQuest LLC. (UMI No. 3370193).

[cl21152-bib-0471] Keita, M. (2011). *Examination of the effects of the response to intervention program on the reading achievement test scores of third grade English as second language students* (Doctoral dissertation). Available from ProQuest Dissertations and Theses (UMI No. 3463488).

[cl21152-bib-0472] Kerchner, L. B. , & Kistinger, B. J. (1984). Language processing/word processing: Written expression, computers and learning disabled students. Learning Disability Quarterly, 7(4), 329–335.

[cl21152-bib-0473] Klijian, G. M. (2009). *What about the children left‐behind? An evaluation of a reading intervention program* (Doctoral dissertation). Available from ProQuest LLC. (UMI No. 3368578).

[cl21152-bib-0474] Klingner, J. K. , Vaughn, S. , Arguelles, M. E. , Tejero Hughes, M. , & Ahwee Leftwich, S. (2004). Collaborative Strategic Reading: “Real‐World” lessons from classroom teachers. Remedial and Special Education, 25(5), 291–302.

[cl21152-bib-0475] Knapp, N. F. , & Winsor, A. P. (1998). A reading apprenticeship for delayed primary readers. Literacy Research and Instruction, 38(1), 13–29.

[cl21152-bib-0476] Kong, J. E. (2009). *Effects of comprehension strategies on test scores of third grade special education students* (Doctoral dissertation). Available from ProQuest LLC. (UMI No. 3376761).

[cl21152-bib-0477] Lara‐Alecio, R. , Tong, F. , Irby, B. J. , Guerrero, C. , Huerta, M. , & Fan, Y. (2012). The effect of an instructional intervention on middle school english learners' science and English reading achievement. Journal of Research in Science Teaching, 49(8), 987–1011.

[cl21152-bib-0478] Laub, C. M. (1997). *Effectiveness of Project Read on word attack skills and comprehension for third and fourth grade students with learning disabilities* (Doctoral dissertation). Available from ProQuest Dissertations and Theses (UMI No. 1386289).

[cl21152-bib-0479] Lawson, S. (2011). *The Read 180 program: Analysis of program effect on the reading achievement, motivation, engagement, and self‐efficacy of sixth grade middle school students* (Doctoral dissertation). Retrieved from ERIC database (ED534548).

[cl21152-bib-0480] Leafstedt, J. M. , Richards, C. R. , & Gerber, M. M. (2004). Effectiveness of explicit phonological‐awareness instruction for at‐risk English learners. Learning Disabilities Research & Practice, 19(4), 252–261.

[cl21152-bib-0481] Leong, C. K. (1995). Effects of on‐line reading and simultaneous DECtalk auding in helping below‐average and poor readers comprehend and summarize text. Learning Disability Quarterly, 18(2), 101–116.

[cl21152-bib-0482] Linan‐Thompson, S. , Pedrotty Bryant, D. , Dickson, S. V. , & Kouzekanani, K. (2005). Spanish literacy instruction for at‐risk kindergarten students. Remedial and Special Education, 26(4), 236–244.

[cl21152-bib-0483] Lleras, C. , & Rangel, C. (2009). Ability grouping practices in elementary school and African American/Hispanic achievement. American Journal of Education, 115(2), 279–304.

[cl21152-bib-0484] Lloyd, J. , Cullinan, D. , Heins, E. D. , & Epstein, M. H. (1980). Direct instruction: Effects on oral and written language comprehension. Learning Disability Quarterly, 3(4), 70–76.

[cl21152-bib-0485] Lo, Y. Y. , Wang, C. , & Haskell, S. (2009). Examining the impacts of early reading intervention on the growth rates in basic literacy skills of at‐risk urban kindergarteners. Journal of Special Education, 43(1), 12–28.

[cl21152-bib-0486] López, M. G. , & Tashakkori, A. (2003). *Utilizing two‐way bilingual education for reducing the achievement lag of LEP students in primary grades: A longitudinal study*. Paper presented at the Annual Meeting of the American Educational Research Association, Chicago, IL. Retrieved from ERIC database (ED478290).

[cl21152-bib-0487] López, M. G. , & Tashakkori, A. (2004a). Effects of a two‐way bilingual program on the literacy development of students in kindergarten and first grade. Bilingual Research Journal, 28(1), 19–34.

[cl21152-bib-0488] López, M. G. , & Tashakkori, A. (2004b). Narrowing the gap: Effects of a two‐way bilingual education program on the literacy development of at‐risk primary students. Journal of Education for Students Placed at Risk, 9(4), 325–336.

[cl21152-bib-0489] Lorence, J. , Dworkin, A. G. , Toenjes, L. A. , Hill, A. N. , Rotherham, A. , & Shepard, L. A. (2002). Grade retention and social promotion in Texas, 1994‐99: Academic achievement among elementary school students. Brookings Papers on Education Policy, 5, 13–67.

[cl21152-bib-0490] Lovett, M. W. , Frijters, J. C. , Wolf, M. , Steinbach, K. A. , Sevcik, R. A. , & Morris, R. D. (2017). Early intervention for children at risk for reading disabilities: The impact of grade at intervention and individual differences on intervention outcomes. Journal of Educational Psychology, 109(7), 889–914. 35664550 10.1037/edu0000181 PMC9164258

[cl21152-bib-0491] Mac Iver, M. A. , & Kemper, E. (2002). The impact of Direct Instruction on elementary students' reading achievement in an urban school district. Journal of Education for Students Placed at Risk, 7(2), 197–220.

[cl21152-bib-0492] Macaruso, P. , & Walker, A. (2008). The efficacy of computer‐assisted instruction for advancing literacy skills in kindergarten children. Reading Psychology, 29(3), 266–287.

[cl21152-bib-0493] MacDonald, C. , & Figueredo, L. (2010). Closing the gap early: Implementing a literacy intervention for at‐risk kindergartners in urban schools. The Reading Teacher, 63(5), 404–419.

[cl21152-bib-0494] Maldonado, J. A. (1994). Bilingual special education: Specific learning disabilities in language and reading. Journal of Education Issues of Language Minority Students, 14(2), 127–147.

[cl21152-bib-0495] Mantzicopoulos, P. , Morrison, D. , Stone, E. , & Setrakian, W. (1992). Use of the SEARCH/TEACH tutoring approach with middle‐class students at risk for reading failure. The Elementary School Journal, 92(5), 573–586.

[cl21152-bib-0496] Marian, V. , Shook, A. , & Schroeder, S. R. (2013). Bilingual two‐way immersion programs benefit academic achievement. Bilingual Research Journal, 36(2), 167–186. 10.1080/15235882.2013.818075 PMC3838203 24277993

[cl21152-bib-0497] Marr, M. B. , Algozzine, B. , Nicholson, K. , & Keller Dugan, K. (2011). Building oral reading fluency with peer coaching. Remedial and Special Education, 32(3), 256–264.

[cl21152-bib-0498] Mathes, P. G. , Howard, J. K. , Allen, S. H. , & Fuchs, D. (1998). Peer‐assisted learning strategies for first‐grade readers: Responding to the needs of diverse learners. Reading Research Quarterly, 33(1), 62–94.

[cl21152-bib-0499] Mathes, P. G. , Torgesen, J. K. , & Allor, J. H. (2001). The effects of peer‐assisted literacy strategies for first‐grade readers with and without additional computer‐assisted instruction in phonological awareness. American Educational Research Journal, 38(2), 371–410.

[cl21152-bib-0500] McDermott, P. A. , & Watkins, M. W. (1983). Computerized vs. conventional remedial instruction for learning‐disabled pupils. Journal of Special Education, 17(1), 81–88.

[cl21152-bib-0501] McIntyre, E. , Jones, D. , Powers, S. , Newsome, F. , Petrosko, J. , Powell, R. , & Bright, K. (2005). Supplemental instruction in early reading: Does it matter for struggling readers? Journal of Educational Research, 99(2), 99–107.

[cl21152-bib-0502] McLean, A. M. (2015). *Math fact automaticity and its effect on student math achievement in a Northern California school district* (Doctoral dissertation, California State University, Chico).

[cl21152-bib-0503] McMasters, A. B. (2011). *Use of a tier 3 evidence‐based intervention with progress monitoring, formative assessment, and student goal‐setting: An evaluation of the immediate and long‐term effects on student reading achievement* (Doctoral dissertation, Indiana University).

[cl21152-bib-0504] Medina, M. , Saldate, M. , & Mishra, S. P. (1985). The sustaining effects of bilingual instruction: A follow‐up study. Journal of Instructional Psychology, 12(3), 132.

[cl21152-bib-0505] Menar, E. A. (2002). *Student Support Program: Analyzing the effectiveness of an academic intervention program* (Doctoral dissertation). Available from ProQuest Information and Learning (UMI No. 3044034).

[cl21152-bib-0506] Mevarech, Z. R. , & Rich, Y. (1985). Effects of computer‐assisted mathematics instruction on disadvantaged pupils' cognitive and affective development. Journal of Educational Research, 79(1), 5–11.

[cl21152-bib-0507] Meyer, P. A. F. (1986). *A comparative analysis of the value of intrinsic motivation in computer software on the math achievement, attitudes, attendance, and depth‐of‐involvement of underachieving students (CAI)* (Doctoral dissertation, The College of William and Mary).

[cl21152-bib-0508] Mitchell, V. (2010). *A quasi‐experimental study of the use of “Dr. Cupp's Readers” in comparison to traditional instruction of at‐risk second grade students' test scores* (Doctoral dissertation). Available from ProQuest LLC. (UMI No. 3397991).

[cl21152-bib-0509] Molina, S. , Garrido, M. A. , & Das, J. P. (1997). Process‐based enhancement of reading: An empirical study. Developmental Disabilities Bulletin, 25(1), 68–76.

[cl21152-bib-0510] Mononen, R. , & Aunio, P. (2014). A mathematics intervention for low‐performing Finnish second graders: findings from a pilot study. European Journal of Special Needs Education, 29(4), 457–473.

[cl21152-bib-0511] Moore, C. (2014). *The effects of a direct‐instruction math intervention on standardized test scores of at‐risk middle school students* (Doctoral dissertation). Available from ProQuest Information and Learning (UMI No. 3632478).

[cl21152-bib-0512] Moran, A. S. , Swanson, H. L. , Gerber, M. M. , & Fung, W. (2014). The effects of paraphrasing interventions on problem‐solving accuracy for children at risk for math disabilities. Learning Disabilities Research & Practice, 29(3), 97–105.

[cl21152-bib-0513] Morgan, P. L. , Fuchs, D. , Compton, D. L. , Cordray, D. S. , & Fuchs, L. S. (2008). Does early reading failure decrease children's reading motivation? Journal of Learning Disabilities, 41(5), 387–404. 18768772 10.1177/0022219408321112

[cl21152-bib-0514] Morocco, C. C. (1989). *The impact of computer‐supported writing instruction on the writing quality of learning‐disabled students. Final Report*. Retrieved from ERIC database (ED319181).

[cl21152-bib-0515] Morta, A. L. (2010). *A Rubric and an Individualized Educational Plan to Increase Academic Achievement in Middle School Students with Disabilities* (Doctoral dissertation). Available from ProQuest Dissertation and Theses (UMI No. 3396331).

[cl21152-bib-0516] Moser, S. E. , West, S. G. , & Hughes, J. N. (2012). Trajectories of math and reading achievement in low‐achieving children in elementary school: Effects of early and later retention in grade. Journal of Educational Psychology, 104(3), 603–621. 23335818 10.1037/a0027571 PMC3547658

[cl21152-bib-0517] Murphy, J. A. (2003). *An application of growth curve analysis: The evaluation of a reading intervention program* (Doctoral dissertation). Available from ProQuest Information and Learning (UMI No. 3114448).

[cl21152-bib-0518] Myers, B. G. (2016). *Investigating the effects of the Academy of Reading program on middle school reading achievement* (Doctoral dissertation). Available from ProQuest Information and Learning (UMI No. 10119087).

[cl21152-bib-0519] Nave, J. (2007). *An assessment of READ 180 regarding its association with the academic achievement of at‐risk students in Sevier County schools* (Doctoral dissertation). East Tennessee State University. Electronic Theses and Dissertations. Paper 2107. http://dc.etsu.edu/etd/2107

[cl21152-bib-0520] Nidich, S. , Mjasiri, S. , Nidich, R. , Rainforth, M. , Grant, J. , Valosek, L. , & Zigler, R. L. (2011). Academic achievement and transcendental meditation: A study with at‐risk urban middle school students. Education, 131(3), 556–564.

[cl21152-bib-0521] O'Connor, R. E. , Bocian, K. M. , Sanchez, V. , & Beach, K. D. (2014). Access to a responsiveness to intervention model: Does beginning intervention in kindergarten matter? Journal of Learning Disabilities, 47(4), 307–328. 23019070 10.1177/0022219412459354

[cl21152-bib-0522] O'Connor, R. E. , Harty, K. R. , & Fulmer, D. (2005). Tiers of intervention in kindergarten through third grade. Journal of Learning Disabilities, 38(6), 532–538. 16392695 10.1177/00222194050380060901

[cl21152-bib-0523] O'Connor, R. E. , Notari‐Syverson, A. , & Vadasy, P. F. (1996). Ladders to literacy: The effects of teacher‐led phonological activities for kindergarten children with and without disabilities. Exceptional Children, 63(1), 117–130.

[cl21152-bib-0524] O'Connor, R. E. , Notari‐Syverson, A. , & Vadasy, P. F. (1998). First‐grade effects of teacher‐led phonological activities in kindergarten for children with mild disabilities: A follow‐up study. Learning Disabilities Research & Practice, 13(1), 43–52.

[cl21152-bib-0525] O'Melia, M. C. , & Rosenberg, M. S. (1994). Effects of cooperative homework teams on the acquisition of mathematics skills by secondary students with mild disabilities. Exceptional Children, 60(6), 538–548.

[cl21152-bib-0526] Osborn, J. , Freeman, A. , Burley, M. , Wilson, R. , Jones, E. , & Rychener, S. (2007). Effect of tutoring on reading achievement for students with cognitive disabilities, specific learning disabilities, and students receiving Title I services. Education and Training in Developmental Disabilities, 42(4), 467–474.

[cl21152-bib-0527] Phillips, N. H. (1990). *Retention, promotion, or two‐tier kindergarten: Which reaches the at‐risk reader more effectively?* Paper presented at the Annual Meeting of the International Reading Association, Atlanta, GA. Retrieved from ERIC database (ED321230).

[cl21152-bib-0528] Pinnell, G. S. , Lyons, C. A. , Deford, D. E. , Bryk, A. S. , & Seltzer, M. (1994). Comparing instructional models for the literacy education of high‐risk first graders. Reading Research Quarterly, 29(1), 9–39.

[cl21152-bib-0529] Piro, J. M. , & Ortiz, C. (2009). The effect of piano lessons on the vocabulary and verbal sequencing skills of primary grade students. Psychology of Music, 37(3), 325–347.

[cl21152-bib-0530] Plony, D. A. (2013). *The effects of Read 180 on student achievement* (Doctoral dissertation). Available from ProQuest LLC. (UMI No. 3563453).

[cl21152-bib-0531] Porter, D. G. (2010). *The relationship of supplemental reading services and the reading achievement scores of first and second grade students* (Doctoral dissertation). Available from ProQuest Dissertations and Theses (UMI No. 3427029).

[cl21152-bib-0532] Rabiner, D. L. , Murray, D. W. , Skinner, A. T. , & Malone, P. S. (2010). A randomized trial of two promising computer‐based interventions for students with attention difficulties. Journal of Abnormal Child Psychology, 38(1), 131–142. 19697119 10.1007/s10802-009-9353-x

[cl21152-bib-0533] Rafdal, B. H. , McMaster, K. L. , McConnell, S. R. , Fuchs, D. , & Fuchs, L. S. (2011). The effectiveness of kindergarten peer‐assisted learning strategies for students with disabilities. Exceptional Children, 77(3), 299–316.

[cl21152-bib-0534] Rapp, J. C. (1991). The effect of cooperative learning on selected student variables (Cooperative Integrated Reading and Composition on academic achievement in reading comprehension, vocabulary and spelling and on student self‐esteem) (Doctoral dissertation). Available from ProQuest Dissertation and Theses (UMI. No. 9207225).

[cl21152-bib-0535] Rasinski, T. , & Oswald, R. (2005). Making and writing words: Constructivist word learning in a second‐grade classroom. Reading & Writing Quarterly, 21(2), 151–163.

[cl21152-bib-0536] Redmon, B. G. (2007). The impact of full inclusion on the academic achievement of students with disabilities in grades 3 to 6 (Doctoral dissertation). Available from ProQuest Dissertation and Theses (UMI. No. 3267063).

[cl21152-bib-0537] Reyes‐Bonilla, M. A. , & Carrasquillo, A. L. (1993). *The development of English oral communication in learning disabled Spanish speaking students in Puerto Rico* (Technical Report: 143). Retrieved from ERIC database (ED357637).

[cl21152-bib-0538] Rhett, T. Y. (2011). *The effectiveness of a reading intervention pull‐out program* (Doctoral dissertation). Available from ProQuest Dissertations and Theses (UMI No. 3487011).

[cl21152-bib-0539] Ross, P. A. , & Braden, J. P. (1991). The effects of token reinforcement versus cognitive behavior modification on learning‐disabled students' math skills. Psychology in the Schools, 28(3), 247–256.

[cl21152-bib-0540] Ross, S. M. , & Smith, L. J. (1994). Effects of the Success for All model on kindergarten through second‐grade reading achievement, teachers' adjustment, and classroom‐school climate at an inner‐city school. Elementary School Journal, 95(2), 121–138.

[cl21152-bib-0541] Ross, S. M. , Smith, L. J. , & Casey, J. P. (1997). Preventing early school failure: Impacts of Success for All on standardized test outcomes, minority group performance, and school effectiveness. Journal of Education for Students Placed at Risk, 2(1), 29–53.

[cl21152-bib-0542] Ross, S. M. , Smith, L. J. , & Casey, J. P. (1999). “Bridging the Gap”: The effects of the Success for All program on elementary school reading achievement as a function of student ethnicity and ability Level. School Effectiveness and School Improvement, 10(2), 129–150.

[cl21152-bib-0543] Rothenberg, J. J. (1990). *Long term outcomes of an early intervention: Performance on three problem‐solving tasks*. Paper presented at the Annual Meeting of the American Educational Research Association, Boston. Retrieved from ERIC database (ED320917).

[cl21152-bib-0544] Saint‐Laurent, L. (1996). *PIER: An inclusive model for at‐risk Students*. Paper presented at the Annual International Convention of the Council for Exceptional Children, Orlando. Retrieved from ERIC database (ED400666).

[cl21152-bib-0545] Saracho, O. N. (1982). The effects of a computer‐assisted instruction program on basic skills achievement and attitudes toward instruction of Spanish‐speaking migrant children. American Educational Research Journal, 19(2), 201–219.

[cl21152-bib-0546] Sauvé, D. (2009). *The suitability of French immersion education for students with reading disabilities* (Doctoral Dissertation). ProQuest Information and Learning. Retrieved from http://search.ebscohost.com/login.aspx?direct=true%26db=psyh%26AN=2009-99190-251%26site=ehost-live

[cl21152-bib-0547] Scalf, G. T. (2014). *Learning‐disabled students: A comparison of achievement scores of students receiving services in pull‐out classrooms and inclusion classrooms* (Doctoral Dissertation). Electronic Theses and Dissertations. Paper 2434. Retrieved from http://dc.etsu.edu/etd/2434

[cl21152-bib-0548] Scientific Learning Corporation . (2004a). Improved language skills by children with low reading performance who used Fast ForWord Language. MAPS for Learning: Product Report, 3(1), 1–13.

[cl21152-bib-0549] Scientific Learning Corporation . (2004b). Improved Ohio Reading Proficiency Test scores by students in the Springfield City School District who used Fast ForWord® products. MAPS for Learning: Educator Report, 8(8), 1–6.

[cl21152-bib-0550] Scientific Learning Corporation . (2004c). Improved reading achievement by students in the school district of Philadelphia who used Fast ForWord® products. MAPS for Learning: Educator Report, 8(21), 1–6.

[cl21152-bib-0551] Scientific Learning Corporation . (2007). Improved reading skills by students in the South Madison Community School Corporation who used Fast ForWord® products. MAPS for Learning: Educator Report, 11(34), 1–7.

[cl21152-bib-0552] Scott, L. S. (1999). *The Accelerated Reader program, reading achievement, and attitudes of students with learning disabilities* (Doctoral dissertation). Retrieved from ERIC database (ED434431).

[cl21152-bib-0553] Scruggs, T. E. , & Osguthorpe, R. T. (1986). Tutoring interventions within special education settings: A comparison of cross‐age and peer tutoring. Psychology in the Schools, 23(2), 187–193.

[cl21152-bib-0554] Shamey, T. (2008). *Effects of early elementary Reading Recovery programs on middle‐school students: A longitudinal evaluation* (Doctoral dissertation). Available from ProQuest LLC. (UMI No. 3337490).

[cl21152-bib-0555] Shepard, L. A. , & Smith, M. L. (1987). Effects of kindergarten retention at the end of first grade. Psychology in the Schools, 24(4), 346–357.

[cl21152-bib-0556] Shields, K. A. , Walsh, M. E. , & Lee‐St John, T. J. (2016). The relationship of a systemic student support intervention to academic achievement in urban catholic schools. Journal of Catholic Education, 19(3), 116–141.

[cl21152-bib-0557] Shields, V. E. (1995). *The impact of selected intervention practices on the academic performance, behavior, and attendance of identified at‐risk elementary school students* (Doctoral dissertation). Available from ProQuest Information & Learning (UMI No. 9502487).

[cl21152-bib-0558] Sigears, K. A. (2008). *The impact of the implementation of the Scholastic READ 180 model on reading skills development of middle school students with learning disabilities as compared to those using the traditional resource reading model* (Doctoral dissertation). Available from ProQuest Information & Learning (UMI No. 3320196).

[cl21152-bib-0559] Silvious, N. B. (2008). *Effects of Saxon Math program of instruction on the mathematics achievement of students with learning disabilities in grades 2 through 8* (Doctoral dissertation). Available from ProQuest Information & Learning (UMI No.3363773).

[cl21152-bib-0560] Simmons, D. C. , Fuchs, L. S. , Fuchs, D. , Mathes, P. , & Hodge, J. P. (1995). Effects of explicit teaching and peer tutoring on the reading achievement of learning‐disabled and low‐performing students in regular classrooms. Elementary School Journal, 95(5), 387–408.

[cl21152-bib-0561] Soriano, M. , Miranda, A. , Soriano, E. , Nievas, F. , & Félix, V. (2011). Examining the efficacy of an intervention to improve fluency and reading comprehension in Spanish children with reading disabilities. International Journal of Disability, Development and Education, 58(1), 47–59.

[cl21152-bib-0562] Southard, N. A. , & May, D. C. (1996). The effects of pre‐first‐grade programs on student reading and mathematics achievement. Psychology in the Schools, 33(2), 132–142.

[cl21152-bib-0563] Spaulding, C. L. (2007). *Early Literacy Intervention as an Alternative Approach to Instruction: A Longitudinal Investigation* (Doctoral dissertation). Available from ProQuest Information & Learning (UMI No. 3260122).

[cl21152-bib-0564] Spencer, F. , Snart, F. , & Das, J. P. (1989). A process‐based approach to the remediation of spelling in students with reading disabilities. Alberta Journal of Educational Research, 35(4), 269–282.

[cl21152-bib-0565] Spillios, J. C. , & Janzen, H. L. (1983). The effect of relaxation therapy on achievement for anxious learning disabled students. School Psychology International, 4(2), 101–107.

[cl21152-bib-0566] Steinberg, D. I. (1991). *The effects of the participation of Chapter One elementary school students, as tutors and tutees, in a cross‐age tutoring program, on their academic achievement* (Doctoral dissertation). Available from ProQuest Dissertations and Theses (UMI No. 9133171).

[cl21152-bib-0567] Stephens, K. E. (2007). *The impact of a science‐based integrated instructional protocol on the motivation, reading comprehension, and science achievement of fourth and fifth graders* (Doctoral dissertation). Available from ProQuest Information & Learning (UMI No. 3295379).

[cl21152-bib-0568] Stevens, R. J. , Van Meter, P. N. , Garner, J. , Warcholak, N. , Bochna, C. , & Hall, T. (2008). Reading and integrated literacy strategies (RAILS): An integrated approach to early reading. Journal of Education for Students Placed at Risk, 13(4), 357–380.

[cl21152-bib-0569] Swanson, H. L. (2015). Cognitive strategy interventions improve word problem solving and working memory in children with math disabilities. Frontiers in Psychology, 6(1099), 1–13. 26300803 10.3389/fpsyg.2015.01099 PMC4523823

[cl21152-bib-0570] Swanson, H. L. , Lussier, C. M. , & Orosco, M. J. (2015). Cognitive strategies, working memory, and growth in word problem solving in children with math difficulties. Journal of Learning Disabilities, 48(4), 339–358. 23963049 10.1177/0022219413498771

[cl21152-bib-0571] Swanson, H. L. , Lussier, C. , & Orosco, M. (2013). Effects of cognitive strategy interventions and cognitive moderators on word problem solving in children at risk for problem solving difficulties. Learning Disabilities Research & Practice, 28(4), 170–183.

[cl21152-bib-0572] Swanson, H. L. , Orosco, M. J. , & Lussier, C. (2013). *Does cognitive strategy training on word problems compensate for working memory capacity in children with math difficulties?* (Conference Abstract Template). Retrieved from ERIC database (ED563039).

[cl21152-bib-0573] Tong, F. , Irby, B. J. , Lara‐Alecio, R. , Guerrero, C. , Fan, Y. , & Huerta, M. (2014). A randomized study of a literacy‐integrated science intervention for low‐socio‐economic status middle school students: Findings from first‐year implementation. International Journal of Science Education, 36(12), 2083–2109.

[cl21152-bib-0574] Tracey, D. H. , & Young, J. W. (2007). Technology and early literacy: The impact of an integrated learning system on high‐risk kindergartners’ achievement. Reading Psychology, 28(5), 443–467.

[cl21152-bib-0575] Trautman, T. , & Howe, Q. (2004). Computer‐aided instruction in mathematics: Improving performance in an inner city elementary school serving mainly English language learners. Oklahoma City, OK: American Education Corporation.

[cl21152-bib-0576] Treat, W. A. (2013). *Animal‐assisted literacy instruction for students with identified learning disabilities: Examining the effects of incorporating a therapy dog into guided oral reading sessions* (Doctoral dissertation). Santa Cruz, CA: University of California.

[cl21152-bib-0577] Trexler, A. W. (2007). *A study to determine the effectiveness of computer‐assisted instruction on the mathematics achievement levels of learning disabled elementary students* (Doctoral dissertation). Available from ProQuest Information & Learning (UMI No. 3361419).

[cl21152-bib-0578] Trifiletti, J. J. , Frith, G. H. , & Armstrong, S. (1984). Microcomputers versus resource rooms for LD students: A preliminary investigation of the effects on math skills. Learning Disability Quarterly, 7(1), 69–76.

[cl21152-bib-0579] Troia, G. A. (2004). Migrant students with limited English proficiency: Can Fast ForWord Language™ make a difference in their language skills and academic achievement? Remedial and Special Education, 25(6), 353–366.

[cl21152-bib-0580] Tucker, C. , & Jones, D. (2010). Response to Intervention: Increasing Fluency, Rate, and Accuracy for Students at Risk for Reading Failure. National Forum of Educational Administration & Supervision Journal, 28(1), 28–47.

[cl21152-bib-0581] Turlo, K. E. (1990). *Expository text comprehension of reading/learning‐disabled and regular fourth‐grade students taught with a hypothesis testing strategy* (Doctoral dissertation). Available from ProQuest Dissertations and Theses (UMI No. 9025202).

[cl21152-bib-0582] Uzomah, S. L. (2012). *Teaching mathematics to kindergarten students through a multisensory approach* (Doctoral dissertation). Walden University.

[cl21152-bib-0583] Vadasy, P. F. , Sanders, E. A. , & Peyton, J. A. (2005). Relative effectiveness of reading practice or word‐level instruction in supplemental tutoring: How text matters. Journal of Learning Disabilities, 38(4), 364–380. 16122070 10.1177/00222194050380041401

[cl21152-bib-0584] Vadasy, P. F. , Sanders, E. A. , Peyton, J. A. , & Jenkins, J. R. (2002). Timing and intensity of tutoring: A closer look at the conditions for effective early literacy tutoring. Learning Disabilities Research & Practice, 17(4), 227–241.

[cl21152-bib-0585] Van Der Jagt, J. W. (1999). *Quantitative analyses of a study investigating three spelling interventions and students with learning disabilities recall*. Paper presented at the Annual Meeting of the Mid‐South Educational Research Association. Retrieved from ERIC database (ED436911).

[cl21152-bib-0586] Van Voorhis, F. L. (2011). Adding families to the homework equation: A longitudinal study of mathematics achievement. Education and Urban Society, 43(3), 313–338.

[cl21152-bib-0587] Vellutino, F. R. , Scanlon, D. M. , Small, S. , & Fanuele, D. P. (2006). Response to intervention as a vehicle for distinguishing between children with and without reading disabilities: Evidence for the role of kindergarten and first‐grade interventions. Journal of Learning Disabilities, 39(2), 157–169. 16583795 10.1177/00222194060390020401

[cl21152-bib-0588] Vernon‐Feagans, L. , Gallagher, K. , Ginsberg, M. C. , Amendum, S. , Kainz, K. , Rose, J. , & Burchinal, M. (2010). A diagnostic teaching intervention for classroom teachers: Helping struggling readers in early elementary school. Learning Disabilities Research & Practice, 25(4), 183–193.

[cl21152-bib-0589] Vollands, S. R. (1996). *Experimental evaluation of computer assisted self‐assessment of reading comprehension: Effects on reading achievement and attitude* (Evaluative report: 142). Retrieved from ERIC database (ED408567).

[cl21152-bib-0590] Walker, H. M. , Seeley, J. R. , Small, J. , Severson, H. H. , Graham, B. A. , Feil, E. G. , Serna, L. , Golly, A. M. , & Forness, S. R. (2009). A randomized controlled trial of the First Step to Success early intervention: Demonstration of program efficacy outcomes in a diverse, urban school district. Journal of Emotional and Behavioral Disorders, 17(4), 197–212.

[cl21152-bib-0591] Warfel, S. J. (2000). *Academic and behavioral effects of a transitional first grade program by the end of second grade* (Doctoral dissertation). Available from ProQuest Information & Learning (UMI No. 9947090).

[cl21152-bib-0592] Wehbe, H. (2012). *The effect of full‐day kindergarten on the reading scores of limited English‐proficient students* (Doctoral dissertation). Available from ProQuest Information & Learning (UMI No. 3546013).

[cl21152-bib-0593] Weiss, D. H. (1992). *The effects of writing process instruction on the writing and reading performance of students with learning disabilities* (Doctoral dissertation). Available from ProQuest Dissertations and Theses (UMI No. 9231100).

[cl21152-bib-0594] Weller, L. D. , Carpenter, S. , & Holmes, C. T. (1998). Achievement gains of low‐achieving students using computer‐assisted vs regular instruction. Psychological Reports, 83(3. 834‐834.

[cl21152-bib-0595] Westbury, M. (1994). The effect of elementary grade retention on subsequent school achievement and ability. Canadian Journal of Education, 19(3), 241–250.

[cl21152-bib-0596] White, R. N. , Williams, I. J. , & Haslem, M. B. (2005). Performance of District 23 students participating in Scholastic READ 180. Washington, DC: Policy Studies Associates.

[cl21152-bib-0597] Whyte, J. (1993). Longitudinal correlates and outcomes of initial reading progress for a sample of Belfast boys. European Journal of Psychology of Education, 8(3), 325–340.

[cl21152-bib-0598] Wilczynski, E. L. (2006). *Reading and self‐esteem in at‐risk students* (Doctoral dissertation). Available from ProQuest Information & Learning (UMI No. 3206265).

[cl21152-bib-0599] Williams, J. P. , Nubla‐Kung, A. M. , Pollini, S. , Stafford, K. B. , Garcia, A. , & Snyder, A. E. (2007). Teaching cause—effect text structure through social studies content to at‐risk second graders. Journal of Learning Disabilities, 40(2), 111–120. 17380986 10.1177/00222194070400020201

[cl21152-bib-0600] Williams, P. (1998). *An investigation of the influences of home‐school collaboration on children's achievement* (Doctoral dissertation). Available from ProQuest Information & Learning (UMI No. 9837664).

[cl21152-bib-0601] Williams, Y. (2012). *An evaluation of the effectiveness of integrated learning systems on urban middle school student achievement* (Doctoral dissertation). Available from ProQuest Information & Learning (UMI No. 3522727).

[cl21152-bib-0602] Woodward, J. , & Baxter, J. (1997). The effects of an innovative approach to mathematics on academically low‐achieving students in inclusive settings. Exceptional Children, 63(3), 373–388.

[cl21152-bib-0603] Woodward, J. , & Brown, C. (2006). Meeting the curricular needs of academically low‐achieving students in middle grade mathematics. Journal of Special Education, 40(3), 151–159.

[cl21152-bib-0604] Wright, J. , & Jacobs, B. (2003). Teaching phonological awareness and metacognitive strategies to children with reading difficulties: A comparison of two instructional methods. Educational Psychology, 23(1), 17–47.

[cl21152-bib-0605] Young, C. , Valadez, C. , & Gandara, C. (2016). Using performance methods to enhance students' reading fluency. Journal of Educational Research, 109(6), 624–630.

[cl21152-bib-0606] Ysseldyke, J. , Betts, J. , Thill, T. , & Hannigan, E. (2004). Use of an instructional management system to improve mathematics skills for students in Title I programs. Preventing School Failure: Alternative Education for Children and Youth, 48(4), 10–14.

[cl21152-bib-0607] Zentall, S. S. , & Lee, J. (2012). A reading motivation intervention with differential outcomes for students at risk for reading disabilities, ADHD, and typical comparisons: “Clever Is and Clever Does”. Learning Disability Quarterly, 35(4), 248–259.

[cl21152-bib-0608] Zeuschner, M. S. (2005). *Phonemic awareness through fluent auditory discrimination and the effects on decoding skills of learning‐disabled students* (Doctoral dissertation). Central Connecticut State University.

[cl21152-bib-0609] Abadie, A. (2005). Semiparametric difference‐in‐differences estimators. Review of Economic Studies, 72(1), 1–19.

[cl21152-bib-0610] Ackerman, P. T. , Dykman, R. A. , Holloway, C. , Paal, N. P. , & Gocio, M. Y. (1991). A trial of piracetam in two subgroups of students with dyslexia enrolled in summer tutoring. Journal of Learning Disabilities, 24(9), 542–549. 1765729 10.1177/002221949102400906

[cl21152-bib-0611] Alexander, K. L. , Entwisle, D. R. , & Olson, L. S. (2001). Schools, achievement, and inequality: A seasonal perspective. Education Evaluation and Policy Analysis, 23(2), 171–191.

[cl21152-bib-0612] Alfieri, L. , Brooks, P. J. , Aldrich, N. J. , & Tenenbaum, H. R. (2011). Does discovery‐based instruction enhance learning? Journal of Educational Psychology, 103(1), 1–18.

[cl21152-bib-0613] Al Otaiba, S. , Connor, C. M. , Sidler Folsom, J. , Greulich, L. , Meadows, J. , & Li, Z. (2011). Assessment data‐informed guidance to individualize kindergarten reading instruction: Findings from a cluster‐randomized control field trial. Elementary School Journal, 111(4), 535–560. 21818158 10.1086/659031 PMC3147177

[cl21152-bib-0614] Allinder, R. M. , Dunse, L. , Brunken, C. D. , & Obermiller‐Krolikowski, H. J. (2001). Improving fluency in at‐risk readers and students with learning disabilities. Remedial and Special Education, 22(1), 48–54.

[cl21152-bib-0615] Almond, D. , Currie, J. , & Duque, V. (2018). Childhood circumstances and adult outcomes: Act II. Journal of Economic Literature, 56(4), 1360–1446.

[cl21152-bib-0616] Andrews, I. , & Kasy, M. (2019). Identification of and correction for publication bias. American Economic Review, 109(8), 2766–2794.

[cl21152-bib-0617] Archambault, I. , Eccles, J. S. , & Vida, M. N. (2010). Ability self‐concepts and subjective value in literacy: Joint trajectories from grades 1 through 12. Journal of Educational Psychology, 102(4), 804–812.

[cl21152-bib-0618] Bailey, D. , Duncan, G. J. , Odgers, C. L. , & Yu, W. (2017). Persistence and fadeout in the impacts of child and adolescent interventions. Journal of Research on Educational Effectiveness, 10(1), 7–39. 29371909 10.1080/19345747.2016.1232459 PMC5779101

[cl21152-bib-0619] Bandura, A. (1977). Social learning theory. Prentice‐Hall.

[cl21152-bib-0620] Bandura, A. (1986). Social foundations of thought and action: A social cognitive theory. Prentice‐Hall.

[cl21152-bib-0621] Berktold, J. , Geis, S. , & Kaufman, P. (1998). *Subsequent educational attainment of high school dropouts* (Postsecondary Education Descriptive Analysis Reports. Statistical Analysis Report. National Education Longitudinal Study 1988). National Center for Education Statistics (ED), Washington, DC. Retrieved from http://nces.ed.gov/pubs98/98085.pdf

[cl21152-bib-0622] Berridge, D. , Brodie, I. , Pitts, J. , Porteous, D. , & Tarling, R. (2001). *The independent effects of permanent exclusion from school on the offending careers of young people* (Occasional Paper No 71). Research, Development and Statistics Directorate, UK. http://troublesofyouth.pbworks.com/f/occ71-exclusion.pdf

[cl21152-bib-0623] Björklund, A. , & Salvanes, K. (2011). Education and family background. In E. A. Hanushek , S. Machin & L. Woessmann (Eds.), Handbook of the Economics of Education (3, pp. 201–247). Amsterdam: Elsevier.

[cl21152-bib-0624] Borenstein, M. , Hedges, L. V. , Higgins, J. P. T. , & Rothstein, H. R. (2009). Introduction to meta‐analysis. John Wiley & Sons Ltd.

[cl21152-bib-0625] Borenstein, M. , Higgins, J. P. , Hedges, L. V. , & Rothstein, H. R. (2017). Basics of meta‐analysis: I^2^ is not an absolute measure of heterogeneity. Research Synthesis Methods, 8(1), 5–18. 28058794 10.1002/jrsm.1230

[cl21152-bib-0626] Bradley, R. H. , & Corwyn, R. F. (2002). Socioeconomic status and child development. Annual Review of Psychology, 53, 371–399. 10.1146/annurev.psych.53.100901.135233 11752490

[cl21152-bib-0627] Brook, J. S. , Stimmel, M. A. , Zhang, C. , & Brook, D. W. (2008). The association between earlier marijuana use and subsequent academic achievement and health problems: A longitudinal study. American Journal on Addictions, 17, 155–160. 18393060 10.1080/10550490701860930 PMC3638839

[cl21152-bib-0628] Bruner, J. S. (2006). In search of pedagogy, Volume I: The selected works of Jerome Bruner, 1957‐1978 (1). Routledge.

[cl21152-bib-0629] Burchinal, M. , McCartney, K. , Steinberg, L. , Crosnoe, R. , Friedman, S. L. , McLoyd, V. , & Pianta, R. (2011). Examining the Black–White achievement gap among low‐income children using the NICHD study of early child care and youth development. Child Development, 82(5), 1404–1420. 21790543 10.1111/j.1467-8624.2011.01620.x

[cl21152-bib-0630] Bus, A. G. , Van IJzendoorn, M. H. , & Pellegrini, A. D. (1995). Joint book reading makes for success in learning to read: A meta‐analysis on intergenerational transmission of literacy. Review of educational research, 65(1), 1–21.

[cl21152-bib-0631] Campbell, S. B. , Shaw, D. S. , & Gilliom, M. (2000). Early externalizing behavior problems: Toddlers and preschoolers at risk for later maladjustment. Development and Psychopathology, 12(3), 467–488. 11014748 10.1017/s0954579400003114

[cl21152-bib-0632] Castles, A. , Rastle, K. , & Nation, K. (2018). Ending the reading wars: Reading acquisition from novice to expert. Psychological Science in the Public Interest, 19(1), 5–51. 29890888 10.1177/1529100618772271

[cl21152-bib-0633] Chetty, R. , Friedman, J. N. , Hendren, N. , Jones, M. R. , & Porter, S. R. (2018). *The Opportunity Atlas: Mapping the childhood roots of social mobility* (NBER Working Paper, no. 25147). Cambridge, MA: National Bureau of Economic Research.

[cl21152-bib-0634] Chetty, R. , Hendren, N. , & Katz, L. F. (2016). The effects of exposure to better neighborhoods on children: New evidence from the Moving to Opportunity experiment. American Economic Review, 106(4), 855–902. 29546974 10.1257/aer.20150572

[cl21152-bib-0635] Cheung, A. , & Slavin, R. (2013). The effectiveness of educational technology applications for enhancing mathematics achievement in K‐12 classrooms: A meta‐analysis. Educational Research Review, 9, 88–113.

[cl21152-bib-0636] Cheung, A. C. , & Slavin, R. E. (2012). How features of educational technology applications affect student reading outcomes: A meta‐analysis. Educational Research Review, 7(3), 198–215.

[cl21152-bib-0637] Compton, D. L. , Gilbert, J. K. , Jenkins, J. R. , Fuchs, D. , Fuchs, L. S. , Cho, E. , Barquero, L. A. , & Bouton, B. (2012). Accelerating chronically unresponsive children to tier 3 instruction: What level of data is necessary to ensure selection accuracy? Journal of Learning Disabilities, 45(3), 204–216. 22491810 10.1177/0022219412442151

[cl21152-bib-0638] Conti, G. , Heckman, J. J. , & Pinto, R. (2016). The effects of two influential early childhood interventions on health and healthy behavior. Economic Journal, 126, F28–F65. 28260805 10.1111/ecoj.12420 PMC5331750

[cl21152-bib-0639] Currie, J. (2009). Healthy, wealthy, and wise: Socioeconomic status, poor health in childhood and human capital development. Journal of Economic Literature, 47(1), 87–122.

[cl21152-bib-0640] Deater‐Deckard, K. , Dodge, K. A. , Bates, J. E. , & Pettit, G. S. (1998). Multiple risk factors in the development of externalizing behavior problems: Group and individual differences. Development and Psychopathology, 10(3), 469–493. 9741678 10.1017/s0954579498001709 PMC2776047

[cl21152-bib-0641] de Boer, H. , Donker, A. S. , & van der Werf, M. P. (2014). Effects of the attributes of educational interventions on students’ academic performance: A meta‐analysis. Review of educational research, 84(4), 509–545.

[cl21152-bib-0642] De Ridder, K. A. A. , Pape, K. , Johnsen, R. , Westin, S. , Holmen, T. L. , & Bjørngaard, J. H. (2012). School dropout: A major public health challenge: A 10‐year prospective study on medical and non‐medical social insurance benefits in young adulthood, the Young‐HUNT 1 Study (Norway). Journal of Epidemiology & Community Health, 66(11), 995–1000. 22315238 10.1136/jech-2011-200047

[cl21152-bib-0643] Dexter, D. D. , & Hughes, C. A. (2011). Graphic organizers and students with learning disabilities: A meta‐analysis. Learning Disability Quarterly, 34(1), 51–72.

[cl21152-bib-0644] Dietrichson, J. , Bøg, M. , Eiberg, M. , Filges, T. , & Klint Jørgensen, A.‐M. (2016). Protocol for a systematic review: Targeted school‐based interventions for improving reading and mathematics for students with or at risk of academic difficulties in grade K‐6: A systematic review. Campbell Systematic Reviews, 12(1), 1–60. 10.1002/cl2.1152 PMC8356298 37131926

[cl21152-bib-0645] Dietrichson, J. , Bøg, M. , Filges, T. , & Klint Jørgensen, A.‐M. (2017). Academic interventions for elementary and middle school students with low socioeconomic status: A systematic review and meta‐analysis. Review of Educational Research, 87(2), 243–282.

[cl21152-bib-0646] Dietrichson, J. , Filges, T. , Klokker, R. H. , Viinholt, B. C. , Bøg, M. , & Jensen, U. H. (2020). Targeted school‐based interventions for improving reading and mathematics for students with, or at risk of, academic difficulties in Grades 7‐12: A systematic review. Campbell Systematic Reviews, 16(2), e1081. 37131416 10.1002/cl2.1081 PMC8356291

[cl21152-bib-0647] Duncan, G. J. , Dowsett, C. J. , Claessens, A. , Magnuson, K. , Huston, A. C. , Klebanov, P. , Pagani, L. S. , Feinstein, L. , Engel, M. , Brooks‐Gunn, J. , Sexton, H. , Duckworth, K. , & Japel, C. (2007). School readiness and later achievement. Developmental Psychology, 43(6), 1428–1446. 18020822 10.1037/0012-1649.43.6.1428

[cl21152-bib-0648] Durlak, J. A. , Weissberg, R. P. , Dymnicki, A. B. , Taylor, R. D. , & Schellinger, K. B. (2011). The impact of enhancing students’ social and emotional learning: A meta‐analysis of school‐based universal interventions. Child Development, 82(1), 405–432. 21291449 10.1111/j.1467-8624.2010.01564.x

[cl21152-bib-0649] Edmonds, M. S. , Vaughn, S. , Wexler, J. , Reutebuch, C. , Cable, A. , Klingler Tackett, K. , & Wick Schnakenberg, J. (2009). A synthesis of reading interventions and effects on reading comprehension outcomes for older struggling readers. Review of Educational Research, 79(1), 262–300. 20072704 10.3102/0034654308325998 PMC2804990

[cl21152-bib-0650] Egger, M. , Smith, G. D. , Schneider, M. , & Minder, C. (1997). Bias in meta‐analysis detected by a simple, graphical test. BMJ, 315, 629–634. 9310563 10.1136/bmj.315.7109.629 PMC2127453

[cl21152-bib-0651] Elbaum, B. , Vaughn, S. , Tejero Hughes, M. , & Watson Moody, S. (2000). How effective are one‐to‐one tutoring programs in reading for elementary students at risk for reading failure? A meta‐analysis of the intervention research. Journal of Educational Psychology, 92(4), 605–619.

[cl21152-bib-0652] Ensminger, M. E. , & Slausarcick, A. L. (1992). Paths to high school graduation or dropout: A longitudinal study of a first‐grade cohort. Sociology of Education, 65(2), 95–113.

[cl21152-bib-0653] Esping‐Andersson, G. , Garfinkel, I. , Han, W.‐J. , Magnuson, K. , Wagner, S. , & Waldfogel, J. (2012). Child care and school performance in Denmark and the United States. Children and Youth Services Review, 34, 576–589. 24163491 10.1016/j.childyouth.2011.10.010 PMC3806146

[cl21152-bib-0654] Fantuzzo, J. W. , King, J. A. , & Heller, L. R. (1992). Effects of reciprocal peer tutoring on mathematics and school adjustment: A component analysis. Journal of Educational Psychology, 84(3), 331–339.

[cl21152-bib-0655] Feinstein, L. , Sabates, R. , Anderson, T. M. , Sorhaindo, A. , & Hammond, C. (2006). *Measuring the effects of education on health and civic engagement*. Proceedings of the Copenhagen Symposium. OECD. http://www.oecd.org/education/country-studies/37437718.pdf

[cl21152-bib-0656] Finn, J. B. , Gerber, S. , & Boyd‐Zaharias, J. (2005). Small classes in the early grades, academic achievement, and graduating from high school. Journal of Educational Psychology, 97(2), 214–223.

[cl21152-bib-0657] Fisher, Z. , Tipton, E. , & Zhipeng, H. (2017). *Package ‘robumeta’*. http://cran.uni-muenster.de/web/packages/robumeta/robumeta.pdf

[cl21152-bib-0658] Flynn, L. J. , Zheng, X. , & Swanson, H. L. (2012). Instructing struggling older readers: A selective meta‐analysis of intervention research. Learning Disabilities Research & Practice, 27(1), 21–32.

[cl21152-bib-0659] Forsman, H. , & Vinnerljung, B. (2012). Interventions aiming to improve school achievements of children in out‐of‐home care: A scoping review. Children and Youth Services Review, 34(6), 1084–1091.

[cl21152-bib-0660] Fortner, V. (1986). Generalization of creative productive‐thinking training to LD students' written expression. Learning Disability Quarterly, 9(4), 274–284.

[cl21152-bib-0661] Francis, D. J. , Shaywitz, S. E. , Stuebing, K. K. , Shaywitz, B. A. , & Fletcher, J. M. (1996). Developmental lag versus deficit models of reading disability: A longitudinal, individual growth curves analysis. Journal of Educational Psychology, 88(1), 3–17.

[cl21152-bib-0662] Franco, A. , Malhotra, N. , & Simonovits, G. (2014). Publication bias in the social sciences: Unlocking the file drawer. Science, 345(6203), 1502–1505. 25170047 10.1126/science.1255484

[cl21152-bib-0663] Fryer, R. G. (2014). Injecting charter school best practices into traditional public schools: Evidence from field experiments. Quarterly Journal of Economics, 129(3), 1355–1407.

[cl21152-bib-0664] Fryer, R. G. (2017). The production of human capital in developed countries: Evidence from 196 randomized field experiments, Handbook of economic field experiments (2, pp. 95–322). Amsterdam: North‐Holland.

[cl21152-bib-0665] Fryer, R. G. , & Levitt, S. D. (2013). Testing for racial differences in the mental ability of young children. American Economic Review, 103(2), 981–1005.

[cl21152-bib-0666] Fuchs, L. S. , Fuchs, D. , & Kazdan, S. (1999). Effects of peer‐assisted learning strategies on high school students with serious reading problems. Remedial and Special Education, 20(5), 309–318.

[cl21152-bib-0667] Gardner, H. (1999). Intelligence reframed: Multiple intelligences for the twenty‐first century. Basic Books.

[cl21152-bib-0668] Gardnier, H. E. , Stein, J. A. , & Jacobs, J. K. (1997). The process of dropping out of high school: A 19‐year perspective. American Educational Research Journal, 34(2), 395–419.

[cl21152-bib-0669] Gershenson, S. (2013). Do summer time‐use gaps vary by socioeconomic status? American Educational Research Journal, 50, 1219–1248.

[cl21152-bib-0670] Gersten, R. , Chard, D. J. , Jayanti, M. , Baker, S. K. , Morphy, P. , & Flojo, P. (2009). Mathematics instruction for students with learning disabilities: A meta‐analysis of instructional components. Review of Educational Research, 79(3), 1202–1242.

[cl21152-bib-0671] Glennerster, R. , & Takavarasha, K. (2013). Running randomized evaluations—A practical guide. Princeton, NJ: Princeton University Press.

[cl21152-bib-0672] Goldschmidt, P. , & Wang, J. (1999). When can schools affect dropout behavior? A longitudinal multilevel analysis. American Educational Research Journal, 36(4), 715–738.

[cl21152-bib-0673] Golinkoff, R. M. , Hoff, E. , Rowe, M. L. , Tamis‐LeMonda, C. S. , & Hirsh‐Pasek, K. (2019). Language matters: Denying the existence of the 30‐million‐word gap has serious consequences. Child Development, 90(3), 985–992. 30102419 10.1111/cdev.13128 PMC10370358

[cl21152-bib-0674] Good, C. , Aronson, J. , & Inzlicht, M. (2003). Improving adolescents' standardized test performance: An intervention to reduce the effects of stereotype threat. Journal of Applied Developmental Psychology, 24(6), 645–662.

[cl21152-bib-0675] Goodwin, A. P. , & Ahn, S. (2010). A meta‐analysis of morphological interventions: Effects on literacy achievement of children with literacy difficulties. Annals of dyslexia, 60(2), 183–208. 20799003 10.1007/s11881-010-0041-x

[cl21152-bib-0676] Guryan, J. , Christenson, S. , Claessens, A. , Engel, M. , Lai, I. , Ludwig, J. , & Turner, M. C. (2017). *The effect of mentoring on school attendance and academic outcomes: A randomized evaluation of the Check & Connect Program*. Institute for Policy Research Working Paper Series, WP‐16‐18. Evanston, IL: Northwestern University. http://www.ipr.northwestern.edu/publications/docs/workingpapers/2016/WP-16-18.pdf

[cl21152-bib-0677] Hart, B. , & Risley, T. R. (2003). The early catastrophe: The 30 million word gap by age 3. American Educator, 27(1), 4–9.

[cl21152-bib-0678] Heckman, J. J. (2006). Skill formation and the economics of investing in disadvantaged children. Science, 312, 1900–1902. 16809525 10.1126/science.1128898

[cl21152-bib-0679] Heckman, J. J. , & Karapakula, G. (2019). *Intergenerational and intragenerational externalities of the Perry Preschool Project* (No. w25889). National Bureau of Economic Research.

[cl21152-bib-0680] Heckman, J. J. , Moon, S. H. , Pinto, R. , Savelyev, P. A. , & Yavitz, A. (2010). The rate of return to the HighScope Perry Preschool Program. Journal of Public Economics, 94, 114–128. 21804653 10.1016/j.jpubeco.2009.11.001 PMC3145373

[cl21152-bib-0681] Hedges, L. V. (1992). Modeling publication selection effects in meta‐analysis. Statistical Science, 7(2), 246–255.

[cl21152-bib-0682] Hedges, L. V. (2007). Effect sizes in cluster‐randomized designs. Journal of Educational and Behavioral Statistics, 32(4), 341–370.

[cl21152-bib-0683] Hedges, L. V. , & Hedberg, E. C. (2007). Intraclass correlation values for planning group randomized trials in education. Education Evaluation and Policy Analysis, 29(1), 60–87.

[cl21152-bib-0684] Hedges, L. V. , Tipton, E. , & Johnson, M. C. (2010). Robust variance estimation in meta‐regression with dependent effect size estimates. Research Synthesis Methods, 1, 39–65. 26056092 10.1002/jrsm.5

[cl21152-bib-0685] Hedges, L. V. , & Vevea, J. L. (2005). Chapter 9: Selection method approaches. In Sutton Rothstein & Borenstein (Eds.), Publication bias in meta‐analysis: Prevention, assessment, and adjustments (pp. 145–174). John Wiley & Sons, Ltd.

[cl21152-bib-0686] Heller, S. B. , Shah, A. K. , Guryan, J. , Ludwig, J. , Mullainathan, S. , & Pollack, H. A. (2017). Thinking, fast and slow? Some field experiments to reduce crime and dropout in Chicago. Quarterly Journal of Economics, 132(1), 1–54. 29456270 10.1093/qje/qjw033 PMC5810151

[cl21152-bib-0687] Higgins, J. P. T. & Green, S. (Eds.). (2008). Cochrane handbook for systematic reviews of interventions. Version 5.0.0. The Cochrane Collaboration.

[cl21152-bib-0688] Higgins, J. P. T. & Green, S. (Eds.) (2011). *Cochrane handbook for systematic reviews of interventions*. Version 5.1.0. Wiley‐Blackwell. The Cochrane Collaboration. www.cochrane-handbook.org

[cl21152-bib-0689] Higgins, J. P. , Thompson, S. G. , Deeks, J. J. , & Altman, D. G. (2003). Measuring inconsistency in meta‐analyses. British Medical Journal, 327(7414), 557–560. 12958120 10.1136/bmj.327.7414.557 PMC192859

[cl21152-bib-0690] Hill, C. J. , Bloom, H. S. , Black, A. R. , & Lipsey, M. W. (2008). Empirical benchmarks for interpreting effect sizes in research. Child Development Perspectives, 2(3), 172–177.

[cl21152-bib-0691] Holmlund, H. , & Sund, K. (2005). *Is the gender gap in school performance affected by the sex of the teacher?* (Working paper 5/2005). Swedish Institute for Social Research (SOFI), Stockholm University.

[cl21152-bib-0692] Horwood, J. L. , Fergusson, D. M. , Hayatbakhsh, M. R. , Najman, J. M. , Coffey, C. , Patton, G. C. , Silins, E. , & Hutchinson, D. M. (2010). Cannabis use and educational achievement: Findings from three Australasian cohort studies. Drug and Alcohol Dependence, 110, 247–253. 20456872 10.1016/j.drugalcdep.2010.03.008

[cl21152-bib-0693] Huaqing, Qi, C. , & Kaiser, A. P. (2003). Behavior problems of preschool children from low‐income families: Review of the literature. Topics in Early Childhood Special Education, 23(4), 188–216.

[cl21152-bib-0694] Hyde, J. S. , Fennema, E. , & Lamon, S. J. (1990). Gender differences in mathematics performance: A meta‐analysis. Psychological Bulletin, 107(2), 139–155. 2138794 10.1037/0033-2909.107.2.139

[cl21152-bib-0695] Hyde, J. S. , & Linn, M. C. (1988). Gender differences in verbal ability: A meta‐analysis. Psychological Bulletin, 104(1), 53–69.

[cl21152-bib-0696] Inns, A. , Lake, C. , Pellegrini, M. , & Slavin, R. (2019). *A synthesis of quantitative research on programs for struggling readers in elementary schools*. http://www.bestevidence.org/word/strug_read_April_2019_full.pdf

[cl21152-bib-0697] Jeynes, W. (2012). A meta‐analysis of the efficacy of different types of parental involvement programs for urban students. Urban Education, 47(4), 706–742.

[cl21152-bib-0698] Johnson, J. , Brett, E. B. , & Deary, I. J. (2010). The pivotal role of education in the association between ability and social class attainment: A look across three generations. Intelligence, 38, 55–65.

[cl21152-bib-0699] Kang, C. Y. , Duncan, G. J. , Clements, D. H. , Sarama, J. , & Bailey, D. H. (2019). The roles of transfer of learning and forgetting in the persistence and fadeout of early childhood mathematics interventions. Journal of Educational Psychology, 111(4), 590–603. 31156273 10.1037/edu0000297 PMC6541454

[cl21152-bib-0700] Kennedy, M. M. (2016). How does professional development improve teaching? Review of educational research, 86(4), 945–980.

[cl21152-bib-0701] Kim, J. S. (2006). Effects of a voluntary summer reading intervention on reading achievement: Results from a randomized field trial. Educational Evaluation and Policy Analysis, 28(4), 335–355.

[cl21152-bib-0702] Kim, J. S. , & Quinn, D. M. (2013). The effects of summer reading on low‐income children's literacy achievement from kindergarten to grade 8: A meta‐analysis of classroom and home interventions. Review of Educational Research, 83(3), 386–431.

[cl21152-bib-0703] Kraft, M. A. , Blazar, D. , & Hogan, D. (2018). The effect of teacher coaching on instruction and achievement: A meta‐analysis of the causal evidence. Review of Educational Research, 88(4), 547–588.

[cl21152-bib-0704] Kraft, M. A. (2020). Interpreting effect sizes of education interventions. Educational Researcher, 49(4), 241–253.

[cl21152-bib-0705] Kyndt, E. , Raes, E. , Lismont, B. , Timmers, F. , Cascallar, E. , & Dochy, F. (2013). A meta‐analysis of the effects of face‐to‐face cooperative learning. Do recent studies falsify or verify earlier findings? Educational Research Review, 10, 133–149.

[cl21152-bib-0706] Langan, D. , Higgins, J. P. T. , Jackson, D. , Bowden, J. , Veroniki, A. A. , Kontopantelis, E. , Viechtbauer, W. , & Simmonds, M. (2019). A comparison of heterogeneity variance estimators in simulated random‐effects meta‐analyses. Research Synthesis Methods, 10(1), 83–98. 30067315 10.1002/jrsm.1316

[cl21152-bib-0707] Link Egger, H. , & Angold, A. (2006). Common emotional and behavioral disorders in preschool children: Presentation, nosology, epidemiology. Journal of Child Psychology and Psychiatry, 47(3/4), 313–337. 16492262 10.1111/j.1469-7610.2006.01618.x

[cl21152-bib-0708] Lipsey, M. W. , Puzio, K. , Yun, C. , Hebert, M. A. , Steinka‐Fry, K. , Cole, M. W. , Roberts, M. , Anthony, K. S. , & Busick, M. D. (2012). *Translating the statistical representation of the effects of education interventions into more readily interpretable forms* (NCSER 2013‐3000). U.S. Department of Education, National Center for Special Education Research, Institute of Education Sciences.

[cl21152-bib-0709] Lipsey, M. W. , & Wilson, D. B. (2001). Practical meta‐analysis. Applied Social Research Methods Series, v. 49.

[cl21152-bib-0710] McWhirter, B. T. , McWhirter, E. H. , McWhirter, J. J. , & McWhirter, R. J. (1994). Youth at risk: Another point of view. Journal of Counselling and Development, 73, 567–569.

[cl21152-bib-0711] McWhirter, B. T. , McWhirter, E. H. , McWhirter, J. J. , & McWhirter, R. J. (2004). At‐risk youth: A comprehensive response for counsellors, teachers, psychologists, and human services professionals. Thompson Brooks/Cole.

[cl21152-bib-0712] Morgan, P. L. , Farkas, G. , Hillemeier, M. M. , & Maczuga, S. (2012). Are minority children disproportionately represented in early intervention and early childhood special education? Educational Researcher, 41(9), 339–351. 24683265 10.3102/0013189x12459678 PMC3966630

[cl21152-bib-0713] National Reading Panel . (2000). Report of the National Reading Panel: Teaching children to read: An evidence‐based assessment of the scientific research literature on reading and its implications for reading instruction: Reports of the subgroups. National Institute of Child Health and Human Development, National Institutes of Health.

[cl21152-bib-0714] Nickow, A. , Oreopoulos, P. , & Quan, V. (2020). *The impressive effects of tutoring on preK‐12 learning: A systematic review and meta‐analysis of the experimental evidence* (NBER Working Paper, no. 27476). Cambridge, MA: National Bureau of Economic Research.

[cl21152-bib-0715] OECD . (2010a). Improving health and social cohesion through education. OECD Publishing. http://www.oecd-ilibrary.org/content/book/9789264086319-en

[cl21152-bib-0716] OECD . (2010b). *PISA 2009 results: What students know and can do—Student performance in reading, mathematics and science (Volume I)*. Retrieved from 10.1787/9789264091450-en

[cl21152-bib-0717] OECD . (2010c). *PISA 2009 results: Overcoming social background—Equity in learning opportunities and outcomes (Volume II)*. Retrieved from 10.1787/9789264091504-en

[cl21152-bib-0718] OECD . (2012). *Education at a glance 2012: Highlights*. OECD Publishing. http://www.oecd-ilibrary.org/education/education-at-a-glance-2012_eag_highlights-2012-en

[cl21152-bib-0719] OECD . (2013). PISA 2012 Results: Excellence Through Equity—Giving Every Student the Chance to Succeed (Volume II). PISA, OECD Publishing. 10.1787/9789264201132-en

[cl21152-bib-0720] OECD . (2015). Helping immigrant students to succeed at school—And beyond. OECD Publishing. https://www.oecd.org/education/Helping-immigrant-students-to-succeed-at-school-and-beyond.pdf

[cl21152-bib-0721] OECD . (2016). PISA 2015 results (Volume I): Excellence and equity in education. PISA, OECD Publishing. http://www.oecd.org/publications/pisa-2015-results-volume-i-9789264266490-en.htm

[cl21152-bib-0722] OECD . (2018). Education at a glance 2018: OECD Indicators. OECD Publishing. https://www.oecd-ilibrary.org/education/education-at-a-glance-2018_eag-2018-en

[cl21152-bib-0723] OECD . (2019). PISA 2018 results (Volume I): What students know and can do. PISA, OECD Publishing. https://www.oecd.org/pisa/publications/pisa-2018-results-volume-i-5f07c754-en.htm

[cl21152-bib-0724] Pearl, J. (2009). Causality—Models, reasoning, and inference (2nd ed.). Cambridge University Press.

[cl21152-bib-0725] Pellegrini, M. , Lake, C. , Inns, A. , & Slavin, R. E. (2018). *Effective programs in elementary mathematics: A best evidence synthesis*. http://www.bestevidence.org/word/elem_math_Oct_8_2018.pdf

[cl21152-bib-0726] Perry, W. G., Jr. (1999). Forms of intellectual and ethical development in the college years: A scheme. Jossey‐Bass Publishers.

[cl21152-bib-0727] Piaget, J. (2001). The psychology of intelligence. Routledge.

[cl21152-bib-0728] Pigott, T. D. (2009). Handling missing data. In H. Cooper , L. V. Hedges & J. C. Valentine (Eds.), The handbook of research synthesis and meta‐analysis (pp. 399–416). Russell Sage Foundation.

[cl21152-bib-0729] Pontoppidan, M. , Keilow, M. , Dietrichson, J. , Solheim, O. J. , Opheim, V. , Gustafson, S. , & Andersen, S. C. (2018). Randomised controlled trials in Scandinavian educational research. Educational Research, 60(3), 311–335.

[cl21152-bib-0730] Pustejovsky, J. E. , & Rodgers, M. A. (2019). Testing for funnel plot asymmetry of standardized mean differences. Research Synthesis Methods, 10(1), 57–71. 30506832 10.1002/jrsm.1332

[cl21152-bib-0731] Pustejovsky, J. E. (2020). *clubSandwich* (version 0.5.1). https://CRAN.R-project.org/package=clubSandwich

[cl21152-bib-0732] RAND Reading Study Group . (2002). Reading for understanding: Toward an R&D program in reading comprehension. Rand Corporation.

[cl21152-bib-0733] Randolph, K. A. , Fraser, M. W. , & Orthner, D. K. (2004). Educational resilience among youth at risk. Substance Use and Misuse, 39(5), 747–767. 15202807 10.1081/ja-120034014

[cl21152-bib-0734] Reljić, G. , Ferring, D. , & Martin, R. (2015). A Meta‐Analysis on the effectiveness of bilingual programs in Europe. Review of Educational Research, 85(1), 92–128.

[cl21152-bib-0735] Reynolds, A. J. , Magnuson, K. A. , & Ou, S.‐R. (2010). Preschool‐to‐third grade programs and practices: A review of research. Child and Youth Services Review, 32, 1121–1131. 10.1016/j.childyouth.2009.10.017 PMC5033058 27667887

[cl21152-bib-0736] Reynolds, A. J. , & Temple, J. A. (2008). Cost‐effective early childhood development programs from preschool to third grade. Annual review of clinical psychology, 4, 109–139. 10.1146/annurev.clinpsy.3.022806.091411 18370615

[cl21152-bib-0737] Ritter, G. , Albin, G. , Barnett, J. , Blankenship, V. , & Denny, G. (2006). The effectiveness of volunteer tutoring programs: A systematic review. Campbell Systematic Reviews, 2, 7–63.

[cl21152-bib-0738] Robinson, D. R. , Schofield, J. W. , & Steers‐Wentzell, K. L. (2005). Peer and cross‐age tutoring in math: outcomes and their design implications. Educational Psychology Review, 17(4), 327–362.

[cl21152-bib-0739] Rodgers, M. A. , & Pustejovsky, J. E. (2020). Evaluating meta‐analytic methods to detect selective reporting in the presence of dependent effect sizes. *Psychological Methods*. Advance online publication. 10.1037/met0000300 32673040

[cl21152-bib-0740] Rosenthal, R. (1979). The file drawer problem and tolerance for null results. Psychological Bulletin, 86(3), 638–641.

[cl21152-bib-0741] Rossin‐Slater, M. , & Wüst, M. (2019). What is the added value of preschool for poor children? Long‐term and intergenerational impacts and interactions with an infant health intervention. American Economic Journal: Applied Economics, 12(3), 255–286.

[cl21152-bib-0742] Rubin, D. B. (1996). Multiple imputation after 18+ years. Journal of the American Statistical Association, 91(434), 473–489.

[cl21152-bib-0743] Ruscio, J. (2008). A probability‐based measure of effect size: Robustness to base rates and other factors. Psychological Methods, 13(1), 19–30. 18331151 10.1037/1082-989X.13.1.19

[cl21152-bib-0744] Sabates, R. , Feinstein, L. , & Shingal, A. (2013). Inequality in academic performance and juvenile convictions: An area‐based analysis. British Journal of Educational Studies, 59(2), 143–158.

[cl21152-bib-0745] Sánchez‐Meca, J. , Marín‐Martínez, F. , & Chacón‐Moscoso, S. (2003). Effect‐size indices for dichotomized outcomes in meta‐analysis. Psychological Methods, 8(4), 448–467. 14664682 10.1037/1082-989X.8.4.448

[cl21152-bib-0746] Scammaca, N. K. , Roberts, G. , Vaughn, S. , & Stuebing, K. K. (2015). A meta‐analysis of interventions for struggling readers in grades 4‐12: 1980‐2011. Journal of Learning Disabilities, 48(4), 369–390. 24092916 10.1177/0022219413504995 PMC3975734

[cl21152-bib-0747] Scruggs, T. , & Mastropieri, M. A. (1986). Improving the test‐taking skills of behaviorally disordered and learning disabled children. Exceptional Children, 53(1), 63–68. 3743609 10.1177/001440298605300107

[cl21152-bib-0748] Scott, M. A. , & Bernhardt, A. (2000). *Pathways to educational attainment and their effect on early career development* (IEE Brief, n28). Institute on Education and the Economy, Teacher's College, Columbia University. http://nrccte.education.louisville.edu/sites/default/files/publication-files/pathways_to_ed_attainment.pdf

[cl21152-bib-0749] Sirin, S. R. (2005). Socioeconomic status and academic achievement: A meta‐analytic review of research. Review of Educational Research, 75(3), 417–453.

[cl21152-bib-0750] Slates, S. L. , Alexander, K. L. , Entwisle, D. R. , & Olson, L. S. (2012). Counteracting summer slide: Social capital resources within socio‐economically disadvantaged families. Journal of Education for Students Placed at Risk, 17, 165–185.

[cl21152-bib-0751] Slavin, R. E. , & Lake, C. (2008). Effective programs in elementary mathematics: A best‐evidence synthesis. Review of educational research, 78(3), 427–515.

[cl21152-bib-0752] Slavin, R. E. , Lake, C. , Chambers, B. , Cheung, A. , & Davis, S. (2009). Effective reading programs for the elementary grades: A best‐evidence synthesis. Review of educational research, 79(4), 1391–1466.

[cl21152-bib-0753] Slavin, R. , Lake, C. , Davis, S. , & Madden, N. (2011). Effective programs for struggling readers: A best‐evidence synthesis. Educational Research Review, 6, 1–26.

[cl21152-bib-0754] Slavin, R. E. , & Madden, N. (2011). Measures inherent to treatments in program effectiveness reviews. Journal of Research on Educational Effectiveness, 4, 370–380.

[cl21152-bib-0755] Sternberg, R. J. (2009). The essential Sternberg: Essays on intelligence, psychology, and education. Springer Publishing Company.

[cl21152-bib-0756] Sterne, J. A. , Becker, B. J. , & Egger, M. (2005). Chapter 5: The funnel plot. In H. R. Rothstein , A. J. Sutton & M. Borenstein (Eds.), Publication bias in meta‐analysis: Prevention, assessment and adjustments (pp. 75–98). John Wiley & Sons, Ltd.

[cl21152-bib-0757] Stoet, G. , & Geary, D. C. (2013). Sex differences in mathematics and reading achievement are inversely related: Within‐ and across‐nation assessment of 10 years of PISA data. PLOS One, 8(3), e57988. 23516422 10.1371/journal.pone.0057988 PMC3596327

[cl21152-bib-0758] Suggate, S. P. (2016). A meta‐analysis of the long‐term effects of phonemic awareness, phonics, fluency, and reading comprehension interventions. Journal of Learning Disabilities, 49(1), 77–96. 24704662 10.1177/0022219414528540

[cl21152-bib-0759] Sullivan, G. M. (2011). Getting off the “gold standard”: Randomized controlled trials and education research. Journal of Graduate Medical Education, 3(3), 285–289. 22942950 10.4300/JGME-D-11-00147.1 PMC3179209

[cl21152-bib-0760] Tanner‐Smith, E. E. , & Tipton, E. (2014). Robust variance estimation with dependent effect sizes: Practical considerations including a software tutorial in Stata and SPSS. Research Synthesis Methods, 5(1), 13–30. 26054023 10.1002/jrsm.1091

[cl21152-bib-0761] Tanner‐Smith, E. E. , Tipton, E. , & Polanin, J. R. (2016). Handling complex meta‐analytic data structures using robust variance estimates: A tutorial in R. Journal of Developmental and Life‐Course Criminology, 2(1), 85–112.

[cl21152-bib-0762] Taylor, R. D. , Oberle, E. , Durlak, J. A. , & Weissberg, R. P. (2017). Promoting positive youth development through school‐based social and emotional learning interventions: A meta‐analysis of follow‐up effects. Child Development, 88(4), 1156–1171. 28685826 10.1111/cdev.12864

[cl21152-bib-0763] The Council of the European Union . (2009). Strategic framework for European cooperation in education and training (‘ET 2020’). Official Journal of the European Union. http://eur-lex.europa.eu/LexUriServ/LexUriServ.do?uri=OJ:C:2009:119:0002:0010:EN:PDF

[cl21152-bib-0764] Thomas, J. , Cook, T. D. , Klein, A. , Starkey, P. , & DeFlorio, L. (2018). The sequential scale‐up of an evidence‐based intervention: A case study. Evaluation Review, 42(3), 318–357. 30081667 10.1177/0193841X18786818

[cl21152-bib-0765] Thompson, S. G. , & Higgins, J. P. T. (2002). How should meta‐regression analyses be undertaken and interpreted? Statistics in Medicine, 21, 1559–1573. 12111920 10.1002/sim.1187

[cl21152-bib-0766] Timperley, H. S. , & Phillips, G. (2003). Changing and sustaining teachers’ expectations through professional development in literacy. Teaching and Teacher Education, 19, 627–641.

[cl21152-bib-0767] Tidwell, R. , & Corona Garret, S. (1994). Youth at risk: In search of a definition. Journal of Counselling and Development, 72, 444–446.

[cl21152-bib-0768] Tipton, E. (2015). Small sample adjustments for robust variance estimation with meta‐regression. Psychological Methods, 20(3), 375–393. 24773356 10.1037/met0000011

[cl21152-bib-0769] Tipton, E. , & Pustejovsky, J. E. (2015). Small‐sample adjustments for tests of moderators and model fit using robust variance estimation in meta‐regression. Journal of Educational and Behavioral Statistics, 40(6), 604–634.

[cl21152-bib-0770] UNESCO . (1994). The Salamanca statement and framework for action on special needs education. Salamanca.

[cl21152-bib-0771] U.S. Congress . (2002). *No child left behind act of 2001*. http://www2.ed.gov/policy/elsec/leg/esea02/107-110.pdf

[cl21152-bib-0772] U.S. Department of Education . (2004). *No child left behind act of 2001*. http://www2.ed.gov/nclb/overview/intro/execsumm.pdf

[cl21152-bib-0773] Van Buuren, S. , & Groothuis‐Oudshoorn, K. (2011). mice: Multivariate imputation by chained equations in R. Journal of Statistical Software, 45(3), 1–67.

[cl21152-bib-0774] Viechtbauer, W. (2010). Conducting meta‐analyses in R with the metafor package. Journal of Statistical Software, 36(3), 1–48.

[cl21152-bib-0775] von Hippel, P. T. , Workman, J. , & Downey, D. B. (2018). Inequality in reading and math skills forms mainly before kindergarten: A replication, and partial correction, of “Are Schools the Great Equalizer?”. Sociology of Education, 91(4), 323–357. 38818352 10.1177/0038040718801760 PMC11139062

[cl21152-bib-0776] Vygotsky, L. S. (1978). Mind in society: The development of higher psychological processes. Cambridge, MA: Harvard University Press.

[cl21152-bib-0777] What Works Clearinghouse . (2014). *Procedures and Standards Handbook Version 3.0*. https://ies.ed.gov/ncee/wwc/Docs/referenceresources/wwc_procedures_v3_0_standard-_handbook.pdf

[cl21152-bib-0778] Wanzek, J. , Stevens, E. A. , Williams, K. J. , Scammacca, N. , Vaughn, S. , & Sargent, K. (2018). Current evidence on the effects of intensive early reading interventions. Journal of Learning Disabilities, 51(6), 612–624. 29779424 10.1177/0022219418775110 PMC6247899

[cl21152-bib-0779] Wanzek, J. , & Vaughn, S. (2007). Research‐based implications from extensive early reading interventions. School psychology review, 36, 541–561.

[cl21152-bib-0780] Wanzek, J. , Vaughn, S. , Scammacca, N. , Gatlin, B. , Walker, M. A. , & Capin, P. (2016). Meta‐analyses of the effects of tier 2 type reading interventions in grades K‐3. Educational Psychology Review, 28(3), 551–576. 27594774 10.1007/s10648-015-9321-7 PMC5007082

[cl21152-bib-0781] Wanzek, J. , Vaughn, S. , Wexler, J. , Swanson, E. A. , Edmonds, M. , & Kim, A.‐H. (2006). A synthesis of spelling and reading interventions and their effects on the spelling outcomes of students with LD. Journal of Learning Disabilities, 39(2), 528–543. 17165620 10.1177/00222194060390060501

[cl21152-bib-0782] Wanzek, J. , Vaughn, S. , Scammacca, N. , Metz, K. , Murray, C. , Roberts, G. , & Danielson, L. (2013). Extensive reading interventions for students with reading difficulties after Grade 3. Review of Educational Research, 83, 163–195.

[cl21152-bib-0783] Wasik, B. , & Slavin, S. (1993). Preventing early reading failure with one‐to‐one tutoring: A review of five programs. Reading Research Quarterly, 28(2), 178–200.

[cl21152-bib-0784] Wasik, B. A. (1997). Volunteer tutoring programs: Do we know what works? Phi Delta Kappan, 79(4), 282–287.

[cl21152-bib-0785] White, K. R. (1982). The relation between socioeconomic status and academic achievement. Psychological Bulletin, 91(3), 461–481.

[cl21152-bib-0786] Wilson, S. , Lipsey, M. W. , Tanner‐Smith, E. E. , Huang, C. , & Steinka‐Fry, K. (2010). Protocol: Dropout prevention and intervention programs: Effects on school completion and dropout among school‐aged children and youth. Campbell Systematic Reviews, 6(1), 1–35.

[cl21152-bib-0787] Wilson, S. J. , Tanner‐Smith, E. E. , Lipsey, M. W. , Steinka‐Fry, K. , & Morrison, J. (2011). Dropout prevention and intervention programs: Effects on school completion and dropout among school‐aged children and youth. Campbell Systematic Reviews, 7(1), 1–61.

[cl21152-bib-0788] Winding, T. N. , Nohr, E. A. , Labriola, M. , Biering, M. , & Andersen, J. H. (2013). Personal predictors of educational attainment after compulsory school: Influence of measures of vulnerability, health, and school performance. Scandinavian Journal of Public Health, 41, 92–101. 23221378 10.1177/1403494812467713

[cl21152-bib-0789] Zief, S. G. , Lauver, S. , & Maynard, R. A. (2006). Impacts of after‐school programs on student outcomes. Campbell Systematic Reviews, 2(1), 1–51.

