# Supplementary material for: Targeted school‐based interventions for improving reading and mathematics for students with or at risk of academic difficulties in Grades K‐6: A systematic review
Source: Campbell Syst Rev. 2021 Apr 6;17(2):e1152. doi: 10.1002/cl2.1152 (PMC8356298; doi:10.1002/cl2.1152)
Supplement: Supplementary file 1 — Supporting information [file CL2-17-e1152-s007.pdf]

Online Appendix A: Search strategy by database

The following figure shows the search results for each bibliographic database searched in the original 2016 search and the 2018 update.

Database	Results in March 2016	Results in July 2018
Academic Search Premier (EBSCO-host)	1,548	759
ERIC (EBSCO-host)	5,862	422
PsycINFO (EBSCO-host)	5,505	1,065
SocIndex (EBSCO-host)	328	91
British Education Index (EBSCO-host)	96	No access in 2018
Teacher Reference Center (EBSCO-host)	643	188
ECONLIT (EBSCO-host)	58	16
FRANCIS (EBSCO-host)	165	No access in 2018
Dissertation & theses A&I (ProQuest-host)	786	No access in 2018
CBCA Education (ProQuest-host)	97	No access in 2018
Australian Education Index (ProQuest-host)	85	No access in 2018
Social Science Citation Index (WOS)	2,376	907
Science Citation Index (WOS)	Searched with SSCI	Searched with SSCI
Medline (OVID-host)	323	0
Embase (OVID-host)	Searched with Medline	Searched with Medline

Search stings for electronic databases

The individual search strings used to search the electronic databases are reported below. Most of the reported search strings are from the 2018 update. The search strings used in the update were identical to the original search strings from the 2016 search.

As mentioned in the deviations from protocol section in the review, the accessibility of some of the electronic databases changed in the period between the original and the updated search. For the searches that were not updated in 2018, or had no new results, we have reported the original search string from 2016.

Academic Search Premier

Searched through EBSCO-host interface. Search performed 2018/25/06. Search modes - Boolean/Phrase. Limiters - Date Published: 2016/01/03 - 2018/25/06.

Search	Search Terms	Results
S42	S20 AND S41	759
S41	S21 OR S22 OR S23 OR S24 OR S25 OR S26 OR S27 OR S28 OR S29 OR S30 OR S31 OR S32 OR S33 OR S34 OR S35 OR S36 OR S37 OR S38 OR S39 OR S40	617,127
S40	TI (regression N1 discontinuity OR difference-in-difference* OR event N1 stud* OR interrupted time serie* OR instrumental variable* OR waitlist control*) OR AB (regression N1 discontinuity OR difference-in-difference* OR event N1 stud* OR interrupted time serie* OR instrumental variable* OR waitlist control*)	13,232

S39	TI ((control N5 case) OR (control N5 subject*) OR (control N5 group*) OR (control N5 patient*) OR (control N5 intervention)) OR AB ((control N5 case) OR (control N5 subject*) OR (control N5 group*) OR (control N5 patient*) OR (control N5 intervention))	406,468
S38	TI ((treatment N5 case) OR (treatment N5 subject*) OR (treatment N5 group*) OR (treatment N5 patient*) OR (treatment N5 intervention)) OR AB ((treatment N5 case) OR (treatment N5 subject*) OR (treatment N5 group*) OR (treatment N5 patient*) OR (treatment N5 intervention))	364,183
S37	TI ((experiment* N5 case) OR (experiment* N5 subject*) OR (experiment* N5 group*) OR (experiment* N5 patient*) OR (experiment* N5 intervention)) OR AB ((experiment* N5 case) OR (experiment* N5 subject*) OR (experiment* N5 group*) OR (experiment* N5 patient*) OR (experiment* N5 intervention))	77,447
S36	((assign* N5 case) OR (assign* N5 subject*) OR (assign* N5 group*) OR (assign* N5 patient*) OR (assign* N5 intervention)) OR AB ((assign* N5 case) OR (assign* N5 subject*) OR (assign* N5 group*) OR (assign* N5 patient*) OR (assign* N5 intervention))	47,070
S35	TI ((intervention N5 case) OR (intervention N5 subject*) OR (intervention N5 group*) OR (intervention N5 patient*)) OR AB ((intervention N5 case) OR (intervention N5 subject*) OR (intervention N5 group*) OR (intervention N5 patient*))	80,066
S34	TI (quasi-experiment* OR quasiexperiment* OR Propensity score* OR (compar* N1 group*) OR (match* N1 control*) OR (match* N1 group*) OR (match* N1 compar*) OR experiment* trial* OR experiment* design* OR experiment* method* OR experiment* stud* OR experiment* evaluation* OR experiment* test* OR experiment* assessment* OR assessment only OR (comparison N1 samp*) OR propensity match* OR (Between N1 group*)) OR AB (quasi-experiment* OR quasiexperiment* OR Propensity score* OR (compar* N1 group*) OR (match* N1 control*) OR (match* N1 group*) OR (match* N1 compar*) OR experiment* trial* OR experiment* design* OR experiment* method* OR experiment* stud* OR experiment* evaluation* OR experiment* test* OR experiment*assessment* OR assessment only OR (comparison N1 samp*) OR propensity match* OR (Between N1 group*))	671,758
S33	TI ((random* N2 trial*) OR RCT) OR AB ((random* N2 trial*) OR RCT)	146,516
S32	TI Non-random* OR nonradom* OR (non N1 random*) OR AB Non-random* OR Nonrandom* OR (non N1 random*)	19,686
S31	TI ((Propensity score* OR (match* N1 control*) OR (match* N1 compar*) OR assessment only OR comparison samp* OR propensity match*)) OR AB ((Propensity score* OR (match* N1 control*) OR (match* N1 compar*) OR assessment only OR comparison samp* OR propensity match*))	75,636
S30	TI assign* N3 (subject* OR patient*) OR AB assign* N3 (subject* OR patient*)	15,465
S29	TI (quasi-experiment* OR quasiexperiment* OR experiment*) OR AB (quasi-experiment* OR quasiexperiment* OR experiment*)	1,897,100
S28	TI Intervention* N1 Stud* OR AB Intervention* N1 Stud*	20,333
S27	TI ((prospective N2 study) OR AB (prospective N2 study)) OR (TI retrospective OR AB retrospective)	299,219
S26	TI longitudinal OR AB longitudinal OR TI observational OR AB observational	241,040
S25	TI (epidemiologic N2 study) OR AB (epidemiologic N2 study) OR TI (follow up OR followup) N2 study OR AB (follow up OR followup) N2 study)	33,112
S24	TI cross sectional OR AB cross sectional	162,455
S23	TI ((case control) OR AB (case control)) OR TI cohort OR AB cohort	296,927
S22	DE "Cohort analysis" OR DE "Case Study"	31,611
S21	AB randomized OR AB placebo OR AB randomly OR trial OR AB groups	2,813,093
S20	S5 AND S12 AND S19	1,842
S19	S6 OR S7 OR S8 OR S9 OR S10 OR S11 OR S12 OR S13 OR S14 OR S15 OR S16 OR S17 OR S18	227,565

S18	transfer* N2 effect	7,211
S17	Writ*	782,474
S16	Numeracy OR Mathematic* OR Math	1,330,548
S15	DE "Mathematics" OR DE "Numeracy"	71,599
S14	Reading OR Literacy	308,484
S13	DE "Reading" OR DE "Literacy"	33,263
S12	S6 OR S7 OR S8 OR S9 OR S10 OR S11	9,299
S11	Intellect* N2 develop*	11,571
S10	DE "Intellectual Development"	1,435
S9	School N1 (performan* OR achiev*)	35,657
S8	Academic* N2 (performance* OR achiev* OR abilit* OR outcome*)	47,766
S7	Learn* N2 (disab* OR Problem*)	37,567
S6	DE "Academic Achievement" OR DE "Academic Ability" OR DE "Learning Problems" OR (DE "Learning Disabilities")	40,235
S5	S1 OR S2 OR S3 OR S4	19,437
S4	(Student* OR pupil*) N3 (Learn* N2 (disab* OR Problem*))	3,838
S3	(Child* N2 (placed N1 care)) OR ((DE "Foster Care") AND child*)	1,380
S2	((Primary N1 School) N3 (Student* OR pupil*)) OR ((Elementary N1 School) N3 (Student* OR pupil*)) OR (DE "Elementary School Students") OR ((Secondary N1 school) OR (high N2 school) OR (middle N1 School) N3 (student* OR pupil*))	265,175
S1	(Underachiev* OR Under N1 achiev* OR lowachiev* OR low N1 achiev* OR Low N1 perform* OR lowperform* OR (at-risk OR at N1 risk)) N1 (student* OR pupil*) OR ((high-risk OR high N1 risk) N1 (student* OR pupil*)) OR ((Special N1 Need*) N1 (Student* OR pupil*)) OR ((Low N1 income) N1 (student* OR pupil*))	12,388

ERIC (Education Resources Information Center)

Searched through EBSCO-host interface. Search performed 2018/25/06. Search modes - Boolean/Phrase. Limiters - Date Published: 2016/01/03 - 2018/25/06.

Search	Search Terms	Results
S63	S41 AND S62	422
S62	S42 OR S43 OR S44 OR S45 OR S46 OR S47 OR S48 OR S49 OR S50 OR S51 OR S52 OR S53 OR S54 OR S55 OR S56 OR S57 OR S58 OR S59 OR S60 OR S61	17,316
S61	TI (regression N1 discontinuity OR difference-in-difference* OR event N1 stud* OR interrupted time serie* OR instrumental variable* OR waitlist control*) OR AB (regression N1 discontinuity OR difference-in-difference* OR event N1 stud* OR interrupted time serie* OR instrumental variable* OR waitlist control*)	2,059
S60	TI ((control N5 case) OR (control N5 subject*) OR (control N5 group*) OR (control N5 patient*) OR (control N5 intervention)) OR AB ((control N5 case) OR (control N5 subject*) OR (control N5 group*) OR (control N5 patient*) OR (control N5 intervention))	22,676

S59	TI ((treatment N5 case) OR (treatment N5 subject*) OR (treatment N5 group*) OR (treatment N5 patient*) OR (treatment N5 intervention)) OR AB ((treatment N5 case) OR (treatment N5 subject*) OR (treatment N5 group*) OR (treatment N5 patient*) OR (treatment N5 intervention))	10,313
S58	TI ((experiment* N5 case) OR (experiment* N5 subject*) OR (experiment* N5 group*) OR (experiment* N5 patient*) OR (experiment* N5 intervention)) OR AB ((experiment* N5 case) OR (experiment* N5 subject*) OR (experiment* N5 group*) OR (experiment* N5 patient*) OR (experiment* N5 intervention))	14,860
S57	TI ((intervention N5 case) OR (intervention N5 subject*) OR (intervention N5 group*) OR (intervention N5 patient*)) OR AB ((intervention N5 case) OR (intervention N5 subject*) OR (intervention N5 group*) OR (intervention N5 patient*))	6,016
S56	((assign* N5 case) OR (assign* N5 subject*) OR (assign* N5 group*) OR (assign* N5 patient*) OR (assign* N5 intervention)) OR AB ((assign* N5 case) OR (assign* N5 subject*) OR (assign* N5 group*) OR (assign* N5 patient*) OR (assign* N5 intervention))	7,399
S55	TI (quasi-experiment* OR quasiexperiment* OR Propensity score* OR (compar* N1 group*) OR (match* N1 control*) OR (match* N1 group*) OR (match* N1 compar*) OR experiment* trial* OR experiment* design* OR experiment* method* OR experiment* stud* OR experiment* evaluation* OR experiment* test* OR experiment* assessment* OR assessment only OR (comparison N1 samp*) OR propensity match* OR (Between N1 group*)) OR AB (quasi-experiment* OR quasiexperiment* OR Propensity score* OR (compar* N1 group*) OR (match* N1 control*) OR (match* N1 group*) OR (match* N1 compar*) OR experiment* trial* OR experiment* design* OR experiment* method* OR experiment* stud* OR experiment* evaluation* OR experiment* test* OR experiment*assessment* OR assessment only OR (comparison N1 samp*) OR propensity match* OR (Between N1 group*))	47,587
S54	TI ((random* N2 trial*) OR RCT) OR AB ((random* N2 trial*) OR RCT)	3,287
S53	TI Non-random* OR nonradom* OR (non N1 random*) OR AB Non-random* OR Nonrandom* OR (non N1 random*)	704
S52	TI ((Propensity score* OR (match* N1 control*) OR (match* N1 compar*) OR assessment only OR comparison samp* OR propensity match*)) OR AB ((Propensity score* OR (match* N1 control*) OR (match* N1 compar*) OR assessment only OR comparison samp* OR propensity match*))	4,737
S51	TI assign* N3 (subject* OR patient*) OR AB assign* N3 (subject* OR patient*)	1,066
S50	TI (quasi-experiment* OR quasiexperiment* OR experiment*) OR AB (quasi-experiment* OR quasiexperiment* OR experiment*)	78,215
S49	TI Intervention* N1 Stud* OR AB Intervention* N1 Stud*	3,913
S48	TI ((prospective n2 study) OR AB (prospective n2 study)) OR (TI retrospective OR AB retrospective)	4,749
S47	TI longitudinal OR AB longitudinal OR TI observational OR AB observational	29,654
S46	TI (epidemiologic N2 study) OR AB (epidemiologic N2 study) OR TI (follow up OR followup) N2 study OR AB (follow up OR followup) N2 study	4,513
S45	TI cross sectional OR AB cross sectional	4,989
S44	TI ((case control) OR AB (case control)) OR TI cohort OR AB cohort	11,928
S43	DE "Cohort analysis" OR DE "Case Studies"	60,360
S42	AB randomized OR AB placebo OR AB randomly OR trial OR AB groups	244,168
S41	S26 AND S33 AND S40	858
S40	S34 OR S35 OR S36 OR S37 OR S38 OR S39	12,270
S39	transfer* N2 effect	549
S38	Writ* OR DE "Writing Ability" OR DE "Writing Achievement"	141,881

S37	Numeracy OR Mathematic* OR Math	122,339
S36	DE "Mathematics" OR DE "Numeracy"	13,685
S35	Reading OR Literacy	193,254
S34	DE "Reading" OR DE "Literacy"	20,099
S33	S27 OR S28 OR S29 OR S30 OR S31 OR S32	4,526
S32	Intellect* N2 develop*	6,579
S31	DE "Intellectual Development"	3,320
S30	School N1 (performan* OR achiev*)	10,680
S29	Academic* N2 (performance* OR achiev* OR abilit* OR outcome*)	93,615
S28	Learn* N2 (disab* OR Problem*)	34,678
S27	DE "Academic Achievement" OR DE "Academic Ability" OR DE "Learning Problems" OR DE "Learning Disabilities"	102,715
S26	S22 OR S23 OR S24 OR S25	11,243
S25	(Student* OR pupil*) N3 (Learn* N2 (disab* OR Problem*))	7,982
S24	(Child* N2 (placed N1 care)) OR ((DE "Foster Care") AND child*)	1,881
S23	((Primary N1 School) N3 (Student* OR pupil*)) OR ((Elementary N1 School) N3 (Student* OR pupil*)) OR (DE "Elementary School Students") OR ((Secondary N1 school) OR (high N2 school) OR (middle N1 School) N3 (student* OR pupil*))	254,280
S22	(Underachiev* OR Under N1 achiev* OR lowachiev* OR low N1 achiev* OR Low N1 perform* OR lowperform* OR (at-risk OR at N1 risk)) N1 (student* OR pupil*) OR ((high-risk OR high N1 risk) N1 (student* OR pupil*)) OR ((Special N1 Need*) N1 (Student* OR pupil*)) OR ((Low N1 income) N1 (student* OR pupil*))	32,696
S21	S1 OR S2 OR S3 OR S4 OR S5 OR S6 OR S7 OR S8 OR S9 OR S10 OR S11 OR S12 OR S13 OR S14 OR S15 OR S16 OR S17 OR S18 OR S19 OR S20	14,138
S20	TI (regression N1 discontinuity OR difference-in-difference* OR event N1 stud* OR interrupted time serie* OR instrumental variable* OR waitlist control*) OR AB (regression N1 discontinuity OR difference-in-difference* OR event N1 stud* OR interrupted time serie* OR instrumental variable* OR waitlist control*)	175
S19	TI ((control N5 case) OR (control N5 subject*) OR (control N5 group*) OR (control N5 patient*) OR (control N5 intervention)) OR AB ((control N5 case) OR (control N5 subject*) OR (control N5 group*) OR (control N5 patient*) OR (control N5 intervention))	1,291
S18	TI ((treatment N5 case) OR (treatment N5 subject*) OR (treatment N5 group*) OR (treatment N5 patient*) OR (treatment N5 intervention)) OR AB ((treatment N5 case) OR (treatment N5 subject*) OR (treatment N5 group*) OR (treatment N5 patient*) OR (treatment N5 intervention))	390
S17	TI ((experiment* N5 case) OR (experiment* N5 subject*) OR (experiment* N5 group*) OR (experiment* N5 patient*) OR (experiment* N5 intervention)) OR AB ((experiment* N5 case) OR (experiment* N5 subject*) OR (experiment* N5 group*) OR (experiment* N5 patient*) OR (experiment* N5 intervention))	921
S16	TI ((intervention N5 case) OR (intervention N5 subject*) OR (intervention N5 group*) OR (intervention N5 patient*)) OR AB ((intervention N5 case) OR (intervention N5 subject*) OR (intervention N5 group*) OR (intervention N5 patient*))	447
S15	((assign* N5 case) OR (assign* N5 subject*) OR (assign* N5 group*) OR (assign* N5 patient*) OR (assign* N5 intervention)) OR AB ((assign* N5 case) OR (assign* N5 subject*) OR (assign* N5 group*) OR (assign* N5 patient*) OR (assign* N5 intervention))	349

S14	TI (quasi-experiment* OR quasiexperiment* OR Propensity score* OR (compar* N1 group*) OR (match* N1 control*) OR (match* N1 group*) OR (match* N1 compar*) OR experiment* trial* OR experiment* design* OR experiment* method* OR experiment* stud* OR experiment* evaluation* OR experiment* test* OR experiment* assessment* OR assessment only OR (comparison N1 samp*) OR propensity match* OR (Between N1 group*)) OR AB (quasi-experiment* OR quasiexperiment* OR Propensity score* OR (compar* N1 group*) OR (match* N1 control*) OR (match* N1 group*) OR (match* N1 compar*) OR experiment* trial* OR experiment* design* OR experiment* method* OR experiment* stud* OR experiment* evaluation* OR experiment* test* OR experiment*assessment* OR assessment only OR (comparison N1 samp*) OR propensity match* OR (Between N1 group*))	2,795
S13	TI ((random* N2 trial*) OR RCT) OR AB ((random* N2 trial*) OR RCT)	338
S12	TI Non-random* OR nonradom* OR (non N1 random*) OR AB Non-random* OR Nonrandom* OR (non N1 random*)	43
S11	TI ((Propensity score* OR (match* N1 control*) OR (match* N1 compar*) OR assessment only OR comparison samp* OR propensity match*)) OR AB ((Propensity score* OR (match* N1 control*) OR (match* N1 compar*) OR assessment only OR comparison samp* OR propensity match*))	259
S10	TI assign* N3 (subject* OR patient*) OR AB assign* N3 (subject* OR patient*)	23
S9	TI (quasi-experiment* OR quasiexperiment* OR experiment*) OR AB (quasi-experiment* OR quasiexperiment* OR experiment*)	3,443
S8	TI Intervention* N1 Stud* OR AB Intervention* N1 Stud*	354
S7	TI (prospective N2 study) OR AB (prospective N2 study) OR TI retrospective OR AB retrospective	240
S6	TI longitudinal OR AB longitudinal OR TI observational OR AB observational	1,452
S5	TI (epidemiologic N2 study) OR AB (epidemiologic N2 study) OR TI (follow up OR followup) N2 study OR AB (follow up OR followup) N2 study	73
S4	TI cross sectional OR AB cross sectional	398
S3	TI case control OR AB case control OR TI cohort OR AB cohort	848
S2	DE "Cohort analysis"	272
S1	AB randomized OR AB placebo OR AB randomly OR trial OR AB groups	10,078

PsycINFO

Searched through EBSCO-host interface. Search performed 2018/25/06. Search modes - Boolean/Phrase. Limiters - Date Published: 2016/01/03 - 2018/25/06.

Search	Search Terms	Results
S42	S20 AND S41	1,065
S41	S21 OR S22 OR S23 OR S24 OR S25 OR S26 OR S27 OR S28 OR S29 OR S30 OR S31 OR S32 OR S33 OR S34 OR S35 OR S36 OR S37 OR S38 OR S39 OR S40	105,107
S40	TI (regression N1 discontinuity OR difference-in-difference* OR event N1 stud* OR interrupted time serie* OR instrumental variable* OR waitlist control*) OR AB (regression N1 discontinuity OR difference-in-difference* OR event N1 stud* OR interrupted time serie* OR instrumental variable* OR waitlist control*)	7,722

S39	TI ((control N5 case) OR (control N5 subject*) OR (control N5 group*) OR (control N5 patient*) OR (control N5 intervention)) OR AB ((control N5 case) OR (control N5 subject*) OR (control N5 group*) OR (control N5 patient*) OR (control N5 intervention))	163,830
S38	TI ((treatment N5 case) OR (treatment N5 subject*) OR (treatment N5 group*) OR (treatment N5 patient*) OR (treatment N5 intervention)) OR AB ((treatment N5 case) OR (treatment N5 subject*) OR (treatment N5 group*) OR (treatment N5 patient*) OR (treatment N5 intervention))	141,556
S37	TI ((experiment* N5 case) OR (experiment* N5 subject*) OR (experiment* N5 group*) OR (experiment* N5 patient*) OR (experiment* N5 intervention)) OR AB ((experiment* N5 case) OR (experiment* N5 subject*) OR (experiment* N5 group*) OR (experiment* N5 patient*) OR (experiment* N5 intervention))	50,402
S36	((assign* N5 case) OR (assign* N5 subject*) OR (assign* N5 group*) OR (assign* N5 patient*) OR (assign* N5 intervention)) OR AB ((assign* N5 case) OR (assign* N5 subject*) OR (assign* N5 group*) OR (assign* N5 patient*) OR (assign* N5 intervention))	26,086
S35	TI ((intervention N5 case) OR (intervention N5 subject*) OR (intervention N5 group*) OR (intervention N5 patient*)) OR AB ((intervention N5 case) OR (intervention N5 subject*) OR (intervention N5 group*) OR (intervention N5 patient*))	47,982
S34	TI (quasi-experiment* OR quasiexperiment* OR Propensity score* OR (compar* N1 group*) OR (match* N1 control*) OR (match* N1 group*) OR (match* N1 compar*) OR experiment* trial* OR experiment* design* OR experiment* method* OR experiment* stud* OR experiment* evaluation* OR experiment* test* OR experiment* assessment* OR assessment only OR (comparison n1 samp*) OR propensity match* OR (Between N1 group*)) OR AB (quasi-experiment* OR quasiexperiment* OR Propensity score* OR (compar* N1 group*) OR (match* N1 control*) OR (match* N1 group*) OR (match* N1 compar*) OR experiment* trial* OR experiment* design* OR experiment* method* OR experiment* stud* OR experiment* evaluation* OR experiment* test* OR experiment*assessment* OR assessment only OR (comparison N1 samp*) OR propensity match* OR (Between N1 group*))	235,705
S33	TI ((random* N2 trial*) OR RCT) OR AB ((random* N2 trial*) OR RCT)	45,390
S32	TI Non-random* OR nonradom* OR (non N1 random*) OR AB Non-random* OR Nonrandom* OR (non N1 random*)	4,483
S31	TI ((Propensity score* OR (match* N1 control*) OR (match* N1 compar*) OR assessment only OR comparison samp* OR propensity match*)) OR AB ((Propensity score* OR (match* N1 control*) OR (match* N1 compar*) OR assessment only OR comparison samp* OR propensity match*))	40,798
S30	TI assign* N3 (subject* OR patient*) OR AB assign* N3 (subject* OR patient*)	5,453
S29	TI (quasi-experiment* OR quasiexperiment* OR experiment*) OR AB (quasi-experiment* OR quasiexperiment* OR experiment*)	392,188
S28	TI Intervention* N1 Stud* OR AB Intervention* N1 Stud*	13,466
S27	TI ((prospective n2 study) OR AB (prospective n2 study)) OR (TI retrospective OR AB retrospective)	57,507
S26	TI longitudinal OR AB longitudinal OR TI observational OR AB observational	119,545
S25	TI (epidemiologic N2 study) OR AB (epidemiologic N2 study))OR TI (follow up OR followup) N2 study OR AB (follow up OR followup) N2 study	19,981
S24	TI cross sectional OR AB cross sectional	64,810
S23	TI ((case control) OR AB (case control)) OR TI cohort OR AB cohort	79,607
S22	DE "Cohort analysis" OR DE "Case Study"	1,263
S21	AB randomized OR AB placebo OR AB randomly OR trial OR AB groups	959,807
S20	S5 AND S12 AND S19	2,307
S19	S6 OR S7 OR S8 OR S9 OR S10 OR S11 OR S12 OR S13 OR S14 OR S15 OR S16 OR S17 OR S18	27,248

S18	transfer* N2 effect	2,650
S17	Writ*	150,277
S16	Numeracy OR Mathematic* OR Math	120,399
S15	DE "Mathematics" OR DE "Numeracy"	15,991
S14	Reading OR Literacy	160,294
S13	DE "Reading" OR DE "Literacy"	38,744
S12	S6 OR S7 OR S8 OR S9 OR S10 OR S11	8,523
S11	Intellect* N2 develop*	47,886
S10	DE "Intellectual Development"	2,906
S9	School N1 (performan* OR achiev*)	12,552
S8	Academic* N2 (performance* OR achiev* OR abilit* OR outcome*)	102,020
S7	Learn* N2 (disab* OR Problem*)	41,045
S6	DE "Academic Achievement" OR DE "Academic Ability" OR DE "Learning Problems" OR DE "Learning Disabilities"	72,815
S5	S1 OR S2 OR S3 OR S4	9,425
S4	(Student* OR pupil*) N3 (Learn* N2 (disab* OR Problem*))	7,083
S3	(Child* N2 (placed N1 care)) OR ((DE "Foster Care") AND child*)	4,875
S2	((Primary N1 School) N3 (Student* OR pupil*)) OR ((Elementary N1 School) N3 (Student* OR pupil*)) OR (DE "Elementary School Students") OR ((Secondary N1 school) OR (high N2 school) OR (middle N1 School) N3 (student* OR pupil*))	155,307
S1	(Underachiev* OR Under N1 achiev* OR lowachiev* OR low N1 achiev* OR Low N1 perform* OR lowperform* OR (at-risk OR at N1 risk)) N1 (student* OR pupil*) OR ((high-risk OR high N1 risk) N1 (student* OR pupil*)) OR ((Special N1 Need*) N1 (Student* OR pupil*)) OR ((Low N1 income) N1 (student* OR pupil*))	10,329

Socindex

Searched through EBSCO-host interface. Search performed 2018/25/06. Search modes - Boolean/Phrase. Limiters - Date Published: 2016/01/03 - 2018/25/06.

Search	Search Terms	Results
S42	S20 AND S41	91
S41	S21 OR S22 OR S23 OR S24 OR S25 OR S26 OR S27 OR S28 OR S29 OR S30 OR S31 OR S32 OR S33 OR S34 OR S35 OR S36 OR S37 OR S38 OR S39 OR S40	15,096
S40	TI (regression N1 discontinuity OR difference-in-difference* OR event N1 stud* OR interrupted time serie* OR instrumental variable* OR waitlist control*) OR AB (regression N1 discontinuity OR difference-in-difference* OR event N1 stud* OR interrupted time serie* OR instrumental variable* OR waitlist control*)	2,256
S39	TI ((control N5 case) OR (control N5 subject*) OR (control N5 group*) OR (control N5 patient*) OR (control N5 intervention)) OR AB ((control N5 case) OR (control N5 subject*) OR (control N5 group*) OR (control N5 patient*) OR (control N5 intervention))	17,748

S38	TI ((treatment N5 case) OR (treatment N5 subject*) OR (treatment N5 group*) OR (treatment N5 patient*) OR (treatment N5 intervention)) OR AB ((treatment N5 case) OR (treatment N5 subject*) OR (treatment N5 group*) OR (treatment N5 patient*) OR (treatment N5 intervention))	18,744
S37	TI ((experiment* N5 case) OR (experiment* N5 subject*) OR (experiment* N5 group*) OR (experiment* N5 patient*) OR (experiment* N5 intervention)) OR AB ((experiment* N5 case) OR (experiment* N5 subject*) OR (experiment* N5 group*) OR (experiment* N5 patient*) OR (experiment* N5 intervention))	7,625
S36	((assign* N5 case) OR (assign* N5 subject*) OR (assign* N5 group*) OR (assign* N5 patient*) OR (assign* N5 intervention)) OR AB ((assign* N5 case) OR (assign* N5 subject*) OR (assign* N5 group*) OR (assign* N5 patient*) OR (assign* N5 intervention))	4,356
S35	TI ((intervention N5 case) OR (intervention N5 subject*) OR (intervention N5 group*) OR (intervention N5 patient*)) OR AB ((intervention N5 case) OR (intervention N5 subject*) OR (intervention N5 group*) OR (intervention N5 patient*))	8,148
S34	TI (quasi-experiment* OR quasiexperiment* OR Propensity score* OR (compar* N1 group*) OR (match* N1 control*) OR (match* N1 group*) OR (match* N1 compar*) OR experiment* trial* OR experiment* design* OR experiment* method* OR experiment* stud* OR experiment* evaluation* OR experiment* test* OR experiment* assessment* OR assessment only OR (comparison N1 samp*) OR propensity match* OR (Between N1 group*)) OR AB (quasi-experiment* OR quasiexperiment* OR Propensity score* OR (compar* N1 group*) OR (match* N1 control*) OR (match* N1 group*) OR (match* N1 compar*) OR experiment* trial* OR experiment* design* OR experiment* method* OR experiment* stud* OR experiment* evaluation* OR experiment* test* OR experiment*assessment* OR assessment only OR (comparison N1 samp*) OR propensity match* OR (Between N1 group*))	32,956
S33	TI ((random* N2 trial*) OR RCT) OR AB ((random* N2 trial*) OR RCT)	6,233
S32	TI Non-random* OR nonradom* OR (non N1 random*) OR AB Non-random* OR Nonrandom* OR (non N1 random*)	893
S31	TI ((Propensity score* OR (match* N1 control*) OR (match* N1 compar*) OR assessment only OR comparison samp* OR propensity match*)) OR AB ((Propensity score* OR (match* N1 control*) OR (match* N1 compar*) OR assessment only OR comparison samp* OR propensity match*))	4,185
S30	TI assign* N3 (subject* OR patient*) OR AB assign* N3 (subject* OR patient*)	960
S29	TI (quasi-experiment* OR quasiexperiment* OR experiment*) OR AB (quasi-experiment* OR quasiexperiment* OR experiment*)	51,006
S28	TI Intervention* N1 Stud* OR AB Intervention* N1 Stud*	1,989
S27	TI ((prospective n2 study) OR AB (prospective n2 study)) OR (TI retrospective OR AB retrospective)	10,706
S26	TI longitudinal OR AB longitudinal OR TI observational OR AB observational	31,832
S25	TI (epidemiologic N2 study) OR AB (epidemiologic N2 study) OR TI (follow up OR followup) N2 study OR AB (follow up OR followup) N2 study	3,515
S24	TI cross sectional OR AB cross sectional	12,689
S23	TI ((case control) OR AB (case control)) OR TI cohort OR AB cohort	19,858
S22	DE "Cohort analysis" OR DE "Case Study"	2,082
S21	AB randomized OR AB placebo OR AB randomly OR trial OR AB groups	272,385
S20	S5 AND S12 AND S19	213
S19	S6 OR S7 OR S8 OR S9 OR S10 OR S11 OR S12 OR S13 OR S14 OR S15 OR S16 OR S17 OR S18	5,214
S18	transfer* N2 effect	356
S17	Writ*	74,830

S16	Numeracy OR Mathematic* OR Math	27,048
S15	DE "Mathematics" OR DE "Numeracy"	1,864
S14	Reading OR Literacy	39,382
S13	DE "Reading" OR DE "Literacy"	3,918
S12	S6 OR S7 OR S8 OR S9 OR S10 OR S11	870
S11	Intellect* N2 develop*	3,034
S10	DE "Intellectual Development"	448
S9	School N1 (performan* OR achiev*)	7,676
S8	Academic* N2 (performance* OR achiev* OR abilit* OR outcome*)	13,803
S7	Learn* N2 (disab* OR Problem*)	5,675
S6	DE "Academic Achievement" OR DE "Academic Ability" OR DE "Learning Problems" OR DE "Learning Disabilities"	10,997
S5	S1 OR S2 OR S3 OR S4	1,721
S4	(Student* OR pupil*) N3 (Learn* N2 (disab* OR Problem*))	634
S3	(Child* N2 (placed N1 care)) OR ((DE "Foster Care") AND child*)	818
S2	((Primary N1 School) N3 (Student* OR pupil*)) OR ((Elementary N1 School) N3 (Student* OR pupil*)) OR (DE "Elementary School Students") OR ((Secondary N1 school) OR (high N2 school) OR (middle N1 School) N3 (student* OR pupil*))	53,472
S1	(Underachiev* OR Under N1 achiev* OR lowachiev* OR low N1 achiev* OR Low N1 perform* OR lowperform* OR (at-risk OR at N1 risk)) N1 (student* OR pupil*) OR ((high-risk OR high N1 risk) N1 (student* OR pupil*)) OR ((Special N1 Need*) N1 (Student* OR pupil*)) OR ((Low N1 income) N1 (student* OR pupil*))	164

British Education Index

Searched through EBSCO-host interface. Search performed 2016/01/03. Search modes - Boolean/Phrase. Limiters - Date Published: 1980/01/01 - 2016/01/03.

Search	Search Terms	Results
S41	S19 AND S40	96
S40	S20 OR S21 OR S22 OR S23 OR S24 OR S25 OR S26 OR S27 OR S28 OR S29 OR S30 OR S31 OR S32 OR S33 OR S34 OR S35 OR S36 OR S37 OR S38 OR S39	13,600
S39	TI (regression N1 discontinuity OR difference-in-difference* OR event N1 stud* OR interrupted time serie* OR instrumental variable* OR waitlist control*) OR AB (regression N1 discontinuity OR difference-in-difference* OR event N1 stud* OR interrupted time serie* OR instrumental variable* OR waitlist control*)	87
S38	TI ((control N5 case) OR (control N5 subject*) OR (control N5 group*) OR (control N5 patient*) OR (control N5 intervention)) OR AB ((control N5 case) OR (control N5 subject*) OR (control N5 group*) OR (control N5 patient*) OR (control N5 intervention))	737

S37	TI ((treatment N5 case) OR (treatment N5 subject*) OR (treatment N5 group*) OR (treatment N5 patient*) OR (treatment N5 intervention)) OR AB ((treatment N5 case) OR (treatment N5 subject*) OR (treatment N5 group*) OR (treatment N5 patient*) OR (treatment N5 intervention))	248
S36	TI ((experiment* N5 case) OR (experiment* N5 subject*) OR (experiment* N5 group*) OR (experiment* N5 patient*) OR (experiment* N5 intervention)) OR AB ((experiment* N5 case) OR (experiment* N5 subject*) OR (experiment* N5 group*) OR (experiment* N5 patient*) OR (experiment* N5 intervention))	487
S35	TI ((intervention N5 case) OR (intervention N5 subject*) OR (intervention N5 group*) OR (intervention N5 patient*)) OR AB ((intervention N5 case) OR (intervention N5 subject*) OR (intervention N5 group*) OR (intervention N5 patient*))	385
S34	((assign* N5 case) OR (assign* N5 subject*) OR (assign* N5 group*) OR (assign* N5 patient*) OR (assign* N5 intervention)) OR AB ((assign* N5 case) OR (assign* N5 subject*) OR (assign* N5 group*) OR (assign* N5 patient*) OR (assign* N5 intervention)	275
S33	TI (quasi-experiment* OR quasiexperiment* OR Propensity score* OR (compar* N1 group*) OR (match* N1 control*) OR (match* N1 group*) OR (match* N1 compar*) OR experiment* trial* OR experiment* design* OR experiment* method* OR experiment* stud* OR experiment* evaluation* OR experiment* test* OR experiment* assessment* OR assessment only OR (comparison n1 samp*) OR propensity match* OR (Between N1 group*)) OR AB (quasi-experiment* OR quasiexperiment* OR Propensity score* OR (compar* N1 group*) OR (match* N1 control*) OR (match* N1 group*) OR (match* N1 compar*) OR experiment* trial* OR experiment* design* OR experiment* method* OR experiment* stud* OR experiment* evaluation* OR experiment* test* OR experiment*assessment* OR assessment only OR (comparison N1 samp*) OR propensity match* OR (Between N1 group*))	1,784
S32	TI ((random* N2 trial*) OR RCT) OR AB ((random* N2 trial*) OR RCT)	250
S31	TI Non-random* OR nonradom* OR (non N1 random*) OR AB Non-random* OR Nonrandom* OR (non N1 random*)	19
S30	TI ((Propensity score* OR (match* N1 control*) OR (match* N1 compar*) OR assessment only OR comparison samp* OR propensity match*)) OR AB ((Propensity score* OR (match* N1 control*) OR (match* N1 compar*) OR assessment only OR comparison samp* OR propensity match*))	150
S29	TI assign* N3 (subject* OR patient*) OR AB assign* N3 (subject* OR patient*)	6
S28	TI (quasi-experiment* OR quasiexperiment* OR experiment*) OR AB (quasi-experiment* OR quasiexperiment* OR experiment*)	2,939
S27	TI Intervention* N1 Stud* OR AB Intervention* N1 Stud*	216
S26	TI ((prospective N2 study) OR AB (prospective n2 study)) OR (TI retrospective OR AB retrospective)	295
S25	TI longitudinal OR AB longitudinal OR TI observational OR AB observational	1,740
S24	TI (epidemiologic N2 study) OR AB (epidemiologic N2 study) OR TI (follow up OR followup) N2 study OR AB (follow up OR followup) N2 study	152
S23	TI cross sectional OR AB cross sectional	340

S22	TI ((case control) OR AB (case control)) OR TI cohort OR AB cohort	855
S21	DE "Cohort analysis" OR DE "Case Studies"	360
S20	AB randomized OR AB placebo OR AB randomly OR trial OR AB groups	8,054
S19	S5 and S12 and S18	487
S18	S13 OR S14 OR S15 OR S16 OR S17	24,585
S17	transfer* N2 effect	36
S16	Numeracy OR Mathematic* OR Math*	12,514
S15	DE "Mathematics" OR DE "Numeracy"	2,099
S14	Reading OR Literacy	12,850
S13	DE "Reading" OR DE "Literacy"	4,323
S12	S6 OR S7 OR S8 OR S9 OR S10 OR S11	12,568
S11	Intellect* N2 develop*	1,090
S10	DE "Intellectual Development"	954
S9	School N1 (performan* OR achiev*)	967
S8	Academic* N2 (performance* OR achiev* OR abilit* OR outcome*)	5,604
S7	Learn* N2 (disab* OR Problem*)	5,763
S6	DE "Academic Achievement" OR DE "Academic Ability" OR DE "Learning Problems" OR DE "Learning Disabilities"	8,570
S5	S1 OR S2 OR S3 OR S4	26,524
S4	(Student* OR pupil*) N3 (Learn* N2 (disab* OR Problem*))	202
S3	Child* N2 placed N1 care OR (DE "foster home care") OR (DE "foster children") AND child*	118
S2	((Primary N1 School) N3 (Student* OR pupil*)) OR ((Elementary N1 School) N3 (Student* OR pupil*)) OR (DE "Elementary School Students") OR ((Secondary N1 school) OR (high N2 school) OR (middle N1 School) N3 (student* OR pupil*))	20,674
S1	(Underachiev* OR Under N1 achiev* OR lowachiev* OR low N1 achiev* OR Low N1 perform* OR lowperform* OR (at-risk OR at N1 risk)) N1 (student* OR pupil*) OR ((high-risk OR high N1 risk) N1 (student* OR pupil*)) OR ((Special N1 Need*) N1 (Student* OR pupil*)) OR ((Low N1 income) N1 (student* OR pupil*))	6,091

Teacher Reference Center

Searched through EBSCO-host interface. Search performed 2018/25/06. Search modes - Boolean/Phrase. Limiters - Date Published: 2016/01/03 - 2018/25/06.

Search	Search Terms	Results
S42	S20 AND S41	188
S41	S21 OR S22 OR S23 OR S24 OR S25 OR S26 OR S27 OR S28 OR S29 OR S30 OR S31 OR S32 OR S33 OR S34 OR S35 OR S36 OR S37 OR S38 OR S39 OR S40	2,476
S40	TI (regression N1 discontinuity OR difference-in-difference* OR event N1 stud* OR interrupted time serie* OR instrumental variable* OR waitlist control*) OR AB (regression N1 discontinuity OR difference-in-difference* OR event N1 stud* OR interrupted time serie* OR instrumental variable* OR waitlist control*)	33
S39	TI ((control N5 case) OR (control N5 subject*) OR (control N5 group*) OR (control N5 patient*) OR (control N5 intervention)) OR AB ((control N5 case) OR (control N5 subject*) OR (control N5 group*) OR (control N5 patient*) OR (control N5 intervention))	144
S38	TI ((treatment N5 case) OR (treatment N5 subject*) OR (treatment N5 group*) OR (treatment N5 patient*) OR (treatment N5 intervention)) OR AB ((treatment N5 case) OR (treatment N5 subject*) OR (treatment N5 group*) OR (treatment N5 patient*) OR (treatment N5 intervention))	46
S37	TI ((experiment* N5 case) OR (experiment* N5 subject*) OR (experiment* N5 group*) OR (experiment* N5 patient*) OR (experiment* N5 intervention)) OR AB ((experiment* N5 case) OR (experiment* N5 subject*) OR (experiment* N5 group*) OR (experiment* N5 patient*) OR (experiment* N5 intervention))	87
S36	((assign* N5 case) OR (assign* N5 subject*) OR (assign* N5 group*) OR (assign* N5 patient*) OR (assign* N5 intervention)) OR AB ((assign* N5 case) OR (assign* N5 subject*) OR (assign* N5 group*) OR (assign* N5 patient*) OR (assign* N5 intervention))	37
S35	TI ((intervention N5 case) OR (intervention N5 subject*) OR (intervention N5 group*) OR (intervention N5 patient*)) OR AB ((intervention N5 case) OR (intervention N5 subject*) OR (intervention N5 group*) OR (intervention N5 patient*))	73
S34	TI (quasi-experiment* OR quasiexperiment* OR Propensity score* OR (compar* N1 group*) OR (match* N1 control*) OR (match* N1 group*) OR (match* N1 compar*) OR experiment* trial* OR experiment* design* OR experiment* method* OR experiment* stud* OR experiment* evaluation* OR experiment* test* OR experiment* assessment* OR assessment only OR (comparison N1 samp*) OR propensity match* OR (Between N1 group*)) OR AB (quasi-experiment* OR quasiexperiment* OR Propensity score* OR (compar* N1 group*) OR (match* N1 control*) OR (match* N1 group*) OR (match* N1 compar*) OR experiment* trial* OR experiment* design* OR experiment* method* OR experiment* stud* OR experiment* evaluation* OR experiment* test* OR experiment*assessment* OR assessment only OR (comparison N1 samp*) OR propensity match* OR (Between N1 group*))	424
S33	TI ((random* N2 trial*) OR RCT) OR AB ((random* N2 trial*) OR RCT)	57

S32	TI Non-random* OR nonradom* OR (non N1 random*) OR AB Non-random* OR Nonrandom* OR (non N1 random*)	5
S31	TI ((Propensity score* OR (match* N1 control*) OR (match* N1 compar*) OR assessment only OR comparison samp* OR propensity match*)) OR AB ((Propensity score* OR (match* N1 control*) OR (match* N1 compar*) OR assessment only OR comparison samp* OR propensity match*))	41
S30	TI assign* N3 (subject* OR patient*) OR AB assign* N3 (subject* OR patient*)	4
S29	TI (quasi-experiment* OR quasiexperiment* OR experiment*) OR AB (quasi-experiment* OR quasiexperiment* OR experiment*)	586
S28	TI Intervention* N1 Stud* OR AB Intervention* N1 Stud*	62
S27	TI ((prospective N2 study) OR AB (prospective N2 study)) OR (TI retrospective OR AB retrospective)	40
S26	TI longitudinal OR AB longitudinal OR TI observational OR AB observational	204
S25	TI (epidemiologic N2 study) OR AB (epidemiologic N2 study) OR TI (follow up OR followup) N2 study OR AB (follow up OR followup) N2 study	7
S24	TI cross sectional OR AB cross sectional	104
S23	TI ((case control) OR AB (case control)) OR TI cohort OR AB cohort	144
S22	DE "Cohort analysis" OR DE "Case Study"	4
S21	AB randomized OR AB placebo OR AB randomly OR trial OR AB groups	1,611
S20	S5 AND S12 AND S19	371
S19	S6 OR S7 OR S8 OR S9 OR S10 OR S11 OR S12 OR S13 OR S14 OR S15 OR S16 OR S17 OR S18	5,070
S18	transfer* N2 effect	8
S17	Writ*	1,345
S16	Numeracy OR Mathematic* OR Math	1,834
S15	DE "Mathematics" OR DE "Numeracy"	131
S14	Reading OR Literacy	1,708
S13	DE "Reading" OR DE "Literacy"	251
S12	S6 OR S7 OR S8 OR S9 OR S10 OR S11	1,055
S11	Intellect* N2 develop*	14
S10	DE "Intellectual Development"	3
S9	School N1 (performan* OR achiev*)	568
S8	Academic* N2 (performance* OR achiev* OR abilit* OR outcome*)	791
S7	Learn* N2 (disab* OR Problem*)	224

S6	DE "Academic Achievement" OR DE "Academic Ability" OR DE "Learning Problems" OR DE "Learning Disabilities"	563
S5	S1 OR S2 OR S3 OR S4	2,961
S4	(Student* OR pupil*) N3 (Learn* N2 (disab* OR Problem*))	65
S3	(Child* N2 (placed N1 care)) OR ((DE "Foster Care") AND child*)	186
S2	((Primary N1 School) N3 (Student* OR pupil*)) OR ((Elementary N1 School) N3 (Student* OR pupil*)) OR (DE "Elementary School Students") OR ((Secondary N1 school) OR (high N2 school) OR (middle N1 School) N3 (student* OR pupil*))	2,748
S1	(Underachiev* OR Under N1 achiev* OR lowachiev* OR low N1 achiev* OR Low N1 perform* OR lowperform* OR (at-risk OR at N1 risk)) N1 (student* OR pupil*) OR ((high-risk OR high N1 risk) N1 (student* OR pupil*)) OR ((Special N1 Need*) N1 (Student* OR pupil*)) OR ((Low N1 income) N1 (student* OR pupil*))	236

ECONLIT

Searched through EBSCO-host interface. Search performed 2018/25/06. Search modes - Boolean/Phrase. Limiters - Date Published: 2016/01/03 - 2018/25/06.

Search	Search Terms	Results
S36	S7 AND S14 AND S35	16
S35	S15 OR S16 OR S17 OR S18 OR S19 OR S20 OR S21 OR S22 OR S23 OR S24 OR S25 OR S26 OR S27 OR S28 OR S29 OR S30 OR S31 OR S32 OR S33 OR S34	21,291
S34	TI (regression N1 discontinuity OR difference-in-difference* OR event N1 stud* OR interrupted time serie* OR instrumental variable* OR waitlist control*) OR AB (regression N1 discontinuity OR difference-in-difference* OR event N1 stud* OR interrupted time serie* OR instrumental variable* OR waitlist control*)	3,187
S33	TI ((control N5 case) OR (control N5 subject*) OR (control N5 group*) OR (control N5 patient*) OR (control N5 intervention)) OR AB ((control N5 case) OR (control N5 subject*) OR (control N5 group*) OR (control N5 patient*) OR (control N5 intervention))	675
S32	TI ((treatment N5 case) OR (treatment N5 subject*) OR (treatment N5 group*) OR (treatment N5 patient*) OR (treatment N5 intervention)) OR AB ((treatment N5 case) OR (treatment N5 subject*) OR (treatment N5 group*) OR (treatment N5 patient*) OR (treatment N5 intervention))	454
S31	TI ((experiment* N5 case) OR (experiment* N5 subject*) OR (experiment* N5 group*) OR (experiment* N5 patient*) OR (experiment* N5 intervention)) OR AB ((experiment* N5 case) OR (experiment* N5 subject*) OR (experiment* N5 group*) OR (experiment* N5 patient*) OR (experiment* N5 intervention))	514

S30	TI ((intervention N5 case) OR (intervention N5 subject*) OR (intervention N5 group*) OR (intervention N5 patient*)) OR AB ((intervention N5 case) OR (intervention N5 subject*) OR (intervention N5 group*) OR (intervention N5 patient*))	132
S29	((assign* N5 case) OR (assign* N5 subject*) OR (assign* N5 group*) OR (assign* N5 patient*) OR (assign* N5 intervention)) OR AB ((assign* N5 case) OR (assign* N5 subject*) OR (assign* N5 group*) OR (assign* N5 patient*) OR (assign* N5 intervention))	168
S28	TI (quasi-experiment* OR quasiexperiment* OR Propensity score* OR (compar* N1 group*) OR (match* N1 control*) OR (match* N1 group*) OR (match* N1 compar*) OR experiment* trial* OR experiment* design* OR experiment* method* OR experiment* stud* OR experiment* evaluation* OR experiment* test* OR experiment* assessment* OR assessment only OR (comparison N1 samp*) OR propensity match* OR (Between N1 group*)) OR AB (quasi-experiment* OR quasiexperiment* OR Propensity score* OR (compar* N1 group*) OR (match* N1 control*) OR (match* N1 group*) OR (match* N1 compar*) OR experiment* trial* OR experiment* design* OR experiment* method* OR experiment* stud* OR experiment* evaluation* OR experiment* test* OR experiment*assessment* OR assessment only OR (comparison N1 samp*) OR propensity match* OR (Between N1 group*))	3,152
S27	TI ((random* N2 trial*) OR RCT) OR AB ((random* N2 trial*) OR RCT)	304
S26	TI Non-random* OR nonradom* OR (non N1 random*) OR AB Non-random* OR Nonrandom* OR (non N1 random*)	121
S25	TI ((Propensity score* OR (match* N1 control*) OR (match* N1 compar*) OR assessment only OR comparison samp* OR propensity match*)) OR AB ((Propensity score* OR (match* N1 control*) OR (match* N1 compar*) OR assessment only OR comparison samp* OR propensity match*))	632
S24	TI assign* N3 (subject* OR patient*) OR AB assign* N3 (subject* OR patient*)	43
S23	TI (quasi-experiment* OR quasiexperiment* OR experiment*) OR AB (quasi-experiment* OR quasiexperiment* OR experiment*)	6,845
S22	TI (Intervention* N1 Stud*) OR AB (Intervention* N1 Stud*)	46
S21	TI ((prospective N2 study) OR AB (prospective N2 study)) OR (TI retrospective OR AB retrospective)	226
S20	TI longitudinal OR AB longitudinal OR TI observational OR AB observational	1,539
S19	TI (epidemiologic N2 study) OR AB (epidemiologic N2 study) OR TI (follow up OR followup) N2 study) OR AB (follow up OR followup) N2 study	31
S18	TI cross sectional OR AB cross sectional	1,418
S17	TI ((case control) OR AB (case control)) OR TI cohort OR AB cohort	1,122
S16	DE "Cohort analysis" OR DE "Case Studies"	0
S15	AB randomized OR AB placebo OR AB randomly OR trial OR AB groups	9,446
S14	S8 OR S9 OR S10 OR S11 OR S12 OR S13	5,259
S13	transfer* N2 effect	113

S12	Writ* OR DE "Writing Ability" OR DE "Writing Achievement"	1,176
S11	Numeracy OR Mathematic* OR Math	3,061
S10	DE "Mathematics" OR DE "Numeracy"	0
S9	Reading OR Literacy	0
S8	DE "Reading" OR DE "Literacy"	0
S7	S1 OR S2 OR S3 OR S4 OR S5 OR S6	1,096
S6	Intellect* N2 develop*	657
S5	DE "Intellectual Development"	0
S4	School N1 (performan* OR achiev*)	137
S3	Academic* N2 (performance* OR achiev* OR abilit* OR outcome*)	304
S2	Learn* N2 (disab* OR Problem*)	24
S1	DE "Academic Achievement" OR DE "Academic Ability" OR DE "Learning Problems" OR DE "Learning Disabilities"	0

FRANCIS

Searched through EBSCO-host interface. Search modes - Boolean/Phrase. Limiters - Date Published: 19800101-20160203.

**Search**	Search Terms	**Results**
S41	S19 AND S40	165
S40	S20 OR S21 OR S22 OR S23 OR S24 OR S25 OR S26 OR S27 OR S28 OR S29 OR S30 OR S31 OR S32 OR S33 OR S34 OR S35 OR S36 OR S37 OR S38 OR S39	401,117
S39	TI (regression N1 discontinuity OR difference-in-difference* OR event N1 stud* OR interrupted time serie* OR instrumental variable* OR waitlist control*) OR AB (regression N1 discontinuity OR difference-in-difference* OR event N1 stud* OR interrupted time serie* OR instrumental variable* OR waitlist control*)	1,376
S38	TI (control N5 case) OR (control N5 subject*) OR (control N5 group*) OR (control N5 patient*) OR (control N5 intervention) OR AB (control N5 case) OR (control N5 subject*) OR (control N5 group*) OR (control N5 patient*) OR (control N5 intervention)	36,547
S37	TI (treatment N5 case) OR (treatment N5 subject*) OR (treatment N5 group*) OR (treatment N5 patient*) OR (treatment N5 intervention) OR AB (treatment N5 case) OR (treatment N5 subject*) OR (treatment N5 group*) OR (treatment N5 patient*) OR (treatment N5 intervention)	25,364
S36	TI (experiment* N5 case) OR (experiment* N5 subject*) OR (experiment* N5 group*) OR (experiment* N5 patient*) OR (experiment* N5 intervention) OR AB (experiment* N5 case) OR	9,282

	(experiment* N5 subject*) OR (experiment* N5 group*) OR (experiment* N5 patient*) OR (experiment* N5 intervention)	
S35	TI (intervention N5 case) OR (intervention N5 subject*) OR (intervention N5 group*) OR (intervention N5 patient*) OR AB (intervention N5 case) OR (intervention N5 subject*) OR (intervention N5 group*) OR (intervention N5 patient*)	6,806
S34	(assign* N5 case) OR (assign* N5 subject*) OR (assign* N5 group*) OR (assign* N5 patient*) OR (assign* N5 intervention) OR AB (assign* N5 case) OR (assign* N5 subject*) OR (assign* N5 group*) OR (assign* N5 patient*) OR (assign* N5 intervention)	4,633
S33	TI ((quasi-experiment* OR quasiexperiment* OR Propensity score* OR (compar* N1 group*) OR (match* N1 control*) OR (match* N1 group*) OR (match* N1 compar*) OR experiment* trial* OR experiment* design* OR experiment* method* OR experiment* stud* OR experiment* evaluation* OR experiment* test* OR experiment* assessment* OR assessment only OR (comparison N1 samp*) OR propensity match* OR (Between N1 group*))) OR AB ((quasi-experiment* OR quasiexperiment* OR Propensity score* OR (compar* N1 group*) OR (match* N1 control*) OR (match* N1 group*) OR (match* N1 compar*) OR experiment* trial* OR experiment* design* OR experiment* method* OR experiment* stud* OR experiment* evaluation* OR experiment* test* OR experiment* assessment* OR assessment only OR (comparison N1 samp*) OR propensity match* OR (Between N1 group*)))	48,429
S32	AB (((random* N2 trial*) OR RCT)) OR TI (((random* N2 trial*) OR RCT))	8,822
S31	TI (Non-random* OR nonradom* OR (non N1 random*)) OR AB (Non-random* OR nonradom* OR (non N1 random*))	456
S30	TI (((Propensity score* OR (match* N1 control*) OR (match* N1 compar*) OR assessment only OR comparison samp* OR propensity match*))) OR AB (((Propensity score* OR (match* N1 control*) OR (match* N1 compar*) OR assessment only OR comparison samp* OR propensity match*)))	10,546
S29	TI (assign* N3 (subject* or patient*)) OR AB (assign* N3 (subject* or patient*))	1,707
S28	TI (quasi-experiment* OR quasiexperiment* OR experiment*) OR AB (quasi-experiment* OR quasiexperiment* OR experiment*)	92,283
S27	TI Intervention* N1 Stud* OR AB Intervention* N1 Stud*	1,582
S26	TI (prospective N2 stud*) OR AB (prospective N2 stud*) OR TI retrospective OR AB retrospective	16,639
S25	TI longitudinal OR AB longitudinal OR TI observational OR AB observational	25,456
S24	TI epidemiologic N2 study OR AB epidemiologic N2 study OR TI (follow up OR followup) N2 stud* OR AB (follow up OR followup) N2 stud*	4,981
S23	TI cross sectional OR AB cross sectional	11,411
S22	AB case control OR TI case control OR AB cohort OR TI cohort	18,411
S21	DE "Cohort analysis" or DE "Case study" OR DE “Case studies”	33,980
S20	AB randomized OR placebo OR random* OR trial* OR group*	234,375
S19	S5 AND S12 AND S18	363

S18	S13 OR S14 OR S15 OR S16 OR S17	25,441
S17	TI transfer* N2 effect* OR AB transfer* N2 effect*	554
S16	AB (Numeracy OR Mathematic* OR Math*) AND TI (Numeracy OR Mathematic* OR Math*)	4,798
S15	DE "Mathematics" OR DE "Numeracy"	7,926
S14	TI (Reading OR Literacy) AND AB (Reading OR Literacy)	5,103
S13	DE "Reading" OR DE "Literacy"	12,790
S12	S6 OR S7 OR S8 OR S9 OR S10 OR S11	17,156
S11	TI Intellect* N2 develop* OR AB Intellect* N2 develop*	1,132
S10	DE "Intellectual Development"	3,281
S9	TI School N1 (performan* or achiev*) OR AB School N1 (performan* or achiev*)	1,512
S8	TI Academic* N2 (performance* OR achiev* OR abilit* OR outcome*) OR AB Academic* N2 (performance* OR achiev* OR abilit* OR outcome*)	3,857
S7	AB (Learn* N2 (disab* OR problem*)) OR TI (Learn* N2 (disab* OR problem*))	4,293
S6	DE "Academic Achievement" or DE "Academic Ability" or DE "Learning Problems" OR DE “learning proces or DE "Learning Disabilities" OR DE “learning disability”	6,205
S5	S2 OR S3 OR S4	15,309
S4	TI (Student* OR pupil*) N3 (Learn* N2 (disab* OR Problem*)) OR AB (Student* OR pupil*) N3 (Learn* N2 (disab* OR Problem*))	710
S3	TI ((Child* N2 placed N1 care) OR (DE "Foster Care") AND child*) OR AB ((Child* N2 placed N1 care) OR (DE "Foster Care") AND child*)	202
S2	((Primary N1 school*) N3 (Student* OR pupil*)) OR ((Elementary N1 school) N3 (Student* OR pupil*)) OR (DE "Elementary School Students") OR (DE “Elementary students” OR ((Secondary N1 school*) OR (high N2 school*) OR (middle N1 school*) N3 (student* OR pupil*)))	14,485
S1	(Underachiev* OR Under N1 achiev* OR lowachiev* OR low N1 achiev* OR Low N1 perform* OR lowperform* OR (at-risk or at N1 risk)) N1 (student* OR pupil*) OR ((high-risk OR high N1 risk) N1 (student* OR pupil*)) OR ((Special N1 Need*) N1 (Student* OR pupil*)) OR ((Low N1 income) N1 (student* OR pupil*))	770

Dissertations and Theses A&I

Search string reported in the grey literature section.

CBCA Education

Searched through ProQuest interface. Searched 02/03/2016. Limiters - Date Published: 01/01/1980 - 2016/02/03.

Search	Search Terms	Results
S1	TI ((Underachiev* OR Under NEAR/1 (achiev* or lowachiev*)) OR (low NEAR/2 (achiev*)) OR (Low NEAR/1 (perform* OR lowperform*)) OR ((at-risk OR at NEAR/1 (risk)) NEAR/1 (student* OR pupil*)) OR ((high-risk OR high NEAR/1 (risk)) NEAR/1 (student* OR pupil*)) OR ((Special NEAR/1 (Need*)) NEAR/1 (Student* OR pupil*)) OR ((Low NEAR/1 (income)) NEAR/1 (student* OR pupil*)))	203
S2	TI(((Primary NEAR/1 (School)) NEAR/3 (Student* OR pupil*)) OR ((Elementary NEAR/1 (School)) NEAR/3 (Student* OR pupil*)) OR ((Secondary NEAR/1 (school*)) OR (high NEAR/2 (school*)) OR (middle NEAR/2 (School*)) NEAR/3 (student* OR pupil*)))	1612
S3	TI((Child* NEAR/2 (placed) NEAR/1 (care)))	1
S4	((SU("Foster Care")) AND child*)	39
S5	TI((Student* OR pupil* NEAR/3 (Learn*) NEAR/2 (disab* OR problem*)))	6839
S6	(TI((Underachiev* OR Under NEAR/1 (achiev* OR lowachiev*)) OR (low NEAR/2 (achiev*)) OR (Low NEAR/1 (perform* OR lowperform*)) OR ((at-risk OR at NEAR/1 (risk)) NEAR/1 (student* OR pupil*)) OR ((high-risk OR high NEAR/1 (risk)) NEAR/1 (student* OR pupil*)) OR ((Special NEAR/1 (Need*)) NEAR/1 (Student* OR pupil*)) OR ((Low NEAR/1 (income)) NEAR/1 (student* OR pupil*)))) OR TI(((Primary NEAR/1 (School)) CBCA Education NEAR/1 (School)) NEAR/3 (Student* OR pupil*)) OR ((Elementary NEAR/1 (School)) NEAR/3 (Student* OR pupil*)) OR ((Secondary NEAR/1 (school*)) OR (high NEAR/2 (school*)) OR (middle NEAR/2 (School*)) NEAR/3 (student* OR pupil*))) OR TI((Child* NEAR/2 (placed) NEAR/1 (care))) OR ((SU("Foster Care")) AND child*) OR (TI((Student* OR pupil* NEAR/3 (Learn*) NEAR/2 (disab* OR problem*))))	8017
S7	(SU("Academic Achievement") OR SU("academic achievement gaps") OR SU("Learning") OR SU("Learning Disabilities"))	9482
S8	TI((Learn* NEAR/2 (disab* OR Problem*)))	360
S9	TI((Academic* NEAR/2 (performance* OR achiev* OR abilit* OR outcome*)))	113
S10	TI((School NEAR/1 (performan* OR achiev*)))	56
S11	TI((Intellect* NEAR/2 (develop*)))	15
S12	(SU("Academic Achievement") OR SU("academic achievement gaps") OR SU("Learning") OR SU("Learning Disabilities")) OR TI((Learn* NEAR/2 (disab* OR Problem*))) OR TI((Academic* NEAR/2 (performance* OR achiev* OR abilit* OR outcome*))) OR TI((School NEAR/1 (performan* OR achiev*))) OR TI((Intellect* NEAR/2 (develop*)))	9618
S13	SU("Reading") OR SU("literacy")	4527
S14	SU("Mathematics") OR SU("Mathematics education")	2406
S15	TI((transfer* NEAR/2 (effect)))	1
S16	S13 OR S14 OR S15	6888
S17	S6 AND S12 AND S16	91

S18	(AB(randomized) OR AB(placebo) OR AB(randomly) OR AB(trial) OR AB(groups))	3741
S19	SU("Cohort") OR SU("case studies")	199
S20	TI(case control) OR AB(case control) OR TI(cohort) OR AB(cohort)	268
S21	TI(cross sectional) OR AB(cross sectional)	23
S22	TI(epidemiologic NEAR/2 study) OR AB(epidemiologic NEAR/2 study)	0
S23	TI(followup NEAR/2 study) OR AB(followup NEAR/2 study)	0
S24	TI(longitudinal) OR AB(longitudinal) OR TI(observational) OR AB(observational)	163
S25	(TI(prospective NEAR/2 study) OR AB(prospective NEAR/2 study) OR TI(retrospective) OR AB(retrospective))	72
S26	TI(Intervention* NEAR/1 Stud*) OR AB(Intervention* NEAR/1 Stud*)	30
S27	TI(assign* NEAR/3 (subject* OR patient*))	0
S28	TI(quasi-experiment* OR quasiexperiment* OR experiment*) OR AB(quasiexperiment* OR quasiexperiment* OR experiment*)	1052
S29	AB(assign* NEAR/3 (subject* OR patient*))	8
S30	TI(Propensity score* OR match* NEAR/1 (control*)) OR (match* NEAR/1 (compar*)) OR (assessment only OR comparison samp* OR propensity match*) OR AB(Propensity score* OR (match* NEAR/1 control*)) OR (match* NEAR/1 compar*) OR (assessment only OR comparison samp* OR propensity match*)	7368
S31	TI(Non-random* OR nonradom* OR (non NEAR/1 random*)) OR AB(Nonrandom* OR Nonrandom* OR (non NEAR/1 random*))	8
S32	TI(random* NEAR/2 (trial* OR RCT)) OR AB(random* NEAR/2 (trial* OR RCT))	9
S33	TI(quasi-experiment* OR quasiexperiment* OR Propensity score* OR (compar* NEAR/1 (group*))) OR (match* NEAR/1 (control*)) OR (match* NEAR/1 (group*)) OR (match* NEAR/1 (compar*)) OR (experiment* trial* OR experiment* design* OR experiment* method* OR experiment* stud* OR experiment* evaluation* OR experiment* test* OR experiment* assessment* OR assessment only OR (comparison NEAR/1 (samp*))) OR propensity match* OR (Between NEAR/1 (group*)) OR AB(quasi-experiment* OR quasiexperiment* OR Propensity score* OR (compar* NEAR/1 (group*))) OR (match* NEAR/1 (control*)) OR (match* NEAR/1 (group*)) OR (match* NEAR/1 (compar*)) OR (experiment* trial* OR experiment* design* OR experiment* method* OR experiment* stud* OR experiment* evaluation* OR experiment* test* OR experiment*assessment* OR assessment only OR (comparison NEAR/1 (samp*))) OR (propensity match* OR (Between NEAR/1 (group*)))	10320
S34	AB(assign* NEAR/5 (case*)) OR AB(assign* NEAR/5 (subject*)) OR AB(assign* NEAR/5 (group*)) OR AB(assign* NEAR/5 (patient*)) OR AB(assign* NEAR/5 (intervention*)) OR AB(assign* NEAR/5 (case*)) OR AB(assign* NEAR/5 (subject*)) OR AB(assign* NEAR/5 (group*)) OR AB(assign* NEAR/5 (patient*)) OR AB(assign* NEAR/5 (intervention*))	102
S35	TI(intervention NEAR/5 (case*)) OR TI(intervention NEAR/5 (subject*)) OR TI(intervention NEAR/5 (group*)) OR TI(intervention NEAR/5 (patient*)) OR AB(intervention NEAR/5 (case*)) OR AB(intervention NEAR/5 (subject*)) OR AB(intervention NEAR/5 (group*)) OR AB(intervention NEAR/5 (patient*))	46

S36	TI(experiment* NEAR/5 (case*)) OR TI(experiment* NEAR/5 (subject*)) OR TI(experiment* NEAR/5 (group*)) OR TI(experiment* NEAR/5 (patient*)) OR (experiment* NEAR/5 (intervention)) OR AB(experiment* NEAR/5 (case*)) OR AB(experiment* NEAR/5 (subject*)) OR AB(experiment* NEAR/5 (group*)) OR AB(experiment* NEAR/5 (patient*)) OR AB(experiment* NEAR/5 (intervention*))	314
S37	TI(treatment NEAR/5 (case)) OR TI(treatment NEAR/5 (subject*)) OR TI(treatment NEAR/5 (group*)) OR TI(treatment NEAR/5 (patient*)) OR TI(treatment NEAR/5 (intervention)) OR AB(treatment NEAR/5 (case)) OR AB(treatment NEAR/5 (subject*)) OR AB(treatment NEAR/5 (group*)) OR AB(treatment NEAR/5 (patient*)) OR AB(treatment NEAR/5 (intervention))	87
S38	TI(control NEAR/5 (case)) OR TI(control NEAR/5 (subject*)) OR TI(control NEAR/5 (group*)) OR TI(control NEAR/5 (patient*)) OR TI(control NEAR/5 (intervention)) OR AB(control NEAR/5 (case)) OR AB(control NEAR/5 (subject*)) OR AB(control NEAR/5 (group*)) OR AB(control NEAR/5 (patient*)) OR AB(control NEAR/5 (intervention))	256
S39	(TI(regression NEAR/1 (discontinuity)) OR TI(difference-in-difference*) OR TI(event NEAR/1 stud*) OR TI (interrupted time serie*) OR TI(instrumental variable*) OR TI(waitlist control*)) OR (AB(regression NEAR/1 (discontinuity)) OR AB(difference-in-difference*) OR AB(event NEAR/1 stud*) OR AB(interrupted time serie*) OR AB(instrumental variable*) OR AB(waitlist control*))	27
S40	S18 OR S19 OR S20 OR S21 OR S22 OR S23 OR S24 OR S25 OR S26 OR S27 OR S28 OR S29 OR S30 OR S31 OR S32 OR S33 OR S34 OR S35 OR S36 OR S37 OR S38 OR S39	13382
S41	S17 AND S40	97

Australian Education Index

Searched through ProQuest interface. Searched 01/03/2016. Limiters - Date Published: 01/01/1980 - 2016/01/03.

Search	Search Terms	Results
1	TI:(school* OR primary school* OR elementary school* OR secondary school* OR high school* OR middle school* OR student* OR pupil* OR child* OR lowachiev* OR underachiev* OR disab*) AND TI:(performance* OR academic* OR achieve* OR abilit* OR learn* OR outcome* OR intell* OR read* OR literac* OR math* OR develop* OR numerac* OR langua*) AND TI:(random* OR placebo OR RCT* OR trial* OR group* OR quasi-experiment* OR cohort* OR case* intervent* OR experiment* OR study* OR evaluat* OR treatment* OR longitudinal* OR evidence* OR best* OR effective*)	398
2	SU:(perform* OR academic* OR achieve* OR abilit* OR learn* OR outcome* OR intell* OR read* OR literac* OR math* OR develop* OR numerac* OR langua*)	509
3	1 AND 2	85

Science Citation Index & Social Science Citation Index

Indexes=SCI-EXPANDED, SSCI Timespan=2016-2018. Searched 29/6 – 2018.

# 55	907	#54 AND #25
# 54	1,794,160	#53 OR #52 OR #51 OR #50 OR #49 OR #48 OR #47 OR #46 OR #45 OR #44 OR #43 OR #42 OR #41 OR #40 OR #39 OR #38 OR #37 OR #36 OR #35 OR #34 OR #33 OR #32 OR #31 OR #30 OR #29 OR #28 OR #27 OR #26
# 53	8,096	(TS=(regression NEAR/1 (discontinuity)) OR TS=(difference-in-difference*) OR TS=(event NEAR/1 stud*) OR TS=(interrupted time serie*) OR TS=(instrumental variable*) OR TS=(waitlist control*))
# 52	8,096	((TI=(regression NEAR/1 (discontinuity)) OR TI=(difference-in-difference*) OR TI=(event NEAR/1 stud*) OR TI=(interrupted time serie*) OR TI=(instrumental variable*) OR TI=(waitlist control*)) OR (TS=(regression NEAR/1 (discontinuity)) OR TS=(difference-in-difference*) OR TS=(event NEAR/1 stud*) OR TS=(interrupted time serie*) OR TS=(instrumental variable*) OR TS=(waitlist control*)))
# 51	145,391	(TI=(control NEAR/5 (case)) OR TI=(control NEAR/5 (subject*)) OR TI=(control NEAR/5 (group*)) OR TI=(control NEAR/5 (patient*)) OR TI=(control NEAR/5 (intervention)) OR TS=(control NEAR/5 (case)) OR TS=(control NEAR/5 (subject*)) OR TS=(control NEAR/5 (group*)) OR TS=(control NEAR/5 (patient*)) OR TS=(control NEAR/5 (intervention)))
# 50	113,967	(TI=(treatment NEAR/5 (case)) OR TI=(treatment NEAR/5 (subject*)) OR TI=(treatment NEAR/5 (group*)) OR TI=(treatment NEAR/5 (patient*)) OR TI=(treatment NEAR/5 (intervention*)) OR TS=(treatment NEAR/5 (case)) OR TS=(treatment NEAR/5 (subject*)) OR TS=(treatment NEAR/5 (group*)) OR TS=(treatment NEAR/5 (patient*)) OR TS=(treatment NEAR/5 (intervention*)))
# 49	23,187	(TI=(experiment* NEAR/5 (case*)) OR TI=(experiment* NEAR/5 (subject*)) OR TI=(experiment* NEAR/5 (group*)) OR TI=(experiment* NEAR/5 (patient*)) OR TI=(experiment* NEAR/5 (intervention*)) OR TS=(experiment* NEAR/5 (case*)) OR TS=(experiment* NEAR/5 (subject*)) OR TS=(experiment* NEAR/5 (group*)) OR TS=(experiment* NEAR/5 (patient*)) OR TS=(experiment* NEAR/5 (intervention*)))
# 48	29,075	(TI=(intervention NEAR/5 (case*)) OR TI=(intervention NEAR/5 (subject*)) OR TI=(intervention NEAR/5 (group*)) OR TI=(intervention NEAR/5 (patient*)) OR TS=(intervention NEAR/5 (case*)) OR TS=(intervention NEAR/5 (subject*)) OR TS=(intervention NEAR/5 (group*)) OR TS=(intervention NEAR/5 (patient*)))
# 47	15,431	((TS=(assign* NEAR/5 (case*)) OR TS=(assign* NEAR/5 (subject*)) OR TS=(assign* NEAR/5 (group*)) OR TS=(assign* NEAR/5 (patient*)) OR TS=(assign* NEAR/5 (intervention*)) OR TS=(assign* NEAR/5 (case*)) OR TS=(assign* NEAR/5 (subject*)) OR TS=(assign* NEAR/5 (group*)) OR TS=(assign* NEAR/5 (patient*)) OR TS=(assign* NEAR/5 (intervention*))) OR (TI=(assign* NEAR/5 (case*)) OR TI=(assign* NEAR/5 (subject*)) OR TI=(assign* NEAR/5 (group*)) OR TI=(assign* NEAR/5 (patient*)) OR TI=(assign* NEAR/5 (intervention*)) OR TI=(assign* NEAR/5 (case*)) OR TI=(assign* NEAR/5 (subject*)) OR TI=(assign* NEAR/5 (group*)) OR TI=(assign* NEAR/5 (patient*)) OR TI=(assign* NEAR/5 (intervention*))))
# 46	565,907	(TI=((quasi-experiment* OR quasiexperiment* OR Propensity score* OR (compar* NEAR/1 (group*))) OR (match* NEAR/1 (control*)) OR (match* NEAR/1 (group*)) OR (match* NEAR/1 (compar*)) OR (experiment* trial* OR experiment* design* OR experiment* method* OR experiment* stud* OR experiment* evaluation* OR experiment* test* OR experiment* assessment*

		OR assessment only OR (comparison NEAR/1 (samp*))) OR propensity match* OR (Between NEAR/1 (group*))) OR TS=(((quasi-experiment* OR quasiexperiment* OR Propensity score* OR (compar* NEAR/1 (group*))) OR (match* NEAR/1 (control*)) OR (match* NEAR/1 (group*)) OR (match* NEAR/1 (compar*)) OR (experiment* trial* OR experiment* design* OR experiment* method* OR experiment* stud* OR experiment* evaluation* OR experiment* test* OR experiment* assessment* OR assessment only OR (comparison NEAR/1 (samp*))) OR propensity match* OR (Between NEAR/1 (group*)))))
# 45	108,956	(TI=((random* NEAR/2 (trial* OR RCT))) OR TS=((random* NEAR/2 (trial* OR RCT))))
# 44	4,062	(TI=((Non-random* OR nonradom* OR (non NEAR/1 random*))) OR TS=((Non-random* OR nonradom* OR (non NEAR/1 random*))))
# 43	3,854	(TI=(((propensity score* OR match* NEAR/1 (control*)) OR (match* NEAR/1 (compar*))) OR (assessment only OR comparison samp* OR propensity match*)))
# 42	5,003	(TI=(assign* NEAR/3 (subject* OR patient*)) OR TS=(assign* NEAR/3 (subject* OR patient*)))
# 41	576,844	((TI=(quasi-experiment* OR quasiexperiment* OR experiment*) OR TS=(quasi-experiment* OR quasiexperiment* OR experiment*)))
# 40	7,598	(TI=(Intervention* NEAR/1 stud*) OR TS=(Intervention* NEAR/1 stud*))
# 39	85,921	(TI=(retrospective) OR TS=(retrospective))
# 38	47,061	(TI=(prospective NEAR/2 stud*) OR TS=(prospective NEAR/2 stud*))
# 37	41,780	(TI=(observational) OR TS=(observational))
# 36	59,575	(TS=(longitudinal) OR TI=(longitudinal))
# 35	111	(TS=(followup NEAR/2 stud*) OR TI=(followup NEAR/2 stud*))
# 34	9,238	(TI=(epidemiologic* NEAR/2 stud*) OR TS=(epidemiologic* NEAR/2 stud*))
# 33	63,729	(TI=("cross sectional*") OR TS=(cross sectional*))
# 32	82,834	(TI=("case control*") OR TS=(case control*))
# 31	265,582	(TS=(case stud*) OR TI=(case stud*))
# 30	137,308	(TS=(cohort*) OR TI=(cohort*))
# 29	577,738	(TS=(group*) OR TI=(group*))
# 28	253,654	(TS=(trial*) OR TI=(trial*))
# 27	30,786	(TS=(placebo) OR TI=(placebo))
# 26	259,337	(TS=(random*) OR TI=(random*))
# 25	1,604	(#24 AND #23 AND #22)
# 24	153,311	(#21 OR #20 OR #19 OR #18 OR #17)
# 23	115,940	(#16 OR #15 OR #14 OR #13 OR #12 OR #11 OR #10 OR #9)
# 22	26,219	(#8 OR #7 OR #6 OR #5 OR #4 OR #3 OR #2 OR #1)
# 21	439	((TI=((transfer* NEAR/2 (effect)))))
# 20	418	((TS=("math* educat*")))
# 19	57,646	((TS=("math*")))

# 18	8,847	((TS=("literac*")))
# 17	90,606	((TS=("read*")))
# 16	351	((TI=((Intellect* NEAR/2 (develop*)))))
# 15	280	((TI=((school* NEAR/1 (performan* or achiev*)))))
# 14	1,266	((TI=((academic* NEAR/2 (performance* or achiev* or abilit* or outcome*)))))
# 13	709	((TI=((learn* NEAR/2 (disab* or problem*)))))
# 12	1,676	((TS=("learn* disabilit*")))
# 11	112,722	((TS=("learn*")))
# 10	11	((TS=("academic* achieve* gap*")))
# 9	3,093	((TS=("academic* achieve*")))
# 8	23,853	((TI=((Student* OR pupil* NEAR/3 (Learn*) NEAR/2 (disab* OR problem*)))))
# 7	3,385	((TI=(((Primary NEAR/1 (School)) NEAR/3 (Student* OR pupil*)) OR ((Elementary NEAR/1 (School)) NEAR/3 (Student* OR pupil*)) OR ((Secondary NEAR/1 (school*)) OR (high NEAR/2 (school*)) OR (middle NEAR/2 (School*)) NEAR/3 (student* OR pupil*)))))
# 6	780	((TI=((Underachiev* OR Under NEAR/1 (achiev* OR lowachiev*)) OR (low NEAR/2 (achiev*)) OR (Low NEAR/1 (perform* OR lowperform*)) OR ((at-risk OR “at” NEAR/1 (risk)) NEAR/1 (student* OR pupil*)) OR ((high-risk OR high NEAR/1 (risk)) NEAR/1 (student* OR pupil*)) OR ((Special NEAR/1 (Need*)) NEAR/1 (Student* OR pupil*)) OR ((Low NEAR/1 (income)) NEAR/1 (student* OR pupil*)))))
# 5	23,853	((TI=((Student* OR pupil* NEAR/3 (Learn*) NEAR/2 (disab* or problem*)))))
# 4	117	((TI=(("Foster Care*") AND child*)))
# 3	0	(TI=((Child* NEAR/2 (placed) NEAR/1 (care))))
# 2	3,385	((TI=(((Primary NEAR/1 (School)) NEAR/3 (Student* or pupil*)) OR ((Elementary NEAR/1 (School)) NEAR/3 (Student* or pupil*)) OR ((Secondary NEAR/1 (school*)) OR (high NEAR/2 (school*)) OR (middle NEAR/2 (School*)) NEAR/3 (student* OR pupil*)))))
# 1	780	(TI=((Underachiev* OR Under NEAR/1 (achiev* or lowachiev*)) OR (low NEAR/2 (achiev*)) OR (Low NEAR/1 (perform* OR lowperform*)) OR ((at-risk OR "at" NEAR/1 (risk)) NEAR/1 (student* or pupil*)) OR ((high-risk or high NEAR/1 (risk)) NEAR/1 (student* or pupil*)) OR ((Special NEAR/1 (Need*)) NEAR/1 (Student* or pupil*)) OR ((Low NEAR/1 (income)) NEAR/1 (student* or pupil*))))

Medline & Embase

Searched through OVID-host. Searched 04/03/2016. Search limited from 1980-2016.

Search	Query	Results
57	34 and 56	323
56	35 OR 36 OR 37 OR 38 OR 39 OR 40 OR 41 OR 42 OR 43 OR 44 OR 45 OR 46 OR 47 OR 48 OR 49 OR 50 OR 51 OR 52 OR 53 OR 54 OR 55	5063720

Search	Query	Results
55	((((regression adj1 discontinuity) OR difference-in-difference* OR event) adj1 stud*) OR interrupted time serie* OR instrumental variable* OR waitlist control*).ti. OR ((((regression adj1 discontinuity) OR difference-in-difference* OR event) adj1 stud*) OR interrupted time serie* OR instrumental variable* OR waitlist control*).ab.	2881
54	((control adj5 case) OR (control adj5 subject*) OR (control adj5 group*) OR (control adj5 patient*) OR (control adj5 intervention)).ti. OR ((control adj5 case) OR (control adj5 subject*) OR (control adj5 group*) OR (control adj5 patient*) OR (control adj5 intervention)).ab.	550849
53	((treatment adj5 case) OR (treatment adj5 subject*) OR (treatment adj5 group*) OR (treatment adj5 patient*) OR (treatment adj5 intervention)).ti. OR ((treatment adj5 case) OR (treatment adj5 subject*) OR (treatment adj5 group*) OR (treatment adj5 patient*) OR (treatment adj5 intervention)).ab.	523471
52	((experiment* adj5 case) OR (experiment* adj5 subject*) OR (experiment* adj5 group*) OR (experiment* adj5 patient*) OR (experiment* adj5 intervention)).ti. OR ((experiment* adj5 case) OR (experiment* adj5 subject*) OR (experiment* adj5 group*) OR (experiment* adj5 patient*) OR (experiment* adj5 intervention)).ab.	79366
51	((intervention adj5 case) OR (intervention adj5 subject*) OR (intervention adj5 group*) OR (intervention adj5 patient*)).ti. OR ((intervention adj5 case) OR (intervention adj5 subject*) OR (intervention adj5 group*) OR (intervention adj5 patient*)).ab.	65864
50	((assign* adj5 case) OR (assign* adj5 subject*) OR (assign* adj5 group*) OR (assign* adj5 patient*) OR (assign* adj5 intervention)).ti. OR ((assign* adj5 case) OR (assign* adj5 subject*) OR (assign* adj5 group*) OR (assign* adj5 patient*) OR (assign* adj5 intervention)).ab.	62637
49	(quasi-experiment* OR quasiexperiment* OR Propensity score* OR (compar* adj1 group*) OR (match* adj1 control*) OR (match* adj1 group*) OR (match* adj1 compar*) OR experiment* trial* OR experiment* design* OR experiment* method* OR experiment* stud* OR experiment* evaluation* OR experiment* test* OR experiment* assessment* OR assessment only OR (comparison adj1 samp*) OR propensity match* OR (Between adj1 group*)).ti. OR (quasi-experiment* OR quasiexperiment* OR Propensity score* OR (compar* adj1 group*) OR (match* adj1 control*) OR (match* adj1 group*) OR (match* adj1 compar*) OR experiment* trial* OR experiment* design* OR experiment* method* OR experiment* stud* OR experiment* evaluation* OR experiment* test* OR experiment* assessment* OR assessment only OR (comparison adj1 samp*) OR propensity match* OR (Between adj1 group*)).ab.	338123
48	((random* adj2 trial*) OR RCT).ti. OR ((random* adj2 trial*) OR RCT).ab.	176530
47	(Non-random* OR nonradom* OR (non adj1 random*)).ti. OR (Non-random* OR nonradom* OR (non adj1 random*)).ab.	11689
46	(Propensity score* OR (match* adj1 control*) OR (match* adj1 compar*) OR assessment only OR comparison samp* OR propensity match*).ti. OR (Propensity score* OR (match* adj1 control*) OR (match* adj1 compar*) OR assessment only OR comparison samp* OR propensity match*).ab.	74039
45	(assign adj3 (subject* OR patient*)).ti. OR (assign adj3 (subject* OR patient*)).ab.	447
44	(quasi-experiment* OR quasiexperiment* OR experiment*).ti. OR (quasi-experiment* OR quasiexperiment* OR experiment*).ab.	1357128
43	(intervention* adj1 stud*).ti. OR (intervention* adj1 stud*).ab.	16512
42	retrospective.ti. OR retrospective.ab.	288643
41	(prospective adj2 stud*).ti. OR (prospective adj2 stud*).ab.	193506
40	longitudinal.ti. OR longitudinal.ab. OR observational.ti. OR observational.ab.	218571

Search	Query	Results
39	(((epidemiologic adj2 study).ti. OR epidemiologic.mp.) adj2 study.ab.) OR ((followup OR follow up) adj2 stud*).ti. OR ((followup OR follow up) adj2 stud*).ab. [mp=title, abstract, original title, name of substance word, subject heading word, keyword heading word, protocol supplementary concept word, rare disease supplementary concept word, unique identifier]	45099
38	cross sectional.ti. OR cross sectional.ab.	175279
37	(case control OR cohort).ti. OR case control.ab. OR cohort.ab.	334639
36	(cohort analysis OR case studies).hw,kf,ui.	13018
35	(randomized OR placebo OR (randomly OR trial*) OR group*).ab.	2929343
34	16 and 27 and 33	787
33	28 OR 29 OR 30 OR 31 OR 32	107384
32	(transfer adj2 effect*).af.	2559
31	((numerac* OR math*) adj1 learn*).af.	1233
30	Literacy.hw,kf,ui.	4390
29	Reading.hw,kf,ui.	43205
28	Language.hw,kf,ui.	60377
27	17 OR 18 OR 19 OR 20 OR 21 OR 22 OR 23 OR 24 OR 25 OR 26	328504
26	(intellect adj2 develop*).af.	13
25	(school* adj1 (performan* OR achiev*)).af.	4038
24	(academic adj2 (performanc* OR achiev* OR abilit* OR outcome*)).af.	6288
23	(learn* adj2 (disab* OR problem*)).af.	18651
22	Intelligence.hw,kf,ui.	52794
21	Learning.hw,kf,ui.	141813
20	Learning Disorders.hw,kf,ui.	13014
19	Students.hw,kf,ui.	91367
18	Child Development.hw,kf,ui.	43742
17	achievement.hw,kf,ui.	13876
16	9 OR 10 OR 11 OR 12 OR 13 OR 14 OR 15	100681
15	((student* OR pupil*) adj3 (learn adj2 (disab* OR problem*))).af.	9
14	foster home care.hw. and child*.af.	2738
13	(((secondary adj 1 school*) OR (high adj2 school) OR (middle adj1 school)) adj3 (student* OR pupil*)).af.	7980
12	school*.hw.	86001
11	(elementary adj1 school* adj3 (student* OR pupil*)).af.	835
10	(primary adj1 school* adj3 (student* OR pupil*)).af.	697
9	1 OR 2 OR 3 OR 4 OR 5 OR 6 OR 7 OR 8	5336
8	(low adj1 income adj1 (student* OR pupil*)).af.	59
7	(special adj1 need* adj1 (student* OR pupil*)).af.	25
6	((high-risk OR high) adj1 risk adj1 (student* OR pupil*)).af.	105

Search	Query	Results
5	((at-risk OR at) adj1 risk adj1 (student* OR pupil*)).af.	424
4	(low adj1 perform*).af.	1507
3	(low adj1 achiev*).af.	948
2	(under adj1 achiev*).af.	1500
1	(underachiev* OR lowachieve* OR lowperform).af.	845

SEARCHES ON OTHER RESOURCES

DIVA

https://www.diva-portal.org/smash/search.jsf?dswid=-7369

Searches were performed in the "Advanced Search – Research publications" fields. We used a combination of terms in the three search facets below:

1. school*, primary school*, elementary school*, secondary school*, high school*, middle school*, student*, pupil*, child*, lowachiev*, underachiev*
2. performance*, academic*, achieve*, abilit*, learn*, outcome*, intell*, read*, literac*, math*, develop*, numerac*
3. random*, placebo, RCT*, trial*, group*, quasi-experiment*, cohort*, case* intervent*, experiment*, study*, evaluat*, treatment*, longitudinal*

The original searches were performed in March 2016 and yielded 597 results. The searches were limited to 1980-2016. The updated searches in September 2018 yielded 102 new results.

CRISTIN

https://app.cristin.no/

Searches were performed in the search field on the main page. Our searches combined terms from the two facets below:

1. school*, primary school*, elementary school*, secondary school*, high school*, middle school*, student*, pupil*, child*, lowachiev*, underachiev*
2. performance*, academic*, achieve*, abilit*, learn*, outcome*, intell*, read*, literac*, math*, develop*, numerac*
3. random*, placebo, RCT*, trial*, group*, quasi-experiment*, cohort*, case* intervent*, experiment*, study*, evaluat*, treatment*, longitudinal*

The original searches were performed in March 2016 and yielded 180 results. The searches were limited to 1980-2016. The resource was not searched in the 2018 update to the maintenance and update of the search interface during the search period.

Since the searches were performed, the search interface of CRISTIN has been changed. The searches we performed might be hard to replicate in the current search interface.

Forskningsdatabasen

https://www.forskningsdatabasen.dk/

Searches were performed in the "Publications" search field. We used a combination of terms in the three search facets below:

1. school*, primary school*, elementary school*, secondary school*, high school*, middle school*, student*, pupil*, child*, lowachiev*, underachiev*
2. performance*, academic*, achieve*, abilit*, learn*, outcome*, intell*, read*, literac*, math*, develop*, numerac*
3. random*, placebo, RCT*, trial*, group*, quasi-experiment*, cohort*, case* intervent*, experiment*, study*, evaluat*, treatment*, longitudinal*

The original searches were performed in March 2016 and yielded 109 results. The searches were limited to 1980-2016. The updated search in September 2018 yielded 6 new results.

Cochrane Library
https://www.cochranelibrary.com/

Searches were performed in the "Record Title" search field. We used a combination of terms in the three search facets below:

1. school*, primary school*, elementary school*, secondary school*, high school*, middle school*, student*, pupil*, child*, lowachiev*, underachiev*
2. performance*, academic*, achieve*, abilit*, learn*, outcome*, intell*, read*, literac*, math*, develop*, numerac*
3. random*, placebo, RCT*, trial*, group*, quasi-experiment*, cohort*, case* intervent*, experiment*, study*, evaluat*, treatment*, longitudinal*

The original searches were performed in March 2016 and yielded 37 results. The searches were limited to 1980-2016. The updated searches in September 2018 yielded no new results.

Social Care Online
https://www.scie-socialcareonline.org.uk/User/login?ReturnUrl=%2fsearch%2fexpert

Searches were performed in the "title" search field. We used a combination of terms in the three search facets below:

1. school*, primary school*, elementary school*, secondary school*, high school*, middle school*, student*, pupil*, child*, lowachiev*, underachiev*
2. performance*, academic*, achieve*, abilit*, learn*, outcome*, intell*, read*, literac*, math*, develop*, numerac*
3. random*, placebo, RCT*, trial*, group*, quasi-experiment*, cohort*, case* intervent*, experiment*, study*, evaluat*, treatment*, longitudinal*

The original searches were performed in March 2016 and yielded 204 results. The searches were limited to 1980-2016. The updated searches in September 2018 yielded 11 new results.

Centre for Reviews and Dissemination (CRD)
https://www.crd.york.ac.uk/CRDWeb/

Search performed in "title" search field. We used a combination of terms in the three search facets below:

1. school*, primary school*, elementary school*, secondary school*, high school*, middle school*, student*, pupil*, child*, lowachiev*, underachiev*
2. performance*, academic*, achieve*, abilit*, learn*, outcome*, intell*, read*, literac*, math*, develop*, numerac*
3. random*, placebo, RCT*, trial*, group*, quasi-experiment*, cohort*, case* intervent*, experiment*, study*, evaluat*, treatment*, longitudinal*

The original searches were performed in March 2016 and yielded 62 results. The searches were limited to 1980-2016. The updated searches in September 2018 yielded 4 new results.

What Works Clearinghouse - U.S. Department of Education (WWC)
https://ies.ed.gov/ncee/wwc/

The indexing structure of the publications on WWC is divided in topics that functions as filters. Each topic is divided into subsections. Individual studies are indexed as themes each subsections. We selected a range of topics relevant for this review, and screened the individual references indexed under each subsection. Below is an overview of the topics and subsections selected:

Topic: *Children and Youth with disabilities*
Subsection: *Peer-Assisted Learning Strategies, Read Naturally, Coping Power, Lindamood Phoneme Sequencing, First Steps to Success, Early Risers, Repeated Reading, Fast Track Elementary School, Reading Mastery, Project Read Phonology*

Topic: *English learners*
Subsection: *Peer Tutoring and Response Groups, Instructional Conversations and Literature Logs, Fast ForWord, Enhanced Proactive Reading, Bilingual Cooperative Integrated Reading and Composition, Vocabulary Improvement Program for English Language Learners and Their Classmates, Arthur, Read Well, Read Naturally*

Topic: *Literacy*
Subsection: *Sound Partners, Read 180, DaisyQuest, Accelerated Reader, SpellRead, Earobics, Cooperative Integrated Reading and Composition, Reading Recovery, Lexia Reading, Open Court Reading, Stepping Stones to Literacy, Student team reading and writing, Voyager Universal Literacy System, Corrective Reading, Start Making a Reader Today, Early Intervention in Reading, Reading Plus, Reading Apprenticeship, ClassWide Peer Tutoring, Little Books, Read, Write & Type, Failure Free Reading, Waterford Early Reading Program, Wilson Reading System, Reciprocal Teaching, SuccessMaker, LANGUAGE!*

Topic: *Mathematics*

Subsection: *Accelerated Math, I CAN Learn® Pre-Algebra and Algebra, DreamBox Learning, I CAN Learn*

Topic: *Behavior*
Subsection: *Positive Action, Lessons in Character*

Topic: *Path to Graduation*
Subsection: *Accelerated Middle Schools, High School Redirection, Check & Connect, Financial Incentives for Teen Parents to Stay in School, Achievement for Latinos through Academic Success (ALAS), Twelve Together, Quantum Opportunity Program, Project GRAD*

The original searches were performed in January 2017 and yielded 150 references. The updated searches in September 2018 identified no new references.

Danish Clearinghouse for Education Research

https://dpu.au.dk/en/research/publications/

Search performed in "Publications" section search field. We used a combination of terms in the three search facets below:

1. school*, primary school*, elementary school*, secondary school*, high school*, middle school*, student*, pupil*, child*, lowachiev*, underachiev*
2. performance*, academic*, achieve*, abilit*, learn*, outcome*, intell*, read*, literac*, math*, develop*, numerac*
3. random*, placebo, RCT*, trial*, group*, quasi-experiment*, cohort*, case* intervent*, experiment*, study*, evaluat*, treatment*, longitudinal*

Terms were combined with the Danish term OG (=AND).

The original searches were performed in March 2016 and yielded 66 results. The searches were limited to 1982-2016 (no earlier limiter was possible). The updated searches in September 2018 yielded no new results.

The Institute for Education Sciences' (IES) & American Economic Association's RCT Registry

Our protocol stated that we should search two trial registries: The Institute for Education Sciences' (IES) Registry of Randomized Controlled Trials (http://ies.ed.gov/ncee/wwc/references/registries/index.aspx), and American Economic Association's RCT Registry (https://www.socialscienceregistry.org). We were however unable to search the IES registry as it was not available (last tried July 23, 2018). We have asked IES about availability, but have to date not received a reply. We updated the search of American Economic Association's RCT Registry on July 23, 2018.

GREY LITERATURE SEARCHES

We performed a wide range of searches on the below institutional and governmental resources, academic clearinghouses and repositories for relevant academic theses, reports and conference/working papers. Most of the resources searched for grey literature include multiple types of references. The resources are listed under the category of literature most prevalent in the resource, even though multiple types of unpublished/published literature might be identified in the resource.

Dissertations and Theses A&I

Searched through the ProQuest interface. Searched 02/03/2016. Search was limited from 1980-2016.

Search	Search	Results
Set#: S42	S18 AND S41	786
Set#: S41	S19 OR S20 OR S21 OR S22 OR S23 OR S24 OR S25 OR S26 OR S27 OR S28 OR S29 OR S30 OR S31 OR S32 OR S33 OR S34 OR S35 OR S36 OR S37 OR S38 OR S39 OR S40	2,082,703
Set#: S40	(TI(regression NEAR/1 (discontinuity)) OR TI(difference-in-difference*) OR TI(event NEAR/1 stud*) OR TI(interrupted time serie*) OR TI(instrumental variable*) OR TI(waitlist control*)) OR (AB(regression NEAR/1 (discontinuity)) OR AB(difference-in-difference*) OR AB(event NEAR/1 stud*) OR AB(interrupted time serie*) OR AB(instrumental variable*) OR AB(waitlist control*))	7,942
Set#: S39	TI(control NEAR/5 (case)) OR TI(control NEAR/5 (subject*)) OR TI(control NEAR/5 (group*)) OR TI(control NEAR/5 (patient*)) OR TI(control NEAR/5 (intervention)) OR AB(control NEAR/5 (case)) OR AB(control NEAR/5 (subject*)) OR AB(control NEAR/5 (group*)) OR AB(control NEAR/5 (patient*)) OR AB(control NEAR/5 (intervention))	64,842
Set#: S38	TI(treatment NEAR/5 (case)) OR TI(treatment NEAR/5 (subject*)) OR TI(treatment NEAR/5 (group*)) OR TI(treatment NEAR/5 (patient*)) OR TI(treatment NEAR/5 (intervention)) OR AB(treatment NEAR/5 (case)) OR AB(treatment NEAR/5 (subject*)) OR AB(treatment NEAR/5 (group*)) OR AB(treatment NEAR/5 (patient*)) OR AB(treatment NEAR/5 (intervention))	41,096
Set#: S37	TI(experiment* NEAR/5 (case*)) OR TI(experiment* NEAR/5 (subject*)) OR TI(experiment* NEAR/5 (group*)) OR TI(experiment* NEAR/5 (patient*)) OR (experiment* NEAR/5 (intervention)) OR AB(experiment* NEAR/5 (case*)) OR AB(experiment* NEAR/5 (subject*)) OR AB(experiment* NEAR/5 (group*)) OR AB(experiment* NEAR/5 (patient*)) OR AB(experiment* NEAR/5 (intervention*))	56901
Set#: S36	TI(intervention NEAR/5 (case*)) OR TI(intervention NEAR/5 (subject*)) OR TI(intervention NEAR/5 (group*)) OR TI(intervention NEAR/5 (patient*)) OR AB(intervention NEAR/5 (case*)) OR AB(intervention NEAR/5 (subject*)) OR AB(intervention NEAR/5 (group*)) OR AB(intervention NEAR/5 (patient*))	12,588
Set#: S35	AB(assign* NEAR/5 (case*)) OR AB(assign* NEAR/5 (subject*)) OR AB(assign* NEAR/5 (group*)) OR AB(assign* NEAR/5 (patient*)) OR AB(assign* NEAR/5 (intervention*)) OR AB(assign* NEAR/5 (case*)) OR AB(assign* NEAR/5 (subject*)) OR AB(assign* NEAR/5 (group*)) OR AB(assign* NEAR/5 (patient*)) OR AB(assign* NEAR/5 (intervention*))	18,102

Set#: S34	TI(quasi-experiment* OR quasiexperiment* OR Propensity score* OR (compar* NEAR/1 (group*))) OR (match* NEAR/1 (control*)) OR (match* NEAR/1 (group*)) OR (match* NEAR/1 (compar*)) OR (experiment* trial* OR experiment* design* OR experiment* method* OR experiment* stud* OR experiment* evaluation* OR experiment* test* OR experiment* assessment* OR assessment only OR (comparison NEAR/1 (samp*))) OR propensity match* OR (Between NEAR/1 (group*)) OR AB(quasi-experiment* OR quasiexperiment* OR Propensity score* OR (compar* NEAR/1 (group*))) OR (match* NEAR/1 (control*)) OR (match* NEAR/1 (group*)) OR (match* NEAR/1 (compar*)) OR (experiment* trial* OR experiment* design* OR experiment* method* OR experiment* stud* OR experiment* evaluation* OR experiment* test* OR experiment*assessment* OR assessment only OR (comparison NEAR/1 (samp*))) OR (propensity match* OR (Between NEAR/1 (group*)))	1,767,065
Set#: S33	TI(random* NEAR/2 (trial* OR RCT)) OR AB(random* NEAR/2 (trial* OR RCT))	4,915
Set#: S32	TI(Non-random* OR nonradom* OR (non NEAR/1 random*)) OR AB(Non-random* OR Nonrandom* OR (non NEAR/1 random*))	4,150
Set#: S31	TI(Propensity score* OR match* NEAR/1 (control*)) OR (match* NEAR/1 (compar*)) OR (assessment only OR comparison samp* OR propensity match*) OR AB(Propensity score* OR (match* NEAR/1 control*)) OR (match* NEAR/1 compar*) OR (assessment only OR comparison samp* OR propensity match*)	1,437,399
Set#: S30	AB(assign* NEAR/3 (subject* OR patient*))	5,488
Set#: S29	TI(assign* NEAR/3 (subject* OR patient*))	22
Set#: S28	TI(quasi-experiment* OR quasiexperiment* OR experiment*) OR AB(quasi-experiment* OR quasiexperiment* OR experiment*)	473,696
Set#: S27	TI(Intervention* NEAR/1 Stud*) OR AB(Intervention* NEAR/1 Stud*)	4,500
Set#: S26	(TI(prospective NEAR/2 study) OR AB(prospective NEAR/2 study) OR TI(retrospective) OR AB(retrospective))	15,836
Set#: S25	TI(longitudinal) OR AB(longitudinal) OR TI(observational) OR AB(observational)	48,855
Set#: S24	TI(followup NEAR/2 study) OR AB(followup NEAR/2 study)	97
Set#: S23	TI(epidemiologic NEAR/2 study) OR AB(epidemiologic NEAR/2 study)	1,434
Set#: S22	TI(cross sectional) OR AB(cross sectional)	22,979
Set#: S21	TI(case control) OR AB(case control) OR TI(cohort) OR AB(cohort)	64,539
Set#: S20	SU("Cohort") OR SU("case studies")	2,078
Set#: S19	(AB(randomized) OR AB(placebo) OR AB(randomly) OR AB(trial) OR AB(groups))	518,004
Set#: S18	S8 AND S13 AND	842
Set#: S17	S14 OR S15 OR S16	116,044
Set#: S16	TI((transfer* NEAR/2 (effect)))	452
Set#: S15	SU("Mathematics") OR SU("Mathematics education")	94,310
Set#: S14	SU("Reading") OR SU("literacy")	21,826
Set#: S13	S9 OR S10 OR S11 OR S12	16,182
Set#: S12	TI((Intellect* NEAR/2 (develop*)))	378
Set#: S11	TI((School NEAR/1 (performan* OR achiev*)))	1,702
Set#: S10	TI((Academic* NEAR/2 (performance* OR achiev* OR abilit* OR outcome*)))	8,298
Set#: S9	TI((Learn* NEAR/2 (disab* OR Problem*)))	6,112
Set#: S8	S6 OR S7	164,850
Set#: S7	(SU("Academic Achievement") OR SU("academic achievement gaps") OR SU("Learning") OR SU("Learning Disabilities"))	34,380
Set#: S6	TI((Underachiev* OR Under NEAR/1 (achiev* OR lowachiev*)) OR (low NEAR/2 (achiev*)) OR (Low NEAR/1 (perform* OR lowperform*)) OR ((at-risk OR at NEAR/1 (risk)) NEAR/1 (student* OR pupil*)) OR ((high-risk OR high NEAR/1 (risk)) NEAR/1	139,796

	(student* OR pupil*)) OR ((Special NEAR/1 (Need*)) NEAR/1 (Student* OR pupil*)) OR ((Low NEAR/1 (income)) NEAR/1 (student* OR pupil*))) OR ti(((Primary NEAR/1 (School)) NEAR/3 (Student* OR pupil*)) OR ((Elementary NEAR/1 (School)) NEAR/3 (Student* OR pupil*)) OR ((Secondary NEAR/1 (school*)) OR (high NEAR/2 (school*)) OR (middle NEAR/2 (School*)) NEAR/3 (student* OR pupil*))) OR ti((Child* NEAR/2 (placed) NEAR/1 (care))) OR ((SU("Foster Care")) AND child*) OR ti((Student* OR pupil* NEAR/3 (Learn*) NEAR/2 (disab* OR problem*)))	
Set#: S5	TI((Student* OR pupil* NEAR/3 (Learn*) NEAR/2 (disab* OR problem*)))	107,191
Set#: S4	((SU("Foster Care")) AND child*)	678
Set#: S3	TI((Child* NEAR/2 (placed) NEAR/1 (care)))	2
Set#: S2	TI(((Primary NEAR/1 (School)) NEAR/3 (Student* OR pupil*)) OR ((Elementary NEAR/1 (School)) NEAR/3 (Student* OR pupil*)) OR ((Secondary NEAR/1 (school*)) OR (high NEAR/2 (school*)) OR (middle NEAR/2 (School*)) NEAR/3 (student* OR pupil*)))	45,355
Set#: S1	TI((Underachiev* OR Under NEAR/1 (achiev* OR lowachiev*)) OR (low NEAR/2 (achiev*)) OR (Low NEAR/1 (perform* OR lowperform*)) OR ((at-risk OR at NEAR/1 (risk)) NEAR/1 (student* OR pupil*)) OR ((high-risk OR high NEAR/1 (risk)) NEAR/1 (student* OR pupil*)) OR ((Special NEAR/1 (Need*)) NEAR/1 (Student* OR pupil*)) OR ((Low NEAR/1 (income)) NEAR/1 (student* OR pupil*)))	3,766

Theses Canada
https://www.bac-lac.gc.ca/eng/services/theses/Pages/search.aspx

Searches were performed in “Title keyword” or “Abstract keyword” search fields. We used a combination of terms in the two search facets below:

1. school*, primary school*, elementary school*, secondary school*, high school*, middle school*, student*, pupil*, child*, lowachiev*, underachiev*
2. performance*, academic*, achieve*, abilit*, learn*, outcome*, intell*, read*, literac*, math*, develop*, numerac*

Original searches were performed January 2017 and yielded 145 results. The searches were limited to 1980-2017. The updated searches in September 2018 yielded 3 new results.

European Educational Research Association
https://eera-ecer.de/ecer-programmes/

Searches were performed in the ECER programme search interface in the “title” search field. Searches were limited to one conference at a time (limited by a search field), dating back to 1998. We used a combination of terms in the two search facets below:

1. school*, primary school*, elementary school*, secondary school*, high school*, middle school*, student*, pupil*, child*, lowachiev*, underachiev*
2. performance*, academic*, achieve*, abilit*, learn*, outcome*, intell*, read*, literac*, math*, develop*, numerac*

The original searches were performed in January 2017 and yielded 73 results. The original searches covered the ECER programmes from 1998-2016. The updated searches in September 2018 yielded 17 new results.

American Educational Research Association
https://www.aera.net/Publications/Online-Paper-Repository/AERA-Online-Paper-Repository

Searches were performed in the AERA Online Paper Repository. We used a combination of terms in the two search facets below:

1. school*, primary school*, elementary school*, secondary school*, high school*, middle school*, student*, pupil*, child*, lowachiev*, underachiev*
2. performance*, academic*, achieve*, abilit*, learn*, outcome*, intell*, read*, literac*, math*, develop*, numerac*

Original searches were performed in January 2017 and yielded 162 results. The original searches covered annual meeting papers dating from 2010. Searches were limited to one year at a time. The updated searches in September 2018 yielded no new results.

German Educational Research Association
https://www.dgfe.de/zeitschrift-erziehungswissenschaft
We hand-searched volumes (heft) 48-53 of the journal "Erziehungwissenschaft" (covering from 2014-2017).

Original hand-search were performed in January 2017 and yielded 47 results. The updated hand-search in September 2018 yielded new results.

NBER working paper series
https://www.nber.org/papers?page=1&perPage=50&sortBy=public_date

Searches were performed in the "Title, Number or Keyword" field. Searches were limited with the "Topics" filter "Health, Education, and Welfare". Original searches were limited from 1/1/1980-01/01/2017. We used a combination of terms in the two search facets below:

1. school*, primary school*, elementary school*, secondary school*, high school*, middle school*, student*, pupil*, child*, lowachiev*, underachiev*
2. performance*, academic*, achieve*, abilit*, learn*, outcome*, intell*, read*, literac*, math*, develop*, numerac*

Original searches were performed in January 2017 and yielded 492 results. The updated searches in September 2018 yielded 5 new results.

OpenGrey

http://www.opengrey.eu/search/

Searches were performed in the main search field. We used a combination of terms in the two search facets below:

1. school*, primary school*, elementary school*, secondary school*, high school*, middle school*, student*, pupil*, child*, lowachiev*, underachiev*
2. performance*, academic*, achieve*, abilit*, learn*, outcome*, intell*, read*, literac*, math*, develop*, numerac*

Original searches were performed in January 2017 and yielded 1427 results. Searches were not limited by year, but we only screened references from 1980-. The updated searches in September 2018 yielded no new results.

Best Evidence Encyclopedia
http://www.bestevidence.org/

Best Evidence Encyclopedia is divided into subsections of "Program Reviews". In each subsection there is links to abstracts, references and full reports. We screened all the subsections for relevant references. Furthermore, we selected relevant reviews to be citation tracked. The reviews selected in this manner is listed in the "Reviews used for citation tracking" section.

Original screening and citation tracking were performed in January 2017 and yielded 112 results. The updated searches in September 2018 yielded no new results.

Google Scholar
https://scholar.google.dk/

We used the advanced search interface for the Google Scholar searches. We used the following search details inserted in the search template below.

Google Scholar Search Template	Insert terms/detalis below
All of the words	school intervention
Any of the words	underachiev lowachiev lowperform "special need"
Year of publication between	1980-2016
Title words only	X
Results/Retrieved	179
Search Date	21/11-2016

The updated search in September 2018 yielded 30 new results.

Google

We searched Google by using a combination of terms in the two search facets below:

1. school*, primary school*, elementary school*, secondary school*, high school*, middle school*, student*, pupil*, child*, lowachiev*, underachiev*
2. performance*, academic*, achieve*, abilit*, learn*, outcome*, intell*, read*, literac*, math*, develop*, numerac*
3. random*, placebo, RCT*, trial*, group*, quasi-experiment*, cohort*, case* intervent*, experiment*, study*, evaluat*, treatment*, longitudinal*

The original searches were performed in January 2017, and yielded 150 results. We did not search Google in the 2018 update.

Reviews used for citation tracking

The reference lists of the following reviews were screened for relevant records. Relevant records were obtained and screened in full text.

Alfieri, L., Brooks, P. J., Aldrich, N. J., & Tenenbaum, H. R. (2011). Does discovery-based instruction enhance learning?. *Journal of Educational Psychology*, 103(1), 1-18.

Cheung, A. C., & Slavin, R. E. (2012). How features of educational technology applications affect student reading outcomes: A meta-analysis. *Educational Research Review*, 7(3), 198-215.

de Boer, H., Donker, A. S., & van der Werf, M. P. (2014). Effects of the attributes of educational interventions on students' academic performance: A meta-analysis. *Review of Educational Research*, 84(4), 509-545.

Dexter, D. D., & Hughes, C. A. (2011). Graphic organizers and students with learning disabilities: A meta-analysis. *Learning Disability Quarterly*, 34(1), 51-72.

Dietrichson, J., Bøg, M., Filges, T., & Klint Jørgensen, A-M. (2017). Academic interventions for elementary and middle school students with low socioeconomic status: A systematic review and meta-analysis. *Review of Educational Research*, 87(2), 243-282.

Edmonds, M. S., Vaughn, S., Wexler, J., Reutebuch, C., Cable, A., Klingler Tackett, K., & Wick Schnakenberg, J. (2009). A synthesis of reading interventions and effects on reading comprehension outcomes for older struggling readers. *Review of Educational Research*, 79(1), 262-300.

Elbaum, B., Vaughn, S., Tejero Hughes, M., & Watson Moody, S. (2000). How effective are one-to-one tutoring programs in reading for elementary students at risk for reading failure? A meta-analysis of the intervention research. *Journal of Educational Psychology*, 92(4), 605-619.

Flynn, L. J., Zheng, X., & Swanson, H. L., (2012). Instructing struggling older readers: A selective meta-analysis of intervention research. *Learning Disabilities Research & Practice*, 27(1), 21-32.

Forsman, H., & Vinnerljung, B. (2012). Interventions aiming to improve school achievements of children in out-of-home care: A scoping review. *Children and Youth Services Review*, 34(6), 1084-1091.

Gersten, R., Chard, D. J., Jayanti, M., Baker, S. K., Morphy, P., & Flojo, P. (2009). Mathematics instruction for students with learning disabilities: A meta-analysis of instructional components. *Review of Educational Research*, 79(3), 1202-1242.

Goodwin, A. P., & Ahn, S. (2010). A meta-analysis of morphological interventions: Effects on literacy achievement of children with literacy difficulties. *Annals of dyslexia*, 60(2), 183-208.

Kyndt, E., Raes, E., Lismont, B., Timmers, F., Cascallar, E., & Dochy, F. (2013). A meta-analysis of the effects of face-to-face cooperative learning. Do recent studies falsify or verify earlier findings?. *Educational Research Review*, 10, 133-149.

Reljić, G., Ferring, D., & Martin, R. (2015). A Meta-Analysis on the effectiveness of bilingual programs in Europe. *Review of Educational Research*, 85(1), 92-128.

Ritter, G., Albin, G., Barnett, J., Blankenship, V., & Denny, G. (2006). The effectiveness of volunteer tutoring programs: A systematic review. *Campbell Systematic Reviews*, 7. DOI: 10.4073/csr.2006.7. Retrieved from http://campbellcollaboration.org/lib/project/16/.

Robinson, D. R., Schofield, J. W., & Steers-Wentzell, K. L. (2005). Peer and cross-age tutoring in math: outcomes and their design implications. *Educational Psychology Review*, 17(4), 327-362.
Scammaca, N. K., Roberts, G., Vaughn, S., & Stuebing, K. K. (2015). A meta-analysis of interventions for struggling readers in grades 4-12: 1980-2011. *Journal of Learning Disabilities*, 48(4), 369-390.
Slavin, R. E., Cheung, A., Groff, C., & Lake, C. (2008). Effective reading programs for middle and high schools: A best-evidence synthesis. *Reading Research Quarterly*, 43(3), 290-322.
Slavin, R. E., & Lake, C. (2008). Effective programs in elementary mathematics: A best-evidence synthesis. *Review of Educational Research*, 78(3), 427-515.
Slavin, R. E., Lake, C., & Groff, C. (2009). Effective programs in middle and high school mathematics: A best-evidence synthesis. *Review of Educational Research*, 79(2), 839-911.
Slavin, R. E., Lake, C., Chambers, B., Cheung, A., & Davis, S. (2009). Effective reading programs for the elementary grades: A best-evidence synthesis. *Review of Educational Research*, 79(4), 1391-1466.
Slavin, R. E., Lake, C., Davis, S., & Madden, N. A. (2011). Effective programs for struggling readers: A best-evidence synthesis. *Educational Research Review*, 6(1), 1-26.
Wilson, S., Tanner-Smith, E. E., Lipsey, M. W., Steinka-Fry, K., & Morrison, J. (2011). Dropout prevention and intervention programs: Effects on school completion and dropout among school-aged children and youth. *Campbell Systematic Reviews*, 7 Retrieved from http://www.campbellcollaboration.org/lib/project/158/.
Wanzek, J., Vaughn, S., Wexler, J., Swanson, E. A., Edmonds, M., & Kim, A-H., 2006. A synthesis of spelling and reading interventions and their effects on the spelling outcomes of students with LD. *Journal of Learning Disabilities*, 39(2), 528-543.
Wanzek, J., Vaughn, S., Wexler, J., Swanson, E. A., Edmonds, M., & Kim, A-H., 2006. A synthesis of spelling and reading interventions and their effects on the spelling outcomes of students with LD. *Journal of Learning Disabilities*, 39(2), 528-543.
