# Supplementary material for: Targeted school‐based interventions for improving reading and mathematics for students with or at risk of academic difficulties in Grades K‐6: A systematic review
Source: Campbell Syst Rev. 2021 Apr 6;17(2):e1152. doi: 10.1002/cl2.1152 (PMC8356298; doi:10.1002/cl2.1152)
Supplement: Supplementary file 2 — Supporting information [file CL2-17-e1152-s010.pdf]

Online Appendix B: Risk of bias tool

Risk of bias table

Item	Judgement[a]	Description (quote from paper, or describe key information)
1. Sequence generation		
2. Allocation concealment		
3. Confounding[b,c]		
4. Blinding?[b]		
5. Incomplete outcome data addressed?[b]		
6. Free of selective reporting?[b]		
7. Free of other bias?		
8. A priori protocol?[d]		
9. A priori analysis plan?[e]		

[a] Some items on low/high risk/unclear scale (double-line border), some on 5 point scale/unclear (single line border), some on yes/no/unclear scale (dashed border). For all items, record "unclear" if inadequate reporting prevents a judgement being made.

[b] For each outcome in the study.

[c] This item is only used for QESs. It is based on a list of confounders considered as important at the outset and defined in the protocol for the review (*assessment against worksheet*).

[d] Did the researchers write a protocol defining the study population, intervention and comparator, primary and other outcomes, data collection methods, etc. in advance of starting the study?

[e] Did the researchers have an analysis plan defining the primary and other outcomes, statistical methods, subgroup analyses, etc. in advance of starting the study?

Risk of bias tool

Studies for which the risk of bias tool is Intended

The risk of bias model is developed by Prof. Barnaby Reeves in association with the Cochrane Non-Randomised Studies Methods Group.[1] This model, an extension of the Cochrane Collaboration's risk of bias tool, covers both risk of bias in randomised controlled trials (RCTs and QRCTs), but also risk of bias in non-randomised studies (QESs).

The point of departure for the risk of bias model is the Cochrane Handbook for Systematic Reviews of interventions (Higgins & Green, 2008). The existing Cochrane risk of bias tool needs elaboration when assessing non-randomised studies because, for non-randomised studies, particular attention should be paid to selection bias / risk of confounding. Additional items on confounding are used only for non-randomised studies (QESs) and are not used for randomised controlled trials (RCTs and QRCTs).

Assessment of Risk of Bias

Issues when using modified RoB tool to assess included non-randomised studies:

- Use existing principle: score judgement and provide information (preferably direct quote) to support judgement.
- Additional items on confounding used only for non-randomised studies (QESs).
- 5-point scale for <u>some</u> items (distinguish "unclear" from intermediate risk of bias).
- Keep in mind the general philosophy – assessment is <u>not</u> about whether researchers could have done better but about risk of bias; the assessment tool must be used in a standard way irrespective of the difficulty / circumstances of investigating the research question of interest or the study design used.
- Anchors: "1/No/low risk" of bias should correspond to a high quality RCT. "5/high risk" of bias should correspond to a risk of bias that means the findings should not be considered (too risky, too much bias, more likely to mislead than inform).

1. Sequence generation

- Low/high/unclear RoB item.
- Always high RoB (not random) for a non-randomised study.
- Might argue that this item is redundant for QES since it is always high – but it is important to include it in an RoB table ('level playing field' argument).

2. Allocation concealment

- Low/high/unclear RoB item.
- Potentially <u>low</u> RoB for a <u>non-randomised study</u>, e.g., quasi-randomised (too high RoB to sequence generation) but concealed (reviewer judges that the people making decisions about including participants didn't know how allocation was being done, e.g., odd/even date of birth/hospital number).

---

[1] This risk of bias model was introduced by Prof. Reeves at a workshop on risk of bias in non-randomised studies at SFI Campbell, February 2011. The model is a further development of work carried out in the Cochrane Non-Randomised Studies Method Group (NRSMG).

3. RoB from confounding (additional item for QES; assess for each outcome)

- Assumes a pre-specified list of potential confounders defined in the protocol
- Low(1) / 2 / 3 / 4 / high(5) / unclear RoB item
- Judgement needs to factor in:
  - proportion of confounders (from pre-specified list) that were considered
  - whether most important confounders (from pre-specified list) were considered
  - resolution/precision with which confounders were measured
  - extent of imbalance between groups at baseline
  - care with which adjustment was done (typically a judgement about the statistical modeling carried out by authors)
- Low RoB requires that all important confounders are balanced at baseline (not primarily/not only a statistical judgement OR measured 'well' and 'carefully' controlled for in the analysis.

Assess against pre-specified worksheet. Reviewers will make an RoB judgement about each factor first and then 'eyeball' these for the judgement RoB table.

4. RoB from lack of blinding (assess for each outcome)

- Low(1) / 2 / 3 / 4 / high(5) / unclear RoB item
- Judgement needs to factor in:
  - nature of outcome (subjective / objective; source of information)
  - who was / was not blinded and the risk that those who were not blinded could introduce performance or detection bias
  - see Ch.8

5. RoB from incomplete outcome data (assess for each outcome)

- Low(1) / 2 / 3 / 4 / high(5) / unclear RoB item
- Judgement needs to factor in:
  - reasons for missing data
  - whether amount of missing data balanced across groups, with similar reasons
  - whether censoring is less than or equal to 25% and has been taken into account
  - see Ch.8

6. RoB from selective reporting (assess for each outcome)

- Low(1) / 2 / 3 / 4 / high(5) /unclear RoB item
- Judgement needs to factor in:
  - existing RoB guidance on selective outcome reporting (see Ch.8)
  - also, extent to which analyses (and potentially other choices) could have been manipulated to bias the findings reported, e.g., choice of method of model fitting, potential confounders considered / included
  - look for evidence that there was a protocol in advance of doing any. analysis / obtaining the data (difficult unless explicitly reported); QES very different from RCTs. RCTs must have a protocol in advance of starting to recruit (for REC/IRB/other regulatory approval); QES need not (especially older studies).
  - hence, separate yes/no items asking reviewers whether they think the researchers had a pre-specified protocol and analysis plan.

7. RoB from other bias

- Low(1) / 2 / 3 / 4 / high(5) /unclear RoB item
- Judgement needs to factor in:
    - existing RoB guidance on other potential threats to validity (see Ch.8)
    - also, assess whether suitable cluster analysis is used (e.g., cluster summary statistics, robust standard errors, the use of the design effect to adjust standard errors, multi-level models and mixture models), if assignment of units to treatment is clustered.

Confounding Worksheet

**Assessment of how researchers dealt with confounding**	
Method for *identifying* relevant confounders described by researchers:<br>yes<br><br>no<br><br>If yes, describe the method used:	☐<br>☐
Relevant confounders described:<br>yes<br><br>no<br><br>List confounders described on next page	☐<br>☐
Method used for controlling for confounding<br><br>At design stage (e.g., matching, regression discontinuity, instrument variable):<br><br>...<br>...<br>...<br><br>At analysis stage (e.g., stratification, regression, difference-indifference):<br><br>...<br>...<br>...	

Describe confounders controlled for below	

Confounders described by researchers

Tick (yes[0]/no[1] judgement) if confounder considered by the researchers [Considered].

Score (1[good precision] to 5[poor precision]) precision with which confounder measured.

Score (1[balanced] to 5[major imbalance]) imbalance between groups.

Score (1[very careful] to 5[not at all careful]) care with which adjustment for confounder was carried out.

**Confounder**	Considered	Precision	Imbalance	Adjustment
Gender	☐	☐	☐	☐
Age	☐	☐	☐	☐
Grade level	☐	☐	☐	☐
Socioeconomic background	☐	☐	☐	☐
Performance at baseline	☐	☐	☐	☐
Unobservables[2]	☐	Irrelevant	☐	☐
Other:	☐	☐	☐	☐

User Guide for Unobservables

Selection bias is understood as systematic baseline differences between groups and can therefore compromise comparability between groups. Baseline differences can be observable (e.g., age and gender) and unobservable (to the researcher; e.g., 'appearance'). There is no single non-randomised study design that always solves the selection problem. Different designs solve the selection problem under different assumptions and require different types of data. There can be particularly great variations in how different designs deal with selection on unobservables. The "right" method depends on the model generating participation, i.e. assumptions about the nature of the process by which participants are selected into an intervention.

As there is no universally correct way to construct counterfactuals, we will assess the extent to which the identifying assumptions (the assumption that makes it possible to identify the counterfactual) are explained and discussed (preferably by the authors in an effort to justify their choice of method). We will look for evidence of authors using the following examples (this is NOT an exhaustive list):

---

[2] See User guide for unobservables.

Natural Experiments

Discuss whether they face a truly random allocation of participants and that there is no change of behavior in anticipation of, e.g., policy rules.

Instrument Variable (IV)

Explain and discuss the assumption that the instrument variable does not affect outcomes other than through their effect on participation.

Matching (including propensity scores)

Explain and discuss the assumption that there is no selection on unobservables, only selection on observables.

(Multivariate, Multiple) Regression

Explain and discuss the assumption that there is no selection on unobservables, only selection on observables. Further discuss the extent to which they compare comparable people.

Regression Discontinuity (RD)

Explain and discuss the assumption that there is a (strict) RD treatment rule. It must not be changeable by the agent in an effort to obtain or avoid treatment. Continuity in the expected impact at the discontinuity point is required.

Difference-in-Difference (Treatment-control-before-after)

Explain and discuss the assumption that outcomes of participants and nonparticipants evolve over time in the same way.
