# Supplementary material for: Targeted school‐based interventions for improving reading and mathematics for students with or at risk of academic difficulties in Grades K‐6: A systematic review
Source: Campbell Syst Rev. 2021 Apr 6;17(2):e1152. doi: 10.1002/cl2.1152 (PMC8356298; doi:10.1002/cl2.1152)
Supplement: Supplementary file 3 — Supporting information [file CL2-17-e1152-s002.pdf]

Online Appendix C: Studies with overlapping samples and lacking information

Table A1 lists the studies that we did not include in the meta-analysis because we could not obtain enough information to calculate an effect size, or because they used samples that overlapped with other included studies. The table also contains information about the studies with which the latter had overlapping samples. Some of these studies were included in the meta-analysis, and some were comparison designs not included in the meta-analysis.

Table A1. Studies with overlapping samples

Authors	Country	Test subject	Reason for not being included in meta-analysis
Baker et al. (2015)	US	Reading	Overlapping with Smith et al. (2016).
Chaparro et al. (2012)	US	Reading	Overlapping with Smith et al. (2016).
Fien et al. (2015)	US	Reading	Overlapping with Smith et al. (2016).
Fives (2016)	Ireland	Reading	Overlapping with Fives et al. (2013).
Foster (2014)	US	Math	Overlapping samples with Morris et al. (2007).
Fuchs et al. (2002)	US	Reading	Overlapping with Fuchs et al. (2001).
Fuchs et al. (2015)	US	Math	Overlapping samples with Fuchs et al. (2013, 2014b).
Gunn et al. (2000)	US	Reading	Overlapping samples with Gunn et al. (2005).
Gunn et al. (2002)	US	Reading	Overlapping samples with Gunn et al. (2005).
Hagan-Burke et al. (2013)	US	Reading	Overlapping with Simmons et al. (2011).
Kamps & Greenwood (2005)	US	Reading	Overlapping with Kamps et al. (2008).
Linan-Thompson et al. (2006)	US	Reading	Overlapping with Vaughn et al. (2006b) and Vaughn et al. (2006c).
Linan-Thompson et al. (2007)	US	Reading	Overlapping with Vaughn et al. (2006a).
Powell et al. (2015)	US	Math	Overlapping with Fuchs et al. (2014).
Sáenz et al. (2005)	US	Reading	Overlapping with Sáenz (2002).
Tong et al. (2011)	US	Reading	Overlapping with Tong et al. (2008) and Tong et al. (2011).
Wanzek et al. (2011)	US	Reading	Overlapping with Vaughn et al. (2010).
Babinski et al. (2018)	US	Reading	Lack information to calculate an effect size.
Baenen et al. (1997)	US	Reading	Lack information to calculate an effect size.
Boardman et al. (2016)	US	Reading	Lack information to calculate an effect size.
Bramlett (1992)	US	Reading	Lack information to calculate an effect size.
Carlo et al. (2004)	US	Reading	Lack information to calculate an effect size.
Carter & Russel (1985)	US	Reading	Lack information to calculate an effect size.

Authors	Country	Test subject	Reason for not being included in meta-analysis
Chiang (2007)	US	Math, reading	Lack information to calculate an effect size.
Chin et al. (2012)	US	Reading	Lack information to calculate an effect size.
Chodkiewicz & Boyle (2016)	Australia	Math, reading	Lack information to calculate an effect size.
Cobb (2001)	US	Reading	Lack information to calculate an effect size.
Glassman (1988)	US	Math, reading	Lack information to calculate an effect size.
Gottshall (2007)	US	Reading	Lack information to calculate an effect size.
Greenwood (1991)	US	Math, reading	Lack information to calculate an effect size.
Hummel & Hahn (1982)	US	Math	Lack information to calculate an effect size.
Mackey et al. (2017)	US	Math	Lack information to calculate an effect size.
Miller & Connolly (2013)	UK	Reading	Lack information to calculate an effect size.
Peña (2008)	US	Reading	Lack information to calculate an effect size.
Scientific Learning Corporation (2006)	US	Reading	Lack information to calculate an effect size.
Stavros (1989)	US	Math, reading	Lack information to calculate an effect size.
Taub et al. (2007)	US	Reading	Lack information to calculate an effect size.
Taub et al. (2015)	US	Math	Lack information to calculate an effect size.
Woods (1986)	Australia	Math, reading	Lack information to calculate an effect size.
Ysseldyke et al. (2003)	US	Math	Lack information to calculate an effect size.
Zunker (2008)	US	Math	Lack information to calculate an effect size.
