# Supplementary material for: Targeted school‐based interventions for improving reading and mathematics for students with or at risk of academic difficulties in Grades K‐6: A systematic review
Source: Campbell Syst Rev. 2021 Apr 6;17(2):e1152. doi: 10.1002/cl2.1152 (PMC8356298; doi:10.1002/cl2.1152)
Supplement: Supplementary file 4 — Supporting information [file CL2-17-e1152-s003.pdf]

Online Appendix D: Coding scheme

The following information was extracted from all studies included in the meta-analysis (see supplementary material Online Appendix H: *Data set Effect sizes from intervention-control studies* and Online Appendix I: *Data set Effect sizes from comparison design studies* for the data used in the analyses):

1. Report characteristics

1.1. Authors
1.2. Title
1.3. Publication year
1.4. Language
1.5. Publishing status (published in scientific journal or not)
1.6. Outlet/Type of publication (e.g., journal name/report series/dissertation)

2. Study characteristics

2.1. Study location (country and state/city)
2.2. Study design (RCT, QRCT, QES)
2.3. Describe treatment assignment
2.4. Number of separate sites included in the study (classrooms, schools, districts, states or regions, countries)
2.5. If multiple sites, describe if there were differences in assignment between sites?

3. Participant characteristics

3.1. Describe the target group of the intervention, e.g., students with specific learning difficulties, low achievement, low SES etc.
3.2. Gender (share of girls)
3.3. Age distribution (min, max, mean)
3.4. Grade distribution (min, max, mean)
3.5. Ethnic, cultural, and language background (share of students with a different background than the majority student population)
3.6. Socioeconomic status (share low income, share low parental education, share low status parental occupation)

4. Intervention characteristics

4.1. Name of intervention
4.2. Describe the instructional methods used in the intervention and any differences between intervention and control groups regarding these methods. State explicitly if there are no differences.
4.3. Describe the content domain targeted by the intervention and any differences between treatment and control groups regarding the content they are instructed in. State explicitly if there are no differences.
4.4. Intervention site (school/other, in which case, specify)
4.5. Group size (e.g. 1:1, 1:2, ...)

4.6. Intervention implementer (Describe the person(s) delivering the intervention: e.g., teachers, college students, researchers).
4.7. Is the implementer trained, and for how long? (the number of hours/days/weeks).
4.8. Duration of intervention in weeks (intended, received)
4.9. Frequency of intervention in sessions (intended, received)
4.10. Intended intensity of intervention in hours per week (intended, received)
4.11. Implementation quality (questions from Wilson, Lipsey, Tanner-Smith, Huang, & Steinka-Fry, 2010): Was the implementation of the programme monitored by the author/researcher or programme personnel to assess whether it was delivered as intended? (Yes/No/Cannot tell)
4.12. Based on evidence or author acknowledgment, was there any uncontrolled variation or degradation in implementation or delivery of treatment, e.g., high dropouts, erratic attendance, treatment not delivered as intended, wide differences between settings or individual providers, etc.? Assume that there is no problem if one is not specified (yes (describe below)/ possible (describe below)/ no, apparently implemented as intended)
4.13. Describe implementation problems, if any.

5. Control/Comparison characteristics

5.1. What is the nature of the control/comparison condition? (Controls do not receive any intervention/treatment/service after the end of intervention; Controls are wait-list controls; Comparison intervention)

6. Outcome measurement (each step is repeated for all relevant outcomes)

6.1. Measurement timing (number of months after end of intervention)
6.2. Name of standardised test
6.3. Subject of standardised test (mathematics, reading)
6.4. Content domain(s) of test (e.g., vocabulary, algebra, general etc)
6.5. Who performs the tests?

7. Sample size

7.1. Sample size used in analysis for outcome measurement (separate for intervention and control groups)

8. Outcomes

8.1. Test score (Code for both pre- and post-intervention tests separate for intervention and control groups if possible. Describe test in terms: continuous/dichotomous; whether high score/1 is desirable; type of statistic, e.g., mean, t-test, beta-coefficient, F-test etc)
8.2. Standard deviation (Code for both pre- and post-intervention tests separate for intervention and control groups if possible)
8.3. Estimation method (e.g. raw means, adjusted means, regression adjusted etc)
8.4. Uncommon standardisation (No/Mention method of standardisation if available standard deviation is other than the raw standard deviation for the intervention and control group)
