# Supplementary material for: Targeted school‐based interventions for improving reading and mathematics for students with or at risk of academic difficulties in Grades K‐6: A systematic review
Source: Campbell Syst Rev. 2021 Apr 6;17(2):e1152. doi: 10.1002/cl2.1152 (PMC8356298; doi:10.1002/cl2.1152)
Supplement: Supplementary file 5 — Supporting information [file CL2-17-e1152-s001.pdf]

Online Appendix E: Description of comparison designs

Table B1 describes the comparison designs in terms of country, test subject, and the two or more alternative interventions the studies examined (i.e., the "Contrast"-column in the table). Note that the table also includes two studies, which contained contrasts from both intervention-control and comparison designs and the three additional comparison designs, which we included in the meta-analysis of group sizes. That is, the below table contains both 104 comparison designs we did not use in any meta-analysis and the 5 studies that we used in at least one meta-analysis.

Table B1. Comparison designs

Authors	Country	Test subject	Contrast
Abbott & Berninger (1999)	US	Reading	Compares structural analysis and alphabet principle training (Structural Analysis) with only alphabetic principle training (Study Skills).
Anthony (2016)	US	Math, reading	Compares CAI in phonological awareness delivered by Earobics Step 1 with CAI in mathematics delivered by Building Blocks.
Baker et al. (2012)	US	Reading	Compares 'bilingual programs' (daily reading instructions in Spanish and English) and an English-only reading program for Spanish-speaking ELs.
Baroody et al. (2013)	US	Math	Compares computer-assisted structured discovery of the add-1 rule with unstructured discovery learning of this regularity.
Berninger et al. (2000)	US	Reading	This 3-layer intervention varies across intervention groups in the second layer. 7 treatment groups receive different combinations of single or multiple approaches to reading and spelling instructions built on connections between units of written and spoken words.
Brailsford et al. (1984)	Canada	Reading	Compares cognitive strategy training with remedial reading.
Brown et al. (2005)	US	Reading	Compares one-to-one tutoring lessons with small-group reading intervention.
Brush (1997)	US	Math	Compares students who were paired either homogeneously or heterogeneously based on ability. Students were asked to complete math activities delivered by an integrated learning system.
Burns (2012)	US	Reading	Compares a systematic transition intervention designed for Spanish-speaking ELLs with the standard school-based intervention. Both conditions receive small-group instruction as a supplement to their core reading program.
Bussjaeger (1993)	US	Reading	Compares Project Read phonology instruction with literature-based instruction.

Authors	Country	Test subject	Contrast
Cantrell et al. (2014)	US	Reading	Both groups benefit from a school-wide model that involves professional development for all content teachers in content area literacy. The experimental group also receive a targeted intervention (Learning Strategies Curriculum).
Caputo (2007)	US	Math	Compares the Accelerated Math Program with Delaware Procedural Fluency Workbook Program.
Cook & Welch (1980)	US	Reading	Compares three treatments: Auditory Training and Reading, Visual Training and Reading, and Reading Only.
Cox (1997)	US	Reading	Compares two reading comprehension instructions: Project Read and visualization and verbalization comprehension strategy.
Coyne et al. (2007)	US	Reading	Compares two small-group vocabulary interventions: extended instruction and embedded instruction.
Coyne et al. (2013a)	US	Reading	Compares two ways of receiving the intervention, where one group receives systematic adjustments based on student performance while the other group receives no instructional modifications.
Coyne et al. (2013b)	US	Reading	Compares Early Reading Intervention with a school-designed typical practice intervention.
Doss (2015)	US	Reading	Compares three conditions: one group receives a basic level of intervention, which is the lower level of decoding, the second group receives the skills level of intervention, which is the higher level of decoding. The control group receives a computer-based intervention.
Duerr (2008)	US	Reading	Both groups receive the Reading Recovery intervention where the classroom teachers of the treatment group receive weekly meetings with the Reading Recovery teachers, the teachers of the control group do not.
Faggella-Luby & Wardwell (2011)	US	Reading	Compares three conditions: Story Structure (SS), Typical Practice delivered by reading specialists (TP), and Sustained Silent Reading (SSR).
Fantuzzo et al. (1995)	US	Math	Compares three conditions: Parent involvement + reciprocal peer tutoring, parent involvement, and practice control.
Fenty et al. (2015)	US	Reading	Compares three conditions: a teacher-led group, a computer-assisted text-equivalent group, and a computer-assisted time-equivalent group.
Fisher et al. (2007)	Australia	Reading	Compares the effectiveness of two tutoring programs for children with persistent spelling difficulties: Look-Say-Cover-Write-Say-Check and Old Way/New Way - Mediational Learning.
Foorman et al. (1997)	US	Reading	Compares three different reading interventions: synthetic phonics, analytic phonics, and sight-word
Fryer et al. (2016)	US	Math, reading	Compares vertical versus horizontal incentives in education.

Authors	Country	Test subject	Contrast
Fuchs et al. (1985)	US	Reading	Compares three goal ambitiousness groups: Low, moderately ambitious, and highly ambitious.
Fuchs et al. (2006)	US	Math, reading	Compares computer-assisted instruction in math and spelling.
Fuchs et al. (2008)	US	Math	Compares four conditions: Word recognition tutoring or 1 of 3 computation tutoring conditions: fact retrieval, procedural computation and computational estimation, and combined (fact retrieval + procedural computation and computational estimation).
Gilbert et al. (2013)	US	Reading	Compares different tiers in a multitiered supplemental tutoring program within a first-grade responsiveness-to-intervention prevention model. (One intervention-control contrast included in the meta-analysis).
Helf et al. (2009)	US	Reading	Compares 1:1 tutoring lessons with 1:3 tutoring lessons.
Hendricks et al. (2006)	US	Math, reading	Compares a patterning intervention with instruction in the academic subject matter, the teachers thought most useful.
Hill (2009)	US	Math	Compares students who have participated in synchronous and asynchronous online learning groups.
Hogan-Gancarz (1999)	US	Math	Compares a group receiving an individualized math program and memory strategy instruction to a group receiving only the individualized math program.
Holmes (1985)	US	Reading	Compares four small-group conditions delivered by the experimenter: Strategy plus materials, strategy only, materials only, and control. However, control group also practices certain questions and is anticipated to be affected; i.e., receives an intervention.
Hudson et al. (2011)	US	Reading	Compares two small-group interventions: An accuracy condition and an accuracy + automaticity condition.
Hunt (2014)	US	Math	Compares to conditions: Core instruction and core + intervention instruction.
Jessup (2017)	US	Reading	Compares direct instruction and repeated reading for fluency.
Jitendra et al. (2007)	US	Math	Compares a single strategy (schema-based instruction) and a multiple strategy (general strategy instruction).
Jitendra et al. (2013a)	US	Math	Compares two small-group tutoring interventions: A school-provided standards-based curriculum (SBC) and a schema-based instruction (SBI) curriculum.
Jitendra et al. (2013b)	US	Math	Compares two small-group tutoring interventions: A schema-based instruction (SBI) curriculum and a standard-based, school-provided curriculum. Extended focus on mathematical word problem solving in the SBI-group.
Joiner (2012)	US	Reading	Compares Reading Recovery intervention with Georgia's Early Intervention Program (Non-Reading Recovery EIP)

Authors	Country	Test subject	Contrast
Kamps et al. (2007)	US	Reading	Compares a direct instruction approach using "integrated curriculum", and a balanced literacy approach.
Kamps et al. (2008)	US	Reading	Compares two small-group interventions: A direct instruction approach using "integrated curriculum", and a condition using either Open Court curriculum or Guided Reading.
Kestel & Forgasz (2018)	Australia	Math	Compares two modes of delivery: personal videoconferencing and face-to-face delivery.
Kim et al. (2009)	US	Reading	Compares the READ 180 intervention to a district after-school program in which teachers are able to select from 16 different enrichment activities.
Lalley & Miller (2006)	US	Math	Compares a pre-teaching condition with a re-teaching condition.
Lamminmäki et al. (1997)	Chile	Math, reading	Compares Neurocognitive Treatment and Homework Assistance programs.
Leh & Jitendra (2012)	US	Math	Compares computer-mediated instruction and teacher-mediated instruction.
Little et al. (2012)	US	Reading	Compares Early Reading Intervention modified in response to student performance and a supplemental reading intervention (regrouping and curriculum pacing adjustments).
Lovett et al. (1990)	Canada	Reading	Compares three interventions: Two word recognition and spelling training programs and a problem solving and study skills training program
Lovett et al. (1994)	Canada	Reading	Compares 2 forms of word identification training to promote transfer of learning by children with dyslexia and a study skills control program.
Lovett & Steinbach (1997)	Canada	Reading	Compares two word identification training programs and a study skills program.
Lovett et al. (2000)	Canada	Reading	Compares the efficacy of a combination of phonological and strategy-based remedial approaches for reading disability (RD) to that of each approach separately.
Lovett et al. (2008)	Canada	Reading	Compares three reading interventions and a special education reading control program.
Lysynchuk et al. (1990)	Canada	Reading	Compares reading-strategy instruction and reading practice. Control subjects were exposed to the same materials as reciprocally trained students but were given no strategy instruction.
Mathes et al. (2005)	US	Reading	Compares two small-group interventions: Proactive Reading and Responsive Reading. The intervention groups and control group all receive enhanced classroom instruction.
McArthur et al. (2015)	Australia	Reading	Compares sight word training and phonics training in children with dyslexia. Three different intervention groups receive the training in different orders.

Authors	Country	Test subject	Contrast
McDermott & Stegemann (1987)	US	Math	Compares three instructional methods: Computer-assisted instruction (CAI) with a reward game, computer-assisted instruction without a reward game, and paper-and-pencil.
McMaster et al. (2005)	US	Reading	For students not responding sufficiently to PALS, three individualized treatments are compared: PALS, Modified PALS, or tutoring by an adult.
Miller (2009)	US	Reading	Compares two instructional approaches: A basal student-directed approach and an explicit teacher-directed approach.
Morris et al. (2012)	US, Canada	Reading	Compares the effectiveness of two multiple-component intervention programs for children with reading disabilities (PHAB + RAVE-O; PHAB + WIST) to alternate (CSS, MATH) and phonological control programs.
Morse-Taylor (2010)	US	Reading	Compares four EIP grouping models: Augmented, self-contained, reduced, and regular.
Nash & Snowling (2006)	UK	Reading	Compares the effects of two different methods of teaching vocabulary on vocabulary knowledge and reading comprehension.
O'Connor et al. (2005)	US	Reading	Multi-layered intervention, the first layer being professional development. The second layer is direct intervention through small-group instruction.
O'Connor et al. (2014a)	US	Math, reading	Compares the effects of INSIGHTS and a supplemental reading program in increasing academic achievement, sustaining attention and reducing disruptive behavior problems.
O'Connor et al. (2014b)	US	Math, reading	Compares the effects of INSIGHTS and a supplemental after-school reading program in enhancing the academic development of shy children.
Olympia et al. (1994)	US	Math	Examined effectiveness of self-managed individual and group contingency procedures in improving completion and accuracy rates of daily mathematics homework assignments.
Oudeans (2003)	US	Reading	Compares two sequences for integrating and teaching letter-sound correspondences and phonological blending and segmenting.
Parrila et al. (1999)	Canada	Reading	Compares the PASS Reading Enhancement Program and Meaning-Based Reading intervention.
Pascarella et al. (1983)	US	Reading	Compares two reading instruction programs on context cue use differing only in extent of student control over determination of errors.
Pasnak et al. (2015)	US	Math, reading	Compares four conditions of small-group instruction: In sequences, reading, mathematics, and social studies.
Pavchinski (1988)	US	Math	Compares operant procedures, cognitive behavior modification and a condition consisting of direct instruction of math skills.
Pflaum et al. (1982)	US	Reading	Compares four comprehension-facilitating conditions (word identification and meaning aids, sentence aids, purpose-setting aids, and prior-knowledge aids).

Authors	Country	Test subject	Contrast
Pinnell (1988)	US	Reading	Compares Reading Recovery, an instructional approach which deliberately links reading and writing, to an alternative compensatory program which focuses on skills and isolated information.
Pinnell et al. (1988)	US	Reading	Compares Reading Recovery to an alternative compensatory program.
Reutzel et al. (2012)	US	Reading	Both groups follow the state-approved Comprehensive Core Reading Program (CCRP) and receive an additional 30 min of supplemental reading instruction every day. The experimental group receives the guided, silent reading intervention, *Reading Plus,* and the control group receives *Soar to Success*, *Essential Elements of Reading: Vocabulary*, *Voyager Passport* or *Earobics.*
Ritchey et al. (2012)	US	Reading	Compares a multicomponent supplemental intervention targeting fluency and expository comprehension of science texts to a group that group that receives progress monitoring.
Ruggiero (2004)	US	Reading	Partial inclusion support services as compared to support service instruction provided on a pull-out or self-contained classroom basis.
Russel & Ford (1983)	US	Reading	Compares a group of students working on a one-to-one basis with tutors and a group working in a small group with a teacher.
Rzoska & Ward (1991)	New Zealand	Math	Compares cooperative and competitive group-learning.
Saine et al. (2011)	Finland	Reading	Compares two conditions for at-risk readers: regular remedial reading intervention, computer-assessed reading intervention.
Saine et al. (2013)	Finland	Reading	Compares two conditions at-risk readers: a regular remedial reading intervention, a computer-assisted remedial reading intervention.
Santoro et al. (2006)	US	Reading	Compares three conditions: two experimental conditions that focus on increasing beginning reading skills and one comparison group that receive commercial reading program's sounds and letters module.
Schwartz et al. (2012)	US	Reading	Compares the effect of group sizes, teaching Reading Recovery in a 1:1 and a small-group instructional format with teacher-student ratios of 1:2, 1:3, or 1:5.
Simmons et al. (2007)	US	Reading	Compares three conditions: (a) 30 min with high design specificity (30/H), (b) 15 min with high design specificity plus 15 min of non-code-based instruction (15/H+15), and (c) a commercial comparison condition that reflected 30 min of moderate design specificity instruction (30/M).
Simmons et al. (2011)	US	Reading	Compares the effects of 2 supplemental reading interventions: An explicit/systematic commercial program and a school-designed practice intervention.
Simmons et al. (2015)	US	Reading	Compares students from four randomized control trials, and divide them into two groups: Students who received ERI with ongoing adjustments (ERI-A) and students who received ERI implemented conventionally (ERI–matched peers).

Authors	Country	Test subject	Contrast
Slavin et al. (2011)	US	Reading	Compares the effect of transitional bilingual education (TBE) and structured English immersion (SEI) on the language and reading performance of Spanish-dominant children.
Smith-Davis (2007)	US	Reading	Compares the effect of the Success for All reading program to other evidenced-based reading programs.
Soltero-Gonzaléz et al. (2016)	US	Reading	Compares the biliteracy outcomes for students in a paired literacy model to those of students in a sequential literacy model
Spies et al. (2018)	US	Reading	Students receive a transitional bilingual education (TBE) or a TBE-enhanced program to gains English language and literacy skills.
Tong et al. (2008a)	US	Reading	Students receiving a structured developmental bilingual education program are compared with students receiving typical-practice model of late-exit transitional bilingual education program.
Tong et al. (2008b)	US	Reading	Compares ELLs students receiving an enhanced Transitional bilingual education (TBE) and structured English immersion (SEI) program and ELLs students receiving typical-practice of TBE and SEI.
Torgerson et al. (2011)	UK	Math	Compares 1:1 tutoring with 1:2 and 1:3 tutoring.
Torgesen et al. (2001)	US	Reading	Compares Auditory Discrimination in Depth Program to Embedded Phonics.
Torgesen et al. (2003)	US	Reading	Compares two computer supported approaches: Auditory Discrimination in Depth and Read, Write, and Type.
Tremblay (2013)	Belgium	Math, reading	Compares co-teaching inclusion and solo-taught special education.
Vaughn et al. (2003)	US	Reading	Compares the effects of three grouping formats: 1:1, 1:3, and 1:10.
Wade & Kass (1986)	US	Reading	Compares remediation of hypothesized component deficits given prior to remediation of known academic deficiencies to remediation of known academic deficiencies alone.
Wages (2013)	US	Reading	Compares the content-based English as a second language program to the late exit transitional bilingual program.
Wang et al. (2016)	US	Reading	Compares academic effects of Language! Live with Corrective Reading, Soar to Success, and Wilson reading intervention programs.
Wanzek & Vaughn (2008)	US	Reading	Compares three conditions: (a) a single dose of intervention, (b) a double dose of intervention, or (c) a school intervention.
Wesson (1983)	US	Reading	Compares two student self-management techniques: student charting and student selection of instructional activities.
Wilson & Kaplan (1994)	Canada	Reading	Compares gained reading skills of two treatment groups receiving individual sessions of sensory integration (therapy) or individual tutoring.

**Authors**	**Country**	**Test subject**	**Contrast**
Wise (2005)	US	Reading	Compares four conditions: PHAB/DI + CSS program, PHAB/DI + WIST, PHAB/DI + RAVE-O, and comparison group (math instruction program + CSS program).
Wise et al. (1997)	US	Reading	Compares phonological training with an articulatory component with phonological awareness training without a specific speech-motor component.
Woodward (2006)	US	Math	Compares an integrated approach (strategies and timed practice drills) with timed practice drills only for teaching multiplication facts.
Xin (1999)	US	Math	Compares cooperative learning to whole-class learning supported by computer technology.
Xin et al. (2011)	US	Math	Compares a conceptual model-based problem-solving approach to a general heuristic instructional approach.
