# Supplementary material for: Targeted school‐based interventions for improving reading and mathematics for students with or at risk of academic difficulties in Grades K‐6: A systematic review
Source: Campbell Syst Rev. 2021 Apr 6;17(2):e1152. doi: 10.1002/cl2.1152 (PMC8356298; doi:10.1002/cl2.1152)
Supplement: Supplementary file 7 — Supporting information [file CL2-17-e1152-s005.pdf]

Online Appendix G: Studies with a too high risk of bias rating

In Table E1, we listed the studies that we assessed had too high risk of bias. That is, a rating of 5 on at least one item. There may have been more than one effect size that received a rating of 5, but the table below contains only the primary reason plus an explanatory comment.

Table E1. Studies not included in meta-analysis because of too high risk of bias.

	Study	Country	Test subject	Study design	Rated 5 on item?	Comment
1	Aagard et al. (2016)	US	Math	QES	Confounding	Compare with one control district. Pre-test imbalances, otherwise no confounders considered. Analyse gain scores, otherwise no adjustment for confounders.
2	Adams (2011)	US	Reading	QES	Confounding	No balance test shown and no adjustment for confounders.
3	Algozzine et al. (2009)	US	Reading	QES	Confounding	Imbalances at pre-test and no adjustment for confounders.
4	Allen (2018)	US	Reading	QES	Other bias	One intervention and one control school.
5	Allinder et al. (2000)	US	Math	QES	Confounding	One part - the assignment of teachers to one of two interventions - is randomly assigned, the other part - the assignment of teachers to intervention or control - unclear but probably not randomly assigned, as this is not mentioned. No other method used to control for confounding.
6	Anderson et al. (1995)	US	Reading	QES	Other bias	Teachers in the school were divided into teams assigned to different students. Treated were selected from one team and control from the other. High risk of bias from team effects.
7	Arnbak & Elbro (2000)	Denmark	Reading	QES	Confounding	Imbalances at pre-test and no adjustment for confounders.
8	Arnold (2013)	US	Math	QES	Confounding	No balance test shown.
9	Arnold (2009)	US	Reading	QES	Other bias	One intervention and one control school.
10	Banerji (1988)	US	Math, reading	QES	Other bias	Treated are from one academic year and control are taken from the preceding academic year. High risk of bias from cohort effects.
11	Barrett (2011)	US	Math	QES	Other bias	One intervention and one control school.

	Study	Country	Test subject	Study design	Rated 5 on item?	Comment
12	Bartik & Lachowska (2014)	US	Math, reading	QES	Confounding	Regression discontinuity using previous grades test score as cutoff. Use the whole sample and not just students close to the cutoff, i.e., students have quite different characteristics at pre-test. Do not consider age among the confounders. Show regression results with either quadratic or cubic terms, and a linear specification for one year in math; no means by group reported. Very large differences between the linear and the quadratic/cubic specifications.
13	Bass et al. (1986)	US	Math, reading	QES	Confounding	Unclear how classes were assigned to the intervention, large imbalances at pre-test and no adjustment for confounders.
14	Bates et al. (2016)	US	Reading	QES	Confounding	Control group consist of students who were near eligible for Reading Recovery. Selection process makes the control group different from the intervention group, as shown by the pre-test imbalance.
15	Bauer (2014)	US	Math, reading	QES	Confounding	Match on age and gender. Unclear at what time students are matched but probably in grade 11 or 12. The intervention takes place in Kindergarten and students are followed to 11 or 12 grade. No pre-tests and no adjustment for other confounders.
16	Becker (1990)	US	Math	QES	Confounding	No balance test shown and no adjustment for confounders.
17	Becker & Gersten (1982)	US	Math, reading	QES	Confounding	No access to pre-tests and insufficient control for pre-interventions grade level.
18	Bell (2008)	US	Reading	QES	Confounding	Based on students' initial oral reading fluency scores, the DIBELS assessment places students in one of the three tiers, which are compared to each other. Intervention and control groups are different by design.
19	Bellert (2009)	Australia	Math	QES	Confounding	No balance test shown and no adjustment for confounders.
20	Biemiller & Siegel (1997)	Canada	Reading	QES	Confounding	No adjustment for or description of relevant confounders.

	Study	Country	Test subject	Study design	Rated 5 on item?	Comment
21	Black (2010)	US	Math, reading	QES	Confounding	Compares students with more or less time in special needs education and with different levels of disabilities.
22	Boges (2015)	US	Reading	QES	Confounding	Compares students scoring above (control) and below (intervention) grade level.
23	Bonnville (2013)	US	Reading	QES	Other bias	Intervention group from one cohort and control from another. High risk of bias from cohort effects.
24	Bowey & Hansen (1994)	Australia	Reading	QES	Confounding	Experiment 1 excluded as the intervention was not targeted. Experiment 2 compares intervention and control groups at either different ability levels, or in different grades.
25	Brady et al. (1994)	US	Reading	QES	Confounding	Four schools, where two classes are intervention classes and two control. Match students for analysis after the intervention on age and IQ. No reading pre-test.
26	Brand-Gruwel et al. (1998)	Nether-lands	Reading	QES	Confounding	Schools asked to be treatment or control. Students selected, there are selection criteria but it is not reported if all eligible students are selected.
27	Bribiescas (2012)	US	Math, reading	QES	Other bias	One intervention and one control school.
28	Brigman & Campbell (2003)	US	Math, reading	QES	Confounding	Intervention group from grades 5, 6, 8, and 9 but grades of control students are not mentioned. Present no student demographics by condition and no information about pre-test imbalances.
29	Brigman et al. (2007)	US	Math, reading	QES	Confounding	Intervention group from grades 5, 6, 8, and 9 but the grades of control students are not mentioned. Present no student demographics by condition and large pre-test imbalances in both math and reading.
30	Brown & Felton (1990)	US	Reading	QES	Confounding	No student characteristics shown by condition. Only stated that the students who left after attrition did not differ on age at initial screening or IQ as measured in kindergarten.
31	Brunson (2016)	US	Reading	QES	Confounding	Randomly select treated and non-treated at-risk students after the intervention. Large pre-test imbalances.

	Study	Country	Test subject	Study design	Rated 5 on item?	Comment
32	Bryant et al. (2008a)	US	Math	QES	Confounding	Compare students above and below 25th percentile on a standardized test, where the students scoring below received an intervention. Compare students far away from the cutoff, so by construction, there are large pre-test differences between intervention and control. No other information about balance and unclear what is adjusted for in the analysis.
33	Bryant et al. (2008b)	US	Math	QES	Confounding	Compare students above and below 25th percentile on a standardized test, where the students scoring below received an intervention. Compare students far away from the cutoff, so by construction, there are large pre-test differences between intervention and control. No other information about balance and unclear what is adjusted for in the analysis.
34	Bunn (2006)	US	Reading	QES	Confounding	Do not show demographic statistics or pre-test score by intervention and control group.
35	Burns et al. (2012)	US	Math	QES	Confounding	Compare an intervention group to a control group that participated less in the intervention. No explanation why they participated less, and some pre-test imbalances.
36	Burton (2005)	US	Math, reading	QES	Other bias	One intervention and one control school.
37	Bøg et al. (2019)	Sweden	Reading	QES	Confounding	All the control students are from two earlier cohorts in one of the 3 schools. Only pre-tests and grade considered among relevant confounders.
38	Calhoon (2005)	US	Reading	RCT	Other bias	Four teachers randomly assigned to the intervention and control groups. Students were not randomly assigned and both intervention classrooms were in the same school and both control classrooms were in another school.
39	Calhoun (2007)	US	Reading	QES	Confounding	No demographics or pre-test presented or information on the number of schools in the intervention is provided.
40	Campbell (2001)	US	Reading	QES	Other bias	One intervention and one control school.

	Study	Country	Test subject	Study design	Rated 5 on item?	Comment
41	Cardelle-Elawar (1992)	US	Math	RCT	Other bias	Experiment 1: Two classes were randomly assigned to the intervention group and one to the control group. Experiment 2: control group from one other school than the treated.
42	Cartelli (1980)	US	Reading	QES	Confounding	Unclear assignment procedures. Compare treatment and control over age, IQ, and pre-test scores. Only one pre-test reported out of two, which has a relatively large imbalance (0.46 of the joint pre-test SD).
43	Cazabon et al. (1993)	US	Math, reading	QES	Other bias	Two intervention sites, one covers grade K-3 and one 4-6, but they only compare grade 1-3 to controls, meaning that there is only one intervention site used in the analysis.
44	Center et al. (1995)	Australia	Reading	RCT	Other bias	Teachers were responsible for random assignment, but assignment procedures are unclear. Very large pre-test imbalances.
45	Cesa (2012)	US	Reading	RCT	Other bias	The same three teachers are involved in the two interventions and teach the control students. Assignment procedures are unclear. Groups are small, 6 students in each intervention group and 6 in the control group. Balancing tests not shown.
46	Chappell et al. (2015)	US	Math	QES	Confounding	Propensity score matching but still very large pre-test imbalances after matching.
47	Cho et al. (2015)	US	Reading	QES	Confounding	Secondary analysis using a subsample of Vaughn et al. (2016). Compares adequate and inadequate responders to the intervention and include a typical reader group.
48	Choi & Lemberger (2010)	South Korea	Math, reading	QES	Confounding	All eligible are offered the intervention and the control group are those who reject participation in the intervention but accept being control group.
49	Clark (2017)	US	Math	QES	Other bias	Intervention 1 designed and implemented by one teacher and intervention 2 designed and implemented by another teacher. High risk of bias from teacher effects.

	Study	Country	Test subject	Study design	Rated 5 on item?	Comment
50	Claus & Quimper (1987)	US	Math, reading	QES	Confounding	Students who score below a threshold participated in the intervention. No confounders other than pre-test considered and no adjustment for any confounders.
51	Clipson-Boyles (2000)	UK	Reading	QES	Confounding	School level matching, but difference at pre-test (in particular between matched time intervention and control). No adjustment for confounding. Unclear description of the design. They state that participant loss in any one group meant the withdrawal of the parallel numbers in the other two groups, yet they end up with an uneven number in the groups. Unclear how attrition is distributed across intervention and if students were assigned to the intervention before or after the school was.
52	Colamarino (2008)	US	Math	QES	Confounding	Compare low-achieving students to low- and higher-achieving students. Large pre-test imbalances and no other confounders considered. Same teacher/researcher in both intervention and control group.
53	Commeyras et al. (1992)	US	Reading	QES	Confounding	The teacher was asked to divide learning disabled students from one class into two groups, each group to be comprised of students of varying reading ability. The researcher provide the intervention and the tests.
54	Conring (2010)	US	Math	QES	Other bias	One intervention and one control class.
55	Coratti (2009)	US	Reading	QES	Confounding	Compare a matched and a non-matched sample. Large imbalances for the non-matched sample. The matched sample has some imbalance at pre-test and compares two types of schools, so the intervention effect is at high risk of being confounded by school type, and, as student composition is different, by peer effects. Unclear what variables that are used in the matching and pre-tests seems to be conducted after the intervention has started.

	Study	Country	Test subject	Study design	Rated 5 on item?	Comment
56	Council of Great City Schools (2002)	US	Reading	QES	Confounding	Failed randomisation. Consider only pre-test and grade. Some imbalances on pre-test and by construction none on grade. Unclear number of classes and students that participate.
57	Daunic et al. (2013)	US	Reading	QES	Other bias	One intervention and one control school.
58	Davis (1996)	US	Reading	QES	Other bias	One intervention and one control school.
59	Davis (2014)	US	Reading	QES	Confounding	Only include control students scoring below grade level but some intervention students score above this level. Some pre-intervention imbalances and no adjustment for confounders.
60	Davis (2008)	US	Reading	QES	Confounding	Random selection of students from existing programs. This does not control for selection into these programs. No adjustment for confounders.
61	Dawes (2012)	US	Math, reading	QES	Confounding	Schools select into the intervention. Only special education students in 3rd grade are included, otherwise no demographics are considered. Large imbalance on “Office Discipline Referrals” and no adjustment for confounders.
62	Denton et al. (2006)	US	Reading	QES	Confounding	Intervention and control groups chosen after participation in a previous intervention. Imbalances on age, but not on pre-test scores. No other characteristic shown and no adjustment for confounders in the contrast when the control groups has not received any intervention.
63	Donawerth (2013)	US	Math	QES	Confounding	Different student populations are compared across years. Thus, there can be variation in general academic performance levels between these student populations. No adjustment for confounders.
64	Dougherty Stahl et al. (2012)	US	Reading	QES	Other bias	Two intervention schools and one control school.

	Study	Country	Test subject	Study design	Rated 5 on item?	Comment
65	Ehri et al. (2007)	US	Reading	QES	Confounding	Compromised randomisation and some of the control groups receive other interventions. Consider pre-test scores and there is some imbalance. We cannot use the reported results where pre-test (but not other confounders) are adjusted for due to lack of information. Raw means have too high risk of bias due to imbalance.
66	Englert et al. (1995)	US	Reading	QES	Confounding	Large imbalances for intervention group 1 on ethnicity and grade, and most pre-tests. Intervention group 2 have large imbalances on 5 of 8 pre-tests. No other confounders considered or adjusted for.
67	Ennemoser & Krajewski (2007)	Germany	Math	QES	Confounding	Only gender considered and no adjustment for confounders.
68	Esteves (2008)	US	Reading	RCT	Other bias	Randomise 3 schools to control and 2 to intervention. Parental consent sought after randomisation and unlikely that all eligible students participate. Large imbalance on grade and gender, and some imbalance on the pre-test.
69	Eversole (2010)	US	Reading	QES	Other bias	Compares three cohorts in the same schools before, during, and after implementation of the intervention. High risk of bias from cohort effects.
70	Falke (2012)	US	Reading	QES	Confounding	Compare an intervention group with similar students who did not utilize the intervention tool. High risk of selection into intervention.
71	Felton (1993)	US	Reading	QES	Confounding	Schools determine who gets which intervention. No adjustment for confounders.
72	Fielding-Barnsley & Hay (2012)	US	Reading	QES	Confounding	No confounders considered or adjusted for except grade (all from year one)
73	Flores (2015)	US	Reading	QES	Other bias	Compares different cohorts, so high risk of bias from cohort effects. No adjustment for confounders exempt a gain score comparison.
74	Foorman et al. (1998)	US	Reading	QES	Other bias	One control school.

	Study	Country	Test subject	Study design	Rated 5 on item?	Comment
75	Frantz (2000)	US	Reading	QES	Confounding	Matching but risk of selection high, as teachers choose who should be in treatment and control groups. Large pre-intervention imbalances.
76	Friesen & Der (1984)	Canada	Reading	QES	Confounding	No explanation of why certain students end up in the treatment and control groups and no adjustment for confounders.
77	Galluzzo (2010)	US	Reading	QES	Confounding	Consider only pre-test, which has a large imbalance.
78	Garcia (2012)	US	Reading	QES	Other bias	Control group from the previous school year. High
79	Gerber et al. (2004)	US	Reading	QES	Confounding	Selected lowest performing 20%, but allowed teachers to substitute/swap students. Control group are randomly identified, but are children who are better performing.
80	Gifford (2004)	US	Reading	QES	Confounding	Participants are the students in the researcher's special education class. Large pre-test and ethnicity imbalance. No adjustment for confounders.
81	Glaeser (1998)	US	Reading	QES	Other bias	Two intervention classes and one control class
82	Gonzalez (1996)	US	Math, reading	QES	Confounding	In the part we could use, the authors compare students whose parents refused participation in the language program with those whose parents accepted. No adjustment for confounding.
83	Gordon & Armour-Thomas (2006a)	US	Math	QES	Confounding	Teachers for the treatment condition either self-selected into the study or were recommended by their principals. No teacher demographics reported. Students were matched with comparison students in other classrooms in the school on ethnicity, gender, free or reduced-priced lunch eligibility, and end-of year math achievement level. No information on imbalance other than group per cents of matching variables are reported. Not reported how students are chosen from the experimental classes (unless all are included, but this seems unlikely given the implied class sizes).

	Study	Country	Test subject	Study design	Rated 5 on item?	Comment
84	Gottesman et al. (1983)	US	Math, reading	QES	Confounding	There is no pre-treatment descriptive statistics on any confounder for the control group, and no adjustment for confounders.
85	Graham et al. (2007)	Australia	Math, reading	QES	Confounding	Control group is high/average achieving students, which are not comparable to intervention students.
86	Graham & Pegg (2010)	Australia	Math	QES	Confounding	Low-achieving students compared to normal achieving students at the same age from the same schools.
87	Graham & Pegg (2013)	Australia	Math	QES	Confounding	Low-achieving students compared to normal achieving students at the same age from the same schools.
88	Grant (1985)	US	Reading	QES	Confounding	Match on IQ and test scores but the intervention group is selected by kindergarten teachers as being at risk of failure, whereas the control group is not at risk. Likely to be unobservable factors that differ between groups, even if IQ differences are small. Other imbalances large.
89	Graves et al. (2011)	US	Reading	RCT	Incomplete outcome data	In Study 2, attrition is 10 out of 60 in Study 2, all of which are in the control group. The differential attrition creates imbalances between the groups. (Study 1 has no standardised test.)
90	Greenwood et al. (1989)	US	Math, reading	RCT	Incomplete outcome data	Attrition rates 44.2% for control and 68.2% for treated by the end of 4th grade.
91	Greenwood et al. (1984)	US	Math, reading	QES	Other bias	One intervention and one control school (Experiment 1). In Experiments 2 and 3, students are their own controls.
92	Gretzula (2007)	US	Math, reading	QES	Confounding	Large imbalances on several confounders, including pre-tests. Adjustment for age only.
93	Guinn (2009)	US	Math, reading	QES	Confounding	Separate gain score analysis by gender, otherwise no confounders considered or adjusted for.
94	Guthrie et al (2009)	US	Reading	QES	Other bias	Two intervention and one control school.

	Study	Country	Test subject	Study design	Rated 5 on item?	Comment
95	Gutiérrez (2012)	US	Math, reading	QES	Confounding	Mentored and not-mentored students from the same school are matched on grade level, racial, socioeconomic and at-risk status. Matching variable unclear for the latter two. No pre-tests considered and no further adjustment for confounding.
96	Gutman (2011)	US	Reading	QES	Confounding	No information about balance on relevant confounders and no adjustment besides mean changes.
97	Halvorsen et al. (2012)	US	Reading	QES	Confounding	Achievement of students in low-SES districts is compared to that of students in high-SES districts.
98	Hasselbring & Moore (1996)	US	Math	QES	Other bias	One intervention and one control school.
99	Hayward et al. (2007)	Canada	Reading	QES	Other bias, Confounding	Only one intervention and control class for the first contrast (a comparison design), the second contrast compares at risk to not at risk students.
100	Hernandez-Gutierrez (2008)	US	Reading	QES	Confounding	Five students identified for the intervention group and five students to the control group at each campus. Not reported how the control students are identified. Some pre-test imbalance and no adjustment for confounders.
101	Hock et al. (2017)	US	Reading	QES	Confounding	Match on pre-test score, grade-level placement, gender, number of hours in special education, ethnicity, race and SES. However, imbalance on gender and disability severity, the size of the pool of potential control students, and the matching of some students is done after attrition. Therefore, high risk of selection on unobserved variables.
102	Holmes & Gathercole (2014)	UK	Math, reading	QES	Other bias	Trial 1 has no standardised test in math or reading. Trial 2 match intervention students with students from previous cohorts (from the same school). High risk of bias from cohort effects.
103	Hopkins (1996)	US	Math, reading	QES	Confounding	Large and systematic pre-intervention differences on most test scores, sometimes up to almost 1 standard deviation, consistently favouring the control group.

	Study	Country	Test subject	Study design	Rated 5 on item?	Comment
104	Hotulainen et al. (2016)	Finland	Math, reading	QES	Confounding	Demographics not shown, and some imbalance on 3 of 4 pre-tests. No adjustment for confounders.
105	Hunt (1994)	US	Reading	QES	Other bias	One intervention class and one control class.
106	Hurford et al. (1994)	US	Reading	QES	Confounding	Screen students and divide into two at risk groups (and a not at-risk group) where half of each group receives training. Unclear how the intervention group is chosen. Matching is mentioned but approach not described. Pre-tests are imbalanced, although size of imbalance difficult to assess as standard deviations are not reported. No adjustment for confounders in the analysis.
107	Irby et al. (2016)	US	Reading	QES	Reporting bias	Campuses within each of seven school districts participated. Findings reported in this study were from 9 schools in one large urban school district. This district was selected in this study because of its long-standing reputation in improving education for ELLs, as well as its high level of fidelity of implementation of the larger RCT project.
108	Ito (1980)	US	Reading	QES	Confounding	No relevant confounders considered and no adjustment in the analysis.
109	Iversen & Tunmer (1993)	US	Reading	QES	Confounding	Match triples but some imbalance on pre-tests. No adjustment in the analysis.
110	Jack (2011)	US	Reading	QES	Confounding	No confounders considered, except all participants are 4th graders.
111	Jesson & Limbrick (2014)	New Zealand	Reading	QES	Confounding	Control group not comparable, consisted of not-at-risk peers or national norms.
112	Jimerson et al. (1997)	US	Math, reading	QES	Confounding	The control group (low-achieving not retained students) show large differences to the intervention group (retained students) on several important variables.
113	Jones et al. (2016)	US	Reading	QES	Confounding	Regression discontinuity design, but besides the test used to estimate an assignment rule, no other confounders considered or imbalance tests presented.

	Study	Country	Test subject	Study design	Rated 5 on item?	Comment
114	Jones-Mason (2012)	US	Math, reading	QES	Confounding	No adjustment for confounders.
115	Jordan et al. (2011)	US	Math	RCT	Reporting bias	No demographics or pre-test means or post-test means are reported, only ANCOVA p-values of post- tests using a variety of pre-tests as covariates and only for significant p-values.
116	Juel (1996)	US	Reading	QES	Confounding	Children were selected for tutoring by the principal and classroom teachers on the basis of need and availability of tutors. The remaining students were mentored, but not tutored, by a student-athlete. All 1st grade otherwise no relevant confounders considered or adjusted for.
117	Kajamies et al. (2010)	Finland	Math	QES	Confounding	Each intervention student is matched to two controls based on scores in word problem solving, arithmetical skills, and non-verbal intelligence. Despite the matching, some large imbalances persist at pre-test, which are not adjusted for in the analysis.
118	Kamberg (2010)	US	Reading	QES	Confounding	No confounders considered except grade and no imbalance tests shown.
119	Keita (2011)	US	Reading	QES	Other bias	Students from two intervention schools in one district and two control schools in another district with different characteristics. High risk of bias from district effects.
120	Kerchner & Kistinger (1984)	US	Reading	QES	Other bias	Intervention students are from one experimental site and control from two other classes in the district.
121	Klijian (2010)	US	Reading	QES	Other bias	Intervention students from one school and control from other schools.
122	Klingner et al. (2004)	US	Reading	QES	Confounding	Two intervention schools and three control schools. No information on how schools were chosen. Imbalances on school and teacher characteristics, as well as pre-tests. No student demographics reported.

	Study	Country	Test subject	Study design	Rated 5 on item?	Comment
123	Knapp & Winsor (1998)	US	Reading	QES	Confounding	Match 9 intervention students to 9 control students. Two intervention student leaves, one is omitted from the analysis along with the matched control and one is replaced by a control group student, which in turn is replaced by a new student. Grade imbalance. Students were recruited from 2 schools, the group distribution across schools is not mentioned. Use gain scores, no other confounders adjusted for.
124	Kong (2009)	US	Reading	QES	Confounding	6 intervention and 6 control classes. Some pre-test imbalance. No adjustment for confounders.
125	Lara-Alecio et al. (2012)	US	Reading	QES	Confounding	Randomize 4 schools to intervention and control, but non-randomly assign teachers to treatment, which is not controlled for. Does not show the balancing tests for the pre-tests for students, only F-values and p-values. Some imbalances on school demographics. Only one model adjusts for pre-test.
126	Laub (1997)	US	Reading	QES	Other bias	Control students are from the same school as treated but attended (and were assessed) the year before the treated. High risk of bias from cohort effects.
127	Lawson (2011)	US	Reading	QES	Confounding	Match on pre-tests but do not show or test imbalance. No further adjustment in the analysis.
128	Leafstedt et al. (2004)	US	Reading	QES	Other bias	One intact class received the intervention and the control group was selected from a longitudinal study that was conducted two years prior to the present study.
129	Leong (1995)	Canada	Reading	QES	Confounding	Only study 2 relevant. Intervention group are students retained from kindergarten. 3 comparison groups matched on gender, and 1) and 3) on starting year of kindergarten: 1) Not retained in any grade (up to 5th); 2) Not retained in any grade and started 1st grade same year as treated, 3) Retained in 1st grade. All three groups have a high risk of selection, as indicated by significant differences on pre-tests.

	Study	Country	Test subject	Study design	Rated 5 on item?	Comment
130	Linan-Thompson et al. (2005)	US	Reading	QES	Other bias	One intervention and one control school.
131	Lleras & Rangel (2009)	US	Reading	QES	Confounding	Intervention group is students placed in low ability groups by the teacher and control is students from classes not using ability grouping (teachers' choice to do or not to do). No demographics or pre-tests shown by group. No adjustment for individual pre-tests in the analysis.
132	Lloyd et al. (1980)	US	Reading	RCT	Other bias	Students randomly assigned to three intervention and one control classroom.
133	Lo et al. (2009)	US	Reading	QES	Confounding	At-risk students were selected to receive intervention because of their literacy skills. Stratified random sampling was used to select 25 control students among the remaining 35 students. Large pre-test and gender imbalance and some ethnicity imbalance. Age and SES not considered
134	Lopez & Tashakkori (2003)	US	Reading	QES	Confounding	Large pre-test differences. Adjust only for free- and reduced-price lunch status.
135	Lopez & Tashakkori (2004a)	US	Reading	QES	Confounding	Large ESOL, pre-test, and free- and reduced-price lunch status imbalances. Report raw means and MANOVAs without adjustment for pre-tests.
136	Lopez & Tashakkori (2004b)	US	Reading	QES	Confounding	Intervention students are children of interested parents who register their children in the program. Large imbalances on some confounders and no adjustment other than separation by grade.
137	Lorence et al. (2002)	US	Reading	QES	Confounding	Retained students scoring below 70 in third grade in 1994 are the intervention group and not-retained students scoring below 70 are the control group. There are imbalances on observable variables, and, given the empirical strategy, a high risk of selection on unobservables, as other factors than pre-tests determine the decision to retain students.

	Study	Country	Test subject	Study design	Rated 5 on item?	Comment
138	Lovett et al. (2017)	Canada and US	Reading	QES	Confounding	Control group were students meeting eligibility criteria but who failed to match into an instructional group or who was referred and screened after classes had started or where from schools where other participants were not available to form an intervention class. Age, grade, gender, SES, intelligence test and 7 pre-tests. Imbalance on grade, gender, SES and 6 of the pre-tests. Use ANCOVA with pre-test as covariates only. The study ran over 5 years with catching up of recruiting control participants in the last year. Intervention and control not evenly distributed by city: Intervention/Control %: Atlanta: 39/0, Boston: 28/19, Toronto: 33/81.
139	Mac Iver & Kemper (2002)	US	Reading	QES	Confounding	Schools are matched on demographics (not reported which). No confounders shown on either school or student level. No adjustment for pre-tests other than in kindergarten.
140	Macaruso & Walker (2008)	US	Reading	RCT	Incomplete outcome data	The analysis uses a subset of the intervention group who got enough sessions. This selection is done after randomization.
141	MacDonald & Figueredo (2010)	Canada	Reading	QES	Confounding	Priority entry into the program was given to those students identified as at risk at the end of junior kindergarten (4-year-olds). The comparison group was created by default (i.e., the remaining students not participating in the program). Imbalance on English as second language. No pre-test considered.
142	Maldonado (1994)	US	Math, reading	RCT	Other bias	Twenty students were randomly selected and randomly assigned into two classes. One of the classes was randomly chosen to receive integrated bilingual special education. One teacher teaches each class so high risk of bias from teacher effects.

	Study	Country	Test subject	Study design	Rated 5 on item?	Comment
143	Mantzicopoulos et al. (1992)	US	Reading	RCT	Incomplete outcome data	Students screened in Kindergarten and 437 were at-risk. At the beginning of 1st grade 'subjects who continued participation in the study' were randomised, but numbers are not reported. By the end of 2nd grade 168 students remained. Attrition rate from Kindergarten to 2nd grade is 62% and from 1st grade to 2nd grade, it is unknown.
144	Marian et al. (2013)	US	Math, reading	QES	Confounding	Do not consider age, pre-test and there is imbalance on SES. No adjustment for confounders in the analysis.
145	Marr et al. (2011)	US	Reading	QES	Confounding	Match with students in control schools that are randomly selected from a group "with similar levels of fall benchmark risk on an oral reading fluency measure" (p. 257). Control schools are not randomly assigned and the matching is unsuccessful in the sense that there is large difference in favor of the treatment group on the pre-test. Pre-tests are not adjusted for in the analysis. Some additional confounders described in text but mainly on a school level.
146	Mathes et al. (1998)	US	Reading	QES	Confounding	Of the 10 First-Grade PALS teachers, 4 piloted the procedures the preceding year and requested continued participation. The 5th pilot teacher moved from the district. Control group matches for these 4 teachers were recruited from among teachers who had similar teaching profiles. The remaining 12 teachers were recruited to participate in the project, then randomly assigned to either the First-Grade PALS or control group. Student level imbalances on disability imbalance and some pre-tests. No adjustment for confounders except through gain scores.

	Study	Country	Test subject	Study design	Rated 5 on item?	Comment
147	Mathes et al. (2001)	US	Reading	RCT	Other bias	Teachers randomised except three that continue using the intervention as in a previous year. Age, education and total years teaching experience imbalances between teachers. Teachers can influence which students are included in the analysis after randomisation. "In several classrooms, we either did not receive parental consent to assess the academic performance of their child, or the class did not have children meeting our definitions of HA, AA, or LA" (p. 382), nothing else reported on this matter, thus unclear if whole classes (and which) do not contribute to the analysis. Do not report if parent consent was sought before or after randomisation. Some imbalance for the LA group on 3 of 10 pre-tests between the PALS group and control. Imbalance between PALS/CAI and control on 7 of 10 pre-test and 4 imbalances are large.
148	McDermott & Watkins (1983)	US	Math, reading	QES	Confounding	Teachers in 2 out of 7 schools volunteer to implement the program. Consider imbalance on gender, age, race, and IQ. Mention that there are differences in WRAT scores but do not show any pre-tests. Adjust for IQ and pre-test scores in an ANCOVA, but not age, gender, race, or other characteristics. Insufficient information to use the ANCOVA results in the calculation of effect sizes.
149	McIntyre et al. (2005)	US	Reading	QES	Confounding	Teachers were asked to identify the lowest achieving 20% of students in their classes (with consent to participate). Less than half of the children that were tested received supplemental instruction according to the author’s definition. These students constitute the comparison group. High risk of bias from selection.
150	McLean (2015)	US	Math	QES	Other bias	One intervention classroom and one control classroom.
151	McMasters (2012)	US	Reading	QES	Other bias	Comparison between three cohorts who received three different interventions. High risk of bias from cohort effects.

	Study	Country	Test subject	Study design	Rated 5 on item?	Comment
152	Medina et al. (1985)	US	Math, reading	QES	Other bias	Intervention group from one school, control group from four other schools.
153	Menar (2002)	US	Reading	QES	Confounding	The intervention and control group are matched, but unclear on what variables. Imbalance on "academic functioning". Examine change scores, otherwise no adjustment for confounders.
154	Mevarech & Rich (1985)	Israel	Math	QES	Confounding	Report that ethnicity and gender of students were balanced and that teachers were all fully certificated and had at least 5 years of experience. Pre-tests and other demographics not considered or adjusted for.
155	Meyer (1986)	US	Math	RCT	Other bias	Unclear how many "class periods" that are randomised. Class periods average 8 students, so possibly two class periods in each of the treatment groups and 3-4 in the control group. The grade distribution is not reported, students are in grades 1-5. Large pre-test imbalance between one intervention group and the control group. No other demographics are mentioned.
156	Mitchell (2010)	US	Reading	QES	Other bias	One intervention and one control school.
157	Molina et al. (1997)	Spain	Reading	RCT	Reporting bias	No data shown other than difference between groups in gains.
158	Mononen & Aunio (2014)	Finland	Math	QES	Confounding	Same grade, gender balanced, large pre-test imbalances, otherwise nothing considered and no adjustment for confounders in the analysis.
159	Moore (2015)	US	Math	QES	Other bias	One intervention and one control school.

	Study	Country	Test subject	Study design	Rated 5 on item?	Comment
160	Moran et al. (2014)	US	Math	RCT	Other bias	Two students in year one and two students in year two were moved from control to intervention (13 in control and 59 in intervention group, after the movements). Participants were identified from four school sites that were part of two California school districts. All 4 groups (1 control and 3 interventions) had participants from at least 3 school sites but it is a two year study, 27 students participated in year one and 45 in year two. Cannot rule out that students in for example the control condition are from only one school (or class) for one of the years. Some pre-test imbalances.
161	Morgan et al. (2008)	US	Reading	RCT	Other bias	Selection of students probably done after randomisation of classes. Randomisation of classes part of a larger project but no information about this project. There is twice as many control students as treated but no information on number of classes, only that there is a total of 30 classroom teachers recruited for the project. Six children that scored extremely high or low are treated as outliers, and their test scores are coded as one unit smaller or larger than the next highest or lowest score. There is no information about the sensitivity to this choice. Two students (of unclear group) are deleted from further analysis as they did not reach basal levels on one pre-test. Systematic and large pre-test, ethnicity, retained and IEP imbalance.

	Study	Country	Test subject	Study design	Rated 5 on item?	Comment
162	Morocco et al. (1989)	US	Reading	QES	Confounding	In Year 1, four treatment classroom teachers were selected within three schools to participate in the project. Participating classroom and specialist teachers were recommended by their school principals and, in some cases, their district language arts supervisors for participation in the study. Intervention teachers were identified as excellent teachers by their peers and administrators. In Year 2, the intervention teachers continued to participate, and control group teachers are selected in each sit (i.e. district, not the same schools except in one district. High risk of bias from teacher effects.
163	Morta (2010)	US	Math, reading	QES	Other bias	One intervention and one control class.
164	Moser et al. (2012)	US	Math, reading	QES	Confounding	Use propensity score matching to match retained (intervention) and not retained (control) students. Balance is reported on a subset of variables. It is stated that "The effect sizes were small, never exceeding Cohen's d = 0.30 standard deviation difference." (p. 9). However, 0.3 is reasonably large, in comparison to expected effect sizes. There is no explanation of why some students are retained and other promoted, i.e., there is likely some unobserved difference that make schools retain students or not, especially since students seem to be matched within schools. The promoted students propensity scores are right skewed whereas the retained students propensity scores are uniform (figure 1A). Do not report statistical diagnostics for the propensity score estimation, only a figure of the scores and F-values from ANOVA of Main effect of retention of 20 of the 72 matching variables.

	Study	Country	Test subject	Study design	Rated 5 on item?	Comment
165	Murphy (2004)	US	Reading	QES	Confounding	The intervention group is successfully discontinued Reading Recovery students, i.e., a selected group. Furthermore, the control group did not score as low on the Observation survey, or were not recommended to Reading Recovery by teachers, i.e., they likely have less problems than the intervention group to start with. No information about pre-test balance.
166	Myers (2017)	US	Reading	QES	Confounding	Imbalance on SES. Pre-tests not shown. Outliers removed in the analyses that adjust for confounders.
167	Nave (2007)	US	Reading	QES	Confounding	Compare Title 1 to not Title 1 schools. Unclear number of schools in each group and unclear why some schools implement the program and some do not. Separate analyses by gender and by SES and analyse gain scores. Some imbalance on pre-tests. No other confounders considered or adjusted for.
168	Nidich (2011)	US	Math, reading	QES	Confounding	Use 8th grade students as control group, but they do not seem to take the same test. I.e. eight graders take their test in 8th grade, 7th in 7th and 6th in 6th. Consider only pre-tests.
169	O'Connor et al. (2005)	US	Reading	QES	Other bias	Compares an intervention group in kindergarten with two earlier cohorts from the same two schools. The two earlier cohorts are in year 2 and 3 the year before the study starts and the comparison is made when the intervention group reach end of year 2 and 3, i.e., 3-4 years after. High risk of bias from cohort effects.
170	O'Connor et al. (2014)	US	Reading	QES	Other bias	Across five schools, students who began having access to Tier 2 intervention in kindergarten or first grade were compared in Grades 1 and 2 with cohort peers who were average readers and 102 control students from an earlier cohort who did not receive Tier 2 intervention. First group is not comparable and second has high risk of bias from cohort effects.

	Study	Country	Test subject	Study design	Rated 5 on item?	Comment
171	O'Connor et al. (1996)	US	Reading	RCT	Other bias	1 intervention class and 1 control class in the one subgroup that is relevant for this review (transition kindergartens).
172	O'Connor et al. (1998)	US	Reading	RCT	Other bias	Follow-up to O'Connor et al. (1998). 1 intervention class and 1 control class in the one subgroup that is relevant for this review (transition kindergartens).
173	O'Melia & Rosenberg (1994)	US	Math	RCT	Other bias	Teachers teach both an intervention class and a control class and the analysed students were recommended to participate in the study by the teachers (the timing is not reported). No check for spillovers, i.e., the control group teacher practice is not examined. Too high risk because students are probably chosen to be included after randomisation and there are large pre-intervention imbalances. Not sufficient information to use the ANCOVA for the effect size calculation.
174	Osborn et al. (2007)	US	Reading	QES	Confounding	Match schools. Imbalance on pre-test scores not shown, only age is mentioned in text. Post-test scores only shown adjusted for pre-test.
175	Phillips (1990)	US	Reading	QES	Confounding	Compare retained to not retained students. Imbalances between groups on either pre-tests or characteristics or both. Unclear why some schools do not offer the developmental program.

	Study	Country	Test subject	Study design	Rated 5 on item?	Comment
176	Pinnell et al. (1994)	US	Reading	RCT	Other bias	Within-school randomisation to intervention and control group. Schools perform the randomisation of students. Two districts (our of 10) divided intervention and control groupsmparison groups, without checking this decision with the researchers. These two districts were among the four whose pretest scores for treatment and comparison group were aberrant, and they were dropped from the analysis. Although explicit directions were given to all schools on how to execute the random assignment of students to the treatment and comparison conditions, four schools apparently chose to ignore the directions and assign their neediest students to the tutorial programs. In two other sites, valid tests were not obtained, and these too were dropped. Another school site had to be dropped because the pretest data was lost in the mail. Pre-test shown with some imbalances but only reported separately for the 4 treatment conditions and one overall control group.
177	Piro & Ortiz (2009)	US	Reading	QES	Other bias	One intervention and one control school.
178	Plony (2003)	US	Reading	QES	Confounding	Compare students who use READ180 with those that do not. No information about selection into treatment, except that the assignment seems purposeful. There are relatively large imbalances over pre-determined characteristics. These imbalances are not shown by grade, but the analysis is done by grade. Also fairly large imbalances on pre-test scores (shown by grade), consistently favoring the control group. Age and SES not considered.
179	Porter (2010)	US	Reading	QES	Confounding	Pre-test adjusted for but imbalances are not shown or discussed. No other confounders considered.
180	Rabiner et al. (2010)	US	Math, reading	RCT	Reporting bias	Only report the percent who had improved at least half a standard deviation. No means by group are reported. Baseline and two follow-up means are reported for the overall sample, not by condition.

	Study	Country	Test subject	Study design	Rated 5 on item?	Comment
181	Rafdal et al. (2011)	US	Reading	QES	Confounding	Classroom are chosen non-randomly from a larger group of randomised classrooms. Reanalyses data on children with learning disabilities from a larger study. The participants are chosen non-randomly within classes using the same criteria for intervention and control group. Small number of clusters in total, and only 9 teachers in the control group. There are some large imbalances across teacher and student characteristics. Not sufficient information to use the results from the analysis where pre-test are adjusted for to calculate effect sizes.
182	Rapp (1991)	US	Reading	QES	Other bias	One intervention and one control school.
183	Rasinski & Oswald (2005)	US	Reading	QES	Other bias	Both studies: one intervention and one control class.
184	Redmon (2007)	US	Math, reading	QES	Other bias	Two intervention and one control school.
185	Reyes-Bonilla & Carrasquillo (1993)	Puerto Rico/US	Reading	QES	Other bias	One intervention and one control class.
186	Rhett (2011)	US	Reading	QES	Confounding	Large pre-test imbalances, no other confounders considered and no adjustment for confounders.
187	Ross & Jeffery (1991)	US	Math	RCT	Other bias	Nine intact classes from 4 schools were randomly assigned to 3 groups. No other confounders reported other than SES. It is not reported which classes from which schools are in any of the 3 groups, may be that one group consists of classes from only one school in which case it is impossible to distinguish between the intervention and school effect.
188	Ross & Smith (1994)	US	Reading	QES	Other bias	One intervention and one control school.
189	Ross et al. (1997)	US	Reading	QES	Confounding	Two intervention schools are matched with two control schools. Demographics for each school are reported but not separately for those students who participate/are analysed. No pre-tests are shown but used in a MANCOVA, which we cannot use to calculate effect sizes.

	Study	Country	Test subject	Study design	Rated 5 on item?	Comment
190	Ross et al. (1999)	US	Reading	QES	Confounding	Match two intervention schools with two control schools, probably on school size, ethnicity and lunch status as they are showed. Some imbalances. No pre-program student demographics are shown. Adjust for ethnicity in analyses. Analyses a low-achieving sub sample but unclear which year the pre-test are taken from.
191	Rothenberg (1990)	US	Math, reading	QES	Other bias	Intervention sample drawn from two schools and the control was randomly selected among the remainder of the at risk pool. Not reported from how many schools, and if they are the same or other schools than the intervention schools. No demographics or pre-test reported.
192	Saint-Laurent (1996)	Canada	Math, reading	QES	Confounding	Unclear assignment procedure, no explanation of why some schools are assigned to treatment and others not. Large imbalance on one pre-test measure. Balance not shown on other student characteristics. No adjustment for confounders.
193	Saracho (1982)	US	Math, reading	QES	Confounding	Placement in intervention and control group depend on "terminal" availability in schools, but no mention of how many schools there are. Some pre-test imbalances but does not seem to systematically favour treatment or control groups. Adjust for pre-tests but report no adjusted mean and not sufficient information to use the F-value from the ANCOVA. Raw means have too high risk of bias.
194	Sauve (2009)	Canada	Math, reading	QES	Confounding	Match students with reading disabilities with students without reading disabilities.
195	Scalf (2014)	US	Math, reading	QES	Confounding	The placement decision, pullout or inclusion, was based on the severity of the gap between the IQ and achievement scores and the student’s unique needs. Intervention and comparison group are by design not the same. No balancing test presented.

	Study	Country	Test subject	Study design	Rated 5 on item?	Comment
196	Scientific Learning Corporation (2004a)	US	Reading	RCT	Reporting bias	Means not shown for either pre or post tests, only figures and results given as F-values of improvement. Only 2 of 4 tests are shown, it is mentioned that one test did not reach significance and the last test is not mentioned.
197	Scientific Learning Corporation (2004b)	US	Reading	QES	Confounding	Grade is the same and pre-test is shown, otherwise no relevant confounders considered and no adjustment for confounders.
198	Scientific Learning Corporation (2004c)	US	Reading	QES	Confounding	Grade and pre-test means shown, large imbalance especially for the lower grades. No adjustment for confounders in the analysis.
199	Scientific Learning Corporation (2007)	US	Reading	QES	Confounding	The intervention group is matched by schools to a control group on grade and pre-tests. No demographics considered and pre-tests are only shown in figures, which indicate large imbalances.
200	Scott (1999)	US	Reading	QES	Other bias	Two classes from one school formed the intervention group and one class each from two other schools form the control group.
201	Scruggs & Osguthorpe (1986)	US	Reading	QES	Confounding	Both experiments use a control group taken from the same settings, same schools, and same teachers as the experimental students, with the only difference being either scheduling or matching difficulties preventing them from easily being integrated into the tutoring program. However, no balancing test is shown, so not possible to know whether they are in fact similar.
202	Shamey (2009)	US	Reading	QES	Confounding	Compares at-risk and “not-necessarily-at-risk” students, which is not comparable to our other included effect sizes.

	Study	Country	Test subject	Study design	Rated 5 on item?	Comment
203	Shepard & Smith (1987)	US	Math, reading	QES	Confounding	Four schools with high retention rates were matched with schools having low retention rates and then match retained and not retained students by gender, age, SES and initial academic achievement. High risk of selection on unobservable factors, as well as some problems with the pre-test matching and attrition. No adjustment for confounders in the analysis.
204	Shields et al. (2016)	US	Math, reading	QES	Confounding	Compare schools in Boston to schools outside of Boston. Show gender, ethnicity, rate having individualized education plan and eligibility for free lunch, large imbalances except gender. No pre-test available.
205	Shields (1995)	US	Math, reading	QES	Confounding	No demographic characteristics other than grade considered. There are pre-tests but balance is not shown.
206	Sigears (2009)	US	Reading	QES	Other bias	One intervention and one control school.
207	Silvious (2008)	US	Math	RCT	Other bias	School level randomisation. Cannot find any information about/comparisons of demographics (gender, SES, ethnicity) between control and intervention group. Imbalances in grade level (table 2). Divide analysis by elementary/middle school and pre-tests/post-tests but show only the treatment group. Gender and time effects considered for intervention group. Pre-test imbalances.
208	Simmons & Fuchs (1995)	US	Reading	QES	Confounding	Control teachers are some (5) who did not want to participate and some (3) from another school. Control teachers are less experienced and their students' reading level (as estimated by the teachers themselves) is lower. No adjustment for confounders.
209	Soriano et al. (2011)	Spain	Reading	QES	Confounding	Mean age difference between intervention and control group is almost 2 years. Use age as a covariate in an MANCOVA and analyse gain scores. We cannot use the MANCOVA results to calculate effect sizes.

	Study	Country	Test subject	Study design	Rated 5 on item?	Comment
210	Southard & Deborah (1996)	US	Math, reading	QES	Confounding	Intervention students are retained from kindergarten. Matching but not on kindergarten student achievement as too many subjects would have been lost. Construct 3 comparison groups are constructed (and matched on gender): 1) Those not retained in any grade (up to 5th) and starting kindergarten same year as the intervention group, 2) Those not retained in any grade and started 1st grade the same year as the intervention group, and 3) Those starting kindergarten same year as the intervention group and were retained in 1st grade. High risk of selection on both observable and unobservable variables for all three groups.
211	Spaulding (2007)	US	Reading	QES	Confounding	Large pre-test differences. No other confounder considered except grade.
212	Spencer et al. (1989)	Canada	Reading	QES	Confounding	Unclear assignment procedure. Some imbalance at pre-test and gender, grade and SES not considered. No adjustment for confounders in the analysis.
213	Spillios & Janzen (1983)	Canada	Reading	RCT	Other bias	Research design changed from four to two groups after original randomisation. Unclear whether or not students in the two omitted groups are re-allocated to the two remaining groups or not. Exclude a non-pre-tested control group that has significantly better post-intervention results than the non-pre-tested intervention group from the analysis.
214	Steinberg (1991)	US	Reading	QES	Other bias	The two schools the researcher were assigned as a Chapter 1 specialist teacher were treatment schools and two other matched schools were control. The analysis conducted as a comparison of treatment/control schools in pairs, e.g. T1 school vs C1 school and T2 school vs C2 school. I.e., one intervention and one control school in each analysis.
215	Stephens (2008)	US	Reading	QES	Other bias	One intervention school.
216	Stevens et al. (2008)	US	Reading	QES	Other bias	Two intervention and one control school.

	Study	Country	Test subject	Study design	Rated 5 on item?	Comment
217	Swanson (2015)	US	Math	RCT	Reporting bias	No descriptive statistics (on groups) and no raw post test means reported (only adjusted pre-tests, gains and post-test z-scores based on the mean and standard deviations at pre-test) and further divided on at-risk/not at-risk and high/low working memory.
218	Swanson et al. (2015)	US	Math	RCT	Other bias	The unequal sample sizes reflect removing children with low reading or fluid intelligence scores from the data analysis T1: 4 of 28 (14%); T2: 7 of 25 (28%); T3: 1 of 20 (5%) and C: 9 of 27 (33%). Wave 1 (pre-intervention) scores are imbalanced but less so after removal of children with low reading or fluid intelligence (table 2). No other demographic considered.
219	Swanson et al. (2013a)	US	Math	RCT	Other bias	Remove students from the analysis after randomisation. Do not report means of pre-tests.
220	Swanson et al. (2013b)	US	Math	RCT	Reporting bias	No descriptives and no raw post-test means reported (only pre-test adjusted). Within classroom assignment to control and 3 interventions. Not reported what intervention is randomised to (or how it is chosen) in classrooms with less than 4 students or a number not a multiple of 4 (if there are such classrooms, which is not reported).
221	Tong et al. (2014)	US	Reading	QES	Confounding	Randomize 4 schools to intervention and control, but non-randomly assign teachers to the intervention, which is not controlled for in any way. Does not provide any balancing test for pre-tests and do not adjust for pre-tests in the analysis. Some imbalances on student demographics.
222	Tracey & Young (2007)	US	Reading	QES	Confounding	Unclear assignment procedure and no information on why the intervention group get the intervention and the control group does not. The balance tests are not shown (means and SDs), only the F-test from one test with a significant difference is reported in text. Analysis is a t-test of differences in gains.

	Study	Country	Test subject	Study design	Rated 5 on item?	Comment
223	Trautman & Howe (2005)	US	Math	QES	Confounding	Students from a school with an intervention are compared to national normative data.
224	Treat (2013)	US	Reading	QES	Confounding	There are two Resource Specialist Program (RSP) teachers at the school, one is the researcher. Students who received the intervention were those who were available to the researcher for participation in the intervention. Students who were not available for participation in the intervention due to general education and special education class schedules were placed in the control group. In the Resource Specialist Program small-group pull-out sessions, both comparison and intervention students received literacy instruction that, with the exception of the animal-assisted intervention, included the same components. Unclear if the second RSP teacher is involved with the control students (and/or intervention students).
225	Trexler (2009)	US	Math	QES	Confounding	Consider gender, ethnicity, free and reduced price lunch, and grade. Large imbalance on grade. Ethnicity, gender and free lunch shown overall and not shown by condition. Pre-test not shown or mentioned by condition. Use gain scores and otherwise no adjustment for confounders.
226	Trifiletti et al. (1984)	US	Math	RCT	Other bias	Unclear what the control group gets and whether that was part of the regular instruction. One intervention and one control class.

	Study	Country	Test subject	Study design	Rated 5 on item?	Comment
227	Troia (2004)	US	Reading	QES	Confounding	Partly randomised (4 schools) and partly matched (3 schools) on grade, IQ, and English language proficiency. Do not report how intervention students are chosen (or who chose) and do not report method of matching. States that 99 students were in the intervention group (out of a total of 269 students who participated in the field trial), and 92 were in the control group. Unclear what happened to the remaining of the 269 students who participated. No adjustment for confounders in the analysis.
228	Tucker & Jones (2010)	US	Reading	RCT	Other bias	Unclear assignment procedure, but probably randomised. Pre-test taken after randomisation. No balance tests presented for pre-tests. Unclear what some of the *t*-tests are testing. Based on student names there seem to be gender imbalance and perhaps also ethnicity imbalance.
229	Turlo (1990)	US	Reading	QES	Confounding	Unclear assignment procedure, some randomisation but assignment to control group seem be non-random. Sixteen control students are from the same classroom, four are from three other classrooms. Grade and gender balanced, unclear if there are pre-test imbalance. SES not considered. No adjustment for confounders.
230	Uzomah (2012)	US	Math	QES	Confounding	Nine Kindergarten classes at the school, 3 use the intervention, 6 does not and only 3 of these are chosen as control, method of selection not reported. Age and gender imbalances not considered. No adjustment for confounders.

	Study	Country	Test subject	Study design	Rated 5 on item?	Comment
231	Vadasy et al. (2002)	US	Reading	QES	Confounding	Unclear assignment procedures. Schools are selected as intervention or control schools (although 2 schools are both T and C). Participating students were selected by their classroom teachers as at risk for reading disability or reading failure and then screened and pretested by project staff. Imbalance on some of the pre-tests and age is not considered. No adjustment for confounders in the analysis, although effect sizes that become significant when pre-tests are adjusted for are mentioned in the text
232	Vadasy et al. (2005)	US	Reading	QES	Confounding	Match triads. Pre-test scores are reasonably well balanced, while some characteristics have larger imbalances (ethnicity, ELLs). Post-intervention selection of students. Of the 78 students completing all phases of the study, 57 are included in the analyses (original groups included 26, 19, and 33 for Reading Practice, Word Study, and controls, respectively).
233	Valenzuela de la Garza & Marcello (1985)	US	Math, reading	QES	Confounding	Intervention and control students differed in their initial language dominance and no pre-tests are presented. No adjustment for confounders.
234	Van der Jagt (1999)	US	Reading	RCT	Other bias	Three schools randomised to one of three conditions.
235	Van Voorhis (2011)	US	Math	RCT	Other bias	One teacher at each of four schools is randomly assigned to control or intervention in year one. Randomisation is compromised in year two as students disperse across teachers. There are imbalances on ethnicity, SES, and the Student and Family Attitude and Emotion Scales. Only 26 of the original (year one) 66 remained in the TIP1 class and became TIP2 students. Not reported if the TIP1 students received treatment in year one or year two. Results of mathematical achievement not shown for year 1, only for year 2.

	Study	Country	Test subject	Study design	Rated 5 on item?	Comment
236	Vellutino et al. (2006)	US	Reading	RCT	Incomplete outcome data	Several participating schools leave the study. Unclear how many though. Table 1 indicates 475 students were randomised and Table 2 report there are 53 intervention students and 68 non-intervention students, thus using only 25% of those randomised due to the removal of students from schools omitted from the analysis. Due to missing data, the number of students are further reduced to 48 treated and 65 control.
237	Vernon-Feagans (2010)	US	Reading	RCT	Other bias	4 schools were matched in pairs and randomly assigned. After assignment one intervention school withdraw, leaving only one intervention school.
238	Vollands et al. (1996)	UK	Reading	QES	Other bias	Each intervention and control group corresponds to one class. High risk of bias from class effects.
239	Walker et al. (2009)	US	Reading	RCT	Other bias	Teachers randomly assigned to intervention or control. Then they screen their students and the student with the highest score on a universal problem behavior screener is selected to participate. After selection of students, parental consent is asked for and there are more students in the control condition whose parents decline.
240	Warfel (2000)	US	Math, reading	QES	Confounding	Large gender and age imbalances. Pre-tests not shown. Adjust for gender, age on entrance to school, and SES, but not pre-tests.
241	Wehbe (2012)	US	Reading	QES	Confounding	No information about why some students are in the half-day and some in the full-day. High risk of selection into one or the other program. No adjustment for confounders.
242	Weiss (1992)	US	Reading	QES	Other bias	One intervention and one control class.
243	Weller et al. (1998)	US	Math, reading	QES	Other bias	One intervention and one control school.

	Study	Country	Test subject	Study design	Rated 5 on item?	Comment
244	Westbury (1994)	Canada	Math, reading	QES	Confounding	Compare retained and non-retained matched on current grade (6), gender and grade 1 achievement data. No information on when the retained were retained except 85% of them were retained in grade 1,2 or 3. No adjustment for confounders in the analysis.
245	White et al. (2005)	US	Reading	QES	Confounding	Compares intervention to all non-intervention students in grades 4-8 within the same schools in one district. 16 schools and 12% of students are in the intervention group, differs widely on grade. Large pre-test imbalances and no adjustment for confounders.
246	Whyte (1993)	UK	Reading	QES	Confounding	Three cohorts of boys in three consecutive years enrolled in same school at same age and with same reception class teacher. The last cohort experienced a language stimulation programme (these are the intervention studens, the other two cohorts are control). Consider only age and gender.
247	Wilczynski (2006)	US	Reading	QES	Confounding	Compares low-achieving and high-achieving students, without adjusting for confounders.
248	Williams et al. (2007)	US	Reading	RCT	Reporting bias	Cannot find post-intervention results from the standardised test, although they are mentioned in text.
249	Williams (1998)	US	Math, reading	QES	Other bias	One control school.
250	Williams (2012)	US	Math, reading	QES	Other bias	One control school.
251	Woodward & Baxter (1997)	US	Math	QES	Other bias	One control school.
252	Woodward & Brown (2006)	US	Math	QES	Other bias	One intervention and one control school.
253	Wright & Barrie (2003)	UK	Reading	QES	Confounding	Large pre-test imbalances on 3 of 4 tests as well as age imbalance. Gender is resonably balanced. No other confounders considered. Adjust means for age only.
254	Young et al. (2016)	US	Reading	QES	Other bias	3 classes in total. 2 different intervention conditions in two different classes, and one control class.

	Study	Country	Test subject	Study design	Rated 5 on item?	Comment
255	Ysseldyke et al. (2004)	US	Math	QES	Confounding	Compare Title I students that have teachers that use and do not use, the program. No information about why some use the program and others do not. No information about imbalance.
256	Zentall & Jiyeon (2012)	US	Reading	RCT	Reporting bias	Post-test scores not reported, only figures (no table with numbers, but bars in a figure).
257	Zeuschner (2005)	US	Reading	QES	Confounding	There were 72 participants from grades 3 to 7. The participants were divided into matched pairs which resulted in two groups, intervention and control. Students were matched on their grade, WRAT-R reading (decoding) pre-test scores, and their Fluid Reasoning scores on the Woodcock-Johnson-R or III. No mention of why some students get the program and others do not. Some tests seems to be measured at "intake" (p. 39), which could potentially be very long before the intervention. The pre-test scores on WRAT-R reading (decoding) and Fluid Reasoning scores are not reported anywhere, imbalance is thus not tested. Gender and SES are not considered. Use change scores, otherwise no adjustment for confounders in the analysis.
