# Supplementary material for: Targeted school‐based interventions for improving reading and mathematics for students with or at risk of academic difficulties in Grades K‐6: A systematic review
Source: Campbell Syst Rev. 2021 Apr 6;17(2):e1152. doi: 10.1002/cl2.1152 (PMC8356298; doi:10.1002/cl2.1152)
Supplement: Supplementary file 10 — Supporting information [file CL2-17-e1152-s012.pdf]

Online Appendix J: Forest plots by intervention component

This section presents forest plots corresponding to the analysis described in the *Subgroup analyses and investigation of heterogeneity*-section, subsection *Math and reading tests, and combined intervention components* (see in particular Figure 6-9 and Table 3). In contrast to the analyses presented in the main text, we used effect sizes that were averaged by study (or cluster) instead of all effect sizes. We again estimated random-effects models but instead of the RVE procedure in the R package *robumeta*, we used the restricted maximum likelihood option with the Knapp and Hartung adjustment in the R package *metafor* (Viechtbauer, 2010). Using study level effect sizes and this procedure provided a robustness check on the methods used in the main text. It also made the forest plots more legible. The number of effect sizes in some of the categories of intervention components otherwise made these figures difficult to read. That being said, it may still be difficult to make out the authors and effect sizes in some cases. Please see Online Appendix H for more information about each effect size and study.

As in the main text, many interventions used more than one component (i.e., included more than one instructional method, or targeted more than one content domain). Thus, the overall effect sizes in the forest plots should be interpreted as the weighted average effect size for interventions that included a certain component, not the effect size of that component in isolation. If two or more components are included in the intervention, we cannot separately identify the association with effect sizes of any component in this analysis. Below, we comment first on the general patterns and mention the few differences to the primary analysis. Then we present the forest plots for each component (instructional method and content domain) along with further information about heterogeneity.

The estimated weighted average effect sizes are, for all components, very close to the estimates presented in Table 3 in the main text. The heterogeneity was in general reduced in this analysis compared with the primary analysis. With the exception of studies targeting social-emotional content and the *tau*-squared for medium-group instruction, all heterogeneity statistics were larger in the primary analysis than in the analysis shown below. This pattern seems reasonable as averaging over effect sizes ought to for example reduce the influence of outliers and remove within-study heterogeneity. However, just as in the primary analysis, we often found evidence of substantial heterogeneity. The *Q*-test is statistically significant for all components except CAI, other methods, progress monitoring, algebra/pre-algebra, geometry, and operations. The within-component heterogeneity of the effect sizes is underscored by the prediction intervals, which in all cases but for CAI and geometry, included or was very close to zero. The prediction interval is shown at the bottom of each figure by the "whiskers" on the diamond indicating the average effect size and 95% confidence interval (see also Appendix L for prediction intervals based on the RVE procedure).

Figure A1. Forest plot of studies of interventions including computer-assisted instruction.

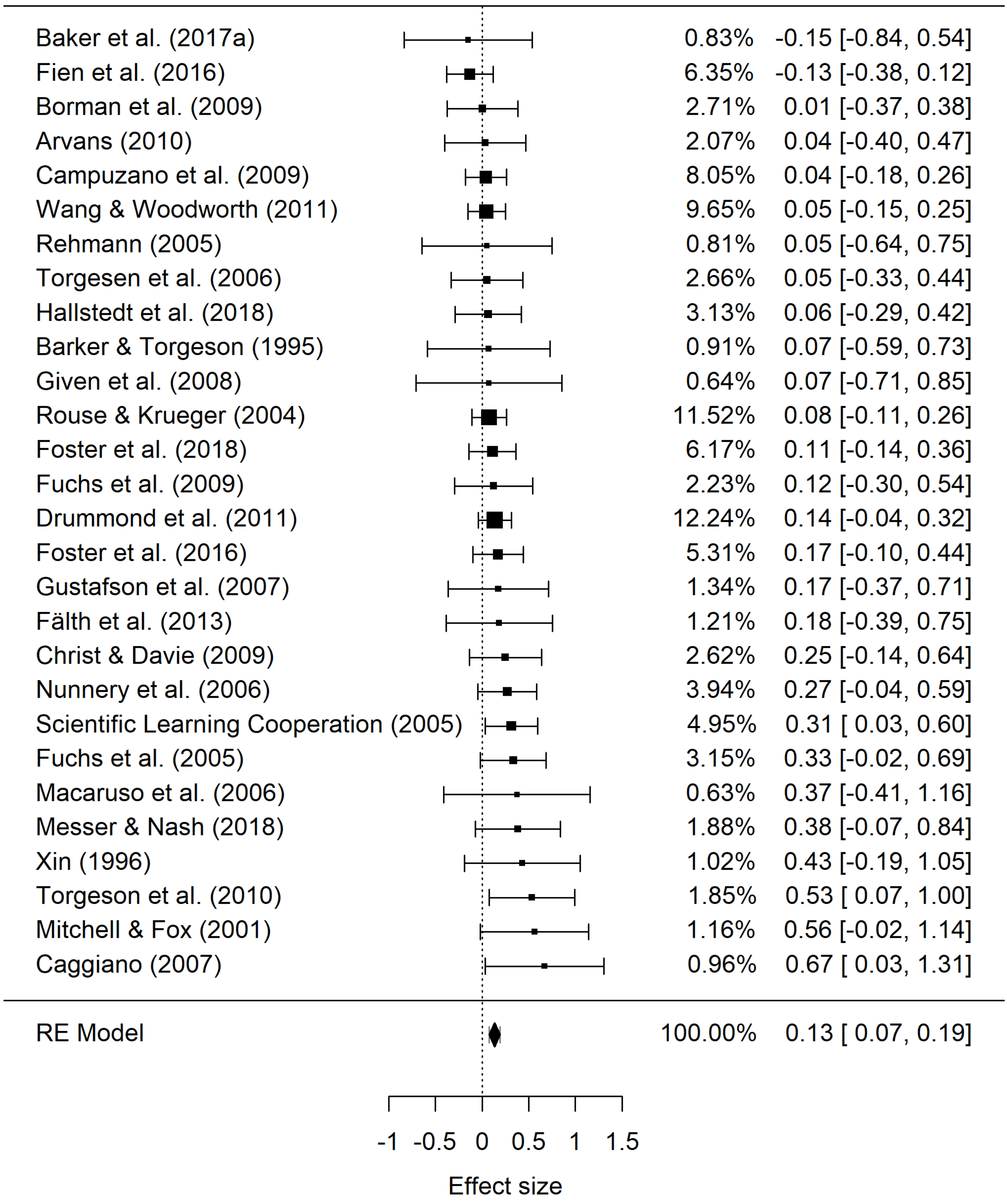

Heterogeneity: τ-squared = 0, I-squared = 0%, $Q(df = 27) = 22.0$, $p = 0.735$.

Figure A2. Forest plot of studies of interventions including coaching of personnel.

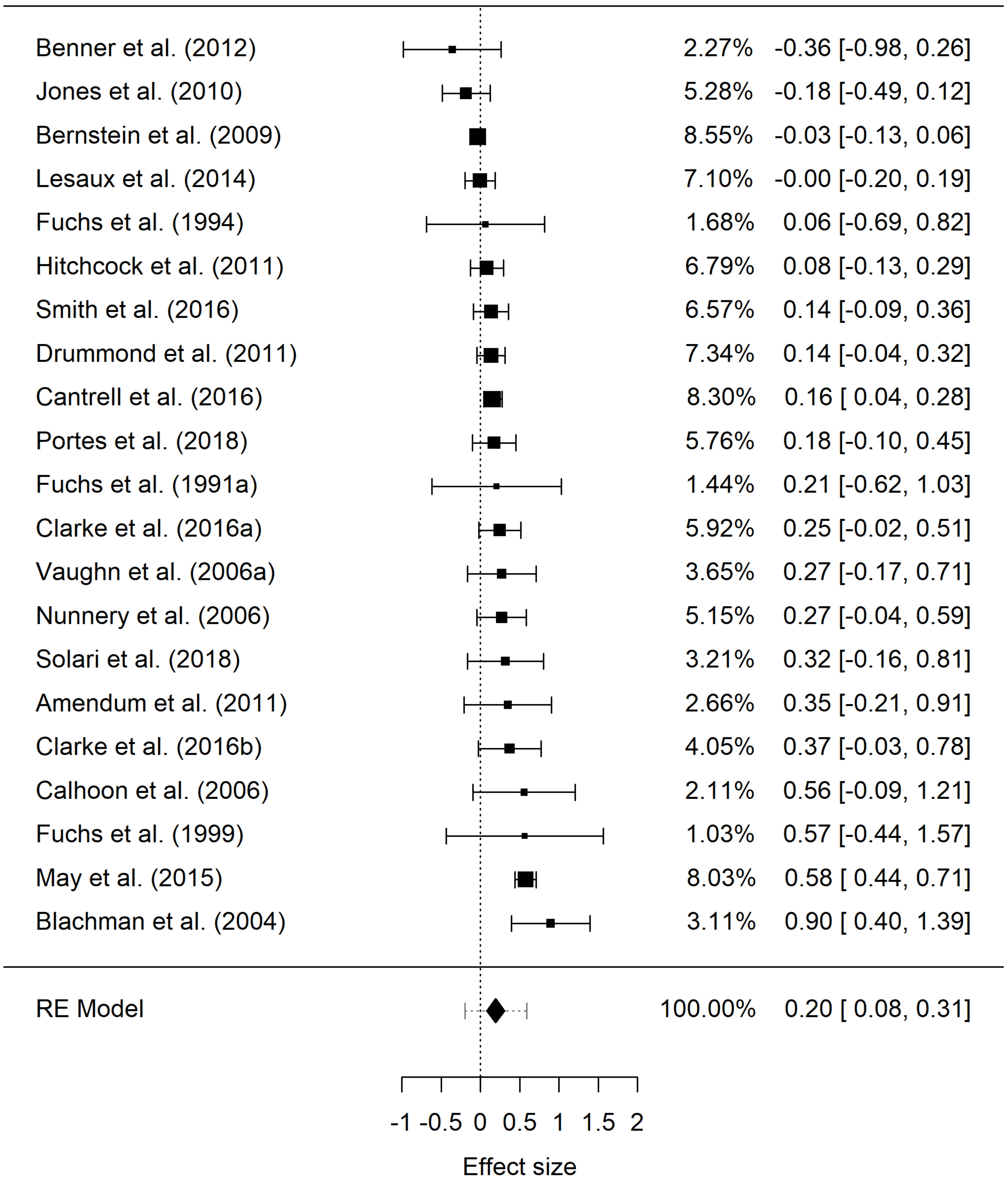

Heterogeneity: τ-squared = 0.033, I-squared = 69.3%, $Q(df = 20) = 75.5$, $p < 0.0001$.

Figure A3. Forest plot of studies of interventions including incentives.

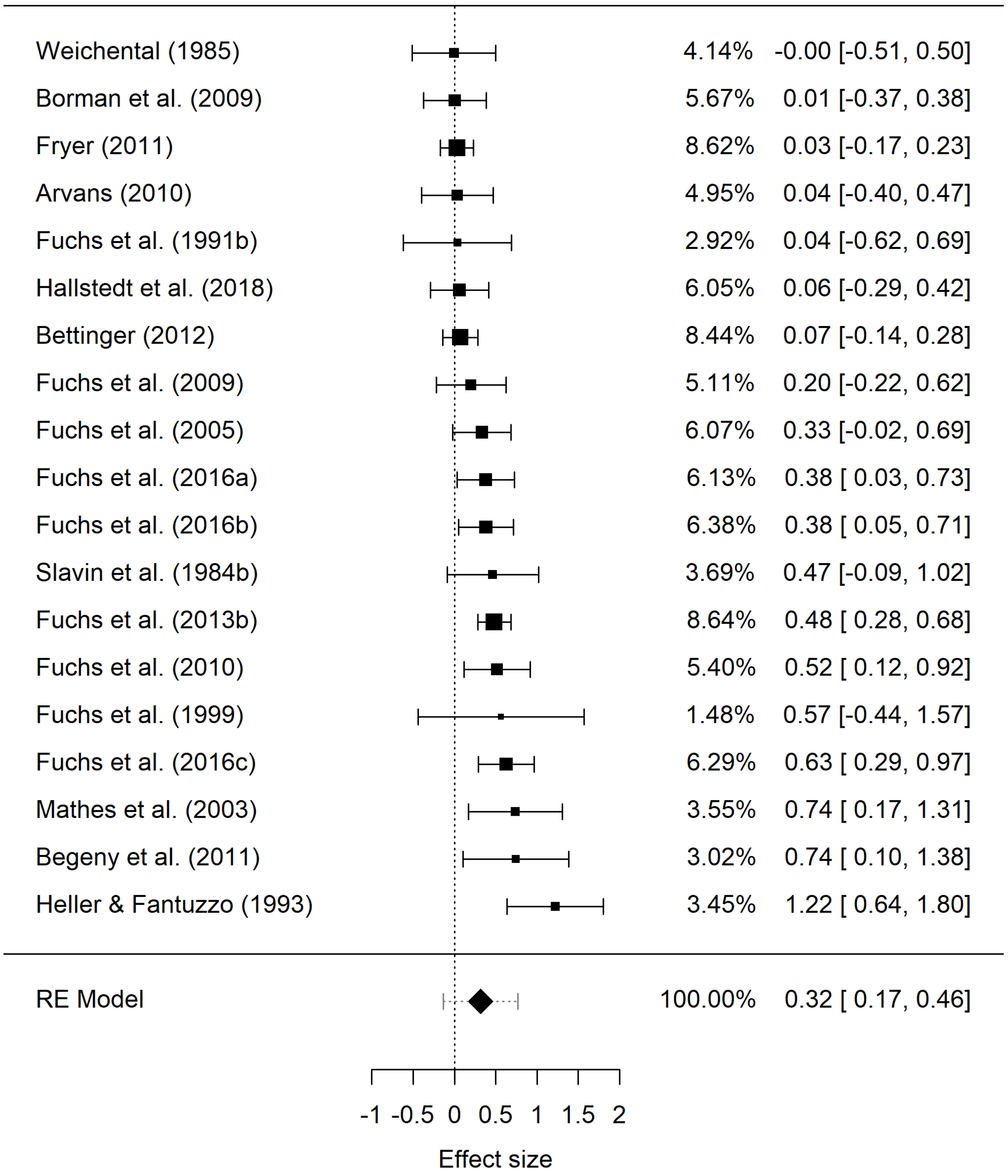

Heterogeneity: τ-squared = 0.041, I-squared = 55.8%, $Q(df = 18) = 41.5$, $p =$ 0.0013.

Figure A4. Forest plot of studies of interventions including medium-group instruction.

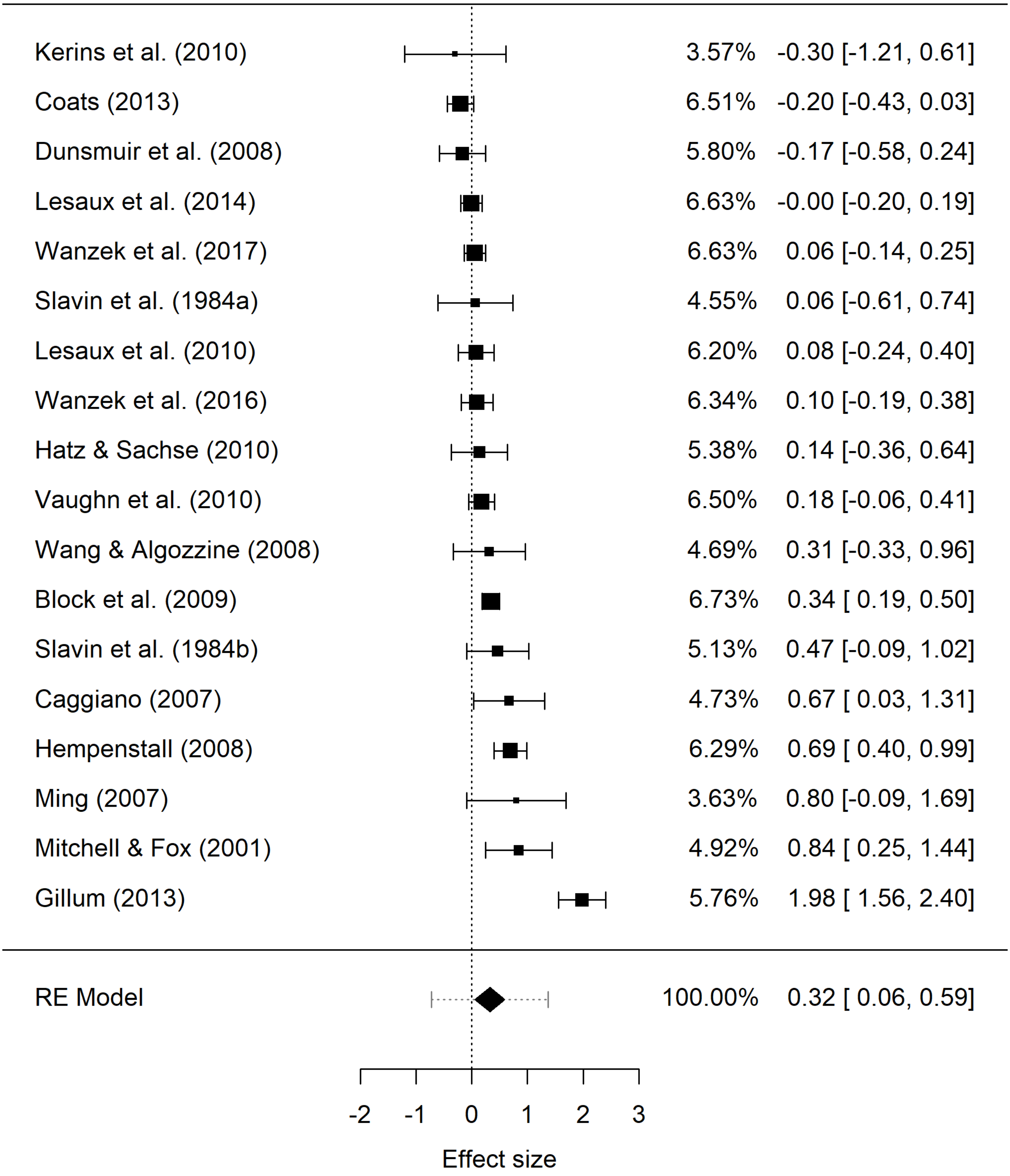

Heterogeneity: τ-squared = 0.230, I-squared = 90.0%, $Q(df = 17) = 115.6$, $p < 0.0001$.

Figure A5. Forest plot of studies of interventions including other methods.

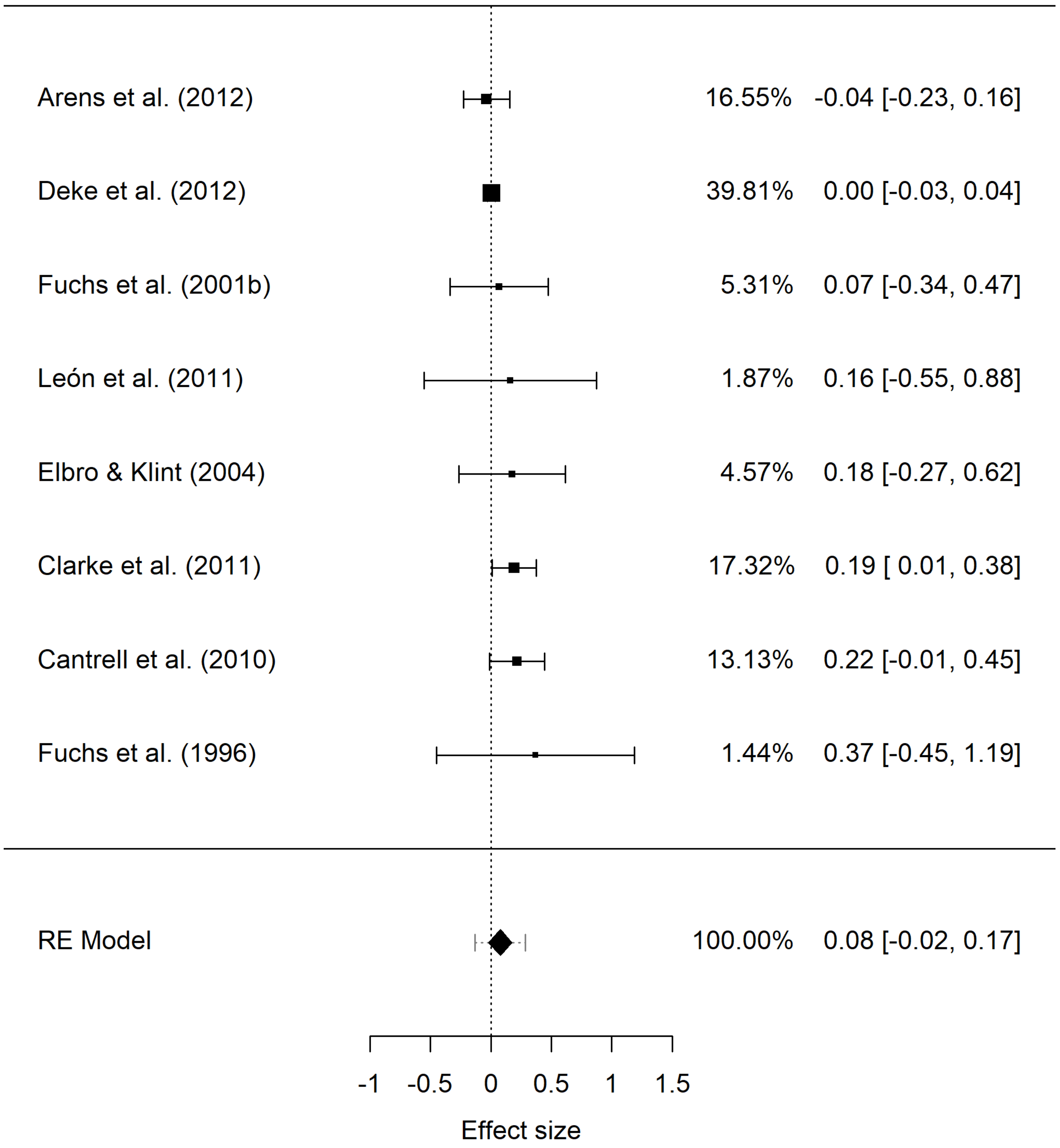

Heterogeneity: τ-squared = 0.006, I-squared = 37.0%, $Q(df = 7) = 8.7$, $p = 0.272$.

Figure A6. Forest plot of studies of interventions including peer-assisted instruction.

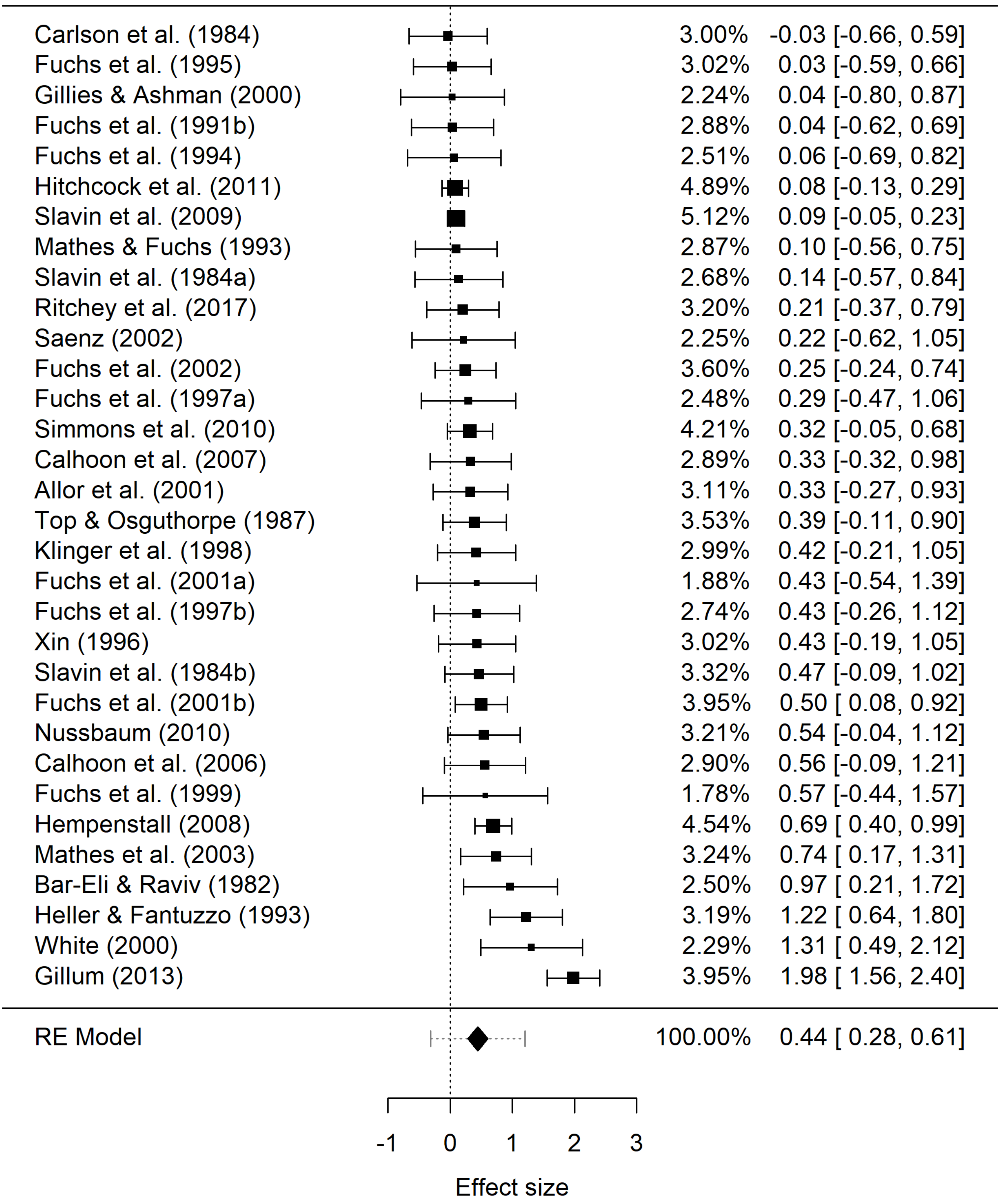

Heterogeneity: τ-squared = 0.132, I-squared = 70.2%, $Q(df = 31) = 108.3$, $p < 0.0001$.

Figure A7. Forest plot of studies of interventions including progress monitoring.

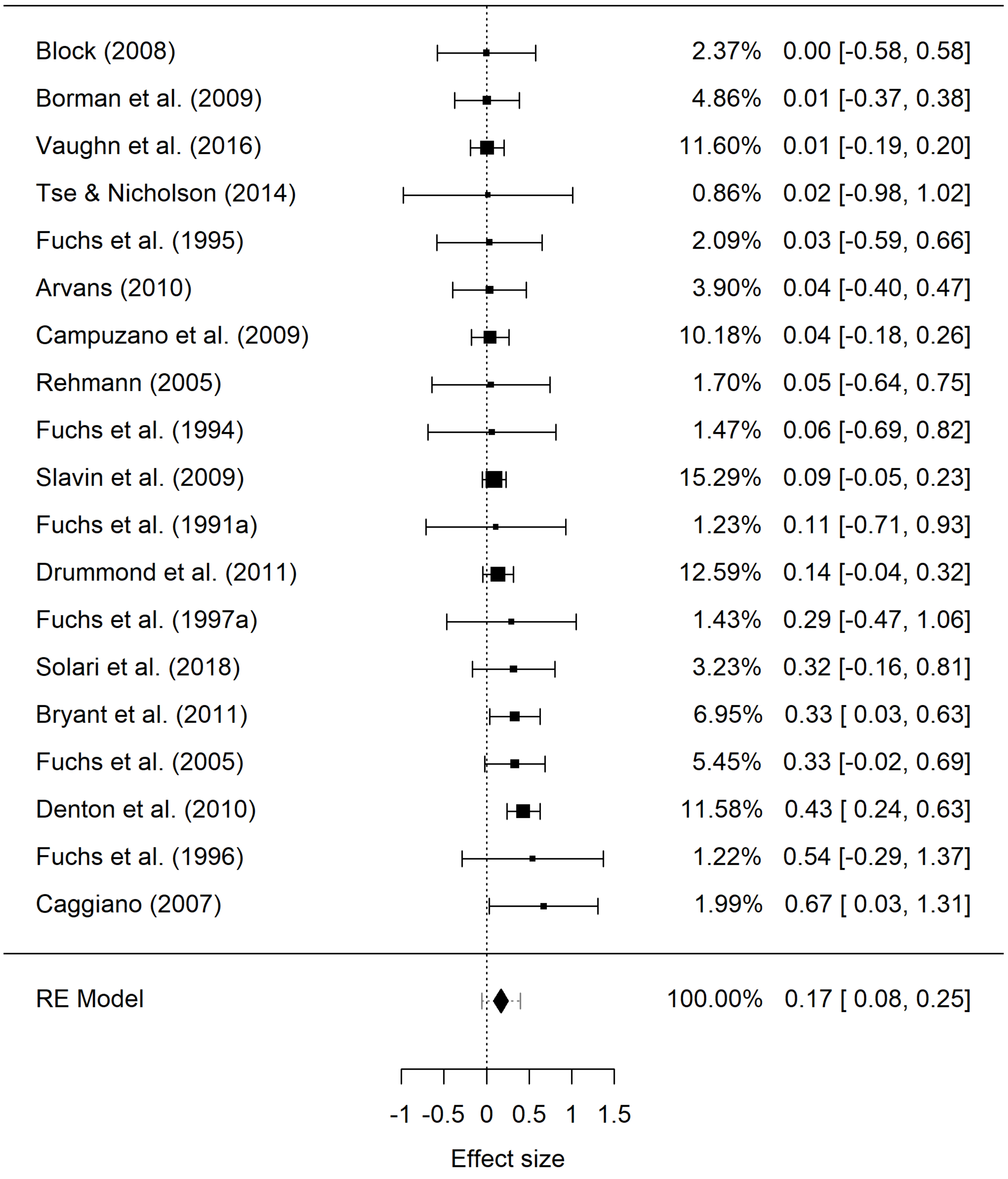

Heterogeneity: τ-squared = 0.010, I-squared = 27.9%, $Q(df = 18) = 19.7$, $p = 0.350$.

Figure A8. Forest plot of studies of interventions including small-group instruction.

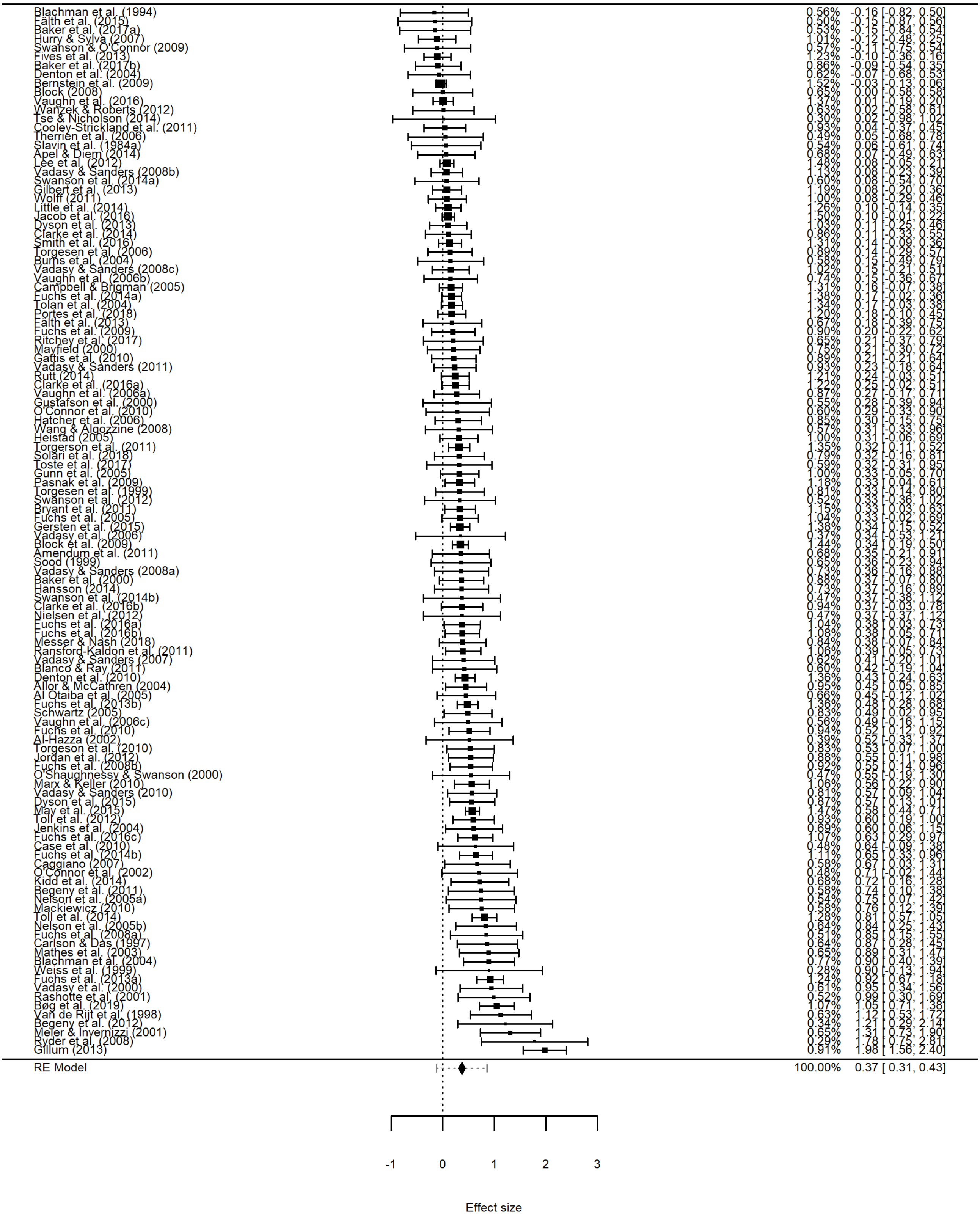

Heterogeneity: τ-squared = 0.061, I-squared = 67.9%, $Q(df = 117) = 363.9$, $p < 0.0001$.

Figure A9. Forest plot of studies of interventions targeting comprehension.

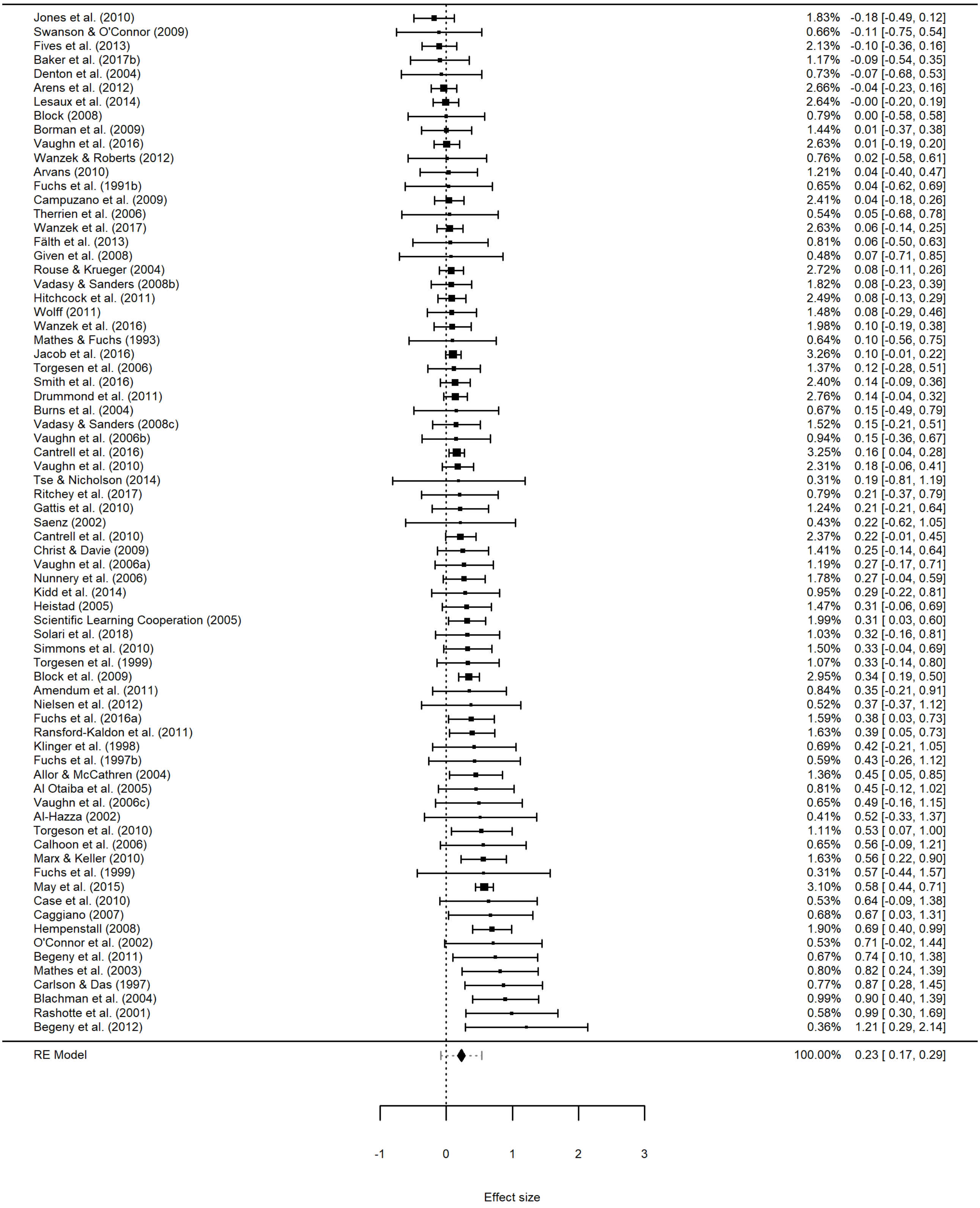

Heterogeneity: τ-squared = 0.023, I-squared = 48.6%, $Q(df = 72) = 139.8$, $p < 0.0001$.

Figure A10. Forest plot of studies of interventions targeting decoding.

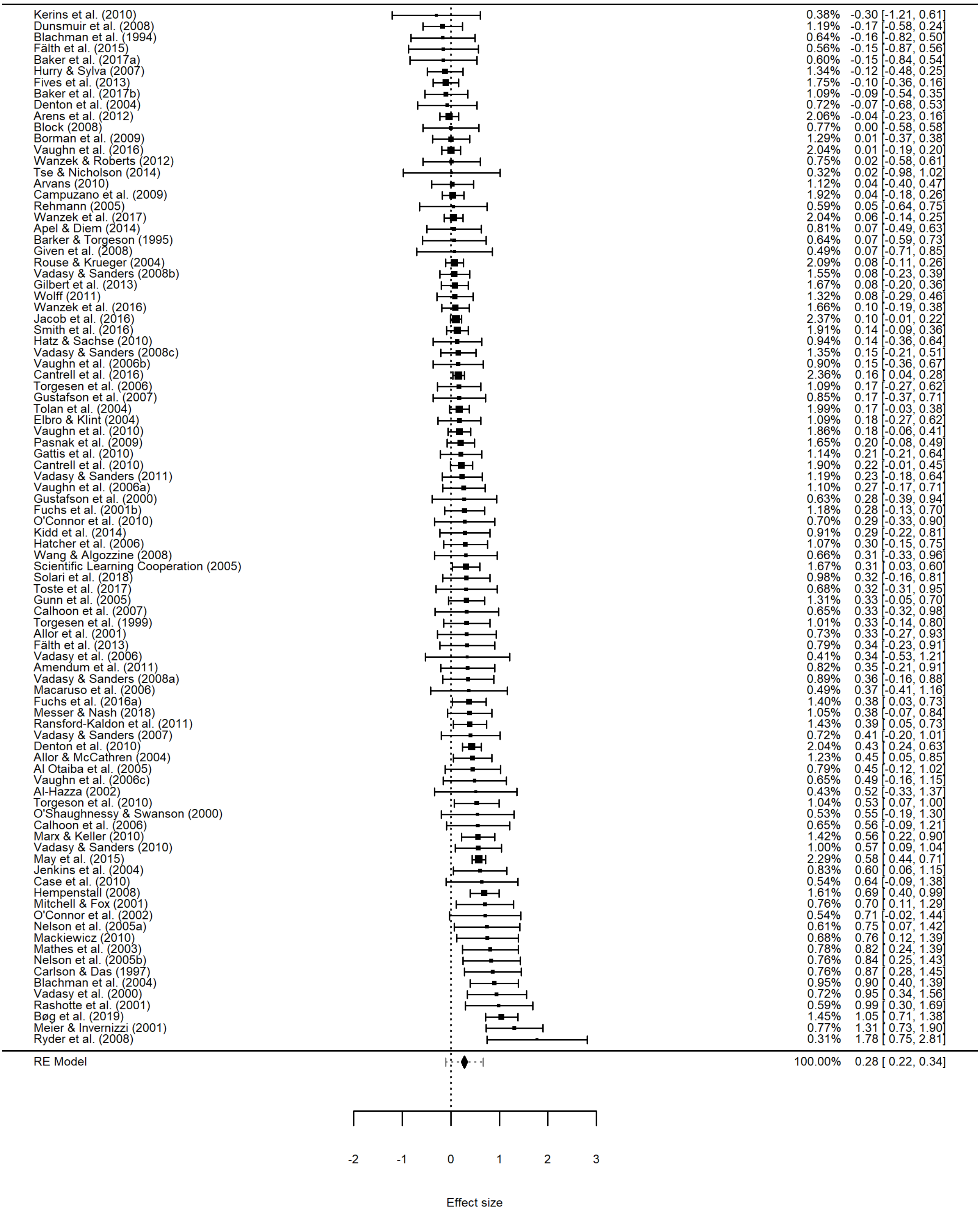

Heterogeneity: τ-squared = 0.037, I-squared = 60.8%, $Q(df = 90) = 233.3$, $p < 0.0001$.

Figure A11. Forest plot of studies of interventions targeting fluency.

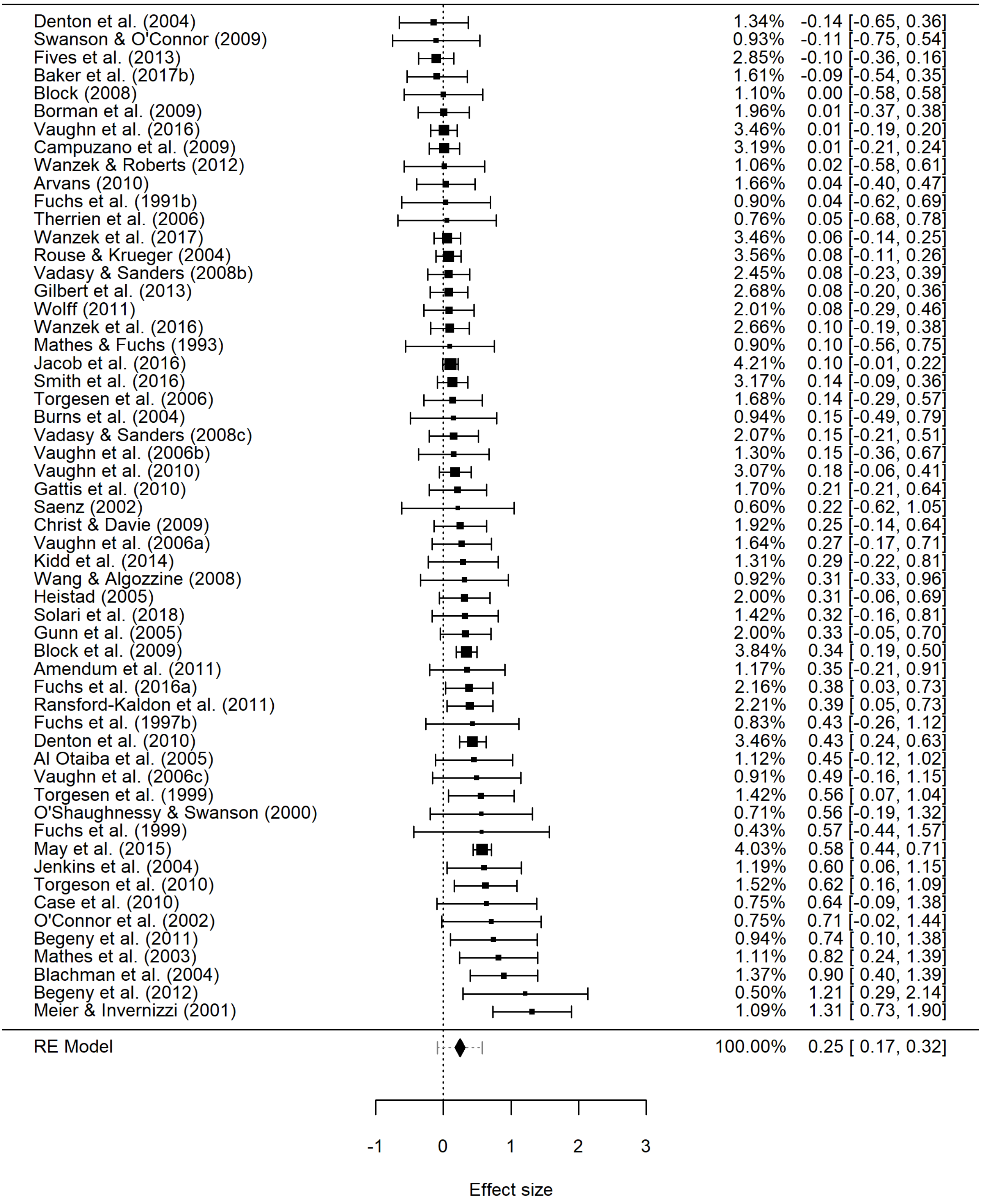

Heterogeneity: τ-squared = 0.026, I-squared = 48.9%, $Q(df = 55) = 115.4$, $p < 0.0001$.

Figure A12. Forest plot of studies of interventions targeting multiple reading domains.

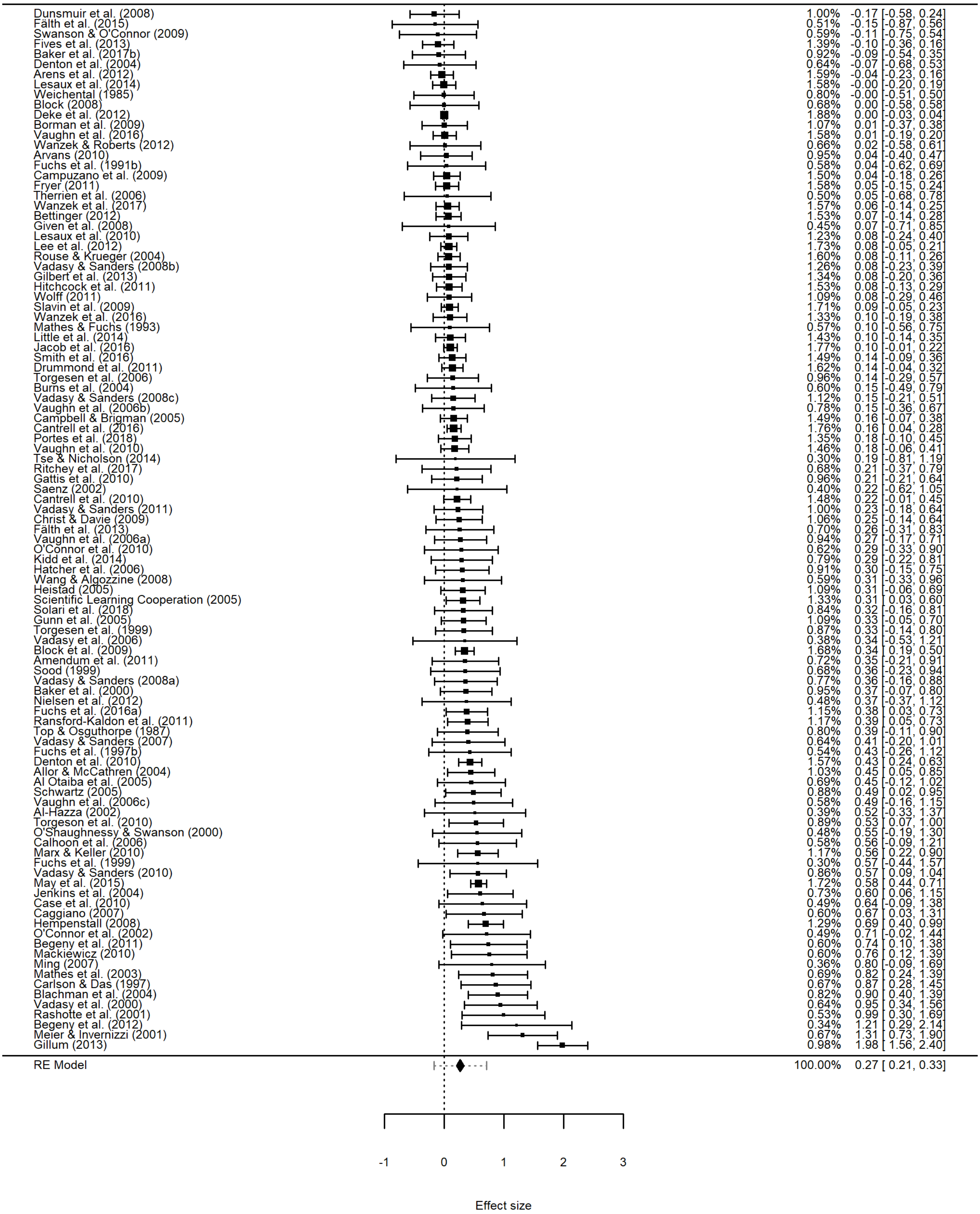

Heterogeneity: τ-squared = 0.049, I-squared = 74.4%, $Q(df = 102) = 336.2$, $p < 0.0001$.

Figure A13. Forest plot of studies of interventions targeting spelling and writing.

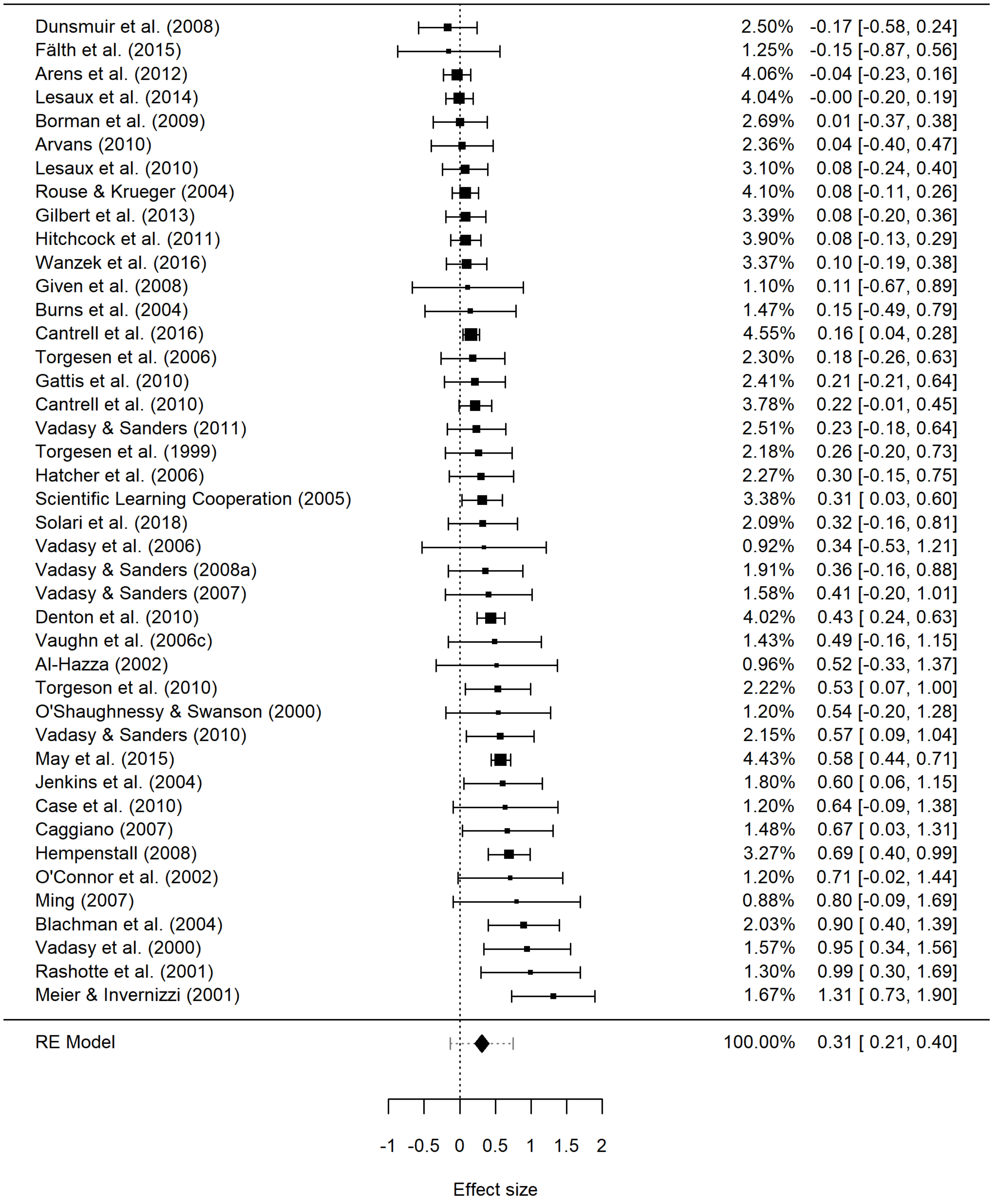

Heterogeneity: τ-squared = 0.046, I-squared = 63.5%, $Q(df = 41) = 110.5$, $p < 0.0001$.

Figure A14. Forest plot of studies of interventions targeting vocabulary.

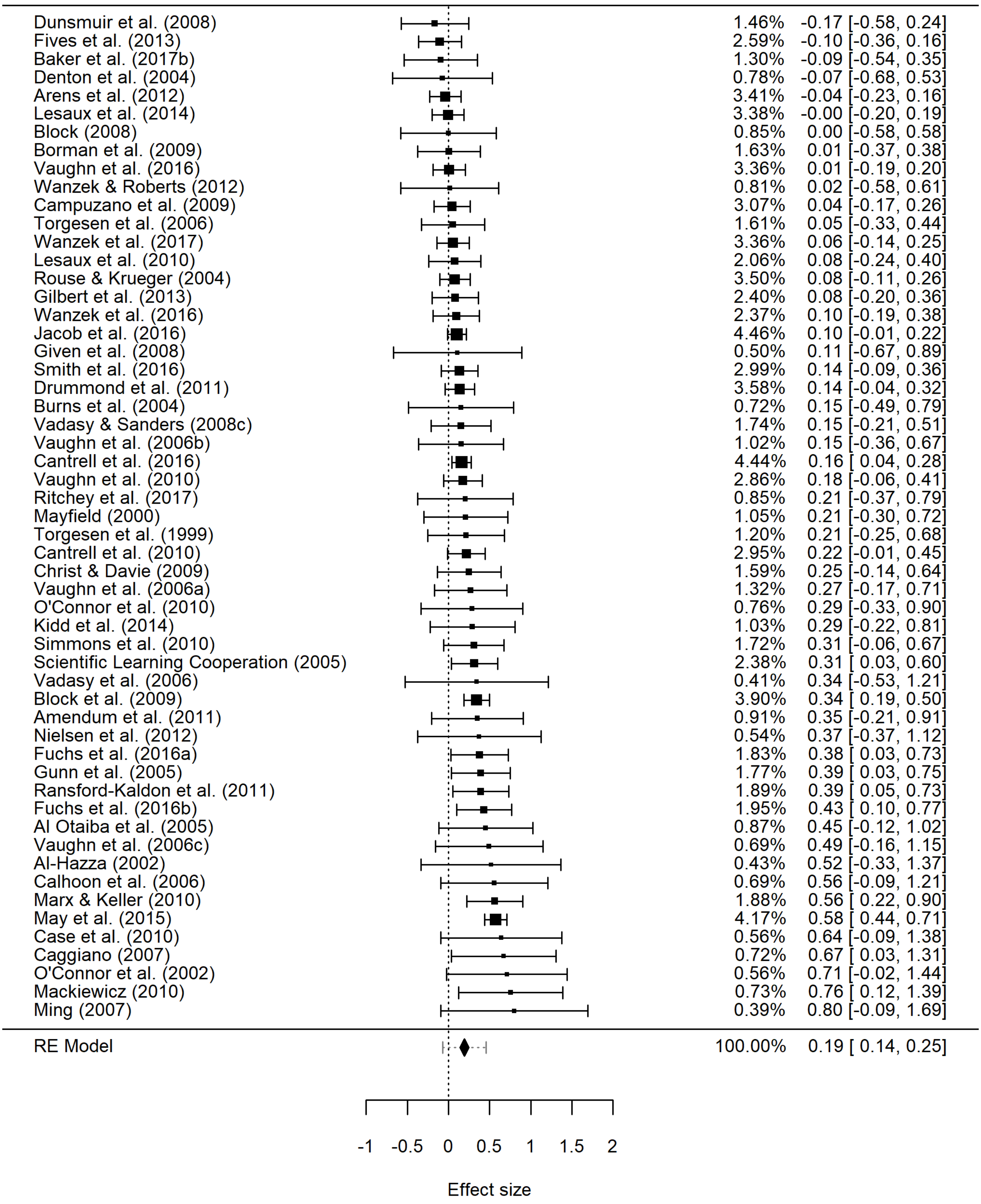

Heterogeneity: τ-squared = 0.016, I-squared = 43.7%, $Q(df = 54) = 95.4$, $p = 0.0004$.

Figure A15. Forest plot of studies of interventions targeting algebra/pre-algebra.

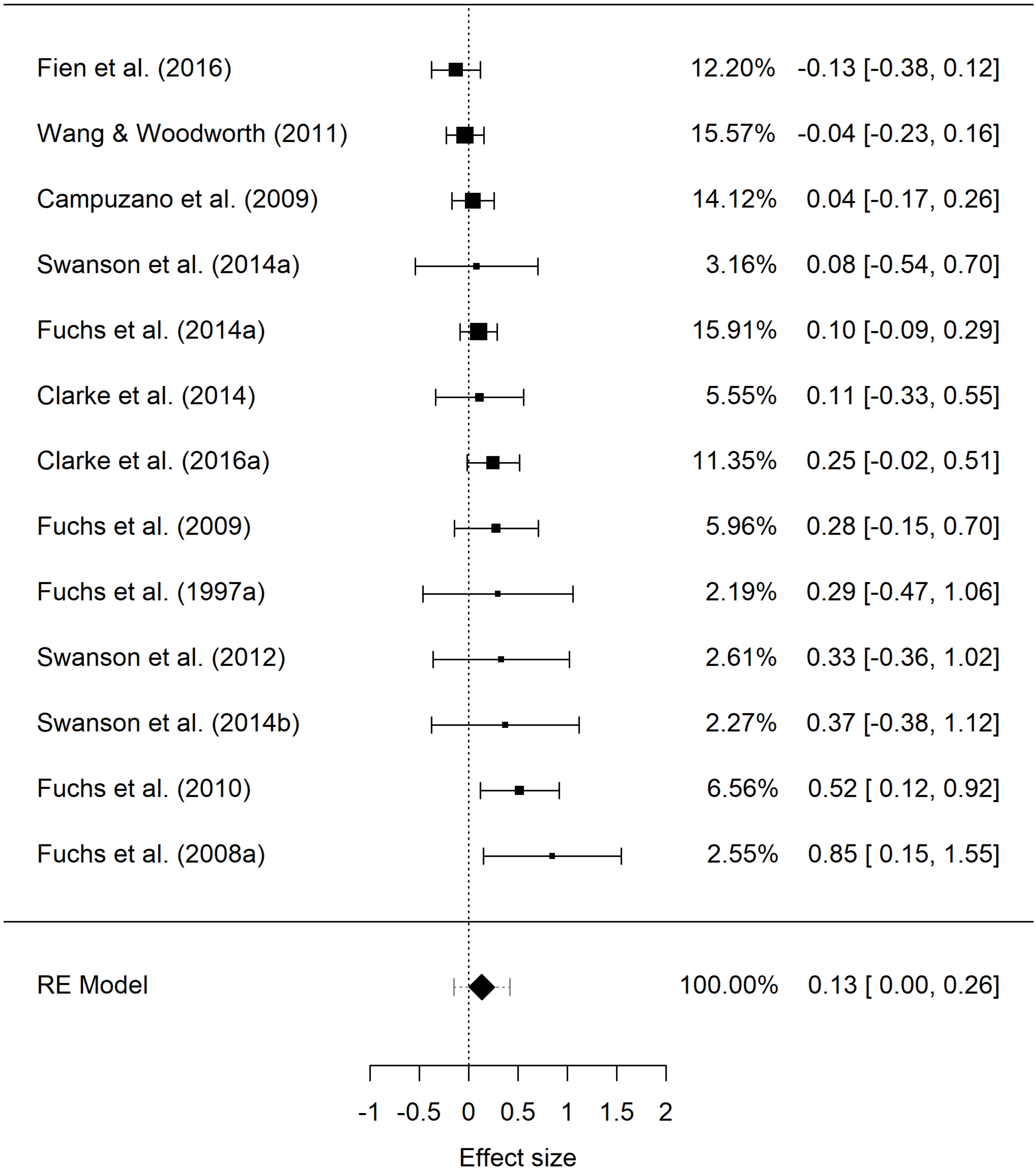

Heterogeneity: τ-squared = 0.014, I-squared = 33.4%, $Q(df = 12) = 17.2$, $p = 0.143$.

Figure A16. Forest plot of studies of interventions targeting fractions.

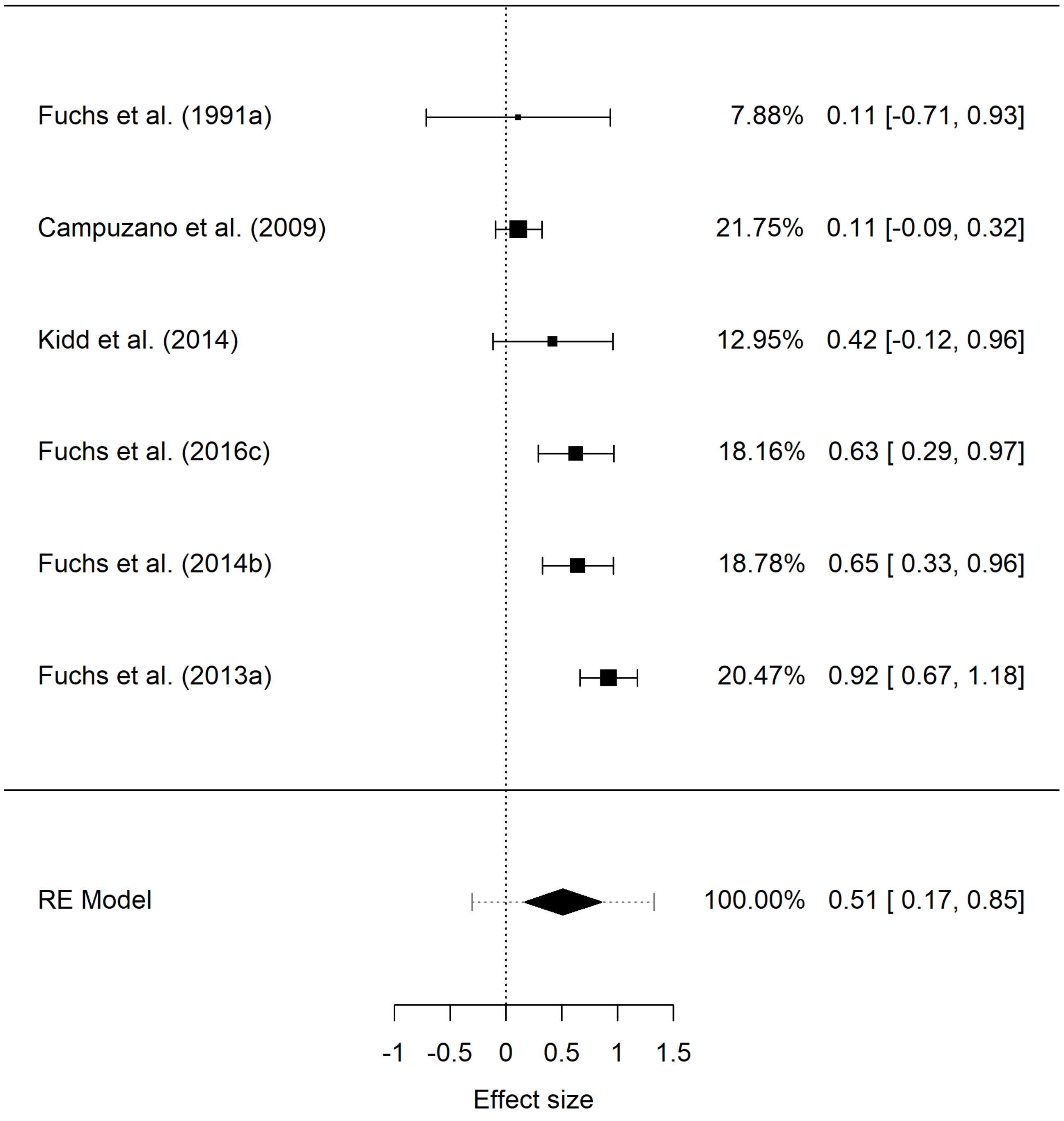

Heterogeneity: τ-squared = 0.083, I-squared = 74.8%, $Q(df = 5) = 26.0$, $p < 0.0001$.

Figure A17. Forest plot of studies of interventions targeting geometry.

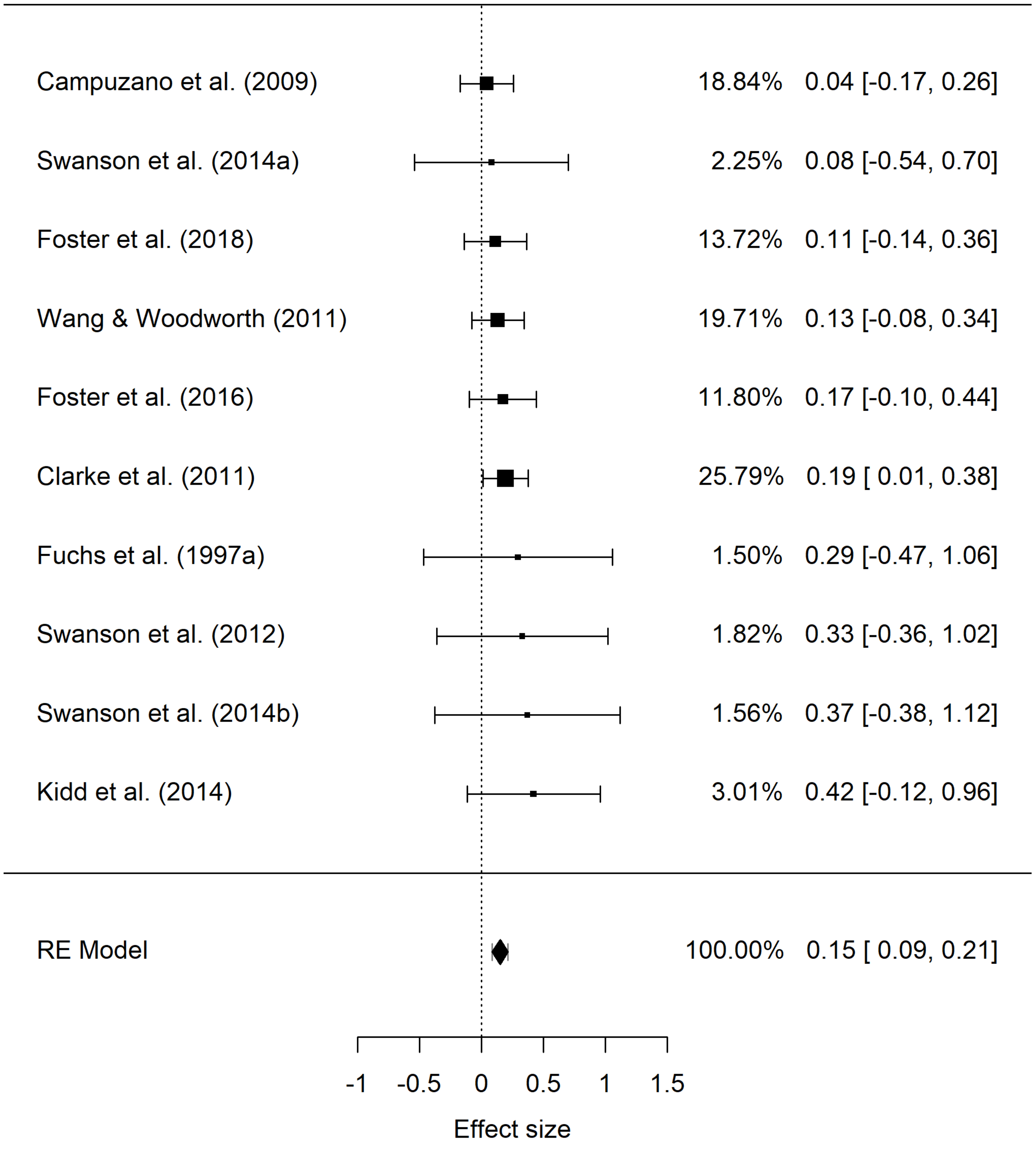

Heterogeneity: τ-squared = 0, I-squared = 0%, $Q(df = 9) = 3.09$, $p < 0.961$.

Figure A18. Forest plot of studies of interventions targeting multiple math domains.

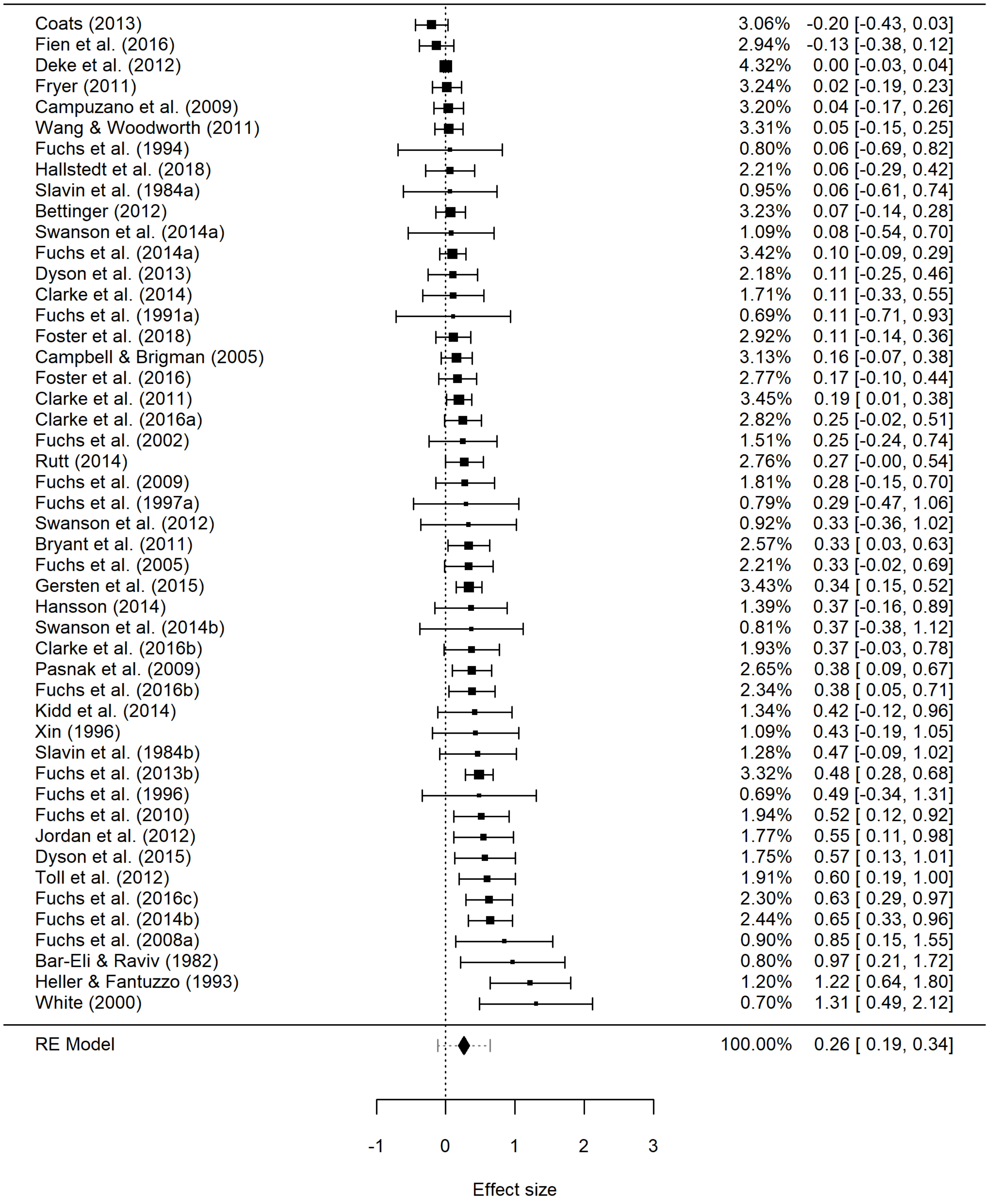

Heterogeneity: τ-squared = 0.033, I-squared = 67.9%, $Q(df = 47) = 156.7$, $p < 0.0001$.

Figure A19. Forest plot of studies of interventions targeting number sense.

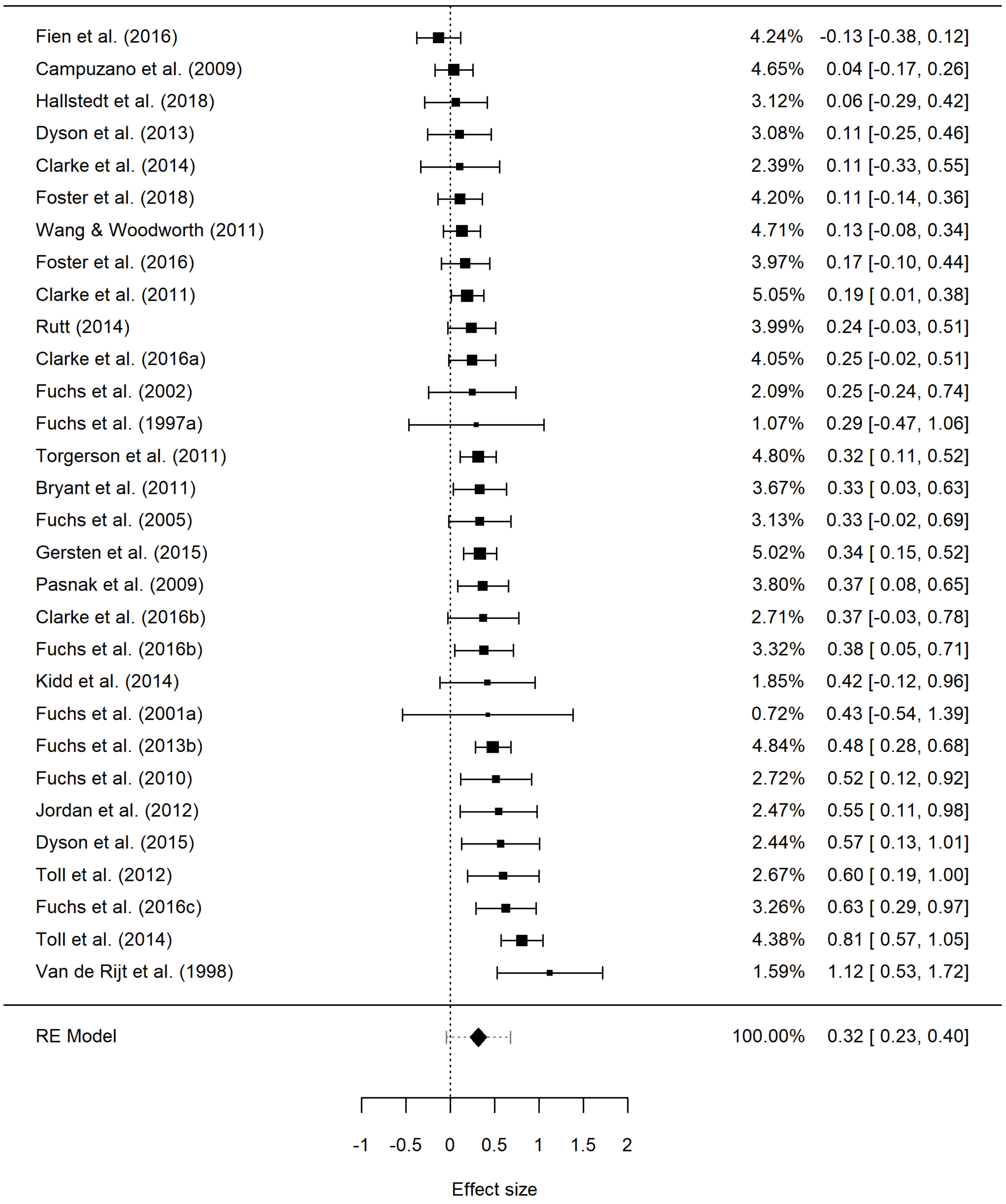

Heterogeneity: τ-squared = 0.030, I-squared = 57.0%, $Q(df = 29) = 66.7$, $p < 0.0001$.

Figure A20. Forest plot of studies of interventions targeting operations.

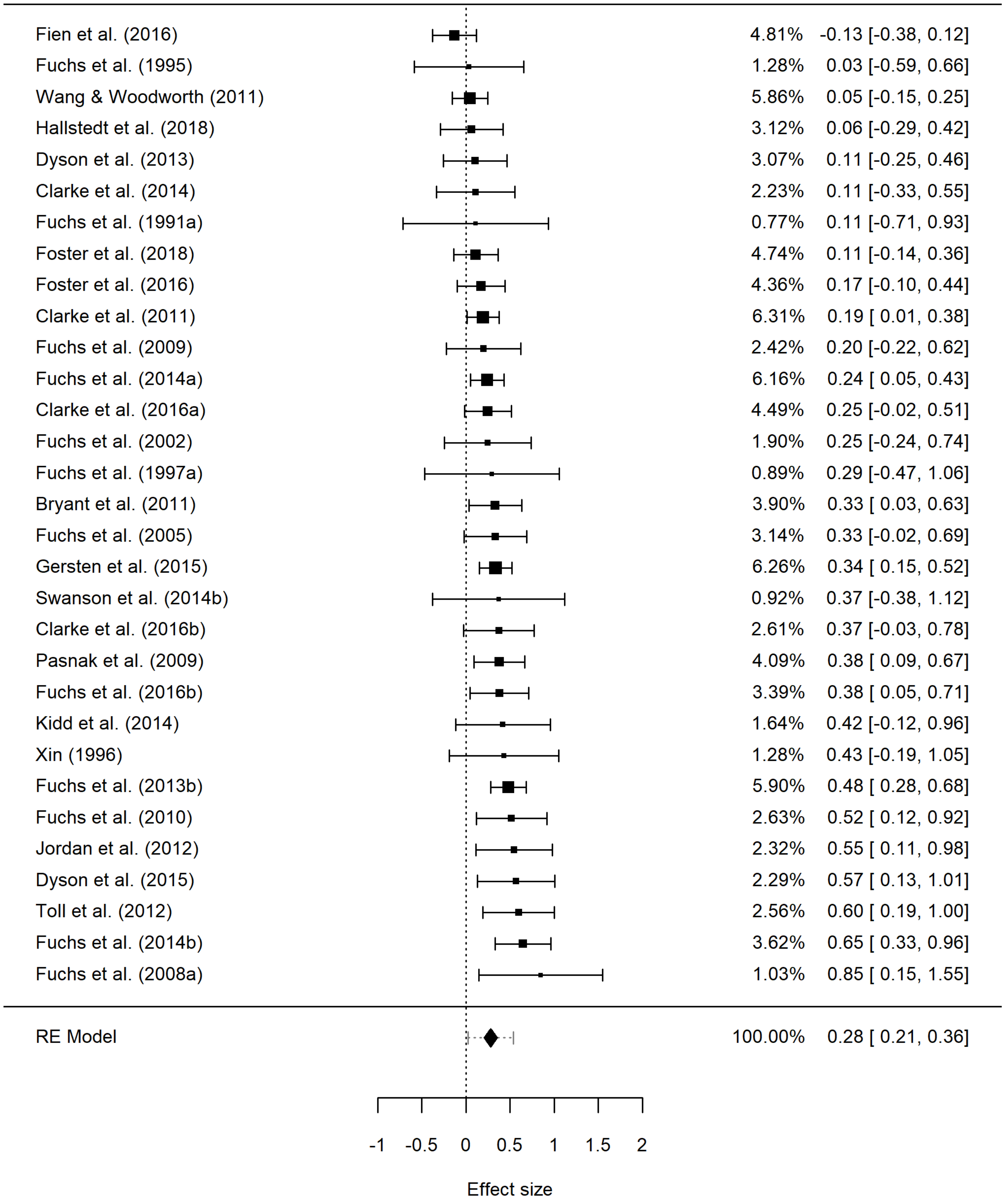

Heterogeneity: τ-squared = 0.015, I-squared = 36.2%, $Q(df = 30) = 48.3$, $p = 0.056$.

Figure A21. Forest plot of studies of interventions targeting problem-solving.

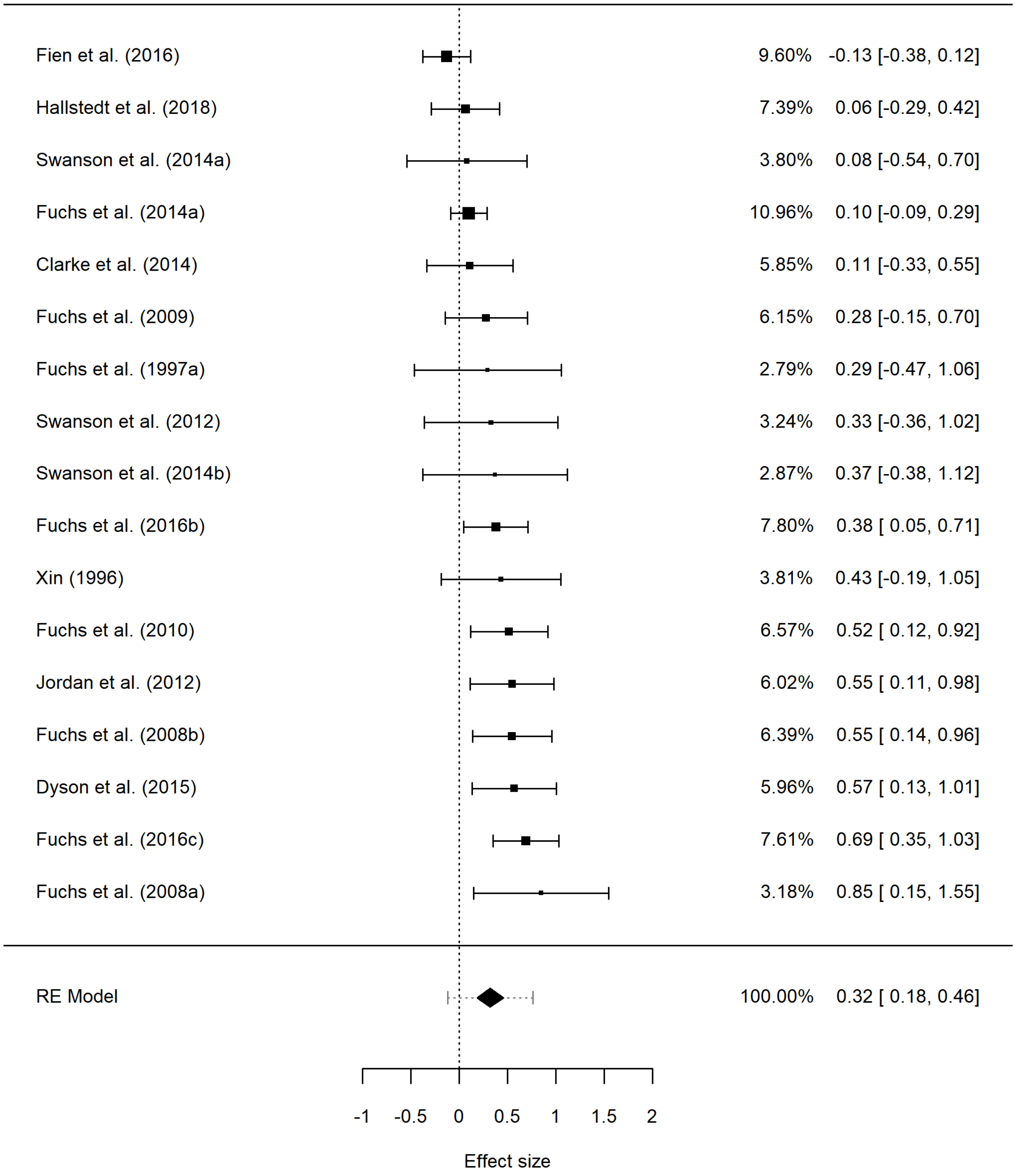

Heterogeneity: τ-squared = 0.039, I-squared = 49.4%, $Q(df = 16) = 31.0$, $p = 0.014$.

Figure A22. Forest plot of studies of interventions targeting general academic skills.

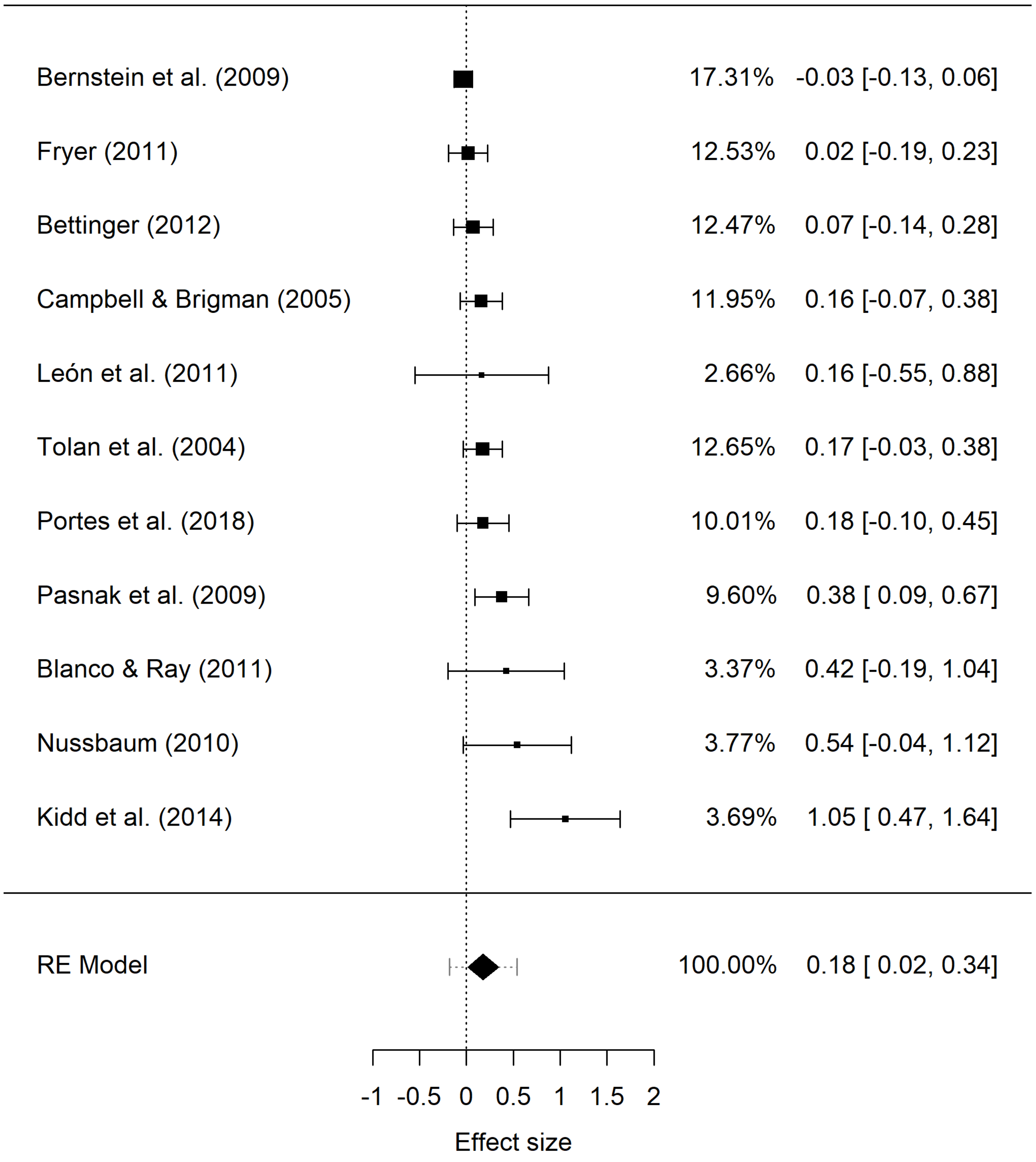

Heterogeneity: τ-squared = 0.021, I-squared = 58.0%, $Q(df = 10) = 25.7$, $p = 0.004$.

Figure A23. Forest plot of studies of interventions targeting meta-cognitive strategies.

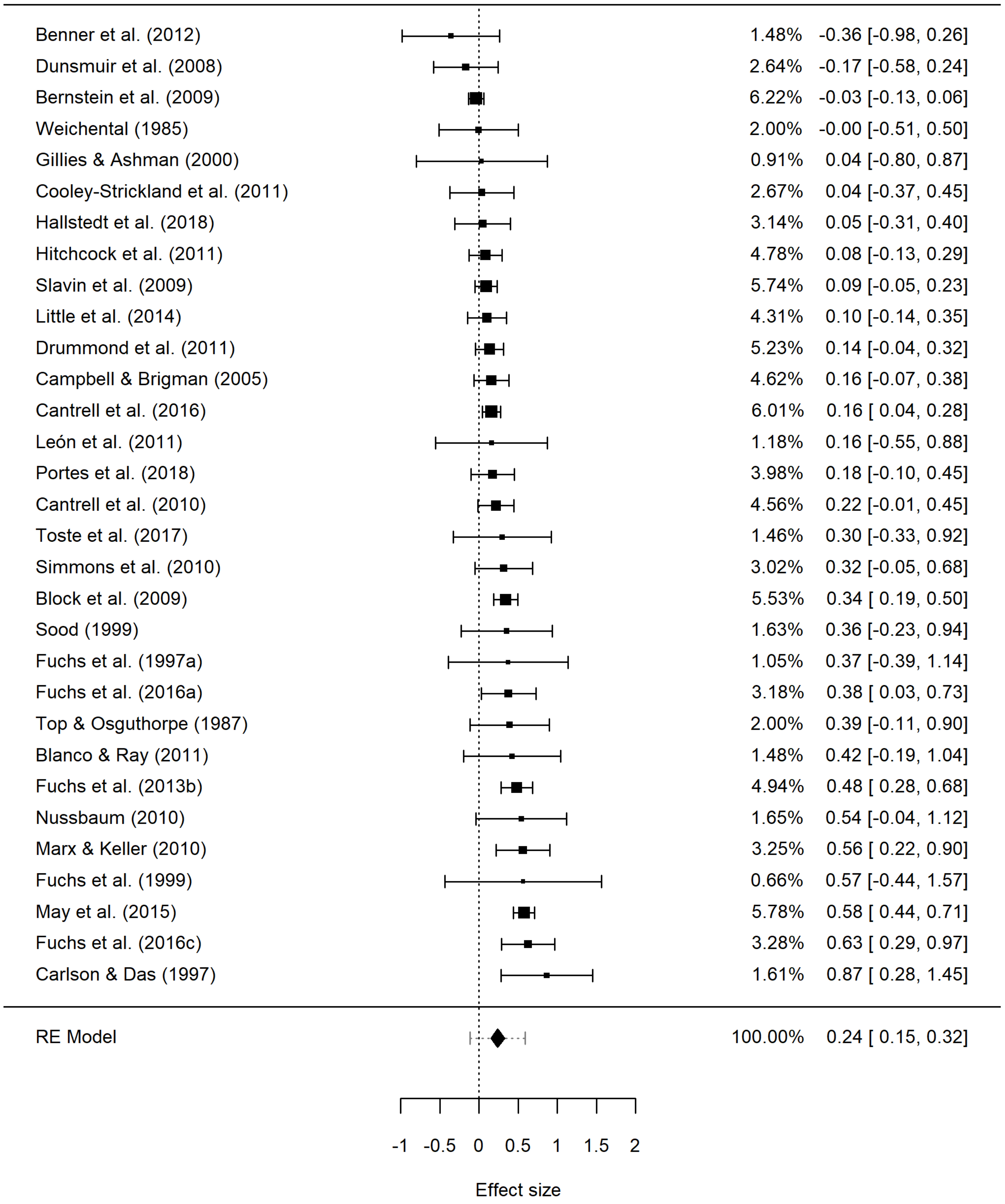

Heterogeneity: τ-squared = 0.028, I-squared = 64.4%, $Q(df = 30) = 95.9$, $p < 0.0001$.

Figure A24. Forest plot of studies of interventions targeting social-emotional skills.

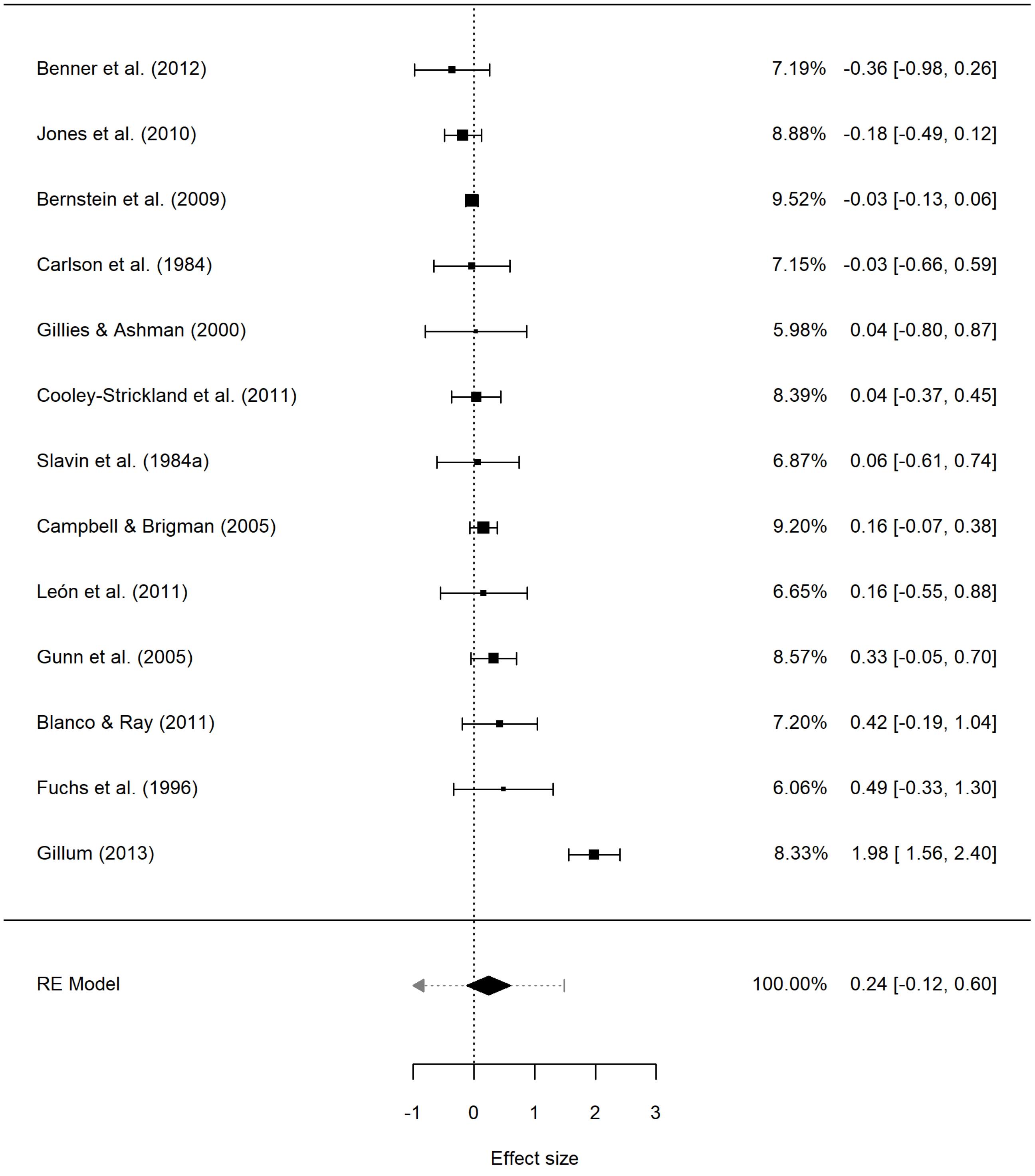

Heterogeneity: τ-squared = 0.30, I-squared = 90.6%, $Q(df = 12) = 93.2$, $p < 0.0001$.
