# Supplementary material for: Targeted school‐based interventions for improving reading and mathematics for students with or at risk of academic difficulties in Grades K‐6: A systematic review
Source: Campbell Syst Rev. 2021 Apr 6;17(2):e1152. doi: 10.1002/cl2.1152 (PMC8356298; doi:10.1002/cl2.1152)
Supplement: Supplementary file 11 — Supporting information [file CL2-17-e1152-s011.pdf]

Online Appendix K: Extra sensitivity analyses

This Online Appendix reports results from sensitivity analyses that we mentioned in the main text but where the quantitative results were not included. There were three instances: In the Peer-assisted instruction and small-group instruction-subsection, we conducted sensitivity analyses for the group size analysis using comparison designs. In the Outliers-subsection, in addition to winsorizing outliers, we also commented on specifications in which we sequentially removed large and small effect sizes. Lastly, in the Clustered assignment to treatment-subsection, we mentioned analyses using an ICC of 0.3. We describe these three analyses below.

Sensitivity of group size analyses

Figure K1 shows a forest plot including the studies in the group size analysis. The effect size estimate is very similar to the primary analysis. Although the heterogeneity statistics indicate a low level of heterogeneity (Q =2.9, I-squared = 16.9%, τ-squared = 0.02, there are only four studies and these statistics are unreliable. The prediction interval is wide and the min-max range is also relatively wide (from -0.39 to 0.22).

We then ran a specification where we adjusted for pre-test differences in the one study where we could only use raw means to calculate the effect sizes. We adjusted by subtracting the pre-test mean in the intervention and control groups from their respective post-test mean. That is, we used the differential gain score as the estimate of mean differences. We then calculated Hedges' g as in the primary analysis (i.e., we used the unadjusted post-test standard deviation). The estimate indicated a slightly higher advantage of one-to-one-tutoring (ES = -0.17, CI = [-0.71, 0.37]) but the effect size estimate is far from significant and the adjusted degrees of freedom only 2.5.

Lastly, we excluded the one intervention that used groups of five students (all others contrasted one-to-one with groups of two or three), which also yielded similar results (ES = -0.14, CI = [-0.70, 0.42]).

Figure K1. Forest plot of group size analysis

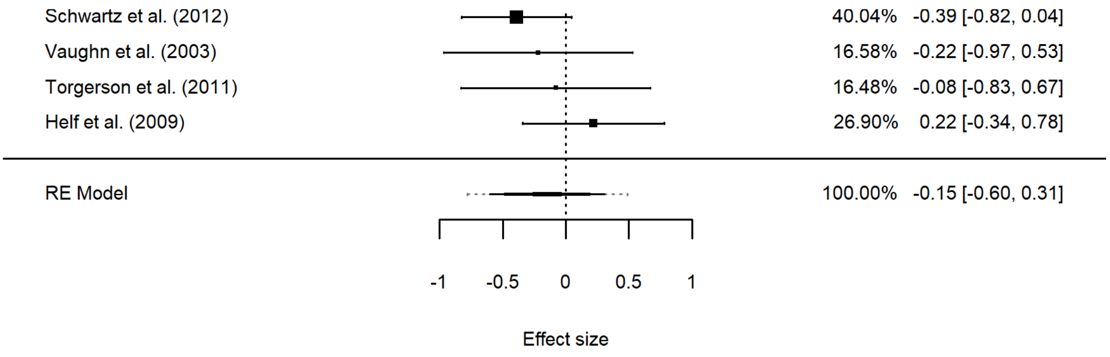

Outliers

Figure K2-K5 displays the effect size distributions for short-term effect sizes (the same as Figure 5 in the main text), follow-up effect sizes, peer-assisted instruction effect sizes, and small-group instruction effect sizes (the latter two from single method interventions). There are quite a few outliers among the short-term and small-group instruction effect sizes, which sense the latter are included in the former, are often the same. The distributions of follow-up and peer-assisted instruction effect sizes display fewer outliers.

Figure K2. Distribution of short-term effect sizes

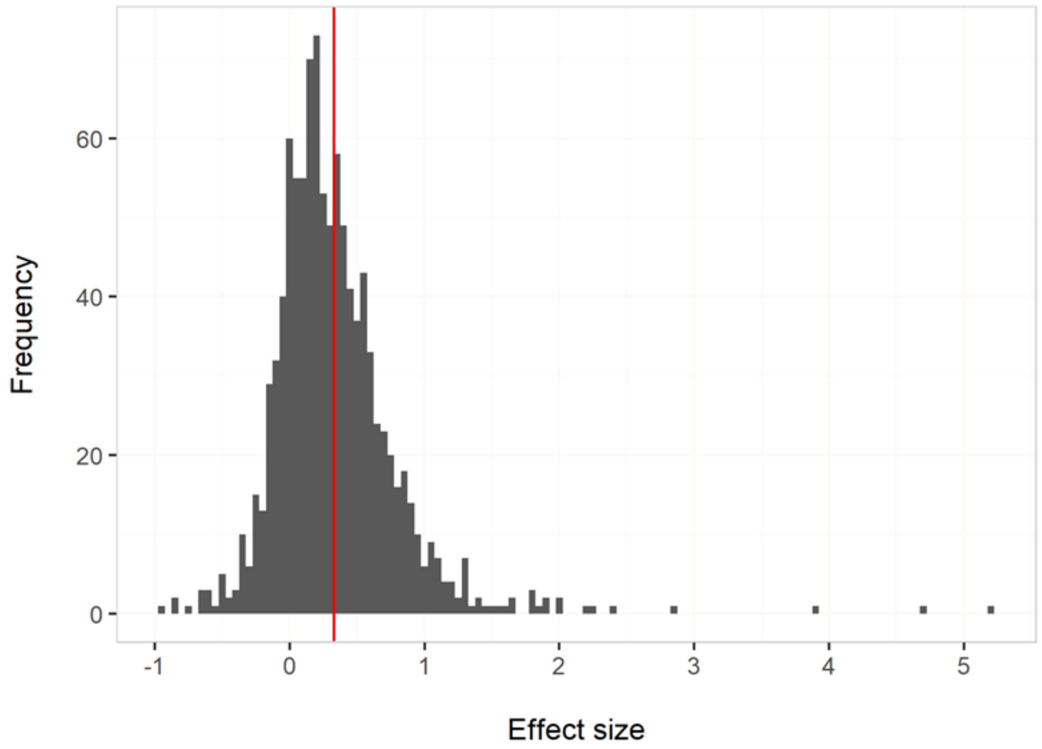

Figure K3. Distribution of follow-up effect sizes

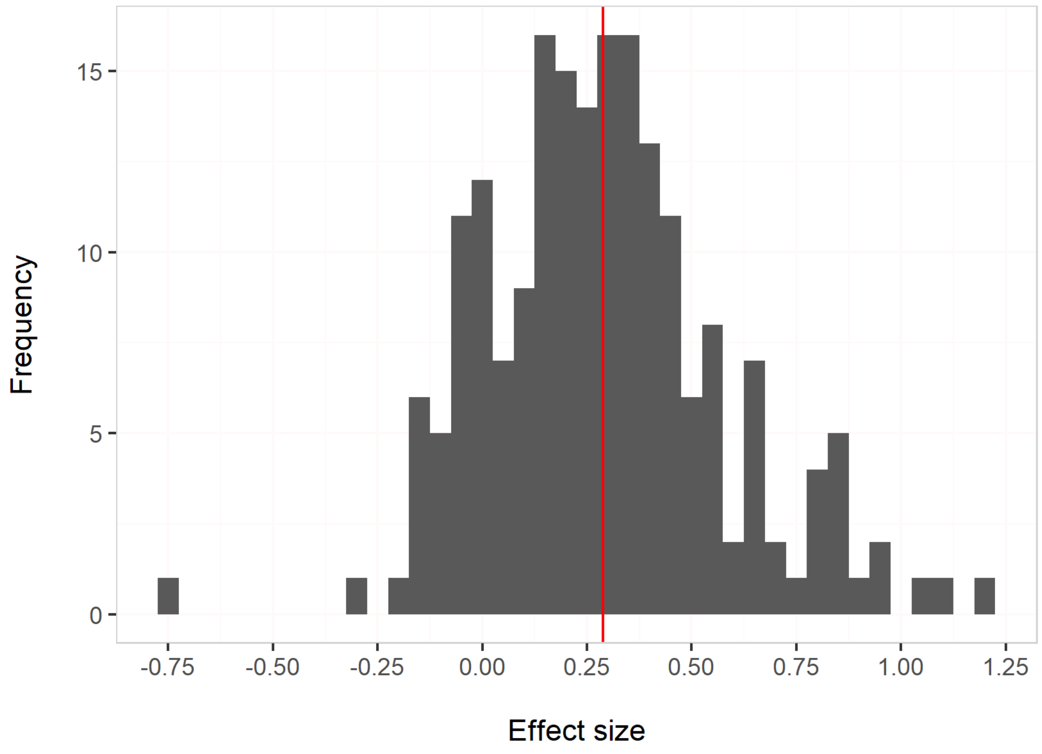

Figure K4. Distribution of peer-assisted instruction effect sizes from single method interventions

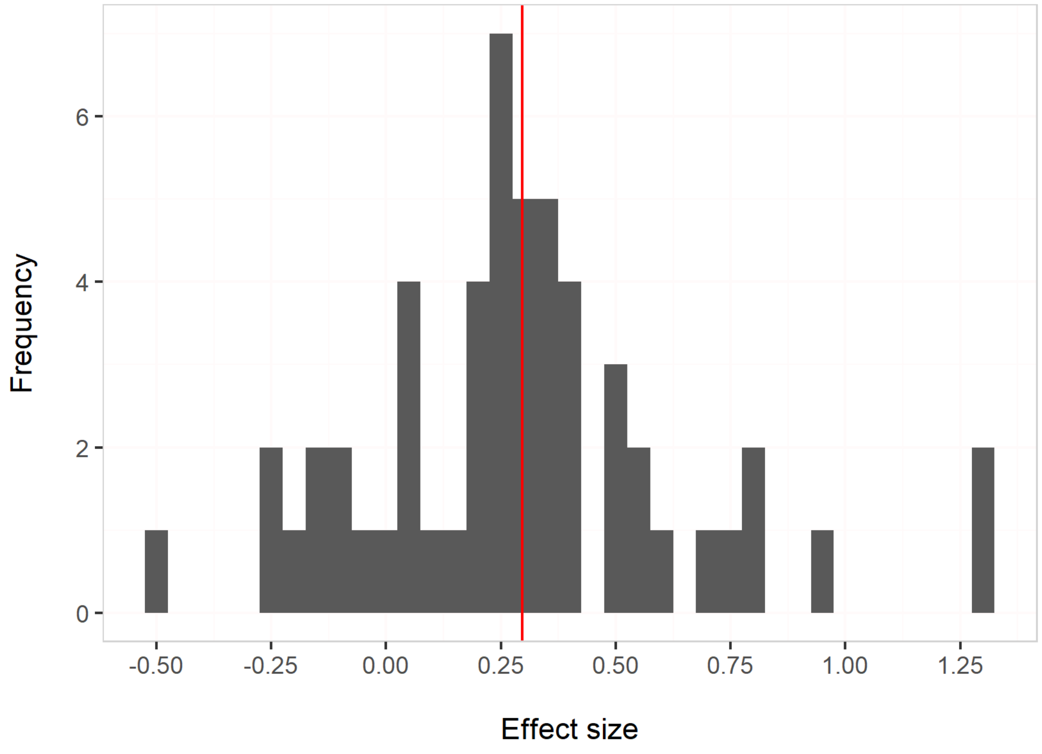

Figure K5. Distribution of small-group instruction effect sizes from single method interventions

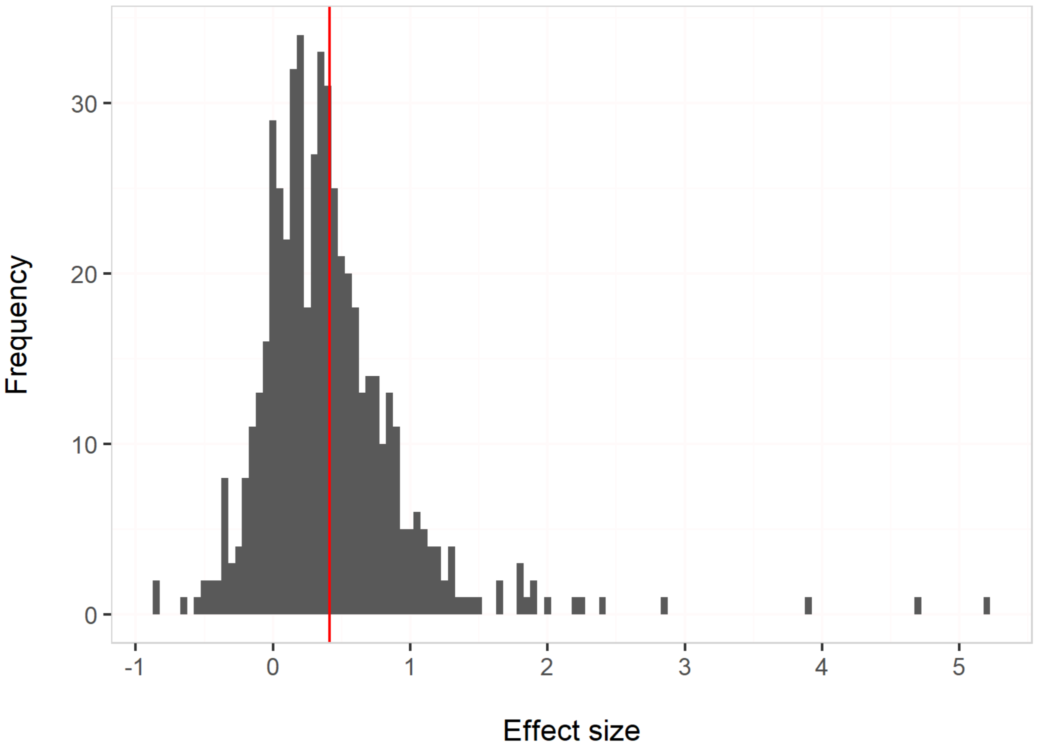

In the main text, we showed results where we winsorized the effect sizes at -0.5 and 1.5, which corresponds approximately to where the short-term/small-group instruction effect size distributions start to thin out. Below, we report results from harsher tests, where we instead removed outliers sequentially. We start by removing outliers in specifications corresponding to cutoffs corresponding to the ones used for winsorizing (i.e., -0.5 and 1.5). We then remove all effect sizes below -0.25 and above 1.5. Lastly, we remove effect sizes below -0.25 and 1.

As shown in Figures K6-K9, none of our results is particularly sensitive to removing outliers this way. The weighted average effect sizes decrease somewhat but even with the harshest cutoffs, all are large and statistically significant.

We also ran the meta-regressions corresponding to the specifications in column 2, Table 5, in the main text imposing the same cutoffs sequentially. For comparison, peer-assisted had β = 0.39 and CI = [0.13, 0.64] in that specification, and small-group instruction had β = 0.32, and CI = [0.11, 0.53]. Removing outliers with cutoffs -0.5 < ES < 1.5, peer-assisted had β = 0.26, and CI = [0.11, 0.40], and small-group instruction β = 0.22 and CI = [0.12, 0.31]. Removing outliers with cutoffs -0.25 < ES < 1.25, peer-assisted had β = 0.21 and CI = [0.07, 0.35], and small-group instruction β = 0.20 and CI = [0.11, 0.29]. Removing outliers with cutoffs -0.25 < ES < 1, peer-assisted had β = 0.17 and CI = [0.09, 0.30], and small-group instruction β = 0.17 and CI = [0.09, 0.25]. Thus, peer-assisted and small-group instruction retained sizeable and statistically significant associations with effect sizes also when remove outliers. However, the coefficients are smaller than the ones in the

primary analysis. Recall though that the coefficients in these specifications represent the marginal associations with effect sizes. When we remove outliers, the coefficient on the constant changes as well. It is 0.01 in the column 2, Table 5, and 0.08, 0.09, and 0.07 in the three outlier-analyses, respectively. The total marginal associations are therefore closer to the primary analysis.

It is also worth noting that as we remove outliers, the heterogeneity decrease by quite a lot. The I-squared is 62.2% and τ-squared is 0.053 in column 2, Table 5. This decreases to I-squared = 51.0%, τ-squared = 0.03, I-squared = 43.7%, τ-squared = 0.02, and I-squared = 34.4%, τ-squared = 0.02 in the three outlier-analyses.

Figure K6. Removing outliers, short-term effects

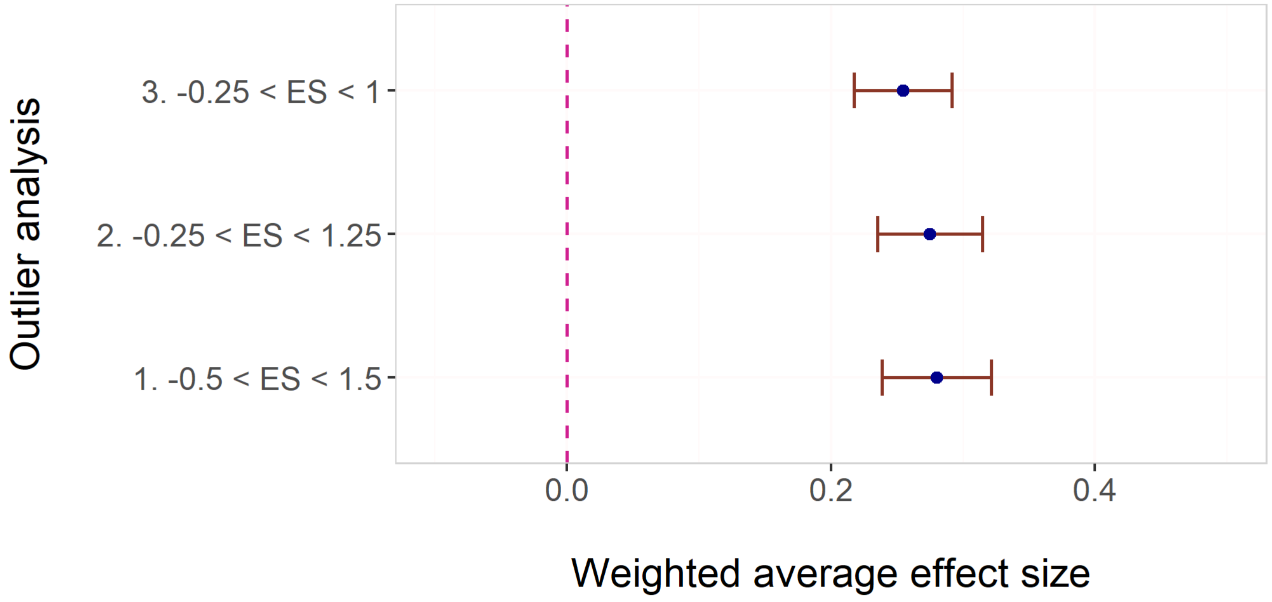

Figure K7. Removing outliers, follow-up effects

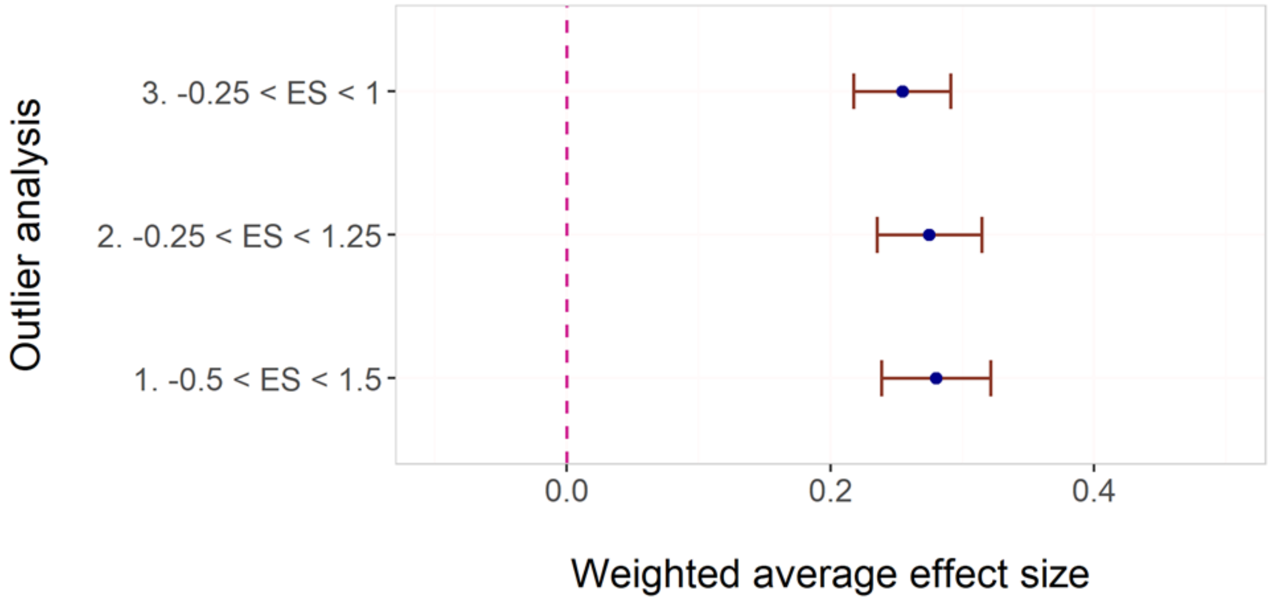

Figure K8. Removing outliers, peer-assisted instruction

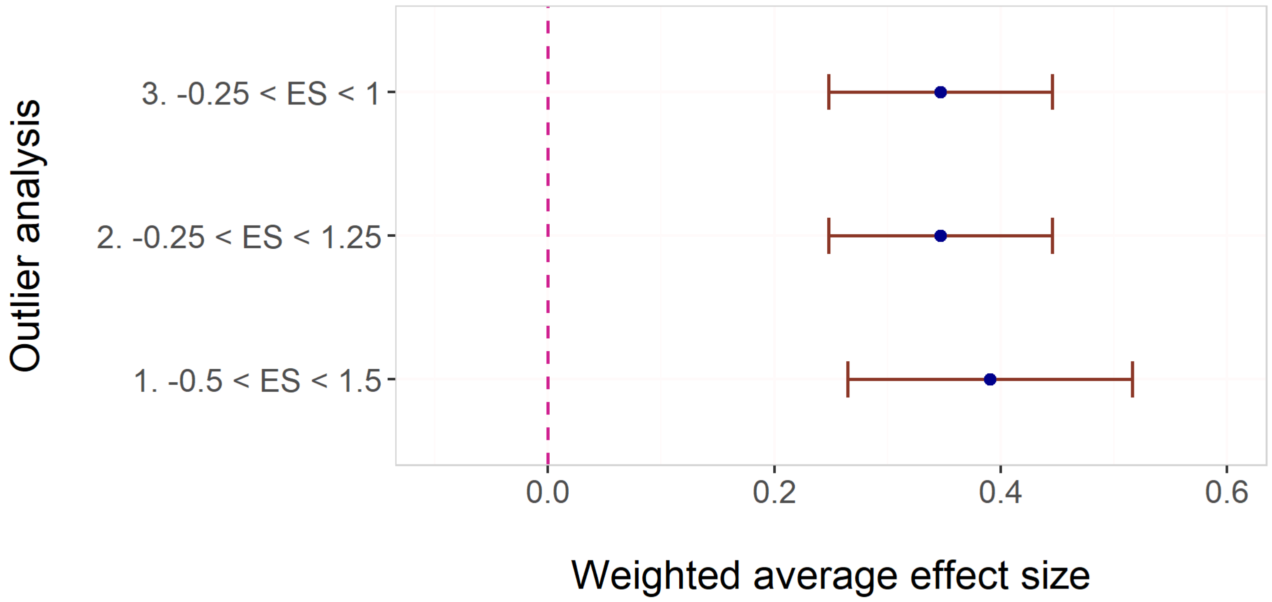

Figure K9. Removing outliers, small-group instruction

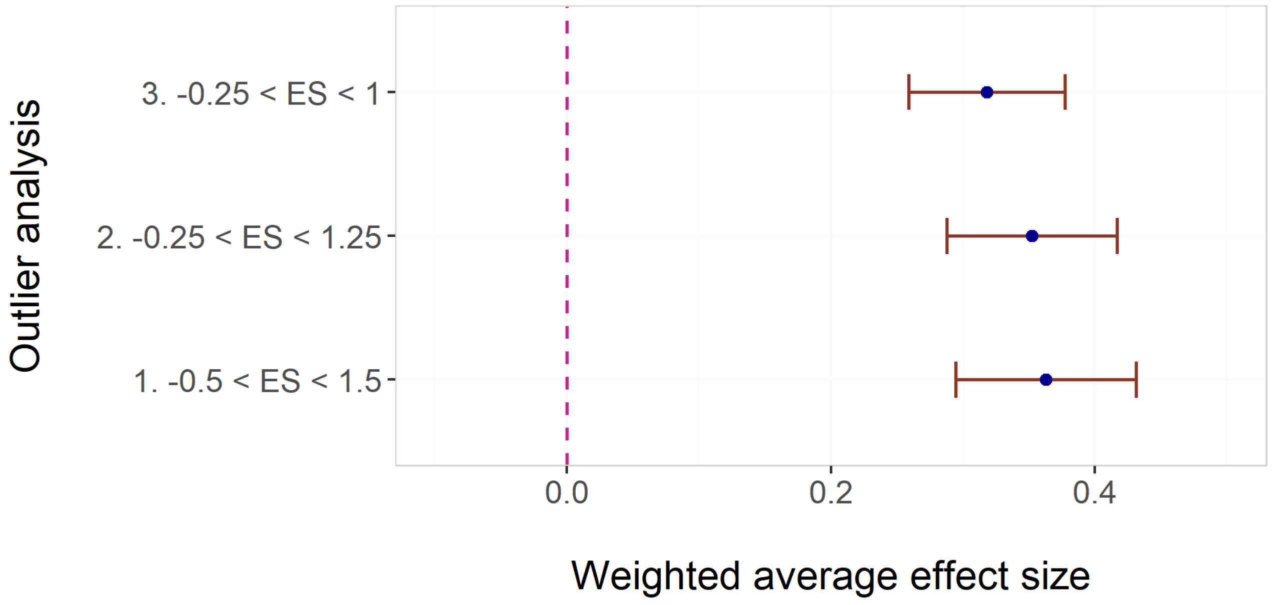

Sensitivity analysis of clustered assignment of treatment with a higher ICC

This section presents results where we used an ICC of 0.3 instead of an ICC of 0.09 to adjust effect sizes from studies using a clustered assignment procedure. Table K1 shows that the differences to the primary subgroup analysis were very small and all of our main results retained their significance with this substantially higher ICC. Note also that the heterogeneity statistics decrease in all cases except the *t*-squared for small-group instruction.

Table K1. Subgroup analysis comparing the primary analysis with an analysis using ICC = 0.3

	**Primary analysis**					**Cluster-adjustment, ICC = 0.3**				
	(1)	(2)	(3)	(4)	(5)	(6)	(7)	(8)	(9)	(10)
Analysis	*ES*	*CI*	*Q*	τ^2	I^2	*ES*	*CI*	*Q*	τ^2	I^2
Short-term	0.30	[0.25, 0.34]	797.3	0.07	76.4	0.30	[0.25, 0.35]	762.4	0.07	75.3
Follow-up	0.27	[0.17, 0.36]	47.8	0.03	45.6	0.27	[0.18, 0.36]	41.0	0.02	36.6
Peer-assisted	0.39	[0.26, 0.52]	18.7	0.02	19.8	0.40	[0.23, 0.56]	15.9	0.01	5.8
Small-group	0.38	[0.30, 0.45]	285.4	0.08	70.6	0.38	[0.31, 0.45]	278.2	0.09	69.8

Note: The short-term and follow-up results were reported in section *Overall short-term and medium- to long-term effects* of the main text, and the peer-assisted and small-group instruction results in Table 4 of the main text. Columns 6-10 report estimates where we have adjusted effect sizes from clustered designs using an ICC = 0.3.
