# Supplementary material for: Targeted school‐based interventions for improving reading and mathematics for students with or at risk of academic difficulties in Grades K‐6: A systematic review
Source: Campbell Syst Rev. 2021 Apr 6;17(2):e1152. doi: 10.1002/cl2.1152 (PMC8356298; doi:10.1002/cl2.1152)
Supplement: Supplementary file 12 — Supporting information [file CL2-17-e1152-s008.pdf]

Online Appendix L: Prediction intervals

This appendix presents the prediction intervals corresponding to the main analyses in the sections *Overall short-term and medium- to long-term effects* and *Results of the subgroup analysis and investigation of heterogeneity*. We calculated the 95% prediction intervals using the following formula from Borenstein et al. (2017):

$$\widehat{M} \pm t_{df} \times \sqrt{V_M + \tau^2}$$

where $\widehat{M}$ is the estimated mean effect size, t_{df} critical value of the t-distribution with the degrees of freedom (*df*) equal to the number of studies minus two, V_M is the variance of the mean effect size, and τ^2 is the estimated between-study variance. Overall, the prediction intervals support our heterogeneity analysis reported in the main text. Most of the results reported below indicate a substantial dispersion of the effect sizes, and thus heterogeneity.

Overall short-term and medium- to long-term effects

The 95% prediction interval for the weighted average short-term effect size (ES = 0.30, CI = [0.25, 0.34]) ranged from -0.22 to 0.81. The 95% prediction interval for the weighted average follow-up effect size was (ES = 0.27, CI = [0.17, 0.36]) ranged from -0.11 to 0.64. Both prediction intervals thus indicate a substantial amount of heterogeneity.

Results of the subgroup analysis and investigation of heterogeneity

Table K1 presents the prediction intervals corresponding to the subgroup analyses presented in Table 3 in the main text. All prediction intervals include zero and are wide. These results thus support the conclusion in the from the heterogeneity analyses presented in the main text, that is, there was substantial heterogeneity also within the subgroups created by separating interventions by the instructional methods used and the targeted content domains.

Table K2 presents the prediction intervals corresponding to the subgroup analyses presented in Table 4 in the main text (i.e., single method and single domain interventions). As mentioned in the main text, the single method and single domain studies are in most cases few and we believe the results should be interpreted with caution. This holds for coaching (3 studies), incentives (3), comprehension (3), and operations (3), and in particular for progress monitoring (2) that has too few studies to be calculated. The results are again in line with the heterogeneity analysis presented in the main text: with the exception of peer-assisted instruction, all components have intervals that include zero, and all intervals are wide.

Table K1. Subgroup analyses: Effect sizes, confidence intervals, and prediction intervals by intervention component

Component	Avg. ES	95%CI lower bound	95%CI upper bound	95% PI lower bound	95% PI lower bound
CAI	0.151	0.086	0.217	-0.101	0.403
Coaching	0.200	0.084	0.316	-0.260	0.660
Incentives	0.328	0.184	0.472	-0.243	0.899
Medium-group	0.320	0.054	0.587	-0.595	1.235
Other method	0.116	0.006	0.225	-0.516	0.748
Peer-assisted	0.444	0.276	0.613	-0.411	1.299
Progress monitoring	0.173	0.071	0.274	-0.151	0.497
Small-group	0.376	0.314	0.438	-0.215	0.967
Comprehension	0.238	0.179	0.297	-0.170	0.646
Decoding	0.290	0.228	0.352	-0.205	0.785
Fluency	0.258	0.186	0.329	-0.209	0.725
Multiple reading	0.272	0.211	0.333	-0.206	0.750
Spelling/Writing	0.317	0.220	0.413	-0.196	0.830
Vocabulary	0.200	0.143	0.258	-0.146	0.546
Algebra	0.148	0.007	0.289	-0.226	0.522
Fractions	0.501	0.146	0.857	-0.769	1.771
Geometry	0.169	0.092	0.246	-0.226	0.564
Multiple math	0.281	0.203	0.358	-0.222	0.784
Number sense	0.324	0.234	0.414	-0.178	0.826
Operations	0.292	0.215	0.370	-0.130	0.714
Problem solving	0.328	0.176	0.480	-0.206	0.862
General academic	0.213	0.050	0.375	-0.338	0.764
Meta-cognitive	0.242	0.153	0.331	-0.197	0.681
Social-emotional	0.241	-0.135	0.618	-0.860	1.342

Table K2. Subgroup analyses: Effect sizes. confidence intervals. and prediction intervals by intervention component in single component studies

Component (single)	Avg. ES	95% PI lower bound	95% PI upper bound	95% PI lower bound	95% PI upper bound
CAI	0.128	0.018	0.239	-0.170	0.426
Coaching	-0.047	-0.878	0.783	-5.510	5.416
Incentives	0.046	-0.086	0.178	-0.695	0.787
Medium-group	0.091	-0.092	0.274	-0.434	0.616
Peer-assisted	0.387	0.257	0.518	0.034	0.740
Progress monitoring	0.277	-3.085	3.638		
Small-group	0.375	0.304	0.446	-0.206	0.956
Comprehension	0.205	-0.409	0.819	-3.703	4.113
Decoding	0.305	0.145	0.465	-0.508	1.118
Number sense	0.510	0.143	0.877	-0.529	1.549
Operations	0.165	-0.149	0.479	-3.154	3.484
